# Tricarbonyl rhenium(i) complexes with 8-hydroxyquinolines: structural, chemical, antibacterial, and anticancer characteristics†

**DOI:** 10.1039/d4ra03141e

**Published:** 2024-06-05

**Authors:** Krzysztof Łyczko, Anna Pogorzelska, Urszula Częścik, Mirosława Koronkiewicz, Joanna E. Rode, Elżbieta Bednarek, Robert Kawęcki, Karolina Węgrzyńska, Anna Baraniak, Małgorzata Milczarek, Jan Cz. Dobrowolski

**Affiliations:** a Institute of Nuclear Chemistry and Technology Dorodna 16 03-195 Warsaw Poland k.lyczko@ichtj.waw.pl j.rode@ichtj.waw.pl; b National Medicines Institute Chełmska 30/34 00-725 Warsaw Poland m.koronkiewicz@nil.gov.pl a.baraniak@nil.gov.pl j.dobrowolski@nil.gov.pl; c Faculty of Science, University of Siedlce 3 Maja 54 08-110 Siedlce Poland

## Abstract

Twelve tricarbonyl rhenium(i) complexes in the ‘2 + 1’ system with the anionic bidentate N,O-donor ligand (deprotonated 8-hydroxyquinoline (HQ) or its 2-methyl (MeHQ) or 5-chloro (ClHQ) derivative) and neutral N-donor diazoles (imidazole (Him), 2-methylimidazole (MeHim), 3,5-dimethylpyrazole (Hdmpz), and 3-phenylpyrazole (HPhpz)) were synthesized: [Re(CO)_3_(L_N,O_)L_N_] (L_N,O_ = Q^−^, MeQ^−^, ClQ^−^; L_N_ = Him, MeHim, Hdmpz, HPhpz). Their crystal structures were determined by the scXRD method, compared with the DFT-calculated ones, and characterized by analytical (EA) and spectroscopic techniques (FT-IR, NMR, and UV-Vis) interpreted with DFT and TD-DFT calculations. Most of the Re(i) complexes did not show relevant antibacterial activity against Gram-negative and Gram-positive bacterial strains. Only [Re(CO)_3_(MeQ)Him] demonstrated significant action 4-fold better against Gram-negative *Pseudomonas aeruginosa* than the free MeHQ ligand. The cytotoxicity of the compounds was estimated using human acute promyelocytic leukemia (HL-60), ovarian (SKOV-3), prostate (PC-3), and breast (MCF-7) cancer, and breast non-cancerous (MCF-10A) cell lines. Only HQ and ClHQ ligands and [Re(CO)_3_(Q)Hdmpz] complex had good selectivity toward MCF-7 cell line. HL-60 cells were sensitive to all complexes (IC_50_ = 1.5–14 μM). Still, pure HQ and ClHQ ligands were slightly more active than the complexes.

## Abstract 2

Twelve tricarbonyl Re(i) complexes with bidentate 8-hydroxyquinolinato and monodentate diazole ligands were synthesized, characterized by scXRD, IR, NMR, and UV-Vis methods, and checked for antibacterial and anticancer activity.

## Graphical abstract

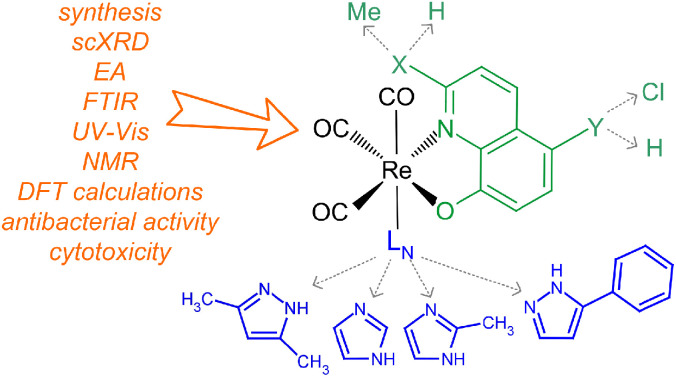

## Introduction

In 2020, the World Health Organization (WHO) listed the following threats resulting from antibiotic resistance (AMR):^1^ “(1) AMR can affect anyone, of any age, in any country; (2) AMR occurs naturally, but misuse of antibiotics in humans and animals is accelerating the process; (3) a growing number of infections – such as pneumonia, tuberculosis, gonorrhea, and salmonellosis – are becoming harder to treat as the antibiotics used to treat them become less effective; (4) AMR leads to longer hospital stays, higher medical costs, and increased mortality”. The WHO describes AMR as “one of the biggest threats to global health, food security, and development today”. Metal complexes are frequently overlooked as potential antibacterial drugs, but recent studies show a significantly higher hit rate against critical pathogens than organic compounds.^2^

On the other hand, numerous metal complexes exhibit potential therapeutic and diagnostic utility in treating cancer, bacterial and fungal infections, and diseases like diabetes, inflammation, cardiovascular, and neurodegenerative disorders.^3–12^ Still, only a few Pt-metallodrugs (cisplatin, carboplatin, oxaliplatin, nedaplatin, lobaplatin, and heptaplatin) have been approved for use in oncology.^13,14^ The systemic toxicity of Pt-metallodrugs and the inherent or acquired resistance of the cancer cells to them is responsible for the underestimation of the potential of anticancer drugs of this type. Therefore, alternative non-platinum (non-Pt) anticancer metallodrugs have been extensively searched since they can offer stereochemical variety and promise to follow modes of action different from those acquired by Pt-resistant cancer cells.^15^ Albeit many non-Pt complexes have been considered for their anticancer activity,^16^ only a few clinical studies of such drugs have been conducted.^17^

This prompted us to search for metalloantibiotics to fight against antimicrobial resistance problems in the group of Re complexes and/or simultaneously for attractive non-Pt anticancer agents. The present work reports studies on tricarbonyl complexes of Re(i) with three 8-hydroxyquinolines.

Tricarbonyl rhenium(i) complexes are the most frequently studied Re compounds, and the 2 + 1 mixed-ligand arrangement of Re(CO)_3_ complexes (one ligand is bidentate, and the other is monodentate) is the most frequently considered.^18–26^ Several investigations showed the potential anticancer activity of Re(CO)_3_, better than used Pt-drugs, as well as their significant antimicrobial effects.^27–33^ On the other hand, 8-hydroxyquinoline and its derivatives are popular organic compounds used as medicines, *e.g.*, clioquinol (5-chloro-8-hydroxy-7-iodoquinoline) exhibits antibacterial and antifungal activity and is used for the treatment of skin infections; nitroxoline (8-hydroxy-5-nitroquinoline) is an antibacterial and anticancer drug; and iodoquinol (5,7-diiodo-8-hydroxyquinoline) is effective in the treatment of amebiasis.^34,35^

Only 16 crystal structures of tricarbonyl rhenium(i) complexes with 8-hydroxyquinolinato ligands have been reported and structurally characterized in the Cambridge Structural Database (CSD, version 5.45).^36^ These include seven crystal structures for 8-hydroxyquinoline,^37–42^ two for 5,7-dimethyl-8-hydroxyquinoline,^43^ one for 5-nitro-8-hydroxyquinoline,^44^ 5-fluoro-8-hydroxyquinoline,^43^ and 5,7-dichloro-8-hydroxyquinoline,^41^ and four for 8-hydroxyquinoline derivatives substituted by diazenyl groups.^45,46^ Structural data for Re(i) complexes with 5-chloro- and 2-methyl-8-hydroxyquinoline have yet to be presented. Nevertheless, cytotoxicity of only [Re(CO)_3_(Q)(PTA)] was evaluated and exhibited a moderate activity against human cervical adenocarcinoma (HeLa) cells higher than for non-cancerous human retinal pigmented epithelial (RPE-1) ones.^41^ Therefore, rhenium complexes are still attractive for searching for new potent non-Pt anticancer agents and metalloantibiotics to fight against antimicrobial resistance problems.

Here, we describe the synthesis, structural, and spectroscopic characteristics of twelve new tricarbonyl rhenium(i) complexes incorporating the bidentate N,O-donor 8-hydroxyquinolines (HQs: unsubstituted HQ, 2-methyl (MeHQ), and 5-chloro (ClHQ) analogs) and an auxiliary 5-membered heterocyclic *N*-donor molecule (imidazole (Him), 2-methyl-imidazole (MeHim), and 3,5-dimethylpyrazole (Hdmpz) or 3-phenylpyrazole (HPhpz)). In addition, their antibacterial effects on Gram-negative and Gram-positive bacterial strains are presented by determining the minimum microbial growth inhibitory concentration (MIC) and minimum bactericidal concentration (MBC). *In vitro* cytotoxicity of the complexes and ligands against human acute promyelocytic leukemia (HL-60), and cancer cell lines such as ovarian (SKOV-3), prostate (PC-3), and breast (MCF-7), as well as normal breast cells (MCF-10A) was also analysed.

## Results and discussion

### Synthesis

**Scheme 1:**
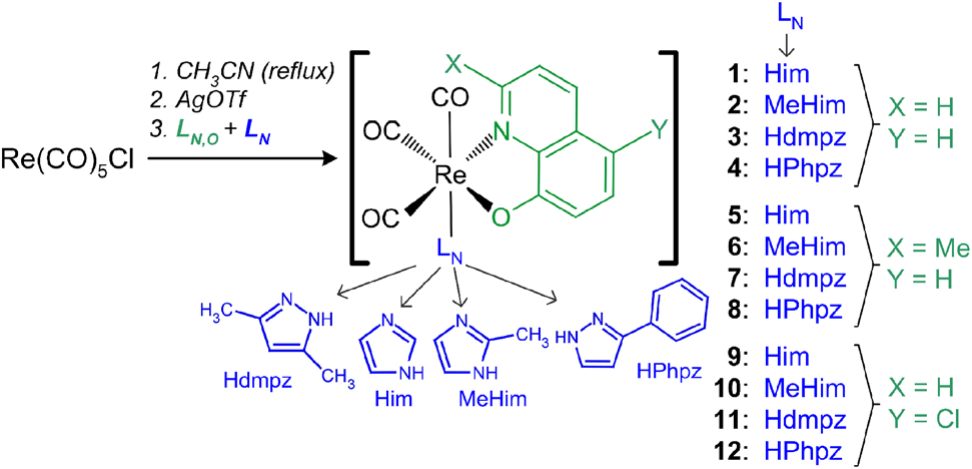
Scheme of the synthesis of the rhenium(i) tricarbonyl complexes 1–12.

The rhenium(i) complexes were synthesized in a three-step process^31,47^ with 27–73% yield (Scheme 1, Table S1†). All reactions were finally made in acetonitrile to avoid the formation of mixed complexes or undesirable forms. Indeed, synthesis of 1 in methanol yields a mixture of brown [Re(CO)_3_(Q)Him] crystals with [Re(CO)_3_(Q)Him]·MeOH solvate (Table S2, Fig. S1a†). Analogously, the synthesis in methanol with the ClHQ ligand gives a mixture of the desired complex with the [Re(CO)_3_(ClQ)MeOH]·MeOH solvate (Table S2, Fig. S1b†). In contrast, in most cases, the synthesis in MeCN eliminates the presence of the co-crystallizing solvates. The solvates were obtained only for 4 and 10: 4·0.5MeCN and 10·MeCN, respectively. In methanol, the 4·MeOH solvate was also formed (Table S2, Fig. S1c†). Surprisingly, the [Re(CO)_3_(Q) MeHim] (2) complex could not be directly isolated from the reaction mixture despite repeated attempts and the use of both solvents. This was achieved only after purification on a silica gel column and further crystallization, as described in the experimental part.

### Molecular and crystal structures

**Fig. 1:**
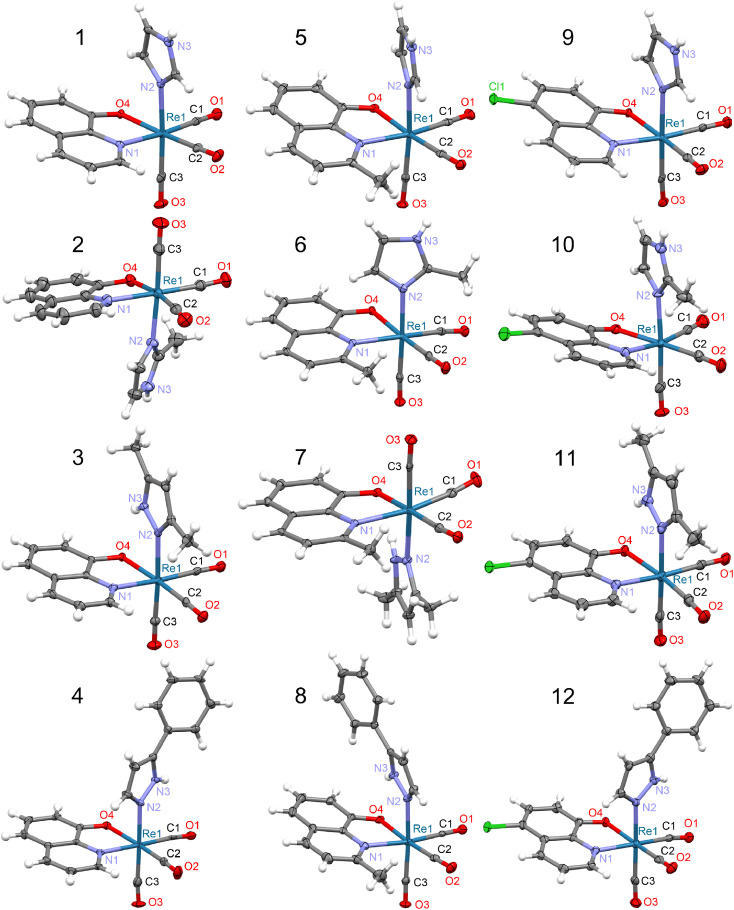
Molecular structures of 8-hydroxyquinolinato rhenium(i) complexes. Thermal ellipsoids are plotted at a 50% probability level. In the case of 10, two crystallographically independent molecules exist in the crystal structure but only one molecule (marked as 1st, see Table S12†) is shown. For 4 and 10, the solvating acetonitrile is omitted.

The molecular structures of 1–12 complexes are shown in Fig. 1, while the selected bond lengths are listed in Tables S3–S14.† The complexes crystallize in the space groups: *P*1̄ (no. 2; 1, 5, 8, 9, and 12), *P*2_1_/*c* (no. 14; 2, 6, 7, and 10), *P*2_1_/*n* (no. 14; 3 and 11), and *C*2/*c* (no. 15; 4). The rhenium(i) ion is always in a slightly distorted octahedral environment with the facial arrangement of the three carbonyl (CO) groups. The Re–C(O) distances vary from 1.893(4) to 1.948(4) Å, with the 1.92 Å mean. An uninegative 8-hydroxyquinolinato bidentate ligand occupies two other positions in a five-membered ring formed through its N and O donor atoms and the Re ion. Moreover, the Q^−^ ligand forms an equatorial plane with two CO groups. The average Re–N1 distance equals 2.17, 2.22, and 2.18 Å for Q^−^, MeQ^−^, and ClQ^−^, respectively. The second distance to chelate, Re–O4, is a bit shorter, and its average value is 2.13, 2.12, and 2.13 Å for Q^−^, MeQ^−^, and ClQ^−^ ions, respectively. The small N1–Re1–O4 bite angles (76.64° on average) are typical for such chelate rings^38–44^ and are the leading cause of the distortion from the octahedral geometry of the metal center. The sixth position in the octahedron is occupied by imidazole (Him or MeHim) or pyrazole (Hdmpz or HPhpz) neutral, monodentate N-donor ligand. The average Re–N distance is 2.20 Å (Him or MeHim), 2.21 Å (Hdmpz), and 2.19 Å (HPhpz).

The DFT calculations reproduced the molecular structures quite well (Fig. S2†), indicating the PBE0 functional, irrespective of the basis set applied (Tables S3–S15†). Significant distortions between experimental and calculated molecular structures were observed only for 4, 8, and 12 structures with the bulky HPhpz ligand, which is the most sensitive to the molecular packing (Fig. S2†).

**Fig. 2:**
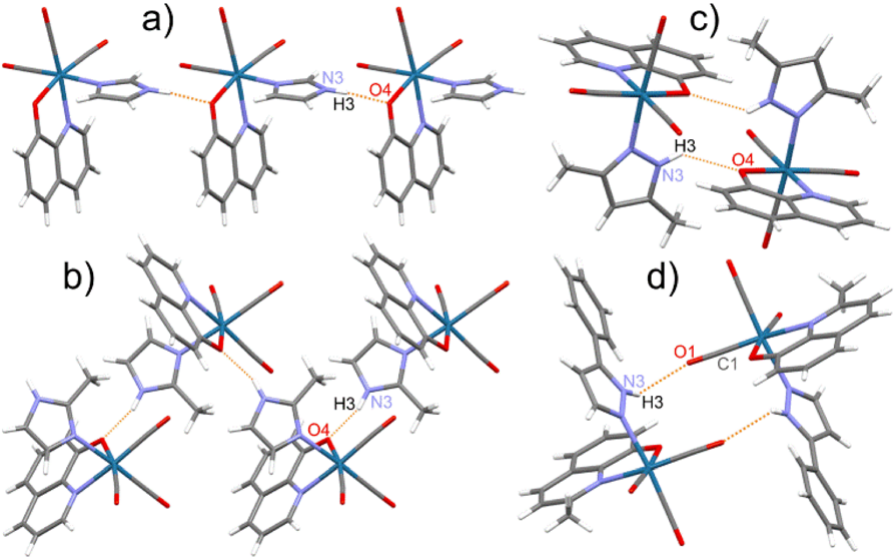
Various motifs of the molecular arrangement in the crystal structure of studied rhenium(i) complexes on the selected examples: (a) linear chain in 1; (b) zigzag chain in 2; (c) dimer with N–H⋯O_(Q)_ bonds in 3; (d) dimer with N–H⋯O_(O=C)_ bonds in 8.

In the crystal structure of studied complexes, the molecules are held together through N–H⋯O intermolecular hydrogen bonds between the O atom of the bidentate ligand and the NH group of the imidazole or pyrazole molecule (Fig. 2a–c). The 8 complex is an exception because in the hydrogen bond the CO group O atom is participating (Fig. 2d). Such H-bonding interactions lead to the formation of chains (1, 2, 5, 9, and 10) or dimeric units (3, 4, 6, 7, 8, 11, and 12) (Fig. 2). Complexes 1, 5, and 9 have similar crystal structures with linear molecular chains, while 2 and 10 form zigzag chains with molecules located around the two-fold screw axes. The N⋯O distances in these hydrogen bonds vary from 2.716(4) to 3.008(3) Å (mean 2.82 Å) with the shortest contact for 4 and the longest for 8 (Table S16†).

The molecular packing accompanied by the chains and dimeric molecular arrangements are shown in Fig S4–S15.† In all crystal structures but 4, dimers and chains are linked together by π⋯π stacking interactions between aromatic rings of the neighbouring 8-hydroxyquinolinates. In most complexes except 4, 10, and 12, the molecular packing is co-stabilized by neighbouring monodentate ligand five-membered rings interactions. Between dimeric motifs, only that in 6 is stabilized by hydrogen bonds and by π⋯π stacking of MeHim ligands (Fig. S9†). In 4, 8, and 12, the five-membered ligands weakly interact with the closest HPhpz benzene rings. All the shortest contacts between the five- and six-membered ring centroids are collected in Table S17.† The 4 and 10 crystals are stabilized by co-crystallized MeCN molecules, which take part in the formation of the C–H⋯N hydrogen bonds and some CH_3_⋯π interactions (Fig. S7 and S13†).

### The prominent spectroscopic features

**Fig. 3:**
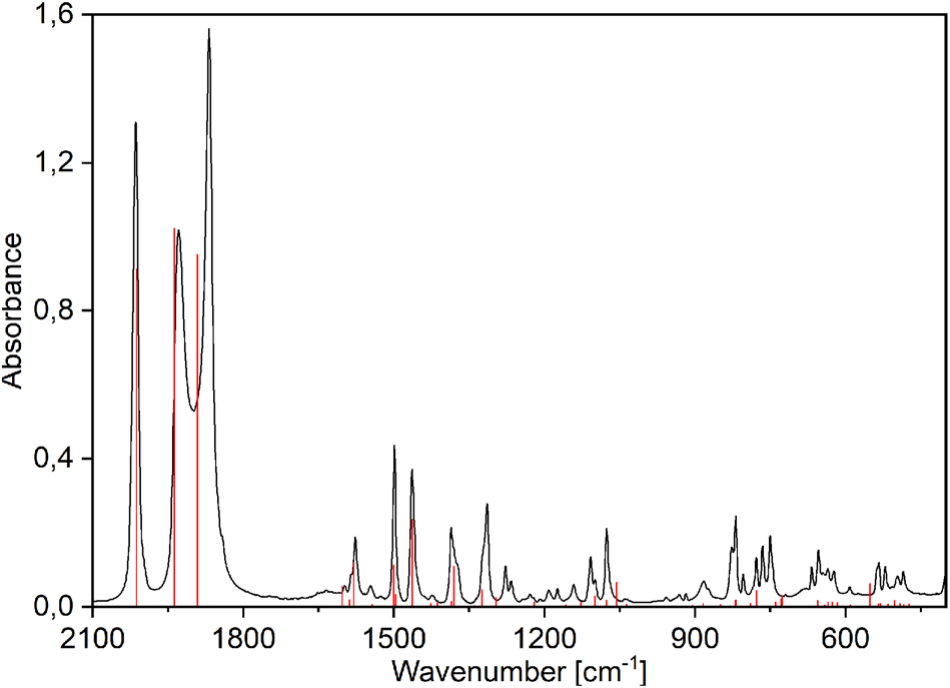
Experimental (black) and PBE0/def2-TZVP simulated (red vertical lines) FTIR spectra of [Re(CO)_3_(Q)Him] (1).

The free ligands broad O–H stretching vibrations band (3400–3000 cm^−1^) disappear in the rhenium(i) complexes spectra due to the complexation of the deprotonated anion (Fig. S16–S30†). The spectra of complexes are dominated by strong CO stretching vibrations' bands characteristic for the tricarbonyl rhenium(i) species observed between 2100 and 1800 cm^−1^ (Fig. 3 and S16–S30†). The first band, at 2008–2021 cm^−1^ is very narrow and is assigned as symmetric stretching vibrations of three carbonyl groups. The other two bands, at 1930–1860 cm^−1^, are ascribed as asymmetric stretching of CO and are much broader and more or less separated. The experimental and calculated IR spectra of complexes 1–12 are superimposed in Fig. S17–S20, S22–S25, and S27–S30.†

**Fig. 4:**
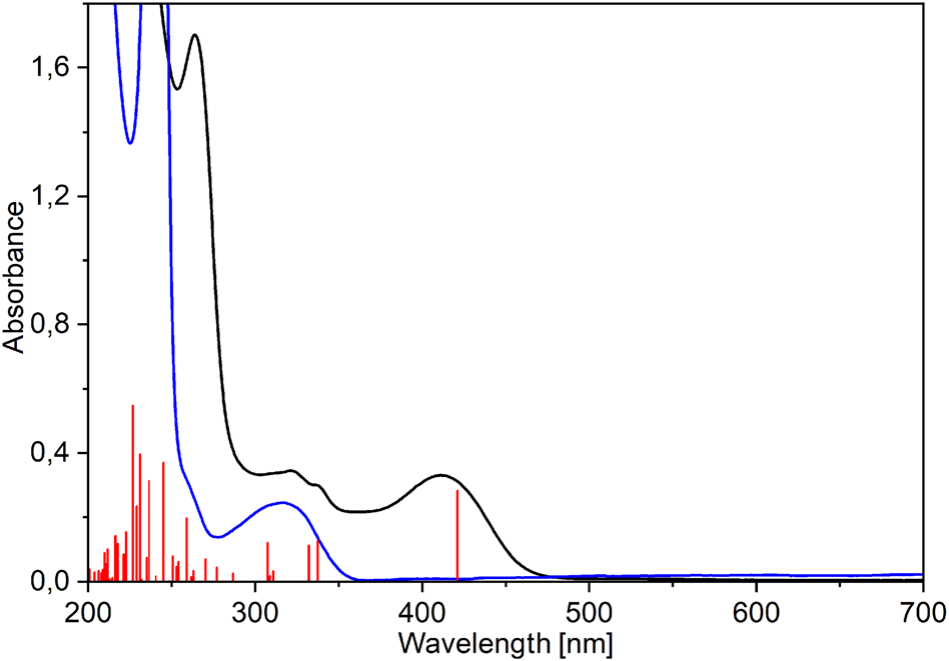
Experimental (black) and TD-PBE0/def2-TZVP/IEFPCM (methanol) simulated (red vertical lines) UV-Vis spectra of [Re(CO)_3_(Q)Him] (1) compared with the spectrum of 8-hydroxyquinoline (blue).

The electronic absorption spectra of pure ligands exhibit strong maxima at 305 (MeHQ), 312 (HQ), and 329 nm (ClHQ). The formation of rhenium(i) complexes is evidenced by the appearance of a band at longer wavelengths: new intense absorptions occur at 409–412, 411–414, and 428–433 nm for MeHQ (5–8), HQ (1–4), and ClHQ (9–12) complexes, respectively (see Fig. 4 for 1 and Fig. S31–S33† for 2–12). As in previous studies,^24,48,49^ the TD-PBE0/def2-TZVP/ECP(Re)/PCM (MeOH) calculated UV-Vis spectra well fitted the experimental ones (Fig. S31–S33†).

**Fig. 5:**
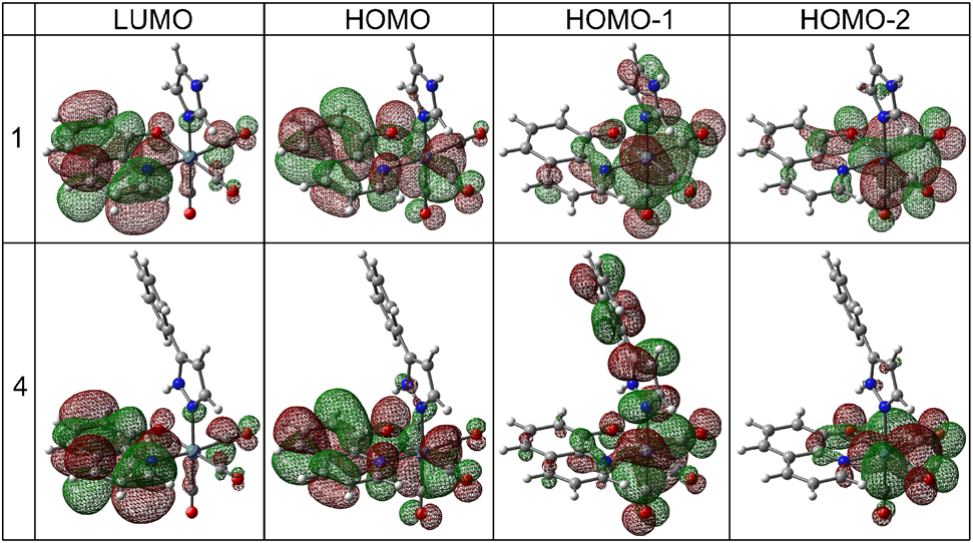
Orbital contours of the lowest energy transitions for the selected tricarbonyl rhenium(i) complexes.

Calculations indicated that the lowest energy transitions involve the promotion of an electron from the HOMO to the LUMO state, which is ligand-centered (LC) π → π* transition (Fig. 5, Table 1 and S18–S30†). Metal-to-ligand (Re → bidentate ligand) and ligand-to-ligand (CO → bidentate ligand) charge transfer dominate the other lowest energy transitions: HOMO-1 → LUMO and HOMO-2 → LUMO (Table S31†). A similar assignment was also reported earlier.^49^ Deeper insight into the computational results shows that the HOMO state consists mainly of bidentate ligand orbitals with a small contribution from metal d-orbital and CO orbitals (Table 1 and S31†). The HOMO-1 and HOMO-2 states have more significant contributions from both metal and CO groups than the HOMO one. In complexes with pyrazoles (3, 4, 7, 8, 11, and 12), the HOMO-1 state also has a distinct contribution from pyrazoles which is the largest for HPhpz. In all complexes, the LUMO state is almost purely antibonding orbital of the bidentate ligand (see 1 and 4 as examples, Fig. 5). Furthermore, energy differences between the HOMO and LUMO levels are in the range of 3.65–3.94 eV, with minimum for the complexes with ClHQ and imidazoles.

**Table:** Compositions (in%) of selected HOMO and LUMO states in the example complexes expressed in respective fragments. L_B_ and L_M_ stand for bidentate and monodentate ligands, respectively^a^

Complex	MO	Re	CO	L_B_	L_M_
1	LUMO	3	3	93	1
	HOMO	12	9	78	1
	HOMO-1	57	30	7	6
	HOMO-2	58	32	9	1
4	LUMO	3	3	93	1
	HOMO	12	9	77	2
	HOMO-1	42	22	6	30
	HOMO-2	60	32	7	1

a Calculations performed at the TD-PBE0/def2-TZVP/ECP(Re)/IEFPCM (methanol) level.

### Stability of the complexes in DMSO

The NMR measurements revealed that the pyrazole ligands (Hdmpz and HPhpz) are slowly replaced with the DMSO solvent molecule. In 3, after two days, an equilibrium between the Hdmpz and DMSO complexes stabilizes at 77% to 23% ratio (Fig. S34†). Complex 3 can be recovered from the mixture by DMSO evaporation and the complex with water is even weaker. In the case of 3-phenylpyrazole in 8, the complex amount drops to 50% immediately after dissolving and after 24 h reaches ratio of 39% to 61% (Fig. S35†). Interestingly, the exchange is not observed for the complexes with imidazoles (Him and MeHim).

**Fig. 6:**
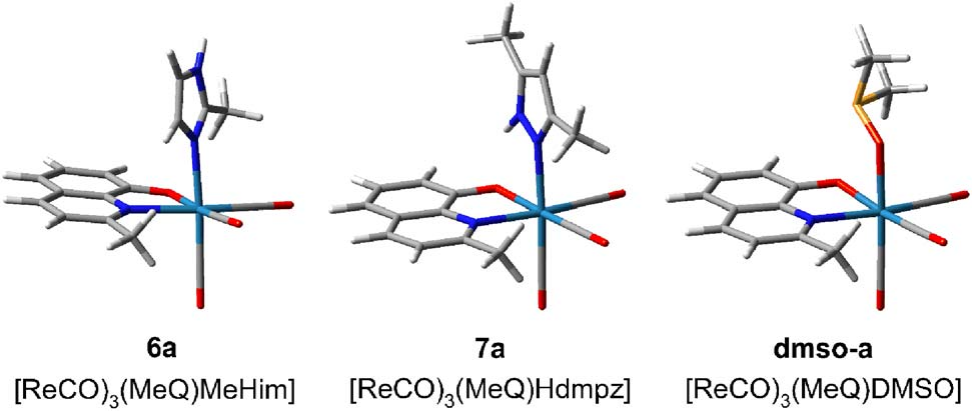
Most stable structures of tricarbonyl rhenium(i) complexes with 2-methyl-8-hydroxyquinolinate (MeO^−^) and 2-methylimidazole (6a, MeHim), 3,5-dimethylpyrazole (7a, Hdmpz), and DMSO (dmso-a) calculated at the B3LYP/aug-cc-pVTZ(N,O,S,C,H)/def2TZVP(Re) level. The remaining structures are shown in Fig. S36.†

To have a deeper insight into the equilibria, model complexes with different positions of 2-methyl-8-hydroxyquinolinate and 2-methylimidazole (6a), 3,5-dimethylpyrazole (7a), or DMSO (dmso-a) were calculated at the B3LYP/aug-cc-pVTZ(N,O,S,C,H)/def2-TZVP(Re) level (Fig. S36†). The most stable are shown in Fig. 6. It appeared that Hdmpz in 7a is additionally stabilized by inter-ligands N–H⋯O hydrogen bond, where NH moiety comes from pyrazole and O from quinoline ligand. Observe that when the nitrogen atoms are separated by the carbon one in the MeHim ligand of 6a, such an inter-ligands hydrogen bond cannot be formed (Fig. 6).

In the case of the DMSO ligand, intermolecular collisions continuously break stabilization between the DMSO methyl group and quinoline's O atom. Ligand binding energies in 6a, 7a, and dmso-a were calculated as the seven-point interaction energy (Δ*E*_7_). The Δ*E*_7_ energy contains the uncorrected interaction energy (Δ*E*), the counterpoise corrected interaction energy (Δ*E*_CP_), the basis set superposition error (BSSE), and the deformation energy (Δ*E*_def_) component, which is especially large (over 10 kcal mol^−1^) for the ligands binding with the Re(CO)_3_ system (Table 2). The Δ*E*_7_ shows that the inter-ligands N–H⋯O hydrogen bond in the pyrazole complex stabilizes it by *ca.* 2 kcal mol^−1^. Also, the DMSO complex is *ca.* 5–7 kcal mol^−1^ weaker than the corresponding diazole complexes. If the 7a complex is the most stable, why does the exchange with DMSO ever occur? The H-bond breaking in 7a by solvent likely produces a considerable change in solvation around the O quinoline and NH pyrazole centers, which the pyrazole dissociation from the complex can follow. In the case of imidazoles, the O quinoline and NH imidazole centers are already solvated, and reorganization of the complex solvation sphere does not occur and is not observed. Finally, notice that the dissociation of bidentate quinoline ligand would require supplying *ca.* 175 kcal mol^−1^ of energy. This could never happen in usual conditions. Therefore, introducing the complex into a biological system cannot release free quinoline ligands, which could act with bacteria or cells separately.

The ligand binding energy expressed as the seven-point interaction energy (Δ*E*_7_) and its components (kcal mol^−1^) for biding the bidentate (MeQ^−^) and monodentate ligands (Hdmpz, dmso, or MeHim) with the Re(CO)_3_ system: uncorrected interaction energy (Δ*E*), counterpoise corrected interaction energy (Δ*E*_CP_), basis set superposition error (BSSE), deformation energy (Δ*E*_def_). The remaining energetics are shown in Table S32

**Table:** 

Interaction energy	Dissociation of monodentate ligand		
	6a	7a	dmso-a
Δ*E*_7_	−20.84	−22.64	−15.89
Δ*E*	−32.26	−35.58	−26.53
Δ*E*_CP_	−31.66	−34.94	−26.02
BSSE	0.59	0.64	0.51
Δ*E*_def_	10.82	12.29	10.13

**Table:** 

Interaction energy	Dissociation of bidentate MeO^−^ ligand		
	6a	7a	dmso-a
Δ*E*_7_	−178.09	−180.54	−172.32
Δ*E*	−190.95	−201.21	−197.00
Δ*E*_CP_	−190.16	−200.39	−196.16
BSSE	0.79	0.82	0.84
Δ*E*_def_	12.07	19.85	23.85

### Antibacterial activity

The MIC and MBC values for the tested 8-hydroxyquinoline ligands (Table 3) showed some antibacterial potency against *Escherichia coli*, *Staphylococcus aureus*, and *Enterococcus faecalis*, which is in line with other studies.^50,51^ Among them, the most considerable inhibitory effects, especially for *E. faecalis*, were demonstrated by ClHQ, which 5-chloro substituent makes the compound more lipophilic and promotes interaction with the lipophilic site of action in the lipid bacterial membranes.^50^ Conceivably, none of the ligands or their complexes, besides 5, had significant activity against *Pseudomonas aeruginosa* due to its exceptional resistance to antibiotics.^52,53^ Although an enhanced antimicrobial activity of various 8-hydroxyquinolines and their metal complexes capable of penetrating bacterial membranes has been reported,^50^ most rhenium(i) complexes tested here did not show a relevant antibacterial action, which often was drastically lower than that of the free ligands (Table 3). Only complex 5 showed 4-fold better activity against *P. aeruginosa* with MIC = 16 mg L^−1^ and MBC = 64 mg L^−1^, than the free MeHQ ligand with activity of 256 mg L^−1^ and >512 mg L^−1^, respectively. The MIC values of 5 for the other microorganisms remained comparable to those obtained for the sole ligand. Interestingly, the MBC of 5 for *E. coli* declined 5-fold, whereas for *E. faecalis* increased 4-fold compared to the free ligand. The antimicrobial activity of MeHQ and its derivatives has been sparingly reported until now.^54,55^

**Table:** MICs/MBCs (mg L^−1^) of the ligands and their tricarbonyl rhenium(i) complexes, and ciprofloxacin (CIP) obtained for reference strains

Compound	*E. coli* ATCC25922	*P. aeruginosa* ATCC27853	*S. aureus* ATCC29213	*E. faecalis* ATCC29212
HQ	32/>512	256/>512	16/64	2/32
1	256/256	128/>512	256/>512	64/>512
2	>512/>512	>512/>512	256/>512	128/>512
3	>512/>512	>512/>512	512/>512	128/512
4	>512/>512	>512/>512	256/>512	32/>512
MeHQ	32/>512	256/>512	32/128	32/32
5	16/16	16/64	16/512	32/>512
6	>512/>512	>512/>512	64/512	64/256
7	>512/>512	>512/>512	>512/>512	128/>512
8	>512/>512	>512/>512	128/>512	32/>512
ClHQ	16/>512	256/>512	8/32	<0.5/<0.5
9	256/256	256/>512	>512/>512	64/>512
10	512/>512	512/>512	64/>512	4/>512
11	512/>512	>512/>512	64/256	64/64
12	>512/>512	>512/>512	128/>512	16/>512
CIP	<0.5/4	1/8	<0.5/1	2/8

### Anticancer activity

The cytotoxicity of the Re(i) complexes with the bidentate N,O-donor ligands has been rarely reported, unlike such compounds with N,N-donor ligands.^56–58^ Moreover, the cytotoxicity of tricarbonyl rhenium(i) complexes was more often tested against solid cancer cells than leukemic cells.^56^

The dose-effect curves for the 8-hydroxyquinoline ligands (HQ, MeHQ, and ClHQ) and their tricarbonyl rhenium(i) complexes in the 100–3.125 μM range were plotted to determine the IC_50_ values in the MTT assay after 24 and 48 hours of treatment (Table 4). The cytotoxicities for studied compounds were evaluated against leukemia cells (HL-60), as well as ovarian (SKOV-3), prostate (PC-3), and breast (MCF-7) cancer cell lines. The potential selectivity of these compounds towards cancer cells was assessed by determination of their cytotoxicities towards breast non-cancerous cells (MCF-10A). For comparison, cisplatin was added to the cytotoxicity assays.

**Table:** Cytotoxicity of the ligands, their tricarbonyl rhenium(i) complexes, and cisplatin toward different cell lines. The data are expressed as IC_50_ ± SD (μM) after 24 and 48 h of incubation time (ND – not determined, * precipitation of the complex)

Compound	HL-60		SKOV-3		PC-3		MCF-7		MCF-10A	
	24 h	48 h	24 h	48 h	24 h	48 h	24 h	48 h	24 h	48 h
HQ	2.2 ± 0.2	2.0 ± 0.2	>100	29 ± 5	18 ± 2	29 ± 3	24 ± 6	7 ± 2	>100	>100
1	4.3 ± 0.2	4 ± 1	36 ± 5	39 ± 3	28 ± 6	27 ± 4	34 ± 4	30 ± 7	39 ± 1	24 ± 1
2	10 ± 1	9 ± 1	>100	44 ± 1	34 ± 3	35 ± 2	36 ± 3	30 ± 1	31 ± 4	29 ± 5
3	7 ± 2	6 ± 1	32 ± 7	31 ± 7	14 ± 2	16 ± 2	19 ± 2	29 ± 5	>100	>100
4	9 ± 1	9 ± 1	>100	81 ± 9	42 ± 6	27 ± 3	73 ± 3	50 ± 3	50 ± 5	36 ± 4
MeHQ	27 ± 2	18 ± 2	>100	>100	62 ± 8	>100	>100	>100	>100	>100
5	11 ± 2	4 ± 1	31 ± 5	26 ± 11	11 ± 3	28 ± 6	47 ± 7	43 ± 6	50 ± 2	>50*
6	14 ± 1	9 ± 1	32 ± 7	30 ± 9	54 ± 7	22 ± 5	44 ± 1	35 ± 6	66 ± 1	35 ± 1
7	13 ± 1	12 ± 1	29 ± 5	26 ± 5	16 ± 2	18 ± 4	27 ± 8	19 ± 4	>25*	>25*
8	9 ± 1	8 ± 1	>100	41 ± 7	39 ± 5	31 ± 5	68 ± 4	53 ± 3	36 ± 1	21 ± 4
ClHQ	1.7 ± 0.1	1.6 ± 0.4	>100	42 ± 7	25 ± 3	39 ± 3	32 ± 8	13 ± 5	>100	70 ± 1
9	11 ± 1	8 ± 2	32 ± 5	28 ± 4	29 ± 4	22 ± 3	58 ± 7	47 ± 6	42 ± 2	24 ± 1
10	5 ± 1	7 ± 1	34 ± 5	32 ± 7	23 ± 2	23 ± 2	54 ± 4	53 ± 4	15 ± 1	12 ± 1
11	5 ± 1	2.8 ± 0.4	40 ± 6	32 ± 7	18 ± 1	24 ± 4	18 ± 5	25 ± 3	24 ± 3	10 ± 1
12	2 ± 1	1.5 ± 0.4	>100	78 ± 12	41 ± 8	23 ± 4	29 ± 3	25 ± 4	34 ± 3	14 ± 1
cisplatin	46 ± 1	ND	>100	>100	58 ± 6	20 ± 2	ND	ND	>100	>100

Only HQ and ClHQ exhibited activity against solid tumor cells among the ligands, while MeHQ remained inactive within the tested range of concentrations (IC_50_ > 100 μM). Compared to pure ligands, most complexes with 8-hydroxyquinolinato ligand and its chloro derivative demonstrated lower activity toward MCF-7 cells. The same holds for the activity of 1, 2, and 4 on the SKOV-3 cell line after 48 h of incubation time. Importantly, the complexes with 2-methyl-8-hydroxyquinolinato ligand showed higher activity against all cell lines than the pure ligand.

Only two tested complexes showed relatively good cytotoxic activity against solid tumor cell lines (IC_50_ < 20 μM), measured after both incubation times. For 3 (IC_50/24_ = 14(2) and IC_50/48_ = 16(2) μM) and 7 (IC_50/24_ = 16(2) and IC_50/48_ = 18(4) μM) cytotoxicity towards PC-3 cells was the highest. In turn, 5 exhibited a higher effect on the same cell line after 24 h (IC_50_ = 11(3) μM) but about 2.5 times lower after 48 h (IC_50_ = 28(6) μM). The remaining complexes showed cytotoxicity ranging from 22(3) to 54(7) μM against PC-3 cells.

Most complexes displayed similar cytotoxicity on the MCF-7 (tumor) and MCF-10A (non-cancerous) cell lines. Still, for some complexes, *e.g.*, 9 and 10, the effect on the cancer cell line was lower than on normal cells, indicating a lack of selectivity towards tumor cells. However, good selectivity was observed for 3 (IC_50_ values of 19(2) μM (24 h) and 29(5) μM (48 h) on MCF-7 and IC_50_ > 100 μM on MCF-10A). In addition, HQ and ClHQ ligands also had a good selectivity as their IC_50_ for normal cells was at least 3 times higher than for breast cancer cells. Nevertheless, for the MCF-10A cell line, complexes 5 and 7 showed lower solubility and precipitated at higher concentrations.

Significant results were obtained for the HL-60 leukemia cells exhibiting high sensitivity to all complexes, with IC_50_ ranging from 1.5(0.4) to 14(1) μM. Even better cytotoxicity on HL-60 cells was obtained for free HQ (IC_50_ = 2 μM) and ClHQ (IC_50_ < 2 μM) ligands. These activities were higher than cisplatin (IC_50/24_ = 46(1) μM). Moreover, cisplatin showed similar activity to the Re(i) complexes on PC-3 cells (after 48 h of incubation) and no action on SKOV-3 and MCF-10A cell lines.

Among the tested rhenium(i) compounds, 3 and 11 had the highest cytotoxicity against all tested cancer cell lines. Complex 11 also showed a similar effect on normal cells (MFC-10A), while 3 had no activity against them.

## Conclusions

Twelve new tricarbonyl rhenium(i) complexes were synthesized and characterized using elemental analyses, single-crystal X-ray diffraction, and molecular spectroscopy (IR, UV-Vis, NMR) methods supported by DFT and TD-DFT calculations. The three-step synthesis led to the formation of neutral [Re(CO)_3_(L_N,O_)L_N_] complexes (1–12) where L_N,O_ is an anionic bidentate ligand, *i.e.*, deprotonated 8-hydroxyquinoline (HQ) and its 2-methyl- (MeHQ) and 5-chloro (ClHQ) derivatives, whereas L_N_ is a neutral monodentate N-donor diazole: imidazole (Him), 2-methylimidazole (MeHim), dimethylpyrazole (Hdmpz) and 3-phenylpyrazole (HPhpz). The preparation was carried out in acetonitrile instead of methanol to eliminate the presence of co-crystallizing solvates or undesirable forms ([Re(CO)_3_(ClQ)MeOH]·MeOH). Still, two solvates with MeCN were obtained: ([Re(CO)_3_(Q)HPhpz]·0.5MeCN and [Re(CO)_3_(ClQ)MeHim]·MeCN). Typically, all complexes contain a slightly distorted octahedral configuration around the Re atom composed of the CO groups in a facial arrangement, the chelating N,O-donor ligand, and the monodentate N-donor molecule. The molecular packing in the crystal structure of the complexes, accompanied by the H-bonded chains or dimers, is stabilized by the formation of π⋯π stacking interactions between neighbouring rings of molecules. A very good agreement was attained between the experimental and DFT-optimized molecular structures. The measured and simulated spectra for the IR and UV-Vis ranges were also reasonably congruent. Furthermore, TD-DFT calculations allowed the proper description of the lowest-lying electronic transitions. The HOMO → LUMO transition is mainly the ligand-centered (LC) π → π* transition. The metal-to-ligand (Re → bidentate ligand) and ligand-to-ligand (CO → bidentate ligand) charge transfers occur between the HOMO-*n* (*n* = 1, 2) and LUMO states.

The NMR measurements revealed that the pyrazole, but not imidazole, ligands in the complexes are slowly replaced with the solvent molecule, and equilibria between pyrazole and DMSO stabilize after one to two days.

The MIC and MBC values of pure ligands showed some antibacterial potency against *E. coli*, *S. aureus*, and *E. faecalis* with 5-chloro-8-hydroxyquinoline offering the highest inhibitory effect. None of the ligands had significant activity against *P. aeruginosa*. Most of the tested rhenium(i) compounds did not show a relevant antibacterial activity, except complex 5, which was 4-fold more active against *P. aeruginosa* (MIC = 16 mg L^−1^; MBC = 64 mg L^−1^) than the ligand alone. In most cases, the antibacterial action of the complex was lower than that of the free ligand.

The cytotoxicity of the studied compounds was evaluated against human acute promyelocytic leukemia (HL-60) and cancer cell lines such as ovarian (SKOV-3), prostate (PC-3), and breast (MCF-7), and non-cancerous breast cells (MCF-10A). Among the ligands, only HQ and ClHQ exhibited activity against solid tumor cells, while MeHQ did not show action at studied concentrations (IC_50_ > 100 μM). Only the complexes with 2-methyl-8-hydroxyquinolinato ligand showed higher activity towards all cell lines than the pure ligand.

Significant results were obtained for the HL-60 leukemia cells exhibiting high sensitivity to all complexes (IC_50_ = 1.5–14 μM), and even better to free HQ (IC_50_ = 2 μM) and ClHQ (IC_50_ < 2 μM) ligands.

## Experimental

### Materials and instruments

The chemicals were purchased from Sigma-Aldrich and only 5-chloro-8-hydroxyquinoline from TCI. Their purity was 98 or 99% (for 3-phenylpyrazole – 97%). Anhydrous solvents with HPLC grade (≥99.9%) were applied. All chemicals were used without further purification. Elemental analysis was performed on an Elementar Vario EL III analyzer. UV-Vis spectra of methanol solutions were recorded in the 200–900 nm range with a Jasco V-750 spectrometer. Infrared absorption spectra in the 400–4000 cm^−1^ range were recorded with a Thermo Scientific Nicolet iS10 FT-IR spectrometer using KBr pellets. ^1^H and ^13^C NMR spectra of DMSO-d_6_ or CDCl_3_ solutions were recorded at 25 °C on a Varian VNMRS-500 or Varian 400MR spectrometer operated at 499.8 and 400 MHz, respectively. The NMR spectra were referenced to the internal reference of tetramethylsilane (TMS).

Bacterial strains and cell lines were obtained from the American Type Culture Collection (ATCC).^59^ All compounds analyzed for antibacterial and anticancer activity were dissolved in dimethylsulfoxide (DMSO, Merck). Antimicrobial activity was tested in Müeller Hinton (MH) II broth (Becton Dickinson). For anticancer activity assay, the following reagents from Merk were used: 3-(4,5-dimethylthiazol-2-yl)-2,5-diphenyltetrazolium bromide (MTT), sodium dodecyl sulfate (SDS) and HCl. The solubilized formazan product was spectrophotometrically quantified in a Power Wave XS (Bio Tek, Winooski, VT, USA) microplate reader. The HL-60 and PC-3 cells were grown in RPMI-1640 medium with stable glutamine (Biowest) supplemented with 10% (v/v) of heat-inactivated fetal bovine serum (FBS, Biowest) and 1% (v/v) of antibiotic–antimycotic solution (Biowest). The SKOV-3 cells were grown in McCoy's 5A medium with l-glutamine (Biowest) supplemented with 10% (v/v) heat-inactivated FBS, and 1% (v/v) antibiotic–antimycotic solution. The MCF-7 cells were grown in Eagle's MEM (Minimal Essential Medium) medium with stable glutamine (Biowest), supplemented with 10% (v/v) heat-inactivated FBS 1% (v/v) MEM non-essential amino acids (Biowest), 1% (v/v) antibiotic–antimycotic solution, and 1 mM sodium pyruvate (Merck). The MCF-10A cells were grown in Ham's F-12K (Kaighn's) Medium with stable glutamine (Gibco) supplemented with 5% (v/v) heat-inactivated FBS, 1% (v/v) MEM non-essential amino acids, 1% (v/v) antibiotic–antimycotic solution, 5 μg mL^−1^ insulin, 0.04 μg mL^−1^ hydrocortisone (Merck), and 15 ng mL^−1^ human Epidermal Growth Factor (hEGF, PeproTech).

### Synthesis of the complexes

Re(CO)_5_Cl (40 mg, 0.111 mmol) was dissolved for 4 h under reflux in acetonitrile (MeCN) (4 mL). Next, an equimolar amount of AgOTf dissolved in a small volume (0.5 mL) of MeCN was added, and the mixture was stirred and heated for approximately 2 h. After separating an AgCl precipitate through a syringe filter (0.45 μm PTFE), the rhenium(i) precursor solution was used in all further syntheses. A mixture of the 8-hydroxyquinolines [HQ (18 mg, 0.124 mmol), MeHQ (20 mg, 0.126 mmol) or ClHQ (22 mg, 0.122 mmol)] and one of the heterocyclic compounds [Him (10 mg, 0.147 mmol), MeHim (12 mg, 0.146 mmol), Hdmpz (13 mg, 0.135 mmol) or HPhpz (23 mg, 0.159 mmol)] in MeCN (1 mL) was added to the rhenium(i) solution. The whole was stirred and heated under reflux for about 24 h. The resulting brown solution was cooled, filtered through a syringe filter, and stored at room temperature (5, 7, 10, and 11) or in a refrigerator (1, 3, 4, 6, 8, 9, and 12). Brown or dark yellow crystals were isolated after a few weeks. Due to the lack of crystallization of 2, its solution was treated as described below.

#### [Re(CO)_3_(Q)Him] (1)

Brown crystals were obtained. Yield: 23 mg (43%). Anal. calc. for C_13_H_8_N_2_O_3_Re: C 37.34, H 2.09, N 8.71. Found: C 37.42, H 2.17, N 8.70%. UV-Vis (methanol) *λ*_max_/nm (*ε*/M^−1^ cm^−1^): 225 (25 500), 265 (20 300), 310 (3900), 337 sh (2900), 411 (3300). IR (KBr) *ν*/cm^−1^: 2015 s (CO), 1929 m (CO), 1868 *vs.* (CO), 1499 w, 1464 w. ^1^H NMR (499.80 MHz, DMSO-d_6_) *δ*/ppm: 12.69 (1H, br s, NH (Him)), 8.99 (1H, dd, *J* = 4.8, 1.4 Hz, H4), 8.44 (1H, dd, *J* = 8.4, 1.4 Hz, H6), 7.83 (1H, m, H13 (Him)), 7.55 (1H, dd, *J* = 8.4, 4.8 Hz, H5), 7.41 (1H, dd, *J* = 7.9, 7.9 Hz, H9), 7.07 (1H, m, H15 (Him)), 6.99 (1H, dd, *J* = 8.0, 0.8 Hz, H8), 6.90 (1H, dd, *J* = 7.9, 0.9 Hz, H10), 6.81 (1H, m, H14 (Him)). ^13^C NMR (125.69 MHz, DMSO-d_6_) *δ*/ppm: 199.4 (CO), 198.6 (CO), 198.0 (CO), 168.9, 149.4, 142.9, 139.2, 138.0, 130.8, 130.7, 127.7, 123.0, 118.3, 115.4, 111.5.

#### [Re(CO)_3_(Q)MeHim] (2)

The dark brown reaction mixture was evaporated under reduced pressure. Then, the obtained solid was purified by means of a silica gel column using a mixture of MeOH and CH_2_Cl_2_ (1 : 20) as a mobile phase. The first yellow-coloured eluate was evaporated giving dark yellow crystalline material. Yield: 40 mg (73%). Anal. calc. for C_16_H_12_N_3_O_4_Re: C 38.71, H 2.44, N 8.46. Found: C 38.98, H 2.72, N 8.59%. UV-Vis (methanol) *λ*_max_/nm (*ε*/M^−1^ cm^−1^): 232 (18 100), 265 (14 000), 321 sh (3300), 338 sh (2800), 414 (2900). IR (KBr) *ν*/cm^−1^: 2014 *vs.* (CO), 1889 *vs.* (CO), 1500 w, 1464 w. ^1^H NMR (499.80 MHz, DMSO-d_6_) *δ*/ppm: 12.39 (1H, br s, NH (MeHim)), 9.09 (1H, dd, *J* = 4.8, 1.5 Hz, H4), 8.43 (1H, dd, *J* = 8.4, 1.5 Hz, H6), 7.57 (1H, dd, *J* = 8.4, 4.8 Hz, H5), 7.36 (1H, dd, *J* = 7.9, 7.9 Hz, H9), 6.96 (1H, dd, *J* = 7.9, 0.9 Hz, H8), 6.86 (1H, t, *J* = 2.1 Hz, MeHim), 6.81 (1H, dd, *J* = 7.9, 0.9 Hz, H10), 6.57 (1H, t, *J* = 1.8 Hz, MeHim), 2.50 (3H, s, MeHim). ^13^C NMR (125.69 MHz, DMSO-d_6_) *δ*/ppm: 199.2 (CO), 198.5 (CO), 197.6 (CO), 168.6, 149.6, 147.7, 143.2, 139.2, 130.8, 130.7, 127.63 123.0, 117.0, 115.1, 111.3, 14.1.

#### [Re(CO)_3_(Q)Hdmpz] (3)

Dark yellow crystals were isolated. Yield: 15 mg (27%). Anal. calc. for C_17_H_14_N_3_O_4_Re: C 40.00, H 2.76, N 8.23. Found: C 40.12, H 2.80, N 8.15%. UV-Vis (methanol) *λ*_max_/nm (*ε*/M^−1^ cm^−1^): 232 (21 600), 264 (17 000), 321 (3500), 337 sh (3000), 412 (3600). IR (KBr) *ν*/cm^−1^: 2013 s (CO), 1893 *vs.* (CO), 1876 s sh (CO). ^1^H NMR (499.80 MHz, CDCl_3_) *δ*/ppm: 11.02 (1H, s, NH (Hdmpz)), 8.98 (1H, dd, *J* = 4.8, 1.5 Hz, H4), 8.21 (1H, dd, *J* = 8.4, 1.4 Hz, H6), 7.46 (1H, dd, *J* = 7.9, 7.9 Hz, H9), 7.36 (1H, dd, *J* = 8.4, 4.8 Hz, H5), 7.15 (1H, dd, *J* = 7.9, 1.1 Hz, H8), 7.02 (1H, dd, *J* = 7.9, 1.1 Hz, H10), 5.74 (1H, m, H14 (Hdmpz)), 2.32 (3H, s, CH_3_ (Hdmpz)), 2.09 (3H, s, CH_3_ (Hdmpz)). ^13^C NMR (125.69 MHz, CDCl_3_) *δ*/ppm: n.r. (CO), 167.4, 152.0, 148.7, 143.3, 140.5, 138.4, 130.7, 130.4, 121.6, 116.3, 113.1, 106.8, 14.5 (CH_3_ (Hdmpz)), 11.0 (CH_3_ (Hdmpz)) (n.r. – not registered due to low solubility).

#### [Re(CO)_3_(Q)HPhpz]·0.5CH_3_CN (4·0.5CH_3_CN)

Brown crystals were obtained. Yield: 20 mg (31%). Anal. calc. for C_44_H_31_N_7_O_8_Re_2_: C 45.63, H 2.70, N 8.47. Found: C 45.62, H 2.61, N 8.49%. UV-Vis (methanol) *λ*_max_/nm (*ε*/M^−1^ cm^−1^): 236 (26 400), 261 (25 900), 320 sh (3600), 337 sh (2600), 411 (3100). IR (KBr) *ν*/cm^−1^: 2018 *vs.* (CO), 1911 m sh (CO), 1881 s (CO), 1500 w, 1465 w, 1319 w. ^1^H NMR (499.80 MHz, CDCl_3_) *δ*/ppm: 11.72 (1H, br s, NH (HPhpz)), 8.96 (1H, dd, *J* = 4.9, 1.5 Hz, H4), 8.21 (1H, dd, *J* = 8.4, 1.5 Hz, H6), 7.67 (1H, t, *J* = 2.1 Hz, HPhpz), 7.50 (1H, dd, *J* = 7.9, 7.9 Hz, H9), 7.39–7.30 (6H, m, H5 + HPhpz), 7.27 (1H, dd, *J* = 8.0, 0.8 Hz, H8), 7.04 (1H, dd, *J* = 8.1, 0.8 Hz, H10), 6.43 (1H, t, *J* = 2.3 Hz, HPhpz). ^13^C NMR (125.69 MHz, CDCl_3_) *δ*/ppm: 197.7 (CO), 196.6 (CO), 196.1 (CO), 167.5, 148.7, 143.6, 143.1, 142.9, 138.4, 130.7, 130.5, 129.7, 129.3, 127.2, 125.3, 121.9, 116.1, 113.4, 104.6.

#### [Re(CO)_3_(MeQ)Him] (5)

Brown crystals were separated. Yield: 23 mg (42%). Anal. calc. for C_16_H_12_N_3_O_4_Re: C 38.71, H 2.44, N 8.46. Found: C 38.92, H 2.50, N 8.62%. UV-Vis (methanol) *λ*_max_/nm (*ε*/M^−1^ cm^−1^): 237 sh (28 700), 271 (27 500), 308 sh (5100), 342 sh (3700), 409 (4200). IR (KBr) *ν*/cm^−1^: 2016 s (CO), 1923 m (CO), 1877 *vs.* (CO), 1564 w. ^1^H NMR (499.80 MHz, DMSO-d_6_) *δ*/ppm: 12.69 (1H, br s, NH (Him)), 8.27 (1H, d, *J* = 8.4 Hz, H6), 7.83 (1H, m, H14 (Him)) 7.55 (1H, d, *J* = 8.4 Hz, H5), 7.30 (1H, dd, *J* = 8.0, 7.8 Hz, H9), 7.07 (1H, m, H16 (Him)), 6.91 (1H, dd, *J* = 8.0, 1.0 Hz, H8), 6.84 (1H, m, H15 (Him)), 6.83 (1H, dd, *J* = 7.8, 1.0 Hz, H10), 3.07 (3H, s, CH_3_ (MeQ)). ^13^C NMR (125.69 MHz, DMSO-d_6_) *δ*/ppm: 198.8 (CO), 198.7 (CO), 197.5 (CO), 168.5, 159.3, 143.1, 139.4, 138.3, 129.4, 129.0, 128.1, 124.0, 118.3, 115.7, 111.7, 29.9 (CH_3_).

#### [Re(CO)_3_(MeQ)MeHim] (6)

Brown crystals were obtained. Yield: 25 mg (45%). Anal. calc. for C_17_H_14_N_3_O_4_Re: C 40.00, H 2.76, N 8.23. Found: C 39.89, H 2.80, N 8.21%. UV-Vis (methanol) *λ*_max_/nm (*ε*/M^−1^ cm^−1^): 235 sh (18 000), 272 (18 600), 309 sh (3500), 342 sh (2000), 412 (2500). IR (KBr) *ν*/cm^−1^: 2008 m (CO), 1882 *vs.* (CO), 1869 s sh (CO), 1566 w, 1433 w, 1326 w. ^1^H NMR (499.80 MHz, DMSO-d_6_) *δ*/ppm: 12.37 (1H, br s, NH (MeHim)), 8.28 (1H, d, *J* = 8.5 Hz, H6), 7.58 (1H, d, *J* = 8.5 Hz, H5), 7.24 (1H, dd, *J* = 8.0, 7.8 Hz, H9), 6.87 (1H, dd, *J* = 8.1, 0.9 Hz, H8), 6.84 (1H, m, H15 (MeHim)), 6.73 (1H, dd, *J* = 7.8, 0.9 Hz, H10), 6.40 (1H, m, H16 (MeHim)), 3.13 (3H, s, CH_3_ (MeQ)), 2.48 (3H, s, CH_3_ (MeHim)). ^13^C NMR (125.69 MHz, DMSO-d_6_) *δ*, ppm: 198.7 (CO), 197.3 (CO), 168.2, 159.2, 147.7, 143.3, 139.4, 129.4, 129.0, 128.2, 124.0, 117.1, 115.3, 111.5, 29.9 (CH_3_ (MeQ)), 14.0 (CH_3_ (MeHim)).

#### [Re(CO)_3_(MeQ)Hdmpz] (7)

Dark yellow crystals were obtained. Yield: 23 mg (40%). Anal. calc. for C_18_H_16_N_3_O_4_Re: C 41.22, H 3.07, N 8.01. Found: C 41.30, H 3.09, N 8.07%. UV-Vis (methanol) *λ*_max_/nm (*ε*/M^−1^ cm^−1^): 271 (26 700), 308 sh (4800), 323 sh (4200), 410 (3800). IR (KBr) *ν*/cm^−1^: 2014 s (CO), 1889 *vs.* (CO), 1434 w. ^1^H NMR (499.80 MHz, CDCl_3_) *δ*/ppm: 11.17 (1H, br s, NH (Hdmpz)), 8.05 (1H, d, *J* = 8.5 Hz), 7.36 (1H, dd, *J* = 7.9, 7.9 Hz), 7.31 (1H, d, *J* = 8.5 Hz), 7.11 (1H, dd, *J* = 7.9, 1.1 Hz), 6.95 (1H, dd, *J* = 7.9, 1.1 Hz, H10), 5.75 (1H, d, *J* = 2.6 Hz, (Hdmpz)), 3.15 (3H, s, CH_3_ (MeQ)), 2.29 (3H, s, CH_3_ (Hdmpz)), 2.09 (3H, s, CH_3_ (Hdmpz)). ^13^C NMR (125.69 MHz, CDCl_3_) *δ*/ppm: 197.7 (CO), 195.7 (CO), 195.3 (CO), 166.9, 159.4, 151.7, 143.3, 140.5, 138.6, 129.1, 128.8, 123.1, 116.5, 113.4, 106.7, 30.3 (CH_3_ (MeQ)), 14.6 (CH_3_ (Hdmpz)), 11.0 (CH_3_ (Hdmpz)).

#### [Re(CO)_3_(MeQ)HPhpz] (8)

Dark yellow crystals were obtained. Yield: 23 mg (37%). Anal. calc. for C_18_H_16_N_3_O_4_Re: C 41.22, H 3.07, N 8.01. Found: C 41.44, H 2.94, N 7.94%. UV-Vis (methanol) *λ*_max_/nm (*ε*/M^−1^ cm^−1^): 243 (31 400), 265 (26 700), 310 sh (4500), 410 (3100). IR (KBr) *ν*/cm^−1^: 2013 s (CO), 1882 *vs.* (CO). ^1^H NMR (400 MHz, CDCl_3_) *δ*/ppm: 11.84 (1H, br s, NH (HPhpz)), 8.04 (1H, d, *J* = 8.3 Hz), 7.67 (1H, t, *J* = 2.1 Hz), 7.41 (1H, t, *J* = 7.9 Hz), 7.40–7.35 (3H, m), 7.34–7.28 (3H, m), 7.23 (1H, dd, *J* = 1.1, 7.8 Hz), 6.97 (1H, dd, *J* = 0.9, 8.0 Hz), 6.45 (1H, t, *J* = 2.3 Hz), 3.13 (3H, s, CH_3_ (MeQ)). ^13^C NMR (100 MHz, CDCl_3_) *δ*/ppm: 197.6 (CO), 195.9 (CO), 195.5 (CO), 167.0, 159.5, 143.5, 143.2, 143.1, 138.7, 129.6, 129.3, 129.2, 128.8, 127.2, 125.3, 123.3, 116.2, 113.7, 104.6, 30.3 (CH_3_ (MeQ)).

#### [Re(CO)_3_(ClQ)Him] (9)

Brown crystals were isolated. Yield: 20 mg (35%). Anal. calc. for C_15_H_9_ClN_3_O_4_Re: C 34.85, H 1.75, N 8.13. Found: C 34.82, H 1.84, N 8.14%. UV-Vis (methanol) *λ*_max_/nm (*ε*/M^−1^ cm^−1^): 240 (21 700), 265 sh (15 600), 329 (3600), 345 sh (3300), 429 (3900). IR (KBr) *ν*/cm^−1^: 2016 s (CO), 1930 m (CO), 1870 *vs.* (CO), 1498 w, 1459 w, 1317 w. ^1^H NMR (499.80 MHz, DMSO-d_6_) *δ*/ppm: 12.72 (1H, br s, NH (Him)), 9.10 (1H, dd, *J* = 4.9, 1.3 Hz, H4), 8.56 (1H, dd, *J* = 8.7, 1.3 Hz, H6), 7.85 (1H, m, H13 (Him)), 7.72 (1H, dd, *J* = 8.7, 4.9 Hz, H5), 7.56 (1H, d, *J* = 8.6 Hz, H9), 7.08 (1H, m, H15 (Him)), 6.88 (1H, d, *J* = 8.6 Hz, H10), 6.81 (1H, m, H14 (Him)). ^13^C NMR (125.69 MHz, DMSO-d_6_) *δ*/ppm: 199.0 (CO), 198.3 (CO), 197.5 (CO), 168.5, 150.3, 143.4, 138.1, 135.6, 130.4, 127.7, 127.7, 124.4, 118.4, 115.1, 112.0.

#### [Re(CO)_3_(ClQ)MeHim]·CH_3_CN (10·CH_3_CN)

Dark yellow crystals were obtained. Yield: 18 mg (29%). Anal. calc. for C_18_H_14_ClN_4_O_4_Re: C 37.80, H 2.47, N 9.80. Found: C 37.50, H 2.35, N 9.48%. UV-Vis (methanol) *λ*_max_/nm (*ε*/M^−1^ cm^−1^): 241 (25 800), 265 sh (17 900), 331 (4200), 346 sh (4000), 433 (4700). IR (KBr) *ν*/cm^−1^: 2013 s (CO), 1905 m (CO), 1878 *vs.* (CO), 1575 w, 1498 w, 1313 w. ^1^H NMR (400 MHz, DMSO-d_6_) *δ*/ppm: 12.44 (1H, br s, H (MeHim)), 9.21 (1H, dd, *J* = 1.2, 4.8 Hz), 8.55 (1H, dd, *J* = 1.2, 8.5 Hz), 7.74 (1H, dd, *J* = 4.8, 8.7 Hz), 7.51 (1H, d, *J* = 8.5 Hz), 6.88 (1H, dd, *J* = 1.8, 2.2 Hz), 6.79 (1H, d, *J* = 8.5 Hz), 6.57 (1H, t, *J* = 1.8 Hz), 2.48 (3H, s, CH_3_ (MeHim)). ^13^C NMR (100 MHz, DMSO-d_6_) *δ*/ppm: 198.4 (CO), 197.7 (CO), 196.6 (CO), 167.8, 150.1, 147.3, 143.2, 135.2, 130.0, 127.2, 127.2, 123.9, 116.7, 114.3, 111.4, 13.6 (CH_3_ (MeHim)).

#### [Re(CO)_3_(ClQ)Hdmpz] (11)

Dark yellow crystals were isolated. Yield: 23 mg (38%). Anal. calc. for C_17_H_13_ClN_3_O_4_Re: C 37.47, H 2.40, N 7.71. Found: C 37.50, H 2.36, N 7.70%. UV-Vis (methanol) *λ*_max_/nm (*ε*/M^−1^ cm^−1^): 236 (24 200), 266 (17 300), 328 (3700), 345 sh (3300), 430 (4100). IR (KBr) *ν*/cm^−1^: 2014 s (CO), 1893 *vs.* sh (CO), 1881 *vs.* sh (CO), 1459 w, 1315 w. ^1^H NMR (499.80 MHz, CDCl_3_) *δ*/ppm: 10.88 (1H, br s, NH (Hdmpz)), 9.03 (1H, dd, *J* = 4.9, 1.4 Hz, H4), 8.56 (1H, dd, *J* = 8.6, 1.4 Hz, H6), 7.52 (1H, d, *J* = 8.5 Hz, H9), 7.48 (1H, dd, *J* = 8.6, 4.9 Hz, H5), 7.05 (1H, d, *J* = 8.6 Hz, H10), 5.76 (1H, d, *J* = 2.6 Hz), 2.31 (3H, s, CH_3_ (Hdmpz)), 2.11 (3H, s, CH_3_ (Hdmpz)). ^13^C NMR (125.69 MHz, CDCl_3_) *δ*/ppm: 197.7 (CO), 196.1 (CO), 195.5 (CO), 166.8, 152.2 (Hdmpz), 149.2, 143.8, 140.7 (Hdmpz), 135.7, 130.0, 128.1, 122.4, 115.7, 115.2, 106.9 (Hdmpz), 14.5 (CH_3_ (Hdmpz)), 11.0 (CH_3_ (Hdmpz)).

#### [Re(CO)_3_(ClQ)HPhpz] (12)

Brown crystals were obtained. Yield: 22 mg (28%). Anal. calc. for C_21_H_13_ClN_3_O_4_Re: C 42.53, H 2.21, N 7.09. Found: C 42.61, H 2.25, N 7.30%. UV-Vis (methanol) *λ*_max_/nm (*ε*/M^−1^ cm^−1^): 242 (37 100), 265 sh (29 200), 324 sh (4400), 344 sh (3700), 428 (4600). IR (KBr) *ν*/cm^−1^: 2021 s (CO), 1916 s sh (CO), 1902 *vs.* (CO), 1499 w, 1458 w, 1316 w. ^1^H NMR (499.80 MHz, CDCl_3_) *δ*/ppm: 11.58 (1H, br s, NH (HPhpz)), 9.01 (1H, dd, *J* = 4.9, 1.4 Hz, H4), 8.56 (1H, dd, *J* = 8.6, 1.4 Hz, H6), 7.66 (1H, t, *J* = 2.1 Hz, HPhpz), 7.56 (1H, d, *J* = 8.5 Hz, H9), 7.49 (1H, dd, *J* = 8.6, 4.9 Hz, H5), 7.42–7.37 (3H, m, HPhpz), 7.34–7.31 (2H, m, HPhpz), 7.17 (1H, d, *J* = 8.5 Hz, H10), 6.44 (1H, t, *J* = 2.1 Hz, HPhpz). ^13^C NMR (125.69 MHz, CDCl_3_) *δ*/ppm: 197.4 (CO), 196.3 (CO), 195.7 (CO), 166.9, 149.2, 143.9, 143.7, 143.0, 135.8, 130.1, 129.8, 129.4, 128.1, 127.1, 125.4, 122.6, 115.5, 115.4, 104.7.

### X-ray crystallography

X-ray diffraction data were measured at 100 K on a Rigaku SuperNova (dual source) four-circle diffractometer equipped with an Eos CCD detector using a mirror-monochromated Mo or Cu Kα radiation (*λ* = 0.71073 or 1.54184 Å) from a microfocus Mova or Nova X-ray source. CrysAlis PRO software was used for data collection, reduction, multi-scan absorption corrections, and other necessary operations. The structures were solved by direct methods and refined by full-matrix least-squares treatment on *F*^2^ data. Non-hydrogen atoms were refined with anisotropic atomic displacement parameters. Hydrogen atoms bonded to C atoms were placed in calculated positions and refined isotropically as a riding model with accepted parameters. The H atoms of NH and OH groups were located from a difference Fourier map, and their positions were freely refined. All calculations were performed using SHELXTL programs^60^ integrated with the OLEX2 crystallographic software.^61^ MERCURY program^62^ was applied for the graphical representation of the molecular and crystal structures. Selected crystallographic data and refinement details are collected in Tables S1 and S2.†

### DFT calculations

DFT calculations were done using the Gaussian 16 program package.^63^ The X-ray structures were the initial geometries for the optimizations which were performed with two hybrid functionals PBE1PBE (equal to PBE0)^64,65^ and B3LYP.^66–68^ The LANL2DZ basis set^69,70^ and effective core potential (ECP) were used for Re,^71^ while the 6-31G(d,p)^72–76^ one for the lighter elements. Furthermore, the def2-TZVP triple-ζ basis set^77^ was applied for all atoms. The results from double- and triple-valence basis sets were compared (Tables S2–S14†). All optimized structures reached the potential energy minima confirmed by solely real harmonic frequencies (further scaled by 0.95 and 0.97 above and below 1800 cm^−1^, respectively). The UV-Vis spectra were calculated with the time-dependent TD-DFT^78^ PBE0/def2-TZVP/ECP(Re) calculations. The solvent effect (methanol) was mimicked using the polarizable continuum model (IEFPCM).^79^ GaussView 5.0 (ref. 80) and GaussSum 3.0 (ref. 81) programs were utilized to visualize and describe the molecular orbitals. The binding energies of monodentate and bidentate ligands in the complexes were estimated at the B3LYP/aug-cc-pVTZ^82,83^/def2-TZVP/ECP(Re) level. The interaction (ligand binding) energies were corrected for the basis set superposition error using the seven-point method, Δ*E*_7_,^84–87^ including the Boys–Bernardi counterpoise correction, Δ*E*_CP_,^84^ and the cage deformation, Δ*E*_def_.

### Antibacterial activity assay

The *in vitro* antibacterial activity was determined according to ISO 20776-1.^88^ Among the tested microorganisms were Gram-negative rods, *E. coli* ATCC25922 and *P. aeruginosa* ATCC27853, and Gram-positive cocci, *S. aureus* ATCC29213 and *E. faecalis* ATCC29212. The selected bacterial species represented the most common etiologic agents of human infections and are used for quality control of antimicrobial susceptibility testing.^89^ The test procedure included the determination of MICs and MBCs of complexes and their free ligands. MIC and MBC determinations of ciprofloxacin, as the reference antibiotic, were also performed. All compounds were dissolved in DMSO to reach the maximum 512 mg L^−1^ concentration. The lack of effect of DMSO on bacterial growth has been verified. The assay consisted of preparing 2-fold dilutions of the substances in a liquid growth medium MH II and inoculating them with a standardized suspension of microorganisms at a McFarland density of 0.5 (≈10^8^ CFU mL^−1^). The culture plates were incubated at 35 °C for 24 h. The lowest concentration of the compound inhibiting visible bacterial growth was specified as MIC, and the concentration that reduced bacterial growth by >99.99% after counting live bacteria on the plates compared to the positive control was defined as MBC.

### Anticancer activity assay

The cells were cultured in 96-well plates and incubated with the tested compound for 24 h and 48 h. All experiments were performed in exponentially growing cultures. The 0.03 M solutions of the compounds in DMSO were appropriately diluted using culture media. The maximal final concentration of DMSO in each well was 0.1%. The cytotoxicity of the compounds was measured by determining cell viability with a colorimetric test based on 3-(4,5-dimethylthiazol-2-yl)-2,5-diphenyltetrazolium bromide (MTT). MTT stock solution was added to each well to a final concentration of 0.5 mg mL^−1^ and incubated for 4 h at 37 °C; following formazan crystals were dissolved by the addition of sodium dodecyl sulfate (SDS) solution (10% SDS in 0.001 M HCl). MTT and SDS were added directly to the cell culture. The compound's IC_50_ values (concentration required to reduce the viability of cells by 50% compared with the control cells) were calculated from the data obtained with the MTT assay.

## Author contributions

Conceptualization, K. Ł., M. K., A. B. and J. Cz. D.; methodology, K. Ł., M. K., J. E. R., A. B. and J. Cz. D.; investigation, K. Ł., A. P., U. C., M. K., J. E. R., E. B., R. K., K. W., A. B., M. M. and J. Cz. D.; resources, K. Ł., M. K., J. E. R., E. B., A. B. and J. Cz. D.; formal analysis, K. Ł., A. P., M. K., J. E. R., E. B., R. K., A. B. and J. Cz. D.; data curation, K. Ł., A. P., M. K., J. E. R., E. B., R. K., K. W., A. B. and J. Cz. D.; writing—original draft preparation, K. Ł., M. K., J. E. R., E. B., R. K., A. B. and J. Cz. D.; writing—review and editing, K. Ł., J. E. R., R. K., A. B., M. M. and J. Cz. D.; visualization, K. Ł. and J. E. R.; supervision, K. Ł. and J. Cz. D.; project administration, J. Cz. D. All authors have read and agreed to the published version of the manuscript.

## Conflicts of interest

There are no conflicts to declare.

## Supplementary Material

RA-014-D4RA03141E-s001

RA-014-D4RA03141E-s002

## References

[cit1] WHO: Antibiotic resistance, https://www.who.int/news-room/fact-sheets/detail/antibiotic-resistance

[cit2] Anthony E. J., Bolitho E. M., Bridgewater H. E., Carter O. W. L., Donnelly J. M., Imberti C., Lant E. C., Lermyte F., Needham R. J., Palau M., Sadler P. J., Shi H., Wang F.-X., Zhang W.-Y., Zhang Z. (2020). Chem. Sci..

[cit3] Englinger B., Pirker C., Heffeter P., Terenzi A., Kowol C. R., Keppler B. K., Berger W. (2019). Chem. Rev..

[cit4] Kumar Singh A., Kumar A., Singh H., Sonawane P., Pathak P., Grishina M., Yadav J. P., Verma A., Kumar P. (2023). Chem. Biodiversity.

[cit5] Frei A., Zuegg J., Elliott A. G., Baker M., Braese S., Brown C., Chen F., Dowson C. G., Dujardin G., Jung N., King A. P., Mansour A. M., Massi M., Moat J., Mohamed H. A., Renfrew A. K., Rutledge P. J., Sadler P. J., Todd M. H., Willans C. E., Wilson J. J., Cooper M. A., Blaskovich M. A. T. (2020). Chem. Sci..

[cit6] Frei A., Elliott A. G., Kan A., Dinh H., Bräse S., Bruce A. E., Bruce M. R., Chen F., Humaidy D., Jung N., Paden King A., Lye P. G., Maliszewska H. K., Mansour A. M., Matiadis D., Paz Muñoz M., Pai T. Y., Pokhrel S., Sadler P. J., Sagnou M., Taylor M., Wilson J. J., Woods D., Zuegg J., Meyer W., Cain A. K., Cooper M. A., Blaskovich M. A. T. (2022). JACS Au.

[cit7] Chukwuma C. I., Mashele S. S., Eze K. C., Matowane G. R., Islam S. M., Bonnet S. L., Noreljaleel A. E. M., Ramorobi L. M. (2020). Pharmacol. Res..

[cit8] Sohrabi M., Binaeizadeh M. R., Iraji A., Larijani B., Saeedi M., Mahdavi M. (2022). RSC Adv..

[cit9] Mucha P., Skoczyńska A., Małecka M., Hikisz P., Budzisz E. (2021). Molecules.

[cit10] Sandhu Q. U. A., Pervaiz M., Majid A., Younas U. (2023). J. Coord. Chem..

[cit11] Bigham N. P., Wilson J. J. (2023). J. Am. Chem. Soc..

[cit12] Ma D.-L., Wu C., Li G., Yung T.-L., Leung C.-H. (2020). J. Mater. Chem. B.

[cit13] Englinger B., Pirker C., Heffeter P., Terenzi A., Kowol C. R., Keppler B. K., Berger W. (2018). Chem. Rev..

[cit14] Oun R. and Wheate N. J., Platinum Anticancer Drugs, in Encyclopedia of Metalloproteins, ed. R. H. Kretsinger, V. N. Uversky and E. A. Permyakov, Springer, New York, NY, 2013, pp. 1710–1714

[cit15] Masternak J., Gilewska A., Barszcz B., Łakomska I., Kazimierczuk K., Sitkowski J., Wietrzyk J., Kamecka A., Milczarek M. (2020). Materials.

[cit16] Meier-Menches S. M., Zappe K., Bileck A., Kreutz D., Tahir A., Cichna-Markl M., Gerner C. (2019). Metallomics.

[cit17] Simpson P. V., Maheshkumar Desai N., Casari I., Massi M., Falasca M. (2019). Future Med. Chem..

[cit18] Muñoz J., Rojas X., Palominos F., Arce R., Cañas F., Pizarro N., Vega A. (2023). Polyhedron.

[cit19] Feng Y., Cheng S.-C., Ko C.-C. (2023). J. Organomet. Chem..

[cit20] Feng W.-W., Liang B.-F., Chen B.-H., Liu Q.-Y., Pan Z.-Y., Liu Y.-J., He L. (2023). Dyes Pigm..

[cit21] Malpicci D., Maver D., Maggioni D., Mercandelli P., Carlucci L., Cariati E., Mussini P., Panigati M. (2023). New J. Chem..

[cit22] Karges J., Giardini M. A., Blacque O., Woodworth B., Siqueira-Neto J. L., Cohen S. M. (2023). Chem. Sci..

[cit23] Stephens L. J., Dallerba E., Kelderman J. T. A., Levina A., Werrett M. V., Lay P. A., Massi M., Andrews P. C. (2023). Dalton Trans..

[cit24] Choroba K., Penkala M., Palion-Gazda J., Malicka E., Machura B. (2023). Inorg. Chem..

[cit25] Jia X., Cui K., Alvarez-Hernandez J. L., Donley C. L., Gang A., Hammes-Schiffer S., Hazari N., Jeon S., Mayer J. M., Nedzbala H. S., Shang B., Stach E. A., Stewart-Jones E., Wang H., Williams A. (2023). Organometallics.

[cit26] Gauthier E. S., Abella L., Caytan E., Roisnel T., Vanthuyne N., Favereau L., Srebro-Hooper M., Williams J. A. G., Autschbach J., Crassous J. (2023). Chem.–Eur. J..

[cit27] Enslin L. E., Purkait K., Pozza M. D., Saubamea B., Mesdom P., Visser H. G., Gasser G., Schutte-Smith M. (2023). Inorg. Chem..

[cit28] Jalilehvand F., Brunskill V., Trung T. S. B., Lopetegui-Gonzalez I., Shemanko C. S., Gelfand B. S., Lin J.-B. (2023). J. Inorg. Biochem..

[cit29] Knopf K. M., Murphy B. L., MacMillan S. N., Baskin J. M., Barr M. P., Boros E., Wilson J. J. (2017). J. Am. Chem. Soc..

[cit30] Schindler K., Cortat Y., Nedyalkova M., Crochet A., Lattuada M., Pavic A., Zobi F. (2022). Pharmaceuticals.

[cit31] Soba M., Scalese G., Casuriaga F., Pérez N., Veiga N., Echeverría G. A., Piro O. E., Faccio R., Pérez-Díaz L., Gasser G., Machado I., Gambino D. (2023). Dalton Trans..

[cit32] Sovari S. N., Golding T. M., Mbaba M., Mohunlal R., Egan T. J., Smith G. S., Zobi F. (2022). J. Inorg. Biochem..

[cit33] Lee L. C.-C., Leung K.-K., Lo K. K.-W. (2017). Dalton Trans..

[cit34] Wykowski R., Fuentefria A. M., de Andrade S. F. (2022). Arch. Microbiol..

[cit35] Chaaban I., Hafez H., AlZaim I., Tannous C., Ragab H., Hazzaa A., Ketat S., Ghoneim A., Katary M., Abd-Alhaseeb M. M., Zouein F. A., Albohy A., Amer A. N., El-Yazbi A. F., Belal A. S. F. (2021). Bioorg. Chem..

[cit36] Groom C. R., Bruno I. J., Lightfoot M. P., Ward S. C. (2016). Acta Crystallogr., Sect. B: Struct. Sci., Cryst. Eng. Mater..

[cit37] Czerwieniec R., Kapturkiewicz A., Anulewicz-Ostrowska R., Nowacki J. (2001). J. Chem. Soc., Dalton Trans..

[cit38] Papagiannopoulou D., Triantis C., Vassileiadis V., Raptopoulou C. P., Psycharis V., Terzis A., Pirmettis I., Papadopoulos M. S. (2014). Polyhedron.

[cit39] Tseng T.-W., Mendiratta S., Luo T.-T., Chen T.-W., Lee Y.-P. (2018). Inorg. Chim. Acta.

[cit40] Vogler A., Bodensteiner M. (2018). Z. Naturforsch., B: J. Chem. Sci..

[cit41] Gantsho V. L., Dotou M., Jakubaszek M., Goud B., Gasser G., Visser H. G., Schutte-Smith M. (2020). Dalton Trans..

[cit42] Priyatharsini M., Mishra I., Shankar B., Srinivasan N., Krishnakumar R. V., Sathiyendiran M. (2021). J. Organomet. Chem..

[cit43] Zhao H. C., Mello B., Fu B.-L., Chowdhury H., Szalda D. J., Tsai M.-K., Grills D. C., Rochford J. (2013). Organometallics.

[cit44] Klenner M. A., Zhang B., Ciancaleoni G., Howard J. K., Maynard-Casely H. E., Clegg J. K., Massi M., Fraser B. H., Pascali G. (2020). RSC Adv..

[cit45] Schutte-Smith M., Muller T. J., Visser H. G., Roodt A. (2013). Acta Crystallogr., Sect. C: Struct. Chem..

[cit46] Sarkar R., Mondal P., Rajak K. K. (2014). Dalton Trans..

[cit47] Lyczko K., Lyczko M., Mieczkowski J. (2015). Polyhedron.

[cit48] Lyczko K., Lyczko M., Meczynska-Wielgosz S., Kruszewski M., Mieczkowski J. (2018). J. Coord. Chem..

[cit49] Corce V., Annereau M., Blanchard S., Dossmann H., Forte J., Gontard G., Martial F., Salmain M. (2023). Appl. Organomet. Chem..

[cit50] Gupta R., Luxami V., Paul K. (2021). Bioorg. Chem..

[cit51] Cherdtrakulkiat R., Boonpangrak S., Sinthupoom N., Prachayasittikul S., Ruchirawat S., Prachayasittikul V. (2016). Biochem. Biophys. Rep..

[cit52] Pang Z., Raudonis R., Glick B. R., Lin T.-J., Cheng Z. (2019). Biotechnol. Adv..

[cit53] Kothari A., Kherdekar R., Mago V., Uniyal M., Mamgain G., Kalia R. B., Kumar S., Jain N., Pandey A., Omar B. J. (2023). Pharmaceuticals.

[cit54] Jeon J.-H., Lee C.-H., Lee H.-S. (2009). J. Korean Soc. Appl. Biol. Chem..

[cit55] Okide G. B., Adikwu M. U., Esimone C. O. (2000). Biol. Pharm. Bull..

[cit56] Bauer E. B., Haase A. A., Reich R. M., Crans D. C., Kühn F. E. (2019). Coord. Chem. Rev..

[cit57] Ho J., Lee W. Y., Koh K. J. T., Lee P. P. F., Yan Y.-K. (2013). J. Inorg. Biochem..

[cit58] Varma R. R., Pursuwani B. H., Suresh E., Bhatt B. S., Patel M. N. (2020). J. Mol. Struct..

[cit59] The American Type Culture Collection (ATCC), available online: https://www.atcc.org

[cit60] Sheldrick G. M. (2015). Acta Crystallogr., Sect. C: Struct. Chem..

[cit61] Dolomanov O. V., Bourhis L. J., Gildea R. J., Howard J. A. K., Puschmann H. (2009). J. Appl. Crystallogr..

[cit62] Macrae C. F., Bruno I. J., Chisholm J. A., Edgington P. R., McCabe P., Pidcock E., Rodriguez-Monge L., Taylor R., van de Streek J., Wood P. A. (2008). J. Appl. Crystallogr..

[cit63] Frisch M. J. , Trucks G. W., Schlegel H. B., Scuseria G. E., Robb M. A., Cheeseman J. R., Scalmani G., Barone V., Petersson G. A., Nakatsuji H., Li X., Caricato M., Marenich A. V., Bloino J., Janesko B. G., Gomperts R., Mennucci B., Hratchian H. P., Ortiz J. V., Izmaylov A. F., Sonnenberg J. L., Williams-Young D., Ding F., Lipparini F., Egidi F., Goings J., Peng B., Petrone A., Henderson T., Ranasinghe D., Zakrzewski V. G., Gao J., Rega N., Zheng G., Liang W., Hada M., Ehara M., Toyota K., Fukuda R., Hasegawa J., Ishida M., Nakajima T., Honda Y., Kitao O., Nakai H., Vreven T., Throssell K., Montgomery Jr J. A., Peralta J. E., Ogliaro F., Bearpark M. J., Heyd J. J., Brothers E. N., Kudin K. N., Staroverov V. N., Keith T. A., Kobayashi R., Normand J., Raghavachari K., Rendell A. P., Burant J. C., Iyengar S. S., Tomasi J., Cossi M., Millam J. M., Klene M., Adamo C., Cammi R., Ochterski J. W., Martin R. L., Morokuma K., Farkas O., Foresman J. B. and Fox D. J., Gaussian 16, Revision C.01, Gaussian, Inc., Wallingford CT, 2019

[cit64] Perdew J. P., Burke K., Ernzerhof M. (1996). Phys. Rev. Lett..

[cit65] Adamo C., Barone V. (1999). J. Chem. Phys..

[cit66] Becke A. D. (1993). J. Chem. Phys..

[cit67] Lee C., Yang W., Parr R. G. (1988). Phys. Rev. B: Condens. Matter Mater. Phys..

[cit68] Stephens P. J., Devlin F. J., Chabalowski C. F., Frisch M. J. (1994). J. Phys. Chem..

[cit69] Hay P. J., Wadt W. R. (1985). J. Chem. Phys..

[cit70] Hay P. J., Wadt W. R. (1985). J. Chem. Phys..

[cit71] Andrae D., Haeussermann U., Dolg M., Stoll H., Preuss H. (1990). Theor. Chim. Acta.

[cit72] Ditchfield R., Hehre W. J., Pople J. A. (1971). J. Chem. Phys..

[cit73] Hehre W. J., Ditchfield R., Pople J. A. (1972). J. Chem. Phys..

[cit74] Hariharan P. C., Pople J. A. (1973). Theor. Chim. Acta.

[cit75] Francl M. M., Pietro W. J., Hehre W. J., Binkley J. S., Gordon M. S., DeFrees D. J., Pople J. A. (1982). J. Chem. Phys..

[cit76] Gordon M. S., Binkley J. S., Pople J. A., Pietro W. J., Hehre W. J. (1982). J. Am. Chem. Soc..

[cit77] Weigend F., Ahlrichs R. (2005). Phys. Chem. Chem. Phys..

[cit78] Adamo C., Jacquemin D. (2013). Chem. Soc. Rev..

[cit79] Cancès E., Mennucci B., Tomasi J. (1997). J. Chem. Phys..

[cit80] Dennington II R. D. , Keith T. A. and Millam J. M., GaussView 6.1.1, Copyright, Semichem, Inc., 2000–2016

[cit81] O'Boyle N. M., Tenderholt A. L., Langner K. M. (2008). J. Comp. Chem..

[cit82] Kendall R. A., Dunning T. H., Harrison R. J. (1992). J. Chem. Phys..

[cit83] Woon D. E., Dunning T. H. (1993). J. Chem. Phys..

[cit84] Boys S. F., Bernardi F. (1970). Mol. Phys..

[cit85] Turi L., Dannenberg J. J. (1993). J. Phys. Chem..

[cit86] Rode J. E., Dobrowolski J. Cz. (2002). Chem. Phys. Lett..

[cit87] Dobrowolski J. Cz., Ostrowski S. (2020). Symmetry.

[cit88] ISO 20776-1:2019: Susceptibility Testing Of Infectious Agents and Evaluation of Performance of Antimicrobial Susceptibility Test Devices–Part 1: Broth Micro-Dilution Reference Method For Testing The *In Vitro* Activity of Antimicrobial Agents Against Rapidly Growing Aerobic Bacteria Involved In Infectious Diseases, available online: https://www.iso.org/standard/70464.html

[cit89] European Committee on Antimicrobial Susceptibility Testing, EUCAST, available online: https://www.eucast.org/

