# Supplementary material for: Tricarbonyl rhenium(i) complexes with 8-hydroxyquinolines: structural, chemical, antibacterial, and anticancer characteristics
Source: RSC Adv. 2024 Jun 5;14(25):18080–92. doi: 10.1039/d4ra03141e (PMC11152041; doi:10.1039/d4ra03141e)
Supplement: RA-014-D4RA03141E-s001 [file RA-014-D4RA03141E-s001.pdf]

Electronic Supplementary Material (ESI) for RSC Advances.
This journal is © The Royal Society of Chemistry 2024

Tricarbonyl rhenium(I) complexes with 8-hydroxyquinolines: structure, chemical, antibacterial, and anticancer characteristics

Krzysztof Łyczko,[1,*] Anna Pogorzelska,[2] Urszula Częścik,[2] Mirosława Koronkiewicz,[2,*] Joanna E. Rode,[1*] Elżbieta Bednarek,[2] Robert Kawęcki,[3] Karolina Węgrzyńska,[2] Anna Baraniak,[2,*] Małgorzata Milczarek,[2] Jan Cz. Dobrowolski[2,*]

[1] Institute of Nuclear Chemistry and Technology, Dorodna 16, 03-195 Warsaw, Poland

[2] National Medicines Institute, Chełmska 30/34, 00-725 Warsaw, Poland

[3] Faculty of Science, University of Siedlce, 3 Maja 54, 08-110 Siedlce, Poland

Electronic supplementary information (ESI)

Table of contents

Table S1. Crystallographic data and structure refinement parameters for studied complexes.

Table S2. Crystallographic data and structure refinement parameters for the methanol solvates of the studied tricarbonyl rhenium(I) complexes.

Fig. S1. Molecular structures of the methanol solvates of the studied tricarbonyl rhenium(I) complexes.

Tables S3-S15. Comparison of the selected experimental and calculated geometrical parameters of complexes **1–12**.

Fig. S2. Overlay of the scXRD and calculated molecular structures of Re(I) complexes.

Fig. S3. Molecular structure of [Re(CO)$_3$(ClQ)MeHim]·CH$_3$CN (**10·CH$_3$CN**) complex.

Table S16. Geometric parameters associated with intermolecular N−H···O hydrogen bond for studied rhenium(I) complexes.

Figs S4-S15. Fragments of the crystal structure of complexes **1–12** showing selected intermolecular interactions.

Table S17. The shortest contacts between centroids of the adjacent five- and six-membered rings in the crystal structure of studied complexes.

Figs S16-S30. Experimental and calculated FTIR spectra of ligands and their Re(I) complexes.

Figs S31-S33. Experimental and calculated UV-Vis spectra of ligands and their Re(I) complexes.

Table S18. The selected experimental and TD-DFT calculated absorptions of Re(I) complexes.

Tables S19-S30. Calculated transitions (with a contribution greater than 5%) for complexes **1–12**.

Table S31. Calculated compositions of HOMO and LUMO orbitals.

Fig. S34. ^{1}H NMR spectra showing exchange of pyrazole ligand with DMSO and H$_2$O in **3**.

Fig. S35. ^{1}H NMR spectra showing exchange of pyrazole ligand with DMSO in **8**.

Fig. S36. Structures of Re(I) complexes considered in the BSSE calculations.

Fig. S37. Scheme of the calculation of interaction energies corrected for BSSE.

Table S32. Energetics obtained in the calculations of interaction energies corrected for BSSE.

Figure S38. Atom labeling in tricarbonyl rhenium(I) complexes for NMR assignment.

Figs S39-S50. ^{1}H and ^{13}C NMR spectra of complexes **1–12**.

Table S1. Crystallographic data and structure refinement parameters for the studied tricarbonyl rhenium(I) complexes.

Compound	**1**	**2**	**3**	**4·0.5CH$_3$CN**	**5**	**6**
Chemical formula	$C_{15}H_{10}N_3O_4Re$	$C_{16}H_{12}N_3O_4Re$	$C_{17}H_{14}N_3O_4Re$	$C_{22}H_{15.5}N_{3.5}O_4Re$	$C_{16}H_{12}N_3O_4Re$	$C_{17}H_{14}N_3O_4Re$
Formula weight	482.46	496.49	510.51	579.08	496.49	510.51
λ (Mo/Cu Kα) (Å)	0.71073	1.54184	0.71073	1.54184	1.54184	1.54184
Crystal system	triclinic	monoclinic	monoclinic	monoclinic	triclinic	monoclinic
Space group	$P\,\bar{1}$	$P\,2_1/c$	$P\,2_1/n$	$C\,2/c$	$P\,\bar{1}$	$P\,2_1/c$
a (Å)	7.44080(10)	13.03930(10)	8.1183(2)	10.26090(10)	7.37137(15)	7.23554(4)
b (Å)	8.1893(2)	9.47380(10)	20.2662(4)	17.4450(2)	8.22667(18)	17.95049(11)
c (Å)	12.2524(3)	14.18770(10)	10.2281(2)	23.0438(3)	12.8439(4)	12.33498(10)
α (°)	87.694(2)	90	90	90	104.939(2)	90
β (°)	82.047(2)	115.3400(10)	95.549(2)	93.5550(10)	94.720(2)	93.2445(6)
γ (°)	85.253(2)	90	90	90	93.3929(17)	90
Volume (Å)	736.57(3)	1584.00(3)	1674.92(6)	4116.94(8)	747.38(3)	1599.517(19)
Z	2	4	4	8	2	4
D_{calc} (g·cm^{-3})	2.175	2.082	2.025	1.869	2.206	2.120
μ (mm^{-1})	8.273	15.248	7.282	11.855	16.158	15.123
F (000)	456	944	976	2232	472	976
Crystal size (mm)	0.10 x 0.06 x 0.04	0.10 x 0.08 x 0.04	0.18 x 0.12 x 0.06	0.10 x 0.05 x 0.03	0.10 x 0.06 x 0.02	0.15 x 0.05 x 0.03
θ range (°)	2.497−31.913	3.751−71.000	2.010−31.392	3.844−69.875	3.580−70.541	4.354−70.632
Reflections collected	29580	48018	19122	21389	19126	53161
Unique reflections	4824	3034	5186	3848	2824	3063
Reflections $I > 2\sigma(I)$	4556	2793	4529	3468	2735	2961
R_{int}	0.0366	0.0303	0.0379	0.0495	0.0361	0.0326
Restraints/parameters	0 / 212	0 / 222	0 / 232	0 / 282	0 / 222	0 / 233
Goodness-of-fit	1.077	1.047	1.083	1.036	1.051	1.064
R_1, wR_2 ($I > 2\sigma(I)$)	0.0166, 0.0317	0.0182, 0.0424	0.0258, 0.0488	0.0251, 0.0600	0.0159, 0.0389	0.0126, 0.0300
R_1, wR_2 (all data)	0.0189, 0.0324	0.0212, 0.0441	0.0332, 0.0520	0.0294, 0.0626	0.0167, 0.0394	0.0134, 0.0303
Max. peak/hole(e·Å^{-3})	1.002/−0.805	1.257/−1.022	1.195/−1.614	1.142/−1.631	0.810/−1.041	0.395/−0.527

Table S1. ***Continued.***

Compound	**7**	**8**	**9**	**10·CH$_3$CN**	**11**	**12**
Chemical formula	$C_{18}H_{16}N_3O_4Re$	$C_{22}H_{16}N_3O_4Re$	$C_{15}H_9ClN_3O_4Re$	$C_{18}H_{14}ClN_4O_4Re$	$C_{17}H_{13}ClN_3O_4Re$	$C_{21}H_{13}ClN_3O_4Re$
Formula weight	524.54	572.58	516.90	571.98	544.95	592.99
λ (Mo/Cu Kα) (Å)	0.71073	1.54184	0.71073	1.54184	1.54184	1.54184
Crystal system	monoclinic	triclinic	triclinic	monoclinic	monoclinic	triclinic
Space group	$P\ 2_1/c$	$P\ \bar{1}$	$P\ \bar{1}$	$P\ 2_1/c$	$P\ 2_1/n$	$P\ \bar{1}$
a (Å)	11.5721(3)	7.8950(2)	7.5357(3)	14.2506(3)	7.96094(6)	9.8714(6)
b (Å)	15.9025(5)	10.3914(4)	8.1793(3)	15.7017(3)	19.9948(2)	10.2322(6)
c (Å)	9.6875(3)	12.3893(3)	12.8369(6)	18.1171(4)	11.14750(11)	11.4613(6)
α (°)	90	79.872(3)	85.268(3)	90	90	67.014(5)
β (°)	106.476(3)	84.527(2)	78.455(4)	111.310(3)	95.6676(8)	75.785(5)
γ (°)	90	83.437(3)	84.500(3)	90	90	65.454(6)
Volume (Å)	1709.55(9)	991.08(5)	770.02(6)	3776.68(15)	1765.75(3)	964.62(11)
Z	4	2	2	8	4	2
D_{calc} (g·cm^{-3})	2.038	1.919	2.229	2.012	2.050	2.042
μ (mm^{-1})	7.138	12.294	8.089	14.187	15.112	13.906
F (000)	1008	552	488	2192	1040	568
Crystal size (mm)	0.08 x 0.06 x 0.05	0.20 x 0.10 x 0.04	0.12 x 0.04 x 0.03	0.14 x 0.05 x 0.04	0.06 x 0.04 x 0.03	0.08 x 0.05 x 0.03
θ range (°)	1.835−28.000	3.635−70.699	2.507−27.999	3.329−70.918	4.423−70.398	4.211−70.843
Reflections collected	24923	14084	17146	17396	34082	13414
Unique reflections	4120	3747	3705	7149	3359	3660
Reflections $I > 2\sigma(I)$	3734	3668	3507	6326	3054	3345
R_{int}	0.0403	0.0389	0.0388	0.0298	0.0380	0.0478
Restraints/parameters	0 / 242	0 / 276	0 / 221	1 / 531	0 / 241	0 / 275
Goodness-of-fit	1.087	1.087	1.044	1.035	1.054	1.035
R_1, wR_2 ($I > 2\sigma(I)$)	0.0177, 0.0371	0.0218, 0.0549	0.0182, 0.0410	0.0276, 0.0680	0.0217, 0.0431	0.0289, 0.0635
R_1, wR_2 (all data)	0.0215, 0.0386	0.0224, 0.0552	0.0202, 0.0418	0.0336, 0.0714	0.0253, 0.0444	0.0365, 0.0659
Max. peak/hole(e·Å^{-3})	0.622/−0.851	1.139/−1.122	1.218/−0.786	1.453/−0.985	0.623/−1.148	1.697/−1.388

Table S2. Crystallographic data and structure refinement parameters for the methanol solvates of the studied tricarbonyl rhenium(I) complexes.

Compound	**1·MeOH**	**4·MeOH**	**[Re(CO)$_3$(ClQ)MeOH]·MeOH**
Chemical formula	$C_{16}H_{14}N_3O_5Re$	$C_{22}H_{18}N_3O_5Re$	$C_{14}H_{13}ClNO_6Re$
Formula weight	514.50	590.59	512.90
λ (Mo/Cu Kα) (Å)	0.71073	1.54184	1.54184
Crystal system	triclinic	triclinic	monoclinic
Space group	$P\bar{1}$	$P\bar{1}$	$P\,2_1/c$
a (Å)	8.1187(2)	7.4449(2)	6.45743(12)
b (Å)	9.2533(3)	9.4022(4)	15.9593(3)
c (Å)	11.8350(4)	16.3685(7)	15.6659(2)
α (°)	92.174(3)	74.151(4)	90
β (°)	102.111(3)	77.427(3)	100.5214(16)
γ (°)	92.651(2)	72.802(3)	90
Volume (Å)	867.35(5)	1041.18(7)	1587.32(5)
Z	2	2	4
D_{calc} (g·cm^{-3})	1.970	1.884	2.146
μ (mm^{-1})	7.036	11.761	16.809
F (000)	492	572	976
Crystal size (mm)	0.15 x 0.10 x 0.03	0.20 x 0.12 x 0.10	0.08 x 0.05 x 0.03
θ range (°)	2.206−28.000	2.838−70.398	3.989−70.295
Reflections collected	9447	7486	12.984
Unique reflections	4178	3879	2.986
Reflections $I > 2\sigma(I)$	3914	3794	2.698
R_{int}	0.0360	0.0276	0.0285
Restraints/parameters	0 / 235	3 / 289	1 / 218
Goodness-of-fit	1.013	1.104	1.032
R_1, wR_2 ($I > 2\sigma(I)$)	0.0247, 0.0450	0.0246, 0.0637	0.0192, 0.0427
R_1, wR_2 (all data)	0.0274, 0.0463	0.0253, 0.0641	0.0254, 0.0442
Max. peak/hole(e·Å^{-3})	1.040/−1.375	1.344/−1.749	0.802/−0.675

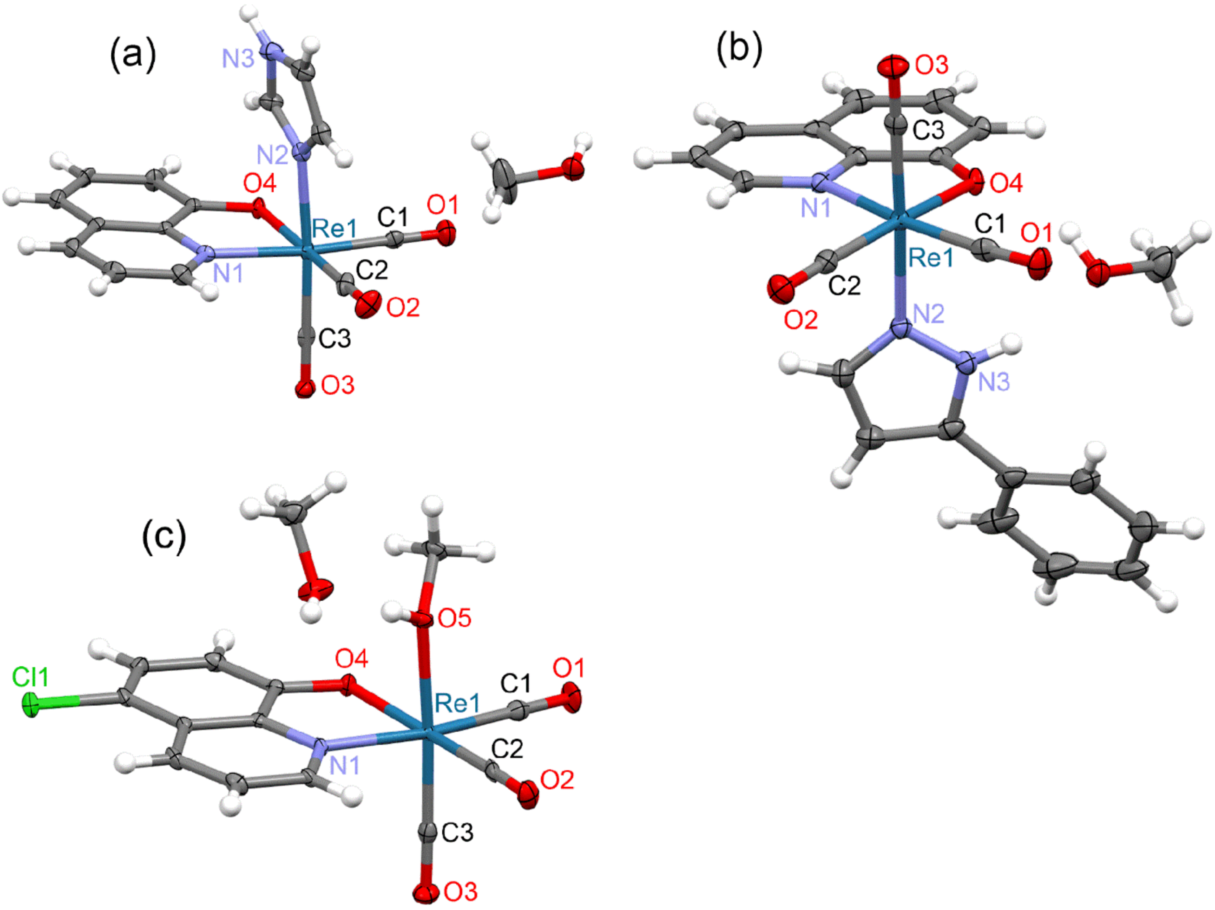

Fig. S1. Molecular structures of rhenium(I) complexes: a) $[Re(CO)_3(Q)Him]$·MeOH (**1·MeOH**), b) $[Re(CO)_3(Q)HPhpz]$·MeOH (**4·MeOH**), and c) $[Re(CO)_3(ClQ)MeOH]$·MeOH. Thermal ellipsoids are plotted at a 50% probability level.

Table S3. Comparison of the experimentally obtained and calculated bond lengths and angles for complex **1** (MAD means the Mean Absolute Deviation).

**Complex 1**		**PBE0 LANL2DZ/6-31G(d,p)**		**PBE0 def2-TZVP**		**B3LYP LANL2DZ/6-31G(d,p)**		**B3LYP def2-TZVP**	
	Exp	Calc	|Exp-Calc|	Calc	|Exp-Calc|	Calc	|Exp-Calc|	Calc	|Exp-Calc|
Re1-C1	1.920	1.921	0.001	1.923	0.003	1.932	0.012	1.938	0.018
Re1-C2	1.909	1.899	0.010	1.902	0.007	1.913	0.004	1.918	0.009
Re1-C3	1.912	1.916	0.004	1.918	0.006	1.927	0.015	1.933	0.021
Re1-N1	2.176	2.184	0.007	2.194	0.018	2.215	0.039	2.223	0.047
Re1-O4	2.126	2.127	0.001	2.120	0.006	2.145	0.019	2.139	0.013
Re1-N2	2.199	2.210	0.011	2.222	0.023	2.247	0.048	2.255	0.056
C11-O4	1.304	1.336	0.032	1.299	0.005	1.311	0.007	1.306	0.002
**MAD**			**0.009**		**0.009**		**0.020**		**0.023**
N1-Re1-O4	76.78	76.40	0.38	75.83	0.95	75.96	0.82	75.40	1.38
N1-Re1-N2	84.81	84.81	0.00	85.33	0.52	85.39	0.58	85.73	0.92
O4-Re1-N2	81.92	81.81	0.11	81.99	0.07	81.95	0.03	82.07	0.15
N1-Re1-C1	173.40	171.82	1.58	173.16	0.24	171.4	2.00	172.24	1.16
O4-Re1-C2	171.99	171.25	0.74	172.25	0.26	170.69	1.30	171.77	0.22
N2-Re1-C3	177.13	175.82	1.31	177.85	0.72	175.94	1.19	177.37	0.24
C1-Re1-C2	88.70	90.99	2.29	88.81	0.11	91.48	2.78	89.68	0.98
C1-Re1-C3	88.75	90.55	1.80	88.70	0.05	90.84	2.09	89.44	0.69
C2-Re1-C3	89.68	90.96	1.28	88.57	1.11	91.38	1.70	89.40	0.28
N2-Re1-C1	93.73	91.77	1.96	92.48	1.25	91.25	2.48	91.81	1.92
N2-Re1-C2	91.82	92.48	0.66	93.24	1.42	92.05	0.23	92.92	1.10
O1-C1-Re1	178.95	179.05	0.10	179.74	0.79	178.7	0.25	179.43	0.48
O2-C2-Re2	177.08	175.58	1.50	177.32	0.24	175.25	1.83	176.89	0.19
O3-C3-Re3	179.44	179.70	0.26	178.92	0.52	179.52	0.08	179.53	0.09
**MAD**			**1.00**		**0.59**		**1.24**		**0.70**
N1-Re1-N2-C13	85.13	88.10	2.97	90.00	4.87	89.28	4.15	89.69	4.56
N1-Re1-N2-C15	-92.73	-93.37	0.64	-92.78	0.05	-92.96	0.23	-93.61	0.88
**MAD**			**1.81**		**2.46**		**2.19**		**2.72**

Table S4. Comparison of the experimentally obtained and calculated bond lengths and angles for complex **2** (MAD means the Mean Absolute Deviation).

**Complex 2**		**PBE0 LANL2DZ/6-31G(d,p)**		**PBE0 def2-TZVP**		**B3LYP LANL2DZ/6-31G(d,p)**		**B3LYP def2-TZVP**	
	Exp	Calc	|Exp-Calc|	Calc	|Exp-Calc|	Calc	|Exp-Calc|	Calc	|Exp-Calc|
Re1-C1	1.922	1.916	0.006	1.918	0.004	1.927	0.005	1.933	0.011
Re1-C2	1.910	1.904	0.006	1.906	0.004	1.917	0.007	1.923	0.013
Re1-C3	1.920	1.916	0.004	1.918	0.002	1.926	0.006	1.932	0.012
Re1-N1	2.172	2.184	0.012	2.194	0.022	2.215	0.043	2.224	0.052
Re1-O4	2.125	2.133	0.008	2.125	0.000	2.151	0.026	2.145	0.020
Re1-N2	2.189	2.218	0.029	2.229	0.040	2.258	0.069	2.265	0.076
C11-O4	1.331	1.303	0.028	1.298	0.033	1.310	0.021	1.305	0.026
**MAD**			**0.013**		**0.015**		**0.025**		**0.030**
N1-Re1-O4	76.64	76.30	0.34	75.74	0.90	75.88	0.76	75.30	1.34
N1-Re1-N2	83.00	83.68	0.68	84.41	1.41	84.28	1.28	84.87	1.87
O4-Re1-N2	83.76	82.41	1.35	82.50	1.26	82.68	1.08	82.72	1.04
N1-Re1-C1	173.49	172.45	1.04	173.78	0.29	171.95	1.54	172.90	0.59
O4-Re1-C2	170.11	171.48	1.37	172.36	2.25	171.04	0.93	171.90	1.79
N2-Re1-C3	176.71	175.41	1.30	177.38	0.67	175.62	1.09	177.06	0.35
C1-Re1-C2	91.36	90.45	0.91	88.31	3.05	90.97	0.39	89.13	2.23
C1-Re1-C3	87.39	90.29	2.90	88.39	1.00	90.65	3.26	89.12	1.73
C2-Re1-C3	90.82	90.67	0.15	88.44	2.38	91.03	0.21	89.25	1.57
N2-Re1-C1	95.77	93.10	2.67	93.83	1.94	92.48	3.29	93.21	2.56
N2-Re1-C2	90.01	92.38	2.37	93.01	3.00	91.97	1.96	92.57	2.56
O1-C1-Re1	177.18	179.03	1.85	179.57	2.39	178.60	1.42	179.53	2.35
O2-C2-Re2	174.96	176.26	1.30	177.68	2.72	175.93	0.97	177.29	2.33
O3-C3-Re3	178.47	179.66	1.19	178.99	0.52	179.46	0.99	179.56	1.09
**MAD**			**1.39**		**1.70**		**1.37**		**1.67**
N1-Re1-N2-C13	-128.09	-125.17	2.92	-126.42	1.67	-123.88	4.21	-124.54	3.55
N1-Re1-N2-C15	43.37	53.94	10.57	54.16	10.79	55.69	12.32	56.23	12.86
**MAD**			**7.44**		**7.08**		**8.95**		**9.04**

Table S5. Comparison of the experimentally obtained and calculated bond lengths and angles for complex **3** (MAD means the Mean Absolute Deviation).

Complex 3		PBE0 LANL2DZ/6-31G(d,p)		PBE0 def2-TZVP		B3LYP LANL2DZ/6-31G(d,p)		B3LYP def2-TZVP	
	Exp	Calc	|Exp-Calc|	Calc	|Exp-Calc|	Calc	|Exp-Calc|	Calc	|Exp-Calc|
Re1-C1	1.922	1.920	0.002	1.922	0.000	1.932	0.010	1.937	0.015
Re1-C2	1.907	1.900	0.007	1.903	0.004	1.913	0.006	1.919	0.012
Re1-C3	1.921	1.915	0.006	1.917	0.004	1.925	0.004	1.931	0.010
Re1-N1	2.171	2.185	0.014	2.195	0.024	2.216	0.045	2.225	0.054
Re1-O4	2.138	2.153	0.015	2.144	0.006	2.173	0.035	2.164	0.027
Re1-N2	2.203	2.214	0.011	2.226	0.023	2.252	0.049	2.261	0.058
C11-O4	1.333	1.315	0.018	1.309	0.024	1.323	0.010	1.315	0.018
**MAD**			**0.011**		**0.012**		**0.023**		**0.028**
N1-Re1-O4	76.62	76.20	0.42	75.63	0.99	75.73	0.89	75.17	1.45
N1-Re1-N2	84.10	86.21	2.11	85.66	1.56	86.79	2.69	87.05	2.95
O4-Re1-N2	80.98	79.84	1.14	79.88	1.10	79.84	1.14	79.96	1.02
N1-Re1-C1	176.30	172.26	4.04	173.37	2.93	171.85	4.45	172.48	3.82
O4-Re1-C2	173.37	172.44	0.93	173.25	0.12	171.73	1.64	172.67	0.70
N2-Re1-C3	175.36	173.58	1.78	175.19	0.17	173.58	1.78	174.77	0.59
C1-Re1-C2	85.70	90.47	4.77	88.47	2.77	90.98	5.28	89.34	3.64
C1-Re1-C3	87.78	90.39	2.61	88.55	0.77	90.67	2.89	89.28	1.50
C2-Re1-C3	86.85	89.65	2.80	87.39	0.54	90.15	3.30	88.25	1.40
N2-Re1-C1	92.86	90.80	2.06	91.67	1.19	90.32	2.54	91.01	1.85
N2-Re1-C2	97.78	96.65	1.13	97.42	0.36	96.18	1.60	96.98	0.80
O1-C1-Re1	176.35	179.08	2.08	179.45	2.45	178.74	1.74	179.26	2.26
O2-C2-Re2	177.00	177.00	1.30	177.70	0.60	176.73	1.57	177.56	0.74
O3-C3-Re3	178.30	179.51	1.21	179.17	0.87	179.28	0.98	179.63	1.33
**MAD**			**2.03**		**1.17**		**2.32**		**1.72**
N1-Re1-N2-C13	93.24	90.68	2.56	92.87	0.37	92.86	0.38	94.04	0.80
N1-Re1-N2-C15	-93.31	-95.22	1.91	-94.06	0.75	-94.09	0.78	-93.58	0.27
**MAD**			**2.23**		**0.56**		**0.58**		**0.54**

Table S6. Comparison of the experimentally obtained and calculated bond lengths and angles for complex **4** (MAD means the Mean Absolute Deviation).

**Complex 4**		**PBE0 LANL2DZ/6-31G(d,p)**		**PBE0 def2-TZVP**		**B3LYP LANL2DZ/6-31G(d,p)**		**B3LYP def2-TZVP**	
	Exp	Calc	|Exp-Calc|	Calc	|Exp-Calc|	Calc	|Exp-Calc|	Calc	|Exp-Calc|
Re1-C1	1.936	1.921	0.015	1.923	0.013	1.932	0.004	1.938	0.002
Re1-C2	1.893	1.901	0.008	1.904	0.011	1.914	0.021	1.920	0.027
Re1-C3	1.925	1.917	0.008	1.919	0.006	1.928	0.003	1.934	0.009
Re1-N1	2.170	2.185	0.015	2.194	0.024	2.216	0.046	2.224	0.054
Re1-O4	2.130	2.151	0.021	2.142	0.012	2.171	0.041	2.162	0.032
Re1-N2	2.199	2.197	0.002	2.208	0.009	2.230	0.031	2.238	0.039
C11-O4	1.335	1.317	0.018	1.310	0.025	1.324	0.011	1.317	0.018
**MAD**			**0.012**		**0.014**		**0.022**		**0.026**
N1-Re1-O4	76.75	76.18	0.57	75.66	1.09	75.74	1.01	75.22	1.53
N1-Re1-N2	85.43	86.46	1.03	86.77	1.34	86.84	1.41	87.01	1.58
O4-Re1-N2	83.19	79.91	3.28	79.93	3.26	79.84	3.35	79.97	3.22
N1-Re1-C1	174.98	172.25	2.73	173.28	1.70	171.81	3.17	172.40	2.58
O4-Re1-C2	171.06	170.69	0.37	171.58	0.52	170.12	0.94	171.07	0.01
N2-Re1-C3	178.59	175.24	3.35	176.97	1.62	175.12	3.47	176.45	2.14
C1-Re1-C2	89.96	90.70	0.74	88.68	1.28	91.18	1.22	89.53	0.43
C1-Re1-C3	85.64	90.31	4.67	88.52	2.88	90.60	4.96	89.24	3.60
C2-Re1-C3	87.37	90.81	3.44	88.66	1.29	91.20	3.83	89.46	2.09
N2-Re1-C1	96.68	91.28	5.40	92.10	4.58	90.88	5.80	91.52	5.16
N2-Re1-C2	93.13	93.65	0.52	94.32	1.19	93.43	0.30	94.01	0.88
O1-C1-Re1	174.62	179.00	4.38	179.33	4.71	178.68	4.06	179.16	4.54
O2-C2-Re2	176.25	176.39	0.14	177.75	1.50	176.07	0.18	177.35	1.10
O3-C3-Re3	175.26	179.77	4.51	179.14	3.88	179.48	4.22	179.67	4.41
**MAD**			**2.51**		**2.20**		**2.71**		**2.38**
N1-Re1-N2-C13	21.72	91.65	69.93	93.38	71.66	92.35	70.63	93.44	71.72
N1-Re1-N2-C15	-163.08	-92.32	70.76	-91.47	71.61	-92.03	71.05	-91.73	71.35
**MAD**			**70.35**		**71.64**		**70.84**		**71.54**

Table S7. Comparison of the experimentally obtained and calculated bond lengths and angles for complex **5** (MAD means the Mean Absolute Deviation).

**Complex 5**		**PBE0 LANL2DZ/6-31G(d,p)**		**PBE0 def2-TZVP**		**B3LYP LANL2DZ/6-31G(d,p)**		**B3LYP def2-TZVP**	
	Exp	Calc	|Exp-Calc|	Calc	|Exp-Calc|	Calc	|Exp-Calc|	Calc	|Exp-Calc|
Re1-C1	1.920	1.913	0.007	1.915	0.005	1.924	0.004	1.930	0.010
Re1-C2	1.923	1.901	0.022	1.903	0.020	1.914	0.009	1.919	0.004
Re1-C3	1.906	1.915	0.009	1.917	0.011	1.925	0.019	1.931	0.025
Re1-N1	2.213	2.236	0.023	2.245	0.032	2.276	0.063	2.283	0.070
Re1-O4	2.116	2.113	0.003	2.106	0.010	2.129	0.013	2.123	0.007
Re1-N2	2.192	2.214	0.022	2.226	0.034	2.251	0.059	2.260	0.068
C11-O4	1.332	1.304	0.028	1.299	0.033	1.311	0.021	1.306	0.026
**MAD**			**0.016**		**0.021**		**0.027**		**0.030**
N1-Re1-O4	76.70	76.40	0.30	75.85	0.85	75.91	0.79	75.37	1.33
N1-Re1-N2	85.11	85.27	0.16	85.57	0.46	85.96	0.85	86.07	0.96
O4-Re1-N2	81.83	81.66	0.17	81.90	0.07	81.85	0.02	81.98	0.15
N1-Re1-C1	169.22	168.89	0.33	170.38	1.16	168.58	0.64	169.59	0.37
O4-Re1-C2	174.25	173.32	0.93	174.48	0.23	172.94	1.31	174.14	0.11
N2-Re1-C3	174.77	176.13	1.36	178.13	3.36	176.27	1.50	177.67	2.90
C1-Re1-C2	87.96	88.55	0.59	86.51	1.45	89.14	1.18	87.46	0.50
C1-Re1-C3	91.31	90.62	0.69	88.71	2.60	90.89	0.42	89.44	1.87
C2-Re1-C3	89.07	91.15	2.08	88.62	0.45	91.55	2.48	89.46	0.39
N2-Re1-C1	93.75	92.05	1.70	92.75	1.00	91.56	2.19	92.09	1.66
N2-Re1-C2	92.43	91.71	0.72	92.65	0.22	91.3	1.13	92.36	0.07
O1-C1-Re1	178.28	178.60	0.32	179.48	1.20	178.31	0.03	179.14	0.86
O2-C2-Re2	178.92	176.90	2.02	178.19	0.73	176.64	2.28	178.13	0.79
O3-C3-Re3	178.21	179.40	1.19	178.79	0.58	179.42	1.21	179.35	1.14
**MAD**			**0.90**		**1.03**		**1.15**		**0.94**
N1-Re1-N2-C13	75.67	91.49	15.82	94.42	18.75	93.25	17.58	94.18	18.51
N1-Re1-N2-C15	-102.37	-90.24	12.13	-88.49	13.88	-89.16	13.21	-89.27	13.10
**MAD**			**13.98**		**16.32**		**15.40**		**15.81**

Table S8. Comparison of the experimentally obtained and calculated bond lengths and angles for complex **6** (MAD means the Mean Absolute Deviation).

**Complex 6**		**PBE0 LANL2DZ/6-31G(d,p)**		**PBE0 def2-TZVP**		**B3LYP LANL2DZ/6-31G(d,p)**		**B3LYP def2-TZVP**	
	Exp	Calc	|Exp-Calc|	Calc	|Exp-Calc|	Calc	|Exp-Calc|	Calc	|Exp-Calc|
Re1-C1	1.904	1.908	0.004	1.910	0.006	1.918	0.014	1.925	0.021
Re1-C2	1.914	1.902	0.012	1.905	0.009	1.915	0.001	1.920	0.006
Re1-C3	1.921	1.916	0.005	1.917	0.004	1.925	0.004	1.931	0.010
Re1-N1	2.225	2.242	0.017	2.249	0.024	2.282	0.057	2.288	0.063
Re1-O4	2.133	2.115	0.017	2.107	0.025	2.129	0.003	2.124	0.008
Re1-N2	2.204	2.222	0.018	2.236	0.032	2.264	0.060	2.275	0.071
C11-O4	1.330	1.301	0.029	1.297	0.033	1.308	0.022	1.304	0.026
**MAD**			**0.015**		**0.019**		**0.023**		**0.029**
N1-Re1-O4	76.30	75.95	0.35	75.51	0.79	75.52	0.78	75.03	1.27
N1-Re1-N2	87.43	86.47	0.96	86.61	0.82	86.99	0.44	87.10	0.33
O4-Re1-N2	81.18	82.80	1.62	82.64	1.46	82.89	1.71	82.80	1.62
N1-Re1-C1	170.42	168.86	1.56	170.13	0.29	168.65	1.77	169.43	0.99
O4-Re1-C2	174.79	176.45	1.66	177.22	2.43	175.66	0.87	176.52	1.73
N2-Re1-C3	177.22	175.37	1.85	177.20	0.02	175.58	1.64	176.88	0.34
C1-Re1-C2	85.78	88.87	3.09	86.96	1.18	89.43	3.65	87.95	2.17
C1-Re1-C3	83.60	90.30	6.70	88.20	4.60	90.70	7.10	89.01	5.41
C2-Re1-C3	88.94	89.80	0.86	82.27	6.67	90.25	1.31	88.11	0.83
N2-Re1-C1	97.21	92.32	4.89	93.48	3.73	91.60	5.61	92.64	4.57
N2-Re1-C2	93.77	94.07	0.30	95.04	1.27	93.55	0.22	94.59	0.82
O1-C1-Re1	174.52	178.76	4.24	179.38	4.86	178.48	3.96	179.18	4.66
O2-C2-Re2	178.19	179.40	1.21	178.80	0.61	179.27	1.08	179.40	1.21
O3-C3-Re3	176.85	179.70	2.85	178.68	1.83	179.65	2.80	179.22	2.37
**MAD**			**2.30**		**2.18**		**2.35**		**2.02**
N1-Re1-N2-C13	164.75	140.97	23.78	139.45	25.30	140.50	24.25	138.66	26.09
N1-Re1-N2-C15	-28.49	-42.04	13.55	-43.02	14.53	-42.85	14.36	-44.21	15.72
**MAD**			**18.67**		**19.92**		**19.31**		**20.91**

Table S9. Comparison of the experimentally obtained and calculated bond lengths and angles for complex **7** (MAD means the Mean Absolute Deviation).

**Complex 7**		**PBE0 LANL2DZ/6-31G(d,p)**		**PBE0 def2-TZVP**		**B3LYP LANL2DZ/6-31G(d,p)**		**B3LYP def2-TZVP**	
	Exp	Calc	|Exp-Calc|	Calc	|Exp-Calc|	Calc	|Exp-Calc|	Calc	|Exp-Calc|
Re1-C1	1.912	1.913	0.001	1.914	0.002	1.924	0.012	1.929	0.017
Re1-C2	1.913	1.902	0.011	1.905	0.008	1.915	0.002	1.921	0.008
Re1-C3	1.906	1.913	0.007	1.915	0.009	1.924	0.018	1.930	0.024
Re1-N1	2.226	2.237	0.011	2.248	0.022	2.277	0.051	2.286	0.060
Re1-O4	2.122	2.139	0.017	2.130	0.008	2.157	0.035	2.148	0.026
Re1-N2	2.226	2.223	0.003	2.233	0.007	2.277	0.051	2.268	0.042
C11-O4	1.335	1.315	0.020	1.309	0.026	1.323	0.012	1.316	0.019
**MAD**			**0.010**		**0.012**		**0.026**		**0.028**
N1-Re1-O4	76.99	76.20	0.79	75.60	1.39	75.68	1.31	75.08	1.91
N1-Re1-N2	86.66	87.36	0.70	87.82	1.16	87.88	1.22	88.10	1.44
O4-Re1-N2	81.21	79.58	1.63	79.75	1.46	79.72	1.49	79.87	1.34
N1-Re1-C1	168.56	169.18	0.62	170.43	1.87	168.85	0.29	169.66	1.10
O4-Re1-C2	177.36	175.56	1.80	176.49	0.87	175.11	2.25	175.98	1.38
N2-Re1-C3	177.53	174.11	3.42	175.71	1.82	174.05	3.48	172.25	5.28
C1-Re1-C2	87.53	87.99	0.46	86.19	1.34	88.61	1.08	87.16	0.37
C1-Re1-C3	85.62	90.46	4.84	88.54	2.92	90.72	5.10	89.26	3.64
C2-Re1-C3	86.15	89.76	3.61	87.39	1.24	90.17	4.02	88.23	2.08
N2-Re1-C1	95.15	90.58	4.57	91.46	3.69	90.26	4.89	90.93	4.22
N2-Re1-C2	96.22	96.08	0.14	96.89	0.67	95.72	0.50	96.51	0.29
O1-C1-Re1	174.21	178.54	4.33	179.06	4.85	178.28	4.07	178.91	4.70
O2-C2-Re2	176.52	179.18	2.66	178.26	1.74	179.54	3.02	178.81	2.29
O3-C3-Re3	177.25	179.72	2.47	179.04	1.79	179.45	2.20	179.57	2.32
**MAD**			**2.29**		**1.92**		**2.49**		**2.31**
N1-Re1-N2-C13	-89.07	-92.68	3.61	-96.74	7.67	-95.32	6.25	-97.64	8.57
N1-Re1-N2-C15	92.17	94.73	2.56	91.21	0.96	92.55	0.38	90.53	1.64
**MAD**			**2.67**		**2.38**		**2.86**		**2.87**

Table S10. Comparison of the experimentally obtained and calculated bond lengths and angles for complex **8** (MAD means the Mean Absolute Deviation).

**Complex 8**		**PBE0 LANL2DZ/6-31G(d,p)**		**PBE0 def2-TZVP**		**B3LYP LANL2DZ/6-31G(d,p)**		**B3LYP def2-TZVP**	
	Exp	Calc	|Exp-Calc|	Calc	|Exp-Calc|	Calc	|Exp-Calc|	Calc	|Exp-Calc|
Re1-C1	1.900	1.913	0.013	1.915	0.015	1.924	0.024	1.930	0.030
Re1-C2	1.918	1.902	0.016	1.904	0.014	1.915	0.003	1.921	0.003
Re1-C3	1.922	1.916	0.006	1.918	0.004	1.927	0.005	1.932	0.010
Re1-N1	2.215	2.240	0.025	2.247	0.032	2.280	0.065	2.285	0.070
Re1-O4	2.105	2.139	0.034	2.129	0.024	2.156	0.051	2.147	0.042
Re1-N2	2.189	2.198	0.009	2.209	0.020	2.231	0.042	2.240	0.051
C11-O4	1.336	1.315	0.021	1.309	0.027	1.322	0.014	1.316	0.020
**MAD**			**0.018**		**0.019**		**0.029**		**0.032**
N1-Re1-O4	77.05	76.03	1.02	75.56	1.49	75.54	1.51	75.07	1.98
N1-Re1-N2	82.22	86.31	4.09	86.76	4.54	86.86	4.64	87.24	5.02
O4-Re1-N2	81.51	80.09	1.42	80.02	1.49	80.00	1.51	80.07	1.44
N1-Re1-C1	169.36	168.92	0.44	170.24	0.88	168.63	0.73	169.50	0.14
O4-Re1-C2	174.22	173.01	1.21	173.78	0.44	172.56	1.66	173.42	0.80
N2-Re1-C3	175.55	175.45	0.10	177.31	1.76	175.38	0.17	176.80	1.25
C1-Re1-C2	87.45	88.21	0.76	86.34	1.11	88.83	1.38	87.29	0.16
C1-Re1-C3	89.04	90.34	1.30	88.48	0.56	90.62	1.58	89.20	0.16
C2-Re1-C3	87.59	90.92	3.33	88.66	1.07	91.31	3.72	89.44	1.85
N2-Re1-C1	95.41	92.13	3.28	92.73	2.68	91.64	3.77	92.07	3.34
N2-Re1-C2	92.75	92.98	0.23	93.81	1.06	92.77	0.02	93.55	0.80
O1-C1-Re1	178.02	178.51	0.49	179.08	1.06	178.28	0.26	178.87	0.85
O2-C2-Re2	178.22	177.84	0.38	178.41	0.19	177.72	0.50	178.50	0.28
O3-C3-Re3	177.61	179.61	2.00	179.05	1.44	179.45	1.84	179.61	2.00
**MAD**			**1.43**		**1.41**		**1.66**		**1.43**
N1-Re1-N2-C13	80.06	97.96	17.90	99.12	19.06	99.25	19.19	99.84	19.78
N1-Re1-N2-C15	-83.94	-85.05	1.11	-85.59	1.65	-84.96	1.02	-85.96	2.02
**MAD**			**9.51**		**10.36**		**10.11**		**10.90**

Table S11. Comparison of the experimentally obtained and calculated bond lengths and angles for complex **9** (MAD means the Mean Absolute Deviation).

**Complex 9**		**PBE0 LANL2DZ/6-31G(d,p)**		**PBE0 def2-TZVP**		**B3LYP LANL2DZ/6-31G(d,p)**		**B3LYP def2-TZVP**	
	Exp	Calc	|Exp-Calc|	Calc	|Exp-Calc|	Calc	|Exp-Calc|	Calc	|Exp-Calc|
Re1-C1	1.913	1.921	0.008	1.922	0.009	1.932	0.019	1.938	0.025
Re1-C2	1.903	1.899	0.004	1.902	0.001	1.912	0.009	1.918	0.015
Re1-C3	1.908	1.917	0.009	1.919	0.011	1.928	0.020	1.934	0.026
Re1-N1	2.194	2.186	0.008	2.195	0.001	2.217	0.023	2.225	0.031
Re1-O4	2.128	2.128	0.000	2.120	0.008	2.146	0.018	2.140	0.012
Re1-N2	2.197	2.21	0.013	2.221	0.024	2.246	0.049	2.254	0.057
C11-O4	1.330	1.302	0.028	1.298	0.032	1.309	0.021	1.304	0.026
**MAD**			**0.010**		**0.012**		**0.023**		**0.027**
N1-Re1-O4	76.47	76.25	0.22	75.71	0.76	75.79	0.68	75.25	1.22
N1-Re1-N2	85.12	84.80	0.32	85.33	0.21	85.38	0.26	85.73	0.61
O4-Re1-N2	82.73	81.86	0.87	82.03	0.70	82.00	0.73	82.11	0.62
N1-Re1-C1	173.98	171.84	2.14	173.16	0.82	171.40	2.58	172.24	1.74
O4-Re1-C2	171.73	171.24	0.49	172.22	0.49	170.68	1.05	171.73	0.00
N2-Re1-C3	176.34	175.83	0.51	177.85	1.51	179.57	3.23	177.36	1.02
C1-Re1-C2	88.43	90.91	2.48	88.77	0.34	91.39	2.96	89.62	1.19
C1-Re1-C3	86.41	90.52	4.11	88.10	1.69	90.81	4.40	89.42	3.01
C2-Re1-C3	89.95	90.93	0.98	88.57	1.38	91.34	1.39	89.40	0.55
N2-Re1-C1	91.06	91.77	0.71	92.48	1.42	91.26	0.20	91.82	0.76
N2-Re1-C2	92.62	92.52	0.10	93.26	0.64	92.09	0.53	92.94	0.32
O1-C1-Re1	178.64	178.98	0.34	179.74	1.10	178.63	0.01	179.36	0.72
O2-C2-Re2	176.85	175.58	1.27	177.29	0.44	175.24	1.61	176.87	0.02
O3-C3-Re3	175.66	179.73	4.07	178.96	3.30	179.57	3.91	179.52	3.86
**MAD**			**1.33**		**1.06**		**1.68**		**1.12**
N1-Re1-N2-C13	98.36	87.50	10.86	89.36	9.00	88.72	9.64	89.07	9.29
N1-Re1-N2-C15	-83.63	-93.94	10.31	-93.34	9.71	-93.50	9.87	-94.18	10.55
**MAD**			**10.59**		**9.36**		**9.76**		**9.92**

Table S12. Comparison of the experimentally obtained and calculated bond lengths and angles for complex **10** (MAD means the Mean Absolute Deviation).

Complex 10	1st molecule	PBE0 LANL2DZ/6-31G(d,p)		PBE0 def2-TZVP		B3LYP LANL2DZ/6-31G(d,p)		B3LYP def2-TZVP	
	Exp	Calc	|Exp-Calc|	Calc	|Exp-Calc|	Calc	|Exp-Calc|	Calc	|Exp-Calc|
Re1-C1	1.926	1.919	0.007	1.922	0.004	1.931	0.005	1.937	0.011
Re1-C2	1.915	1.900	0.015	1.903	0.012	1.913	0.002	1.919	0.004
Re1-C3	1.948	1.915	0.033	1.917	0.031	1.926	0.022	1.931	0.017
Re1-N1	2.165	2.186	0.021	2.198	0.033	2.218	0.053	2.228	0.063
Re1-O4	2.134	2.130	0.004	2.122	0.012	2.148	0.014	2.141	0.007
Re1-N2	2.189	2.224	0.035	2.236	0.047	2.264	0.075	2.273	0.084
C11-O4	1.329	1.300	0.029	1.296	0.033	1.307	0.022	1.303	0.026
**MAD**			**0.021**		**0.025**		**0.028**		**0.030**
N1-Re1-O4	76.39	76.25	0.14	75.66	0.73	75.81	0.58	75.20	1.19
N1-Re1-N2	82.40	85.28	2.88	85.98	3.58	85.68	3.28	86.35	3.95
O4-Re1-N2	81.55	80.50	1.05	80.82	0.73	80.72	0.83	80.99	0.56
N1-Re1-C1	170.53	171.77	1.24	173.17	2.64	171.32	0.79	172.26	1.73
O4-Re1-C2	174.65	173.00	1.65	173.66	0.99	172.47	2.18	173.16	1.49
N2-Re1-C3	176.43	173.82	2.61	175.79	0.64	173.90	2.53	175.36	1.07
C1-Re1-C2	89.74	90.35	0.61	88.31	1.43	90.82	1.08	89.15	0.59
C1-Re1-C3	88.82	90.52	1.70	88.67	0.15	90.79	1.97	89.38	0.56
C2-Re1-C3	86.46	89.63	3.17	87.31	0.85	90.10	3.64	88.19	1.73
N2-Re1-C1	91.72	90.02	1.70	91.66	0.06	90.51	1.21	91.04	0.68
N2-Re1-C2	97.08	96.37	0.71	96.89	0.19	95.84	1.24	96.44	0.64
O1-C1-Re1	177.78	178.93	1.15	179.65	1.87	178.59	0.81	179.37	1.59
O2-C2-Re2	176.10	177.12	1.02	177.87	1.77	176.87	0.77	177.70	1.60
O3-C3-Re3	176.17	179.63	3.46	179.09	2.92	179.36	3.19	179.66	3.49
**MAD**			**1.65**		**1.33**		**1.72**		**1.49**
N1-Re1-N2-C13	69.10	75.32	6.22	77.87	8.77	76.39	7.29	78.67	9.57
N1-Re1-N2-C15	-109.84	-107.33	2.51	-106.34	3.50	-106.79	3.05	-105.78	4.06
**MAD**			**4.37**		**6.14**		**5.17**		**6.82**

Table S13. Comparison of the experimentally obtained and calculated bond lengths and angles for complex **10** (MAD means the Mean Absolute Deviation).

Complex 10	2nd molecule	PBE0 LANL2DZ/6-31G(d,p)		PBE0 def2-TZVP		B3LYP LANL2DZ/6-31G(d,p)		B3LYP def2-TZVP	
	Exp	Calc	|Exp-Calc|	Calc	|Exp-Calc|	Calc	|Exp-Calc|	Calc	|Exp-Calc|
Re2-C21	1.923	1.919	0.004	1.922	0.001	1.931	0.008	1.937	0.014
Re2-C22	1.899	1.900	0.001	1.903	0.004	1.913	0.014	1.919	0.020
Re2-C23	1.936	1.915	0.021	1.917	0.019	1.926	0.010	1.931	0.005
Re2-N4	2.176	2.186	0.010	2.198	0.022	2.218	0.042	2.228	0.052
Re2-O8	2.131	2.130	0.001	2.122	0.009	2.148	0.017	2.141	0.010
Re2-N5	2.197	2.224	0.027	2.236	0.039	2.264	0.067	2.273	0.076
C31-O8	1.332	1.300	0.032	1.296	0.036	1.307	0.025	1.303	0.029
**MAD**			**0.014**		**0.019**		**0.026**		**0.029**
N4-Re2-O8	76.48	76.25	0.23	75.66	0.82	75.81	0.67	75.20	1.28
N4-Re2-N5	82.50	85.28	2.78	85.98	3.48	85.68	3.18	86.35	3.85
O8-Re2-N5	81.24	80.50	0.74	80.82	0.42	80.72	0.52	80.99	0.25
N4-Re2-C21	172.30	171.77	0.53	173.17	0.87	171.32	0.98	172.26	0.04
O8-Re2-C22	175.32	173.00	2.32	173.66	1.66	172.47	2.85	173.16	2.16
N5-Re2-C23	175.73	173.82	1.91	175.79	0.06	173.90	1.83	175.36	0.37
C21-Re2-C22	87.00	90.35	3.35	88.31	1.31	90.82	3.82	89.15	2.15
C21-Re2-C23	89.42	90.52	1.10	88.67	0.75	90.79	1.37	89.38	0.04
C22-Re2-C23	86.35	89.63	3.28	87.31	0.96	90.10	3.75	88.19	1.84
N5-Re2-C21	91.80	90.02	1.78	91.66	0.14	90.51	1.29	91.04	0.76
N5-Re2-C22	97.80	96.37	1.43	96.89	0.91	95.84	1.96	96.44	1.36
O5-C21-Re2	179.22	178.93	0.29	179.65	0.43	178.59	0.63	179.37	0.15
O6-C22-Re2	176.84	177.12	0.28	177.87	1.03	176.87	0.03	177.70	0.86
O7-C23-Re2	177.38	179.63	2.25	179.09	1.71	179.36	1.98	179.66	2.28
**MAD**			**1.59**		**1.04**		**1.78**		**1.24**
N4-Re2-N5-C33	70.90	75.32	4.42	77.87	6.97	76.39	5.49	78.67	7.77
N4-Re2-N5-C35	-109.27	-107.33	1.94	-106.34	2.93	-106.79	2.48	-105.78	3.49
**MAD**			**3.18**		**4.95**		**3.98**		**5.63**

Table S14. Comparison of the experimentally obtained and calculated bond lengths and angles for complex **11** (MAD means the Mean Absolute Deviation).

Complex 11		PBE0 LANL2DZ/6-31G(d,p)		PBE0 def2-TZVP		B3LYP LANL2DZ/6-31G(d,p)		B3LYP def2-TZVP	
	Exp	Calc	|Exp-Calc|	Calc	|Exp-Calc|	Calc	|Exp-Calc|	Calc	|Exp-Calc|
Re1-C1	1.917	1.920	0.003	1.922	0.005	1.931	0.014	1.937	0.020
Re1-C2	1.908	1.900	0.008	1.903	0.005	1.913	0.005	1.920	0.012
Re1-C3	1.923	1.916	0.007	1.917	0.006	1.926	0.003	1.932	0.009
Re1-N1	2.172	2.187	0.015	2.197	0.025	2.218	0.046	2.227	0.055
Re1-O4	2.140	2.153	0.013	2.144	0.004	2.172	0.032	2.164	0.024
Re1-N2	2.204	2.214	0.010	2.225	0.021	2.252	0.048	2.261	0.057
C11-O4	1.333	1.312	0.021	1.307	0.026	1.320	0.013	1.313	0.020
**MAD**			**0.011**		**0.013**		**0.023**		**0.028**
N1-Re1-O4	76.56	76.06	0.50	75.50	1.06	75.57	0.99	75.03	1.53
N1-Re1-N2	84.29	86.24	1.95	86.72	2.43	86.85	2.56	87.12	2.83
O4-Re1-N2	81.15	79.95	1.20	79.97	1.18	79.95	1.20	80.02	1.13
N1-Re1-C1	175.77	172.28	3.49	173.39	2.38	171.88	3.89	172.50	3.27
O4-Re1-C2	171.46	172.41	0.95	173.19	1.73	171.67	0.21	172.58	1.12
N2-Re1-C3	176.15	173.61	2.54	175.20	0.95	173.63	2.52	174.77	1.38
C1-Re1-C2	87.24	90.40	3.16	88.43	1.19	90.92	3.68	89.30	2.06
C1-Re1-C3	87.52	90.35	2.83	88.53	1.01	90.63	3.11	89.26	1.74
C2-Re1-C3	85.32	89.60	4.28	87.36	2.04	90.10	4.78	88.24	2.92
N2-Re1-C1	91.80	90.79	1.01	91.66	0.14	90.29	1.51	90.96	0.84
N2-Re1-C2	98.43	96.68	1.75	97.44	0.99	96.20	2.23	96.99	1.44
O1-C1-Re1	177.39	179.04	1.65	179.45	2.06	178.70	1.31	179.23	1.84
O2-C2-Re2	175.56	176.96	1.40	177.64	2.08	176.68	1.12	177.52	1.96
O3-C3-Re3	176.71	179.51	2.80	179.18	2.47	179.28	2.57	179.61	2.90
**MAD**			**2.11**		**1.55**		**2.26**		**1.93**
N1-Re1-N2-C13	97.44	90.66	6.78	92.86	4.58	92.75	4.69	93.84	3.60
N1-Re1-N2-C15	-93.83	-95.48	1.65	-94.22	0.39	-94.36	0.53	-94.01	0.18
**MAD**			**2.60**		**1.68**		**2.46**		**1.94**

Table S15. Comparison of the experimentally obtained and calculated bond lengths and angles for complex **12** (MAD means the Mean Absolute Deviation).

**Complex 12**		**PBE0 LANL2DZ/6-31G(d,p)**		**PBE0 def2-TZVP**		**B3LYP LANL2DZ/6-31G(d,p)**		**B3LYP def2-TZVP**	
	Exp	Calc	|Exp-Calc|	Calc	|Exp-Calc|	Calc	|Exp-Calc|	Calc	|Exp-Calc|
Re1-C1	1.924	1.921	0.003	1.923	0.001	1.932	0.008	1.938	0.014
Re1-C2	1.904	1.901	0.003	1.904	0.000	1.914	0.010	1.920	0.016
Re1-C3	1.918	1.918	0.000	1.920	0.002	1.928	0.010	1.934	0.016
Re1-N1	2.173	2.187	0.014	2.196	0.023	2.218	0.045	2.226	0.053
Re1-O4	2.145	2.151	0.006	2.142	0.003	2.170	0.025	2.161	0.016
Re1-N2	2.189	2.195	0.006	2.207	0.018	2.229	0.040	2.237	0.048
C11-O4	1.320	1.314	0.006	1.308	0.012	1.321	0.001	1.314	0.006
**MAD**			**0.005**		**0.008**		**0.020**		**0.024**
N1-Re1-O4	76.60	76.05	0.55	75.53	1.07	75.57	1.03	75.08	1.52
N1-Re1-N2	85.05	86.39	1.34	86.73	1.68	86.77	1.72	86.95	1.90
O4-Re1-N2	81.41	80.00	1.41	80.00	1.41	79.94	1.47	80.06	1.35
N1-Re1-C1	173.23	172.25	0.98	173.29	0.06	171.80	1.43	172.38	0.85
O4-Re1-C2	169.03	170.70	1.67	171.56	2.53	170.11	1.08	171.06	2.03
N2-Re1-C3	178.23	175.29	2.94	177.00	1.23	175.17	3.06	176.48	1.75
C1-Re1-C2	90.28	90.60	0.32	88.61	1.67	91.08	0.80	89.47	0.81
C1-Re1-C3	83.59	90.27	6.68	88.49	4.90	90.56	6.97	89.21	5.62
C2-Re1-C3	89.59	90.79	1.20	88.66	0.93	91.18	1.59	89.46	0.13
N2-Re1-C1	98.17	91.33	6.84	92.13	6.04	90.92	7.25	91.56	6.61
N2-Re1-C2	90.24	93.62	3.38	94.29	4.05	93.39	3.15	93.98	3.74
O1-C1-Re1	173.28	178.95	5.67	179.35	6.07	178.63	5.35	179.12	5.84
O2-C2-Re2	175.07	176.36	1.29	177.75	2.68	176.03	0.96	177.34	2.27
O3-C3-Re3	176.49	179.75	3.26	179.14	2.65	179.49	3.00	179.67	3.18
**MAD**			**2.68**		**2.64**		**2.78**		**2.69**
N1-Re1-N2-C13	17.01	91.06	74.05	92.73	75.72	91.82	74.81	92.81	75.80
N1-Re1-N2-C15	-168.05	-92.87	75.18	-92.04	76.01	-92.80	75.25	-92.56	75.49
**MAD**			**74.62**		**75.87**		**75.03**		**75.65**

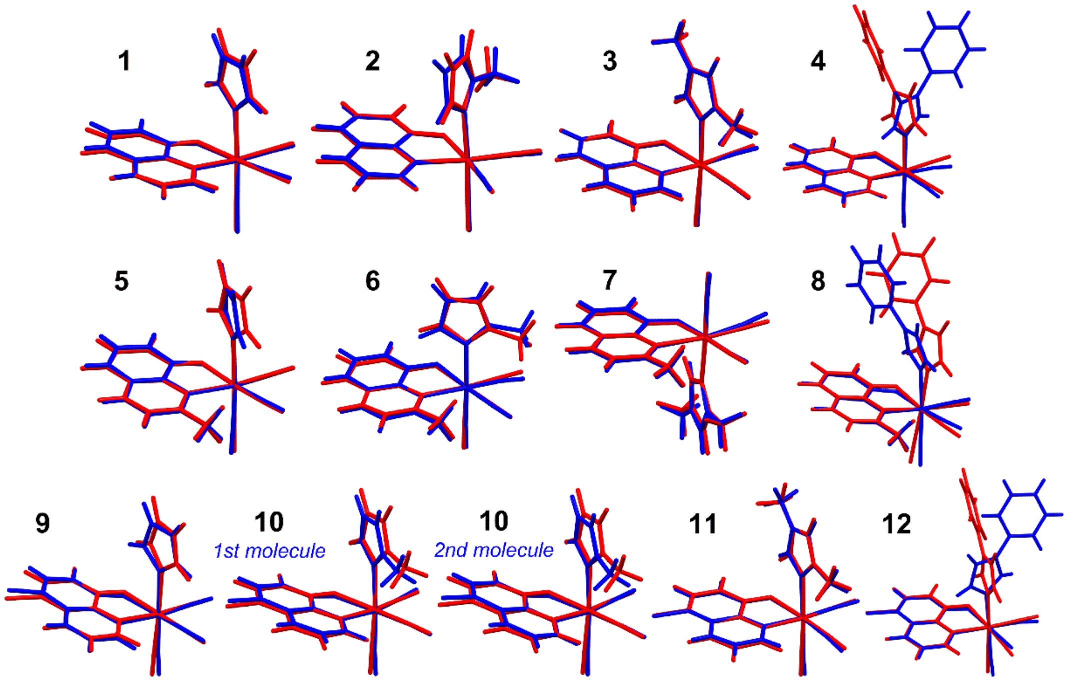

Fig. S2. Illustration of the overlay of the scXRD molecular structures (blue) and DFT (pbe0/ def2-TZVP/ECP(Re)) optimized ones (red) for the Re(I) complexes: **1** (RMSD = 0.115 Å), **2** (RMSD = 0.146 Å), **3** (RMSD = 0.105 Å), **4** (RMSD = 1.172 Å), **5** (RMSD = 0.152 Å), **6** (RMSD = 0.231 Å), **7** (RMSD = 0.128 Å), **8** (RMSD = 0.451 Å), **9** (RMSD = 0.187 Å), **10** 1st molecule (RMSD = 0.187 Å), **10** 2nd molecule (RMSD = 0.209 Å), **11** (RMSD = 0.109 Å), **12** (RMSD = 1.152 Å). For **10**, two scXRD molecular structures are considered (Figure S3).

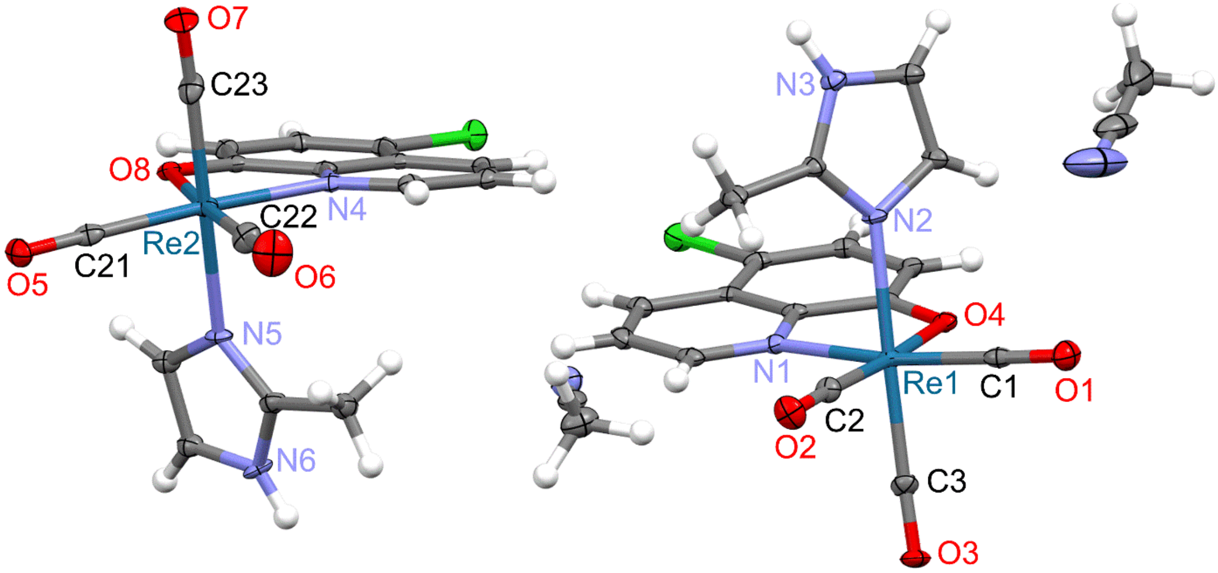

Fig. S3. Molecular structure of [Re(CO)$_3$(ClQ)MeHim]·CH$_3$CN (**10·CH$_3$CN**) complex showing two crystallographically independent metal complex molecules and co-crystallized solvent molecules. Thermal ellipsoids are plotted at a 50% probability level.

Table S16. Geometric parameters associated with intermolecular N−H···O hydrogen bond for studied rhenium(I) complexes (atoms labeling for complex **10** according to Figure S3).

Complex	Structural motif	D−H···A	d(D−H) [Å]	d(H···A) [Å]	d(D···A) [Å]	<(D−H−A) [°]	Symmetry code (*i*)
**1**	chain	N3−H3···O4^{i}	0.85(3)	1.90(3)	2.741(2)	173(3)	*x+1, y, z*
**2**	chain	N3−H3···O4^{i}	0.83(4)	1.97(4)	2.772(4)	160(4)	*-x+1, y+0.5, -z+0.5*
**3**	dimer	N3−H3···O4^{i}	0.84(3)	2.27(3)	2.927(3)	135(3)	*-x, -y, -z+1*
**4**	dimer	N3−H3···O4^{i}	0.95(5)	1.78(5)	2.716(4)	167(5)	*-x+1.5, -y+0.5, -z*
**5**	chain	N3−H3···O4^{i}	0.85(3)	1.95(3)	2.787(3)	168(3)	*x+1, y, z*
**6**	dimer	N3−H3···O4^{i}	0.78(2)	2.12(2)	2.863(2)	159(3)	*-x+1, -y+1, -z*
**7**	dimer	N3−H3···O4^{i}	0.86(3)	2.26(3)	2.914(3)	132(3)	*-x+1, -y+1, -z*
**8**	dimer	N3−H3···O1^{i}	0.79(4)	2.40(3)	3.008(3)	135(3)	*-x, -y+1, -z+2*
**9**	chain	N3−H3···O4^{i}	0.81(4)	1.97(4)	2.751(4)	162(4)	*x+2, y, z*
**10**	chain	N3−H3···O8^{i}	0.94(6)	1.84(6)	2.755(6)	166(5)	*-x+1, -y+1, -z+1*
		N6−H6···O4^{i}	0.84(4)	1.92(3)	2.750(5)	167(4)	*-x, -y+1, -z+1*
**11**	dimer	N3−H3···O4^{i}	0.89(3)	2.24(4)	2.955(4)	137(4)	*-x+2, -y, -z+1*
**12**	dimer	N3−H3···O4^{i}	0.85(9)	1.88(9)	2.719(6)	167(7)	*-x+1, -y+1, -z+1*

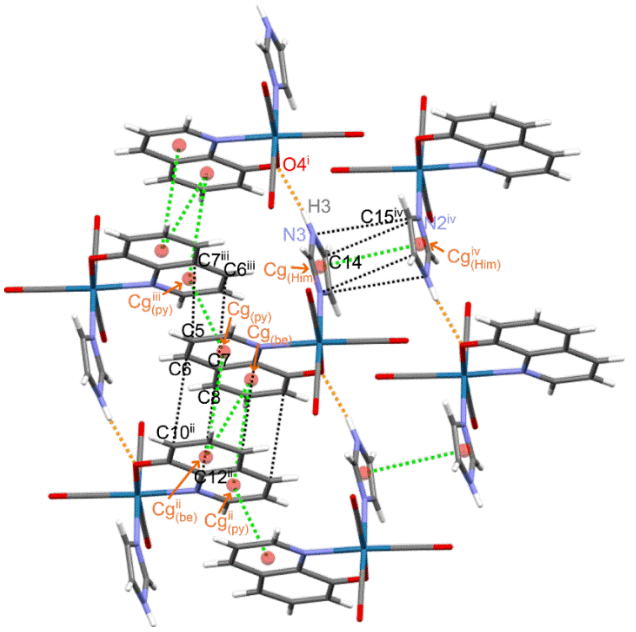

Figure S4. Fragment of the crystal structure of **1** showing some intermolecular interactions: hydrogen bonds (orange dotted lines) – 2.741(2) Å (N3−H3···O4^{i}), the shortest interatomic contacts between rings (black dotted lines) – 3.336(3) Å (C6···C10^{ii}), 3.380(3) Å (C7···C6^{iii}), 3.345(3) Å (C5···C7^{iii}), 3.352(3) Å (C8···C12^{ii}), 3.481(3) Å (N3···C15^{iv}), 3.587(3) Å (C14···N2^{iv}); and the shortest distances between centroids (green dotted lines) – 3.607 Å (Cg$_{(Him)}$···Cg$^{iv}_{(Him)}$), 3.619 Å (Cg$_{(be)}$···Cg$^{ii}_{(be)}$), 3.737 Å (Cg$_{(py)}$···Cg$^{iii}_{(py)}$), 3.768 Å (Cg$_{(py)}$···Cg$^{ii}_{(be)}$). (Symmetry codes: *(i) x+1, y, z*; *(ii) = −x, −y+1, −z*; *(iii) = −x+1, −y+1, −z*; *(iv) = −x+1, −y+1, −z+1)*.

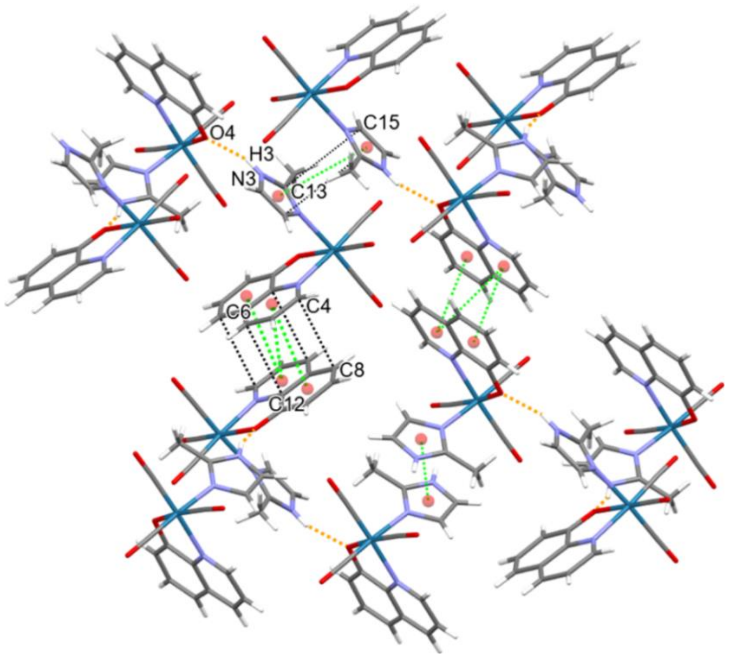

Figure S5. Fragment of the crystal structure of **2** showing some intermolecular interactions: hydrogen bonds (orange dotted lines) – 2.772(4) Å (N3−H3···O4^{i}), the shortest interatomic contacts between rings (black dotted lines) – 3.420(3) Å (C4···C8^{ii}), 3.500(4) Å (C6···C12^{ii}), 3.586(4) Å (C5···C9^{ii}), 4.019(4) Å (C13···C15^{iii}), and the shortest distances between centroids of rings (green dotted lines) – 3.725 Å (Cg$_{(py)}$···Cg$^{ii}_{(be)}$), 3.775 Å (Cg$_{(py)}$···Cg$^{ii}_{(py)}$), 4.179 Å (Cg$_{(MeHim)}$···Cg$^{iii}_{(MeHim)}$) (Symmetry codes: *(i) −x+1, y+0.5, −z+0.5*; *(ii) = −x+2, −y+1, −z+1*; *(iii) = −x+1, −y+1, −z)*.

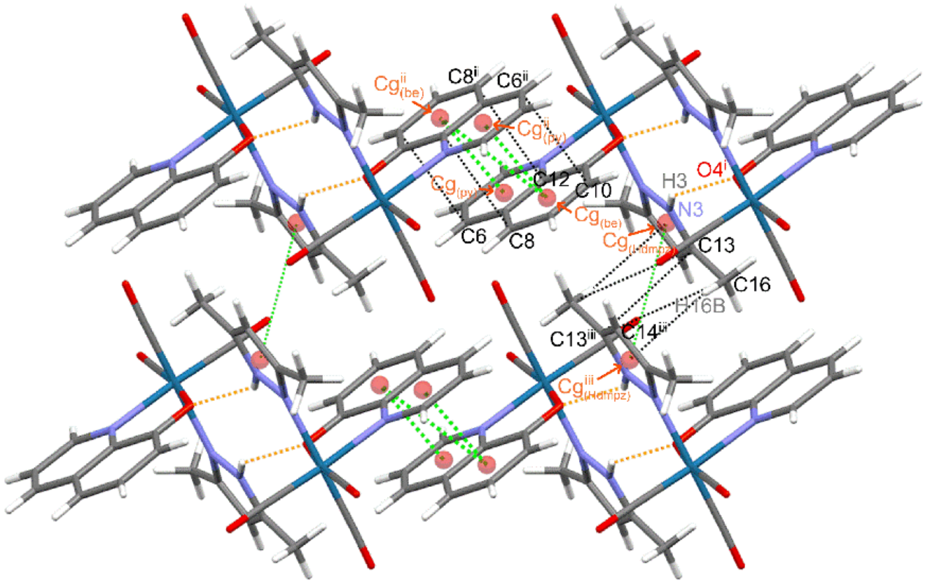

Figure S6. Fragment of the crystal structure of **3** showing some intermolecular interactions: hydrogen bonds (orange dotted lines) – 2.927(3) Å (N3−H3···O4^{i}), the shortest interatomic contacts between rings (black dotted lines) c 3.468(5) Å (C13···C13^{iii}), 3.538(4) Å (C16···C14^{iii}), 2.690(4) Å (H16B···C14^{iii}), 2.725(4) Å (H16B···Cg$^{iii}_{(Hdmpz)}$); and the shortest distances between centroids (green dotted lines) – 3.690 Å (Cg$_{(be)}$···Cg$^{ii}_{(be)}$), 3.963 Å (Cg$_{(py)}$···Cg$^{ii}_{(be)}$), 4.447 Å (Cg$_{(Hdmpz)}$···Cg$^{iii}_{(Hdmpz)}$) (Symmetry codes: *(i)* = *−x, −y, −z+1*; *(ii)* = *−x*, *−y*, *−z*; *(iii)* = *−x+1*, *−y*, *−z+1*).

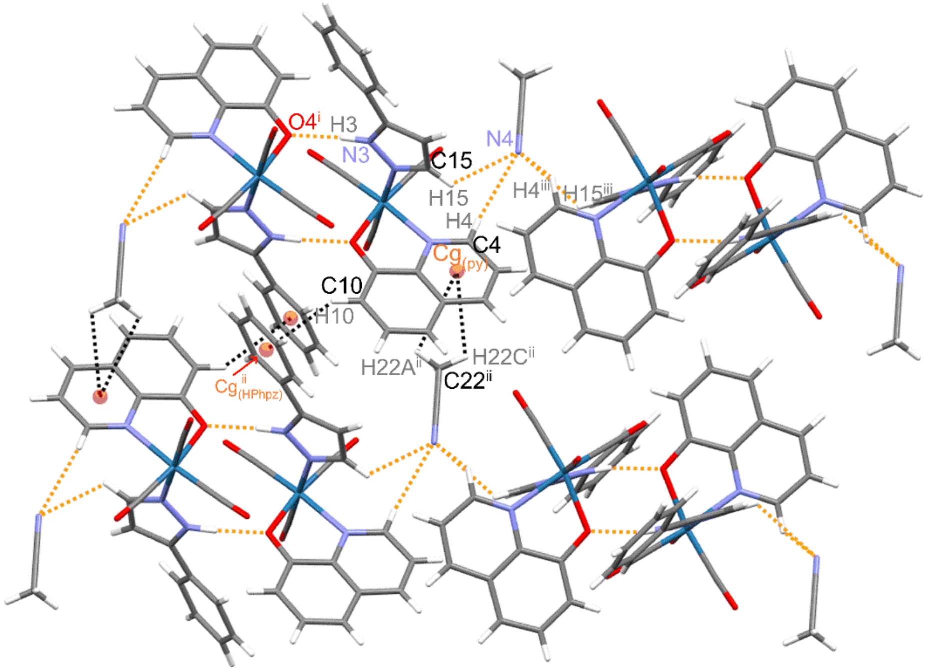

Figure S7. Fragment of the crystal structure of **4** showing some intermolecular interactions: hydrogen bonds (orange dotted lines) – 2.716(4) Å (N3−H3···O4^{i}), 3.298 Å (C15−H15···N4), 3.546 Å (C4−H4···N4), and the shortest interatomic contacts (black dotted lines) – 2.811 Å (H10···Cg$^{ii}_{(HPhpz)}$), 3.421 Å (Cg$_{(py)}$···H22A^{ii}), 3.454 Å (Cg$_{(py)}$···H22C^{ii}), 3.909 Å (Cg$_{(py)}$···C22^{ii}) (Symmetry codes: *(i)* = *−x+1.5, −y+0.5, −z*; *(ii)* = *x+0.5*, *y−0.5*, *z*; *(iii)* = *−x+1*, *y*, *−z+0.5*).

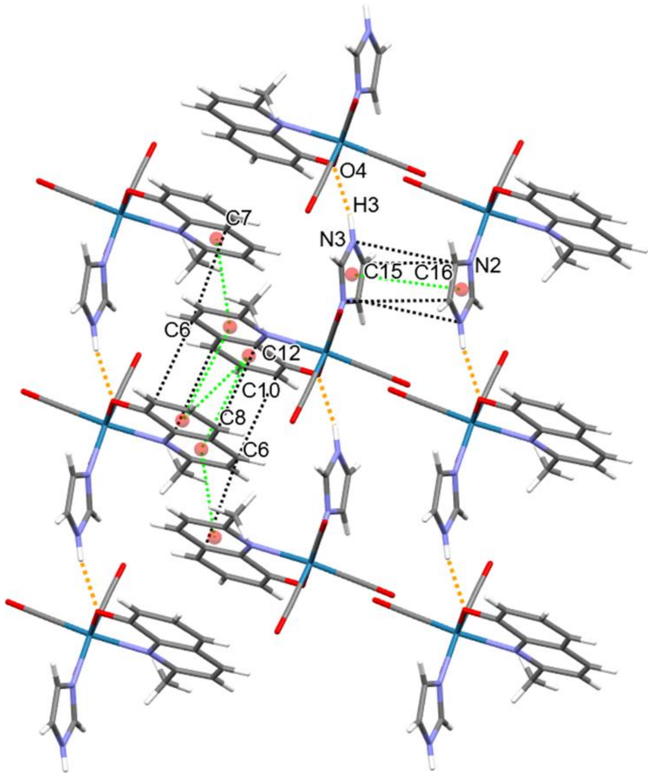

Figure S8. Fragment of the crystal structure of **5** showing some intermolecular interactions: hydrogen bonds (orange dotted lines) – 2.787(3) Å (N3−H3···O4^{i}), the shortest interatomic contacts between rings (black dotted lines) – 3.297(5) Å (C10···C6^{ii}), 3.345(4) Å (C12···C8^{ii}), 3.365(4) Å (C6···C7^{iii}), 3.819(5) Å (N2···C15^{iv}), 3.822(4) Å (N3···C16^{iv}), and the shortest distances between centroids of rings (green dotted lines) – 3.636 Å (Cg$_{(be)}$···Cg$^{ii}_{(be)}$), 3.688 Å (Cg$_{(py)}$···Cg$^{ii}_{(be)}$), 3.769 Å (Cg$_{(py)}$···Cg$^{iii}_{(py)}$), 3.891 Å (Cg$_{(Him)}$···Cg$^{iv}_{(Him)}$). (Symmetry codes: *(i) = x+1, y, z*; *(ii) = −x, −y+1, −z+2*; *(iii) = −x+1, −y+1, −z+2*; *(iv) = −x+1, −y+1, −z+1)*.

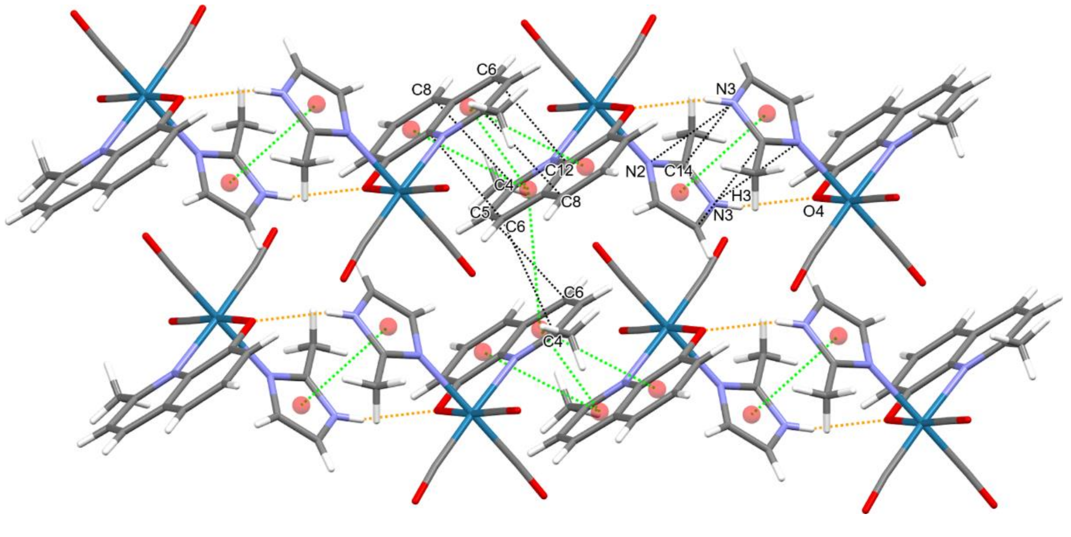

Figure S9. Fragment of the crystal structure of **6** showing some intermolecular interactions: hydrogen bonds (orange dotted lines) – 2.863(2) Å (N3−H3···O4^{i}), the shortest interatomic contacts between rings (black dotted lines) – 3.371(3) Å (C12···C6^{ii}), 3.461(3) Å (C4···C8^{ii}), 3.429(3) Å (C5···C6^{iii}), 3.471(3) Å (C6···C4^{iii}), 3.383(3) Å (N2···N3^{i}), 3.397(3) Å (C14···C15^{i}), and the shortest distances between centroids of rings (green dotted lines) – 3.722 Å (Cg$_{(py)}$···Cg$^{ii}_{(be)}$), 3.810 Å (Cg$_{(py)}$···Cg$^{ii}_{(py)}$), 3.931 Å (Cg$_{(py)}$···Cg$^{iii}_{(py)}$), 3.467 Å (Cg$_{(MeHim)}$···Cg$^{i}_{(meHim)}$). (Symmetry codes: *(i) = −x+1, −y+1, −z*; *(ii) = −x+2, −y+1, −z+1*; *(iii) = −x+1, −y+1, −z+1*).

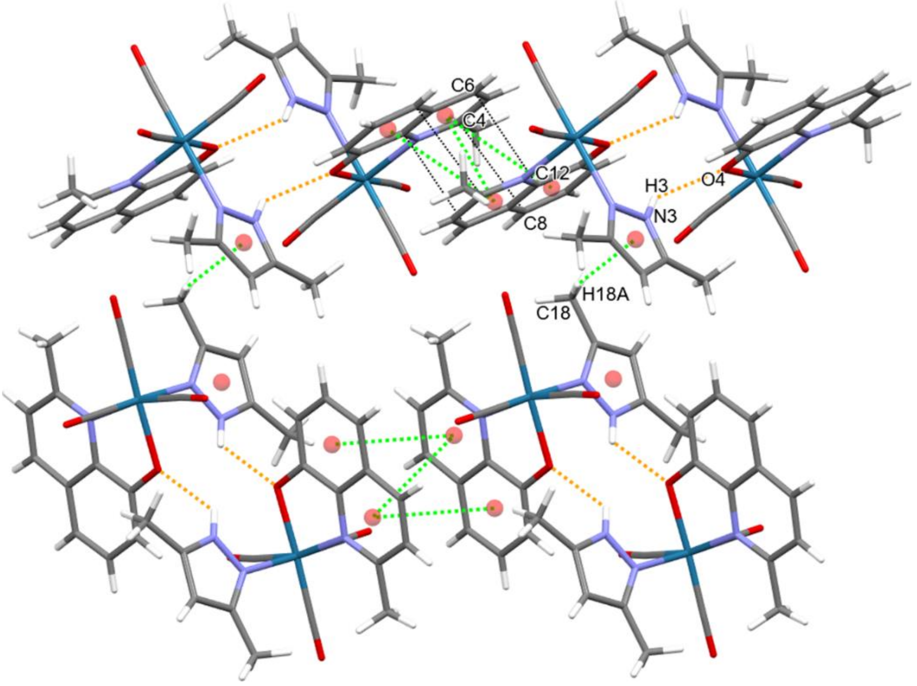

Figure S10. Fragment of the crystal structure of **7** showing some intermolecular interactions: hydrogen bonds (orange dotted lines) – 2.914(3) Å (N3−H3···O4^{i}), the shortest interatomic contacts between rings (black dotted lines) – 3.301(3) Å (C8···C4^{ii}), 3.347(3) Å (C12···C6^{ii}), 3.411(4) Å (C8···C5^{ii}), 3.697 Å (Cg$_{(Hdmpz)}$···C18^{iii}), and the shortest distances between centroids of rings (green dotted lines) – 3.659 Å (Cg$_{(py)}$···Cg$^{ii}_{(py)}$), 3.733 Å (Cg$_{(py)}$···Cg$^{ii}_{(be)}$), 5.027 Å (Cg$_{(Hdmpz)}$···Cg$^{iii}_{(Hdmpz)}$). (Symmetry codes: *(i)* = *−x+1, −y+1, −z*; *(ii)* = *−x+2, −y+1, −z*; *(iii)* = *x*, *−y+1.5*, *z−0.5*).

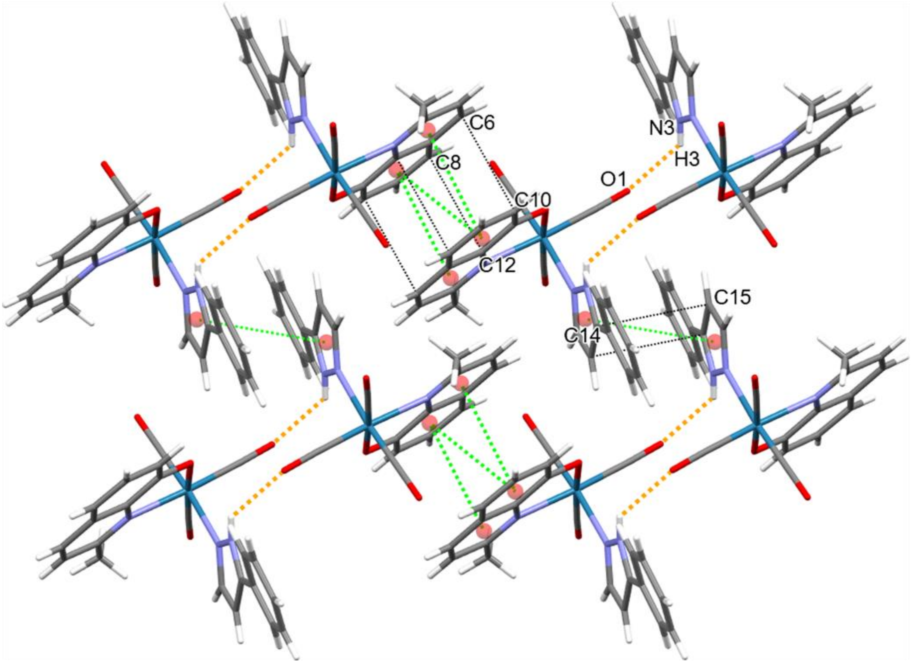

Figure S11. Fragment of the crystal structure of **8** showing some intermolecular interactions: hydrogen bonds (orange dotted lines) – 3.008(3) Å (N3−H3···O1^{i}), the shortest interatomic contacts between rings (black dotted lines) – 3.459(5) Å (C10···C6^{ii}), 3.478(5) Å (C12···C8^{ii}), 3.671(4) Å (C14···C15^{iii}), and the shortest distances between centroids of rings (green dotted lines) – 3.613 Å (Cg$_{(be)}$···Cg$^{ii}_{(be)}$), 3.769 Å (Cg$_{(py)}$···Cg$^{ii}_{(be)}$), 4.058 Å (Cg$_{(HPhpz)}$···Cg$^{iii}_{(HPhpz)}$). (Symmetry codes: *(i)* = *−x, −y+1, −z+2*; *(ii)* = *−x, −y+1, −z+1*; *(iii)* = *−x+1*, *−y+1*, *−z+2*).

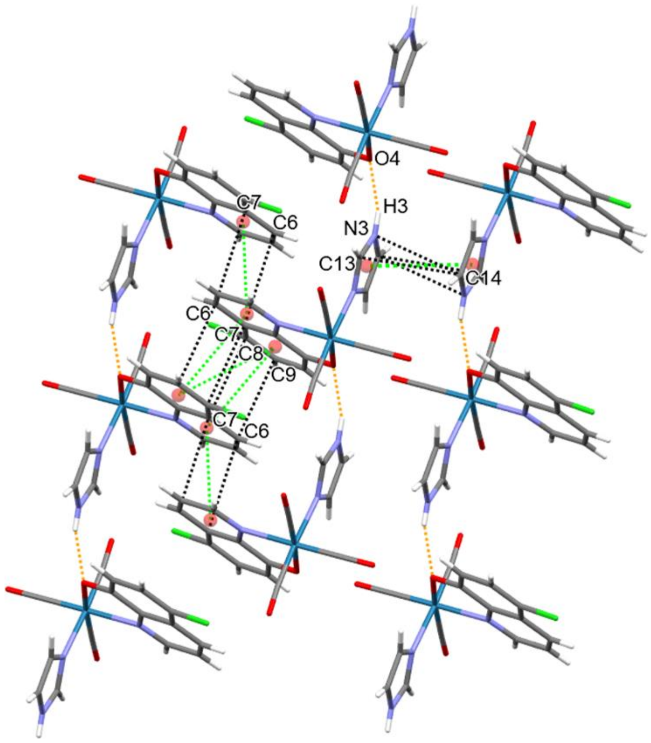

Figure S12. Fragment of the crystal structure of **9** showing some intermolecular interactions: hydrogen bonds (orange dotted lines) – 2.751(4) Å (N3−H3···O4^{i}), the shortest interatomic contacts between rings (black dotted lines) – 3.407(4) Å (C9···C6^{ii}), 3.464(4) Å (C8···C7^{ii}), 3.500(4) Å (C6···C7^{iii}), 3.738(4) Å (C13···C14^{iv}), 3.794(4) Å (N3···C14^{iv}), and the shortest distances between centroids of rings (green dotted lines) – 3.897 Å (Cg$_{(py)}$···Cg$^{ii}_{(be)}$), 3.915 Å (Cg$_{(py)}$···Cg$^{iii}_{(py)}$), 4.022 Å (Cg$_{(be)}$···Cg$^{ii}_{(be)}$), 3.878 Å (Cg$_{(Him)}$···Cg$^{iv}_{(Him)}$). (Symmetry codes: *(i)* = *x+1, y, z*; *(ii)* = *−x, −y, −z*; *(iii)* = *−x+1, −y, −z*; *(iv)* = *−x+1, −y, −z+1*).

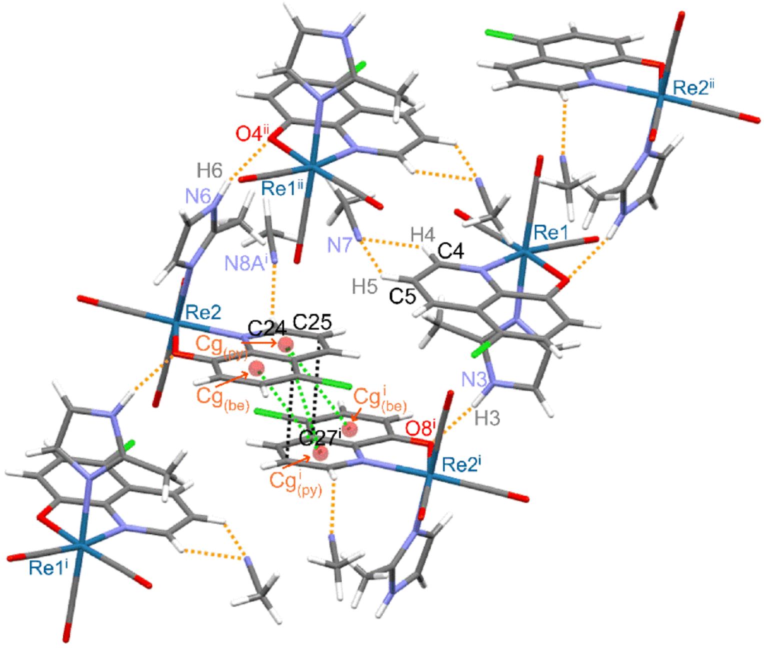

Figure S13. Fragment of the crystal structure of **10** showing some intermolecular interactions: hydrogen bonds (orange dotted lines) – 2.750(5) Å (N6−H6···O4^{i}), 2.755(6) Å (N3−H3···O8^{ii}), 3.217(7) Å (C4−H4···N7), 3.641(7) Å (C5−H5···N7), 3.91(3) Å (C24−H24···N8A^{i}), the shortest interatomic contacts between rings (black dotted lines) – 3.331(5) Å (C25···C27^{i}), 3.576(5) Å (C24···C27^{i}) and the shortest distances between centroids of rings (green dotted lines) – 3.593 Å (Cg$_{(py)}$···Cg$^{i}_{(py)}$), 4.298 Å (Cg$_{(py)}$···Cg$^{i}_{(be)}$) (Symmetry codes: *(i)* = *−x+1, −y+1, −z+1*; *(ii)* = *−x, −y+1, −z+1*).

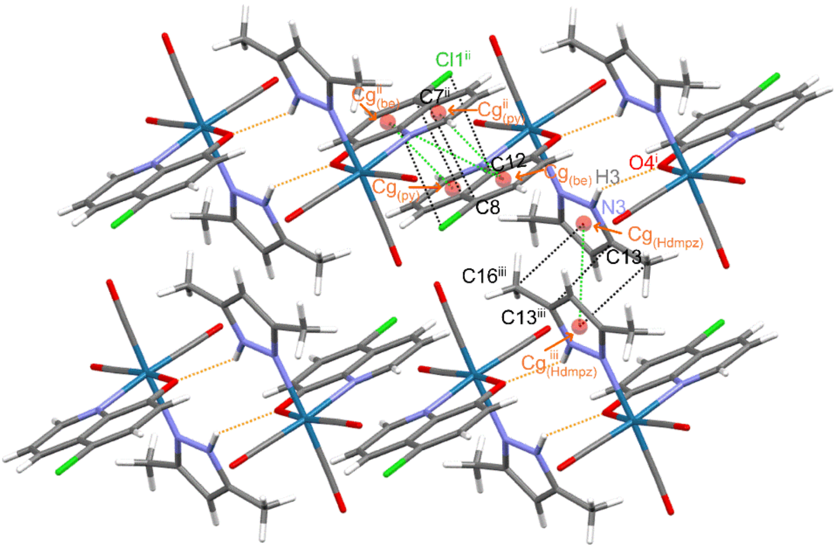

Figure S14. Fragment of the crystal structure of **9** showing some intermolecular interactions: hydrogen bonds (orange dotted lines) – 2.751(4) Å (N3−H3···O4^{i}), the shortest interatomic contacts between rings (black dotted lines) – 3.423(3) Å (C12···Cl1^{ii}), 3.445(4) Å (C8···C7^{ii}), 3.477 Å (Cg$_{(Hdmpz)}$···C16^{iii}), 3.496(5) Å (C13···C13^{iii}), and the shortest distances between centroids of rings (green dotted lines) – 4.251 Å (Cg$_{(be)}$···Cg$^{ii}_{(be)}$), 4.371 Å (Cg$_{(py)}$···Cg$^{ii}_{(be)}$), 4.551 Å (Cg$_{(Hdmpz)}$···Cg$^{iii}_{(Hdmpz)}$), 3.878 Å (Cg$_{(Him)}$···Cg$^{iv}_{(Him)}$). (Symmetry codes: *(i) = −x+2, −y, −z+1*; *(ii) = −x+2, −y, −z+2*; *(iii) = −x+1, −y, −z+1*).

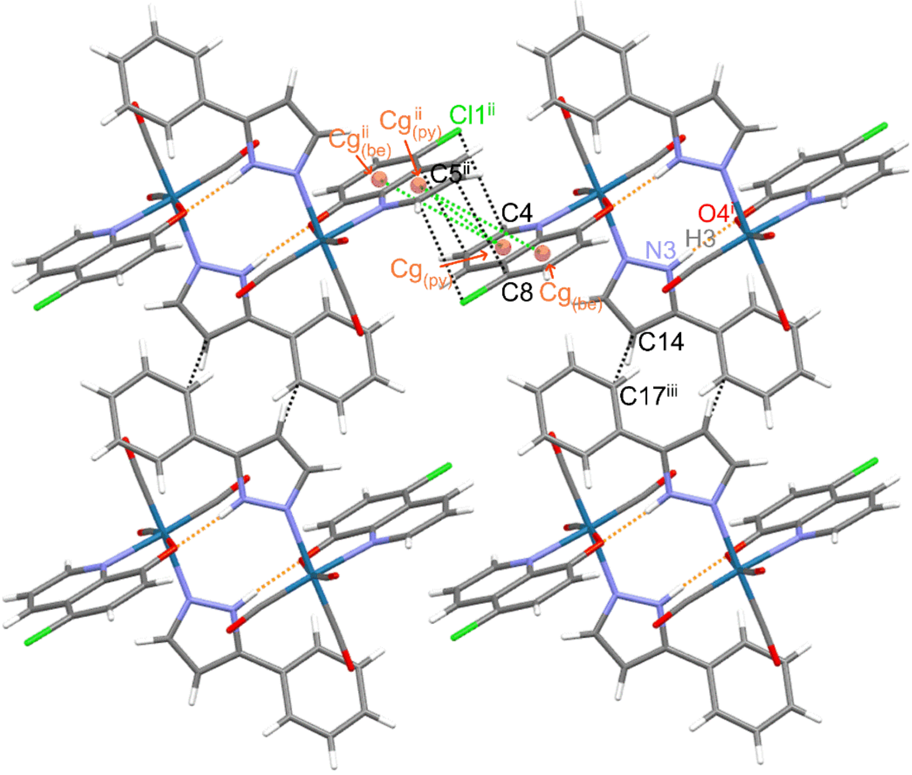

Figure S15. Fragment of the crystal structure of **12** showing some intermolecular interactions: hydrogen bonds (orange dotted lines) – 2.719(6) Å (N3−H3···O4^{i}), the shortest interatomic contacts between rings (black dotted lines) – 3.400(9) Å (C8···C5^{ii}), 3.411(9) Å (C4···Cl1^{ii}), 3.443(5) Å (C14···C17^{iii}), and the shortest distances between centroids of rings (green dotted lines) – 3.999 Å (Cg$_{(py)}$···Cg$^{ii}_{(py)}$), 4.316 Å (Cg$_{(py)}$···Cg$^{ii}_{(be)}$) (Symmetry codes: *(i) = −x+1, −y+1, −z+1*; *(ii) = −x, −y+1, −z+2*; *(iii) = −x+1, −y+2, −z+1*).

Table S17. The shortest contacts between centroids of the adjacent five- and six-membered rings in the crystal structure of studied complexes ($Cg_{(py)}$ and $Cg_{(be)}$ – centroids of the pyridine and benzene ring, respectively, in the bidentate ligand; $Cg_{(Him)}$, $Cg_{(MeHim)}$ and $Cg_{(Hdmpz)}$ – centroids of the respective monodentate ligand; $Cg_{(pz\text{-}HPhpz)}$ and $Cg_{(Ph\text{-}HPhpz)}$ – centroids of the pyrazole and phenyl part, respectively, in the HPhpz ligand).

Complex	interaction	distance [Å]
**1**	$Cg_{(be)}\cdots Cg_{(be)}$	3.619
	$Cg_{(py)}\cdots Cg_{(py)}$	3.737
	$Cg_{(py)}\cdots Cg_{(be)}$	3.768
	$Cg_{(Him)}\cdots Cg_{(Him)}$	3.607
**2**	$Cg_{(py)}\cdots Cg_{(be)}$	3.725
	$Cg_{(py)}\cdots Cg_{(py)}$	3.815
	$Cg_{(MeHim)}\cdots Cg_{(MeHim)}$	4.179
**3**	$Cg_{(be)}\cdots Cg_{(be)}$	3.690
	$Cg_{(py)}\cdots Cg_{(be)}$	3.963
	$Cg_{(Hdmpz)}\cdots Cg_{(Hdmpz)}$	4.447
**4**	$Cg_{(pz\text{-}HPhpz)}\cdots Cg_{(Ph\text{-}HPhpz)}$	5.135
**5**	$Cg_{(be)}\cdots Cg_{(be)}$	3.636
	$Cg_{(py)}\cdots Cg_{(be)}$	3.688
	$Cg_{(py)}\cdots Cg_{(py)}$	3.660
	$Cg_{(Him)}\cdots Cg_{(Him)}$	3.891
**6**	$Cg_{(py)}\cdots Cg_{(be)}$	3.722
	$Cg_{(py)}\cdots Cg_{(py)}$	3.810
	$Cg_{(py)}\cdots Cg_{(py)}$	3.931
	$Cg_{(MeHim)}\cdots Cg_{(MeHim)}$	3.467
**7**	$Cg_{(py)}\cdots Cg_{(py)}$	3.659
	$Cg_{(py)}\cdots Cg_{(be)}$	3.733
	$Cg_{(Hdmpz)}\cdots Cg_{(Hdmpz)}$	5.027
**8**	$Cg_{(be)}\cdots Cg_{(be)}$	3.613
	$Cg_{(py)}\cdots Cg_{(be)}$	3.769
	$Cg_{(pz\text{-}HPhpz)}\cdots Cg_{(pz\text{-}HPhpz)}$	4.058
	$Cg_{(pz\text{-}HPhpz)}\cdots Cg_{(Ph\text{-}HPhpz)}$	4.752
**9**	$Cg_{(py)}\cdots Cg_{(be)}$	3.897
	$Cg_{(py)}\cdots Cg_{(py)}$	3.915
	$Cg_{(be)}\cdots Cg_{(be)}$	4.022
	$Cg_{(Him)}\cdots Cg_{(Him)}$	3.878
**10**	$Cg_{(py)}\cdots Cg_{(py)}$	3.593
	$Cg_{(py)}\cdots Cg_{(be)}$	4.298
**11**	$Cg_{(be)}\cdots Cg_{(be)}$	4.251
	$Cg_{(py)}\cdots Cg_{(be)}$	4.371
	$Cg_{(Hdmpz)}\cdots Cg_{(Hdmpz)}$	4.551
**12**	$Cg_{(py)}\cdots Cg_{(py)}$	3.999
	$Cg_{(py)}\cdots Cg_{(be)}$	4.316
	$Cg_{(Ph\text{-}HPhpz)}\cdots Cg_{(pz\text{-}HPhpz)}$	5.230

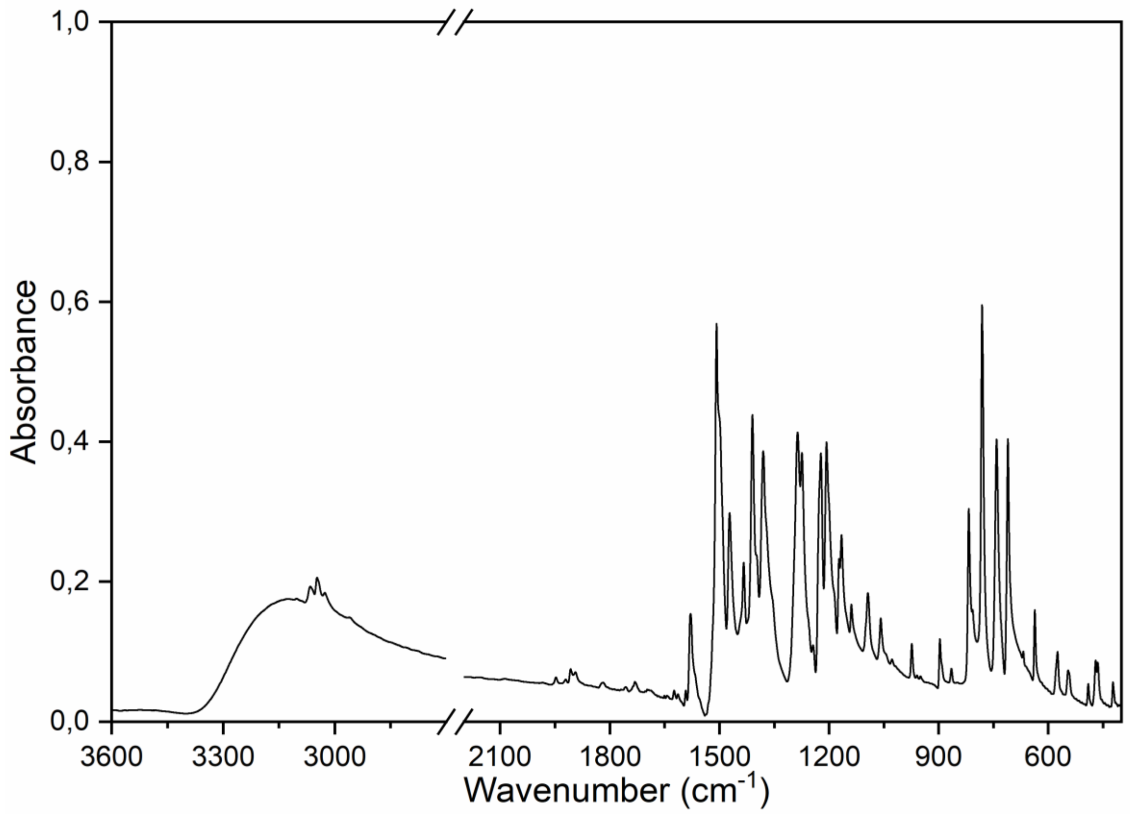

Fig. S16. FTIR spectrum of 8-hydroxyquinoline (HQ).

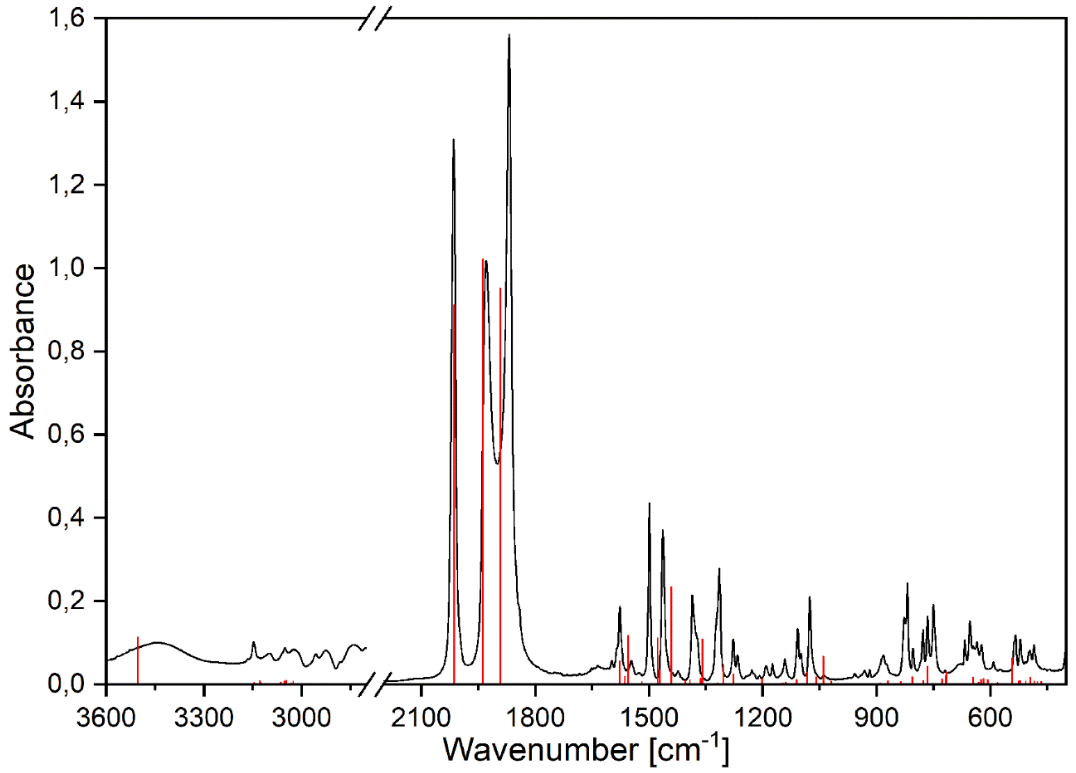

Fig. S17. Experimental and PBE0/def2-TZVP simulated (vertical lines) FTIR spectrum of [Re(CO)$_3$(Q)Him] (**1**).

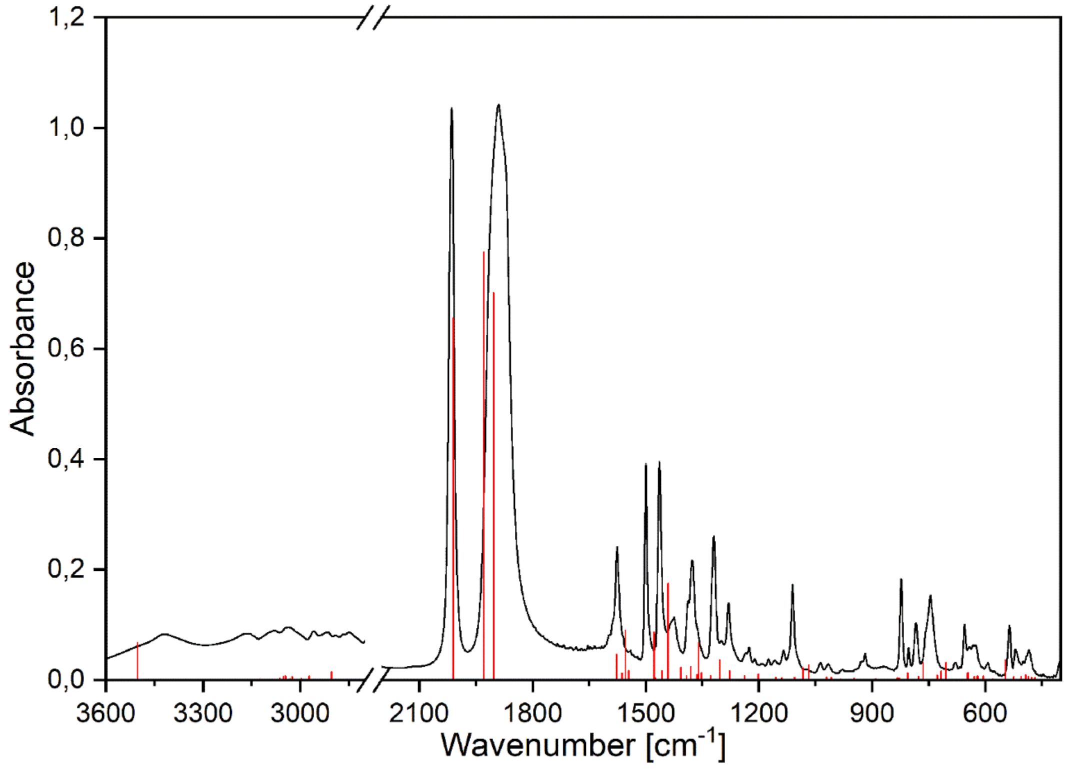

Fig. S18. Experimental and PBE0/def2-TZVP simulated (vertical lines) FTIR spectrum of $[Re(CO)_3(Q)MeHim]$ (**2**).

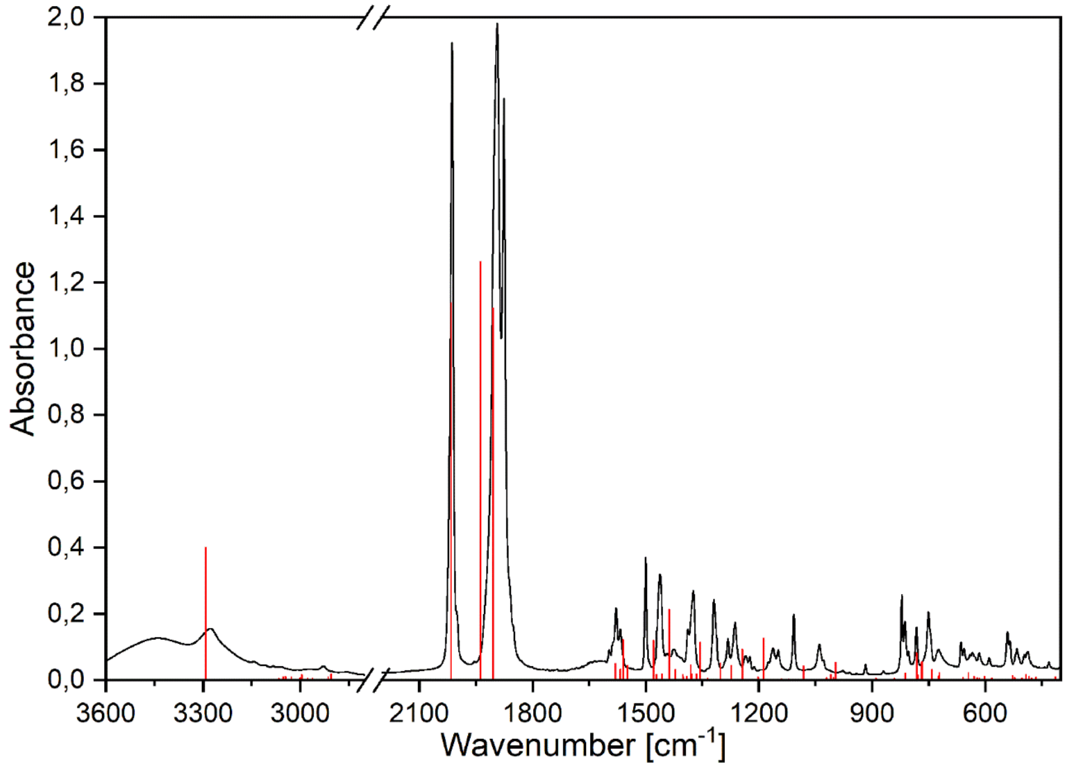

Fig. S19. Experimental and PBE0/def2-TZVP simulated (vertical lines) FTIR spectrum of $[Re(CO)_3(Q)Hdmpz]$ (**3**).

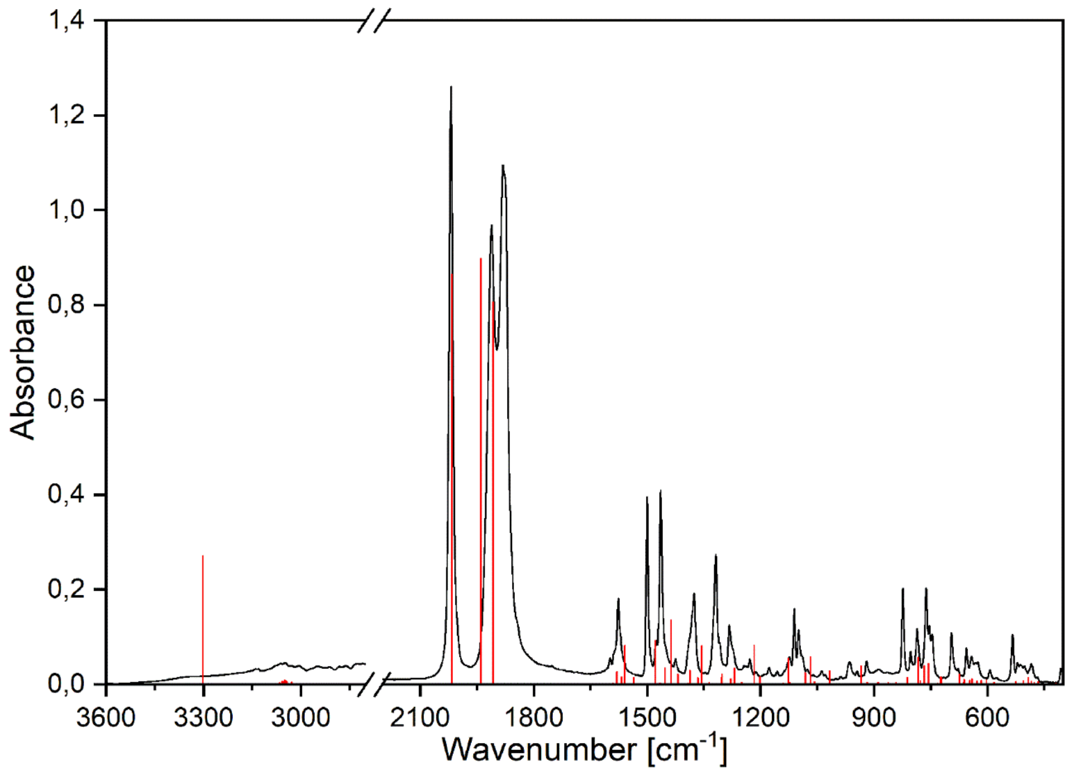

Fig. S20. Experimental and PBE0/def2-TZVP simulated (vertical lines) FTIR spectrum of [Re(CO)$_3$(Q)HPhpz] (**4**).

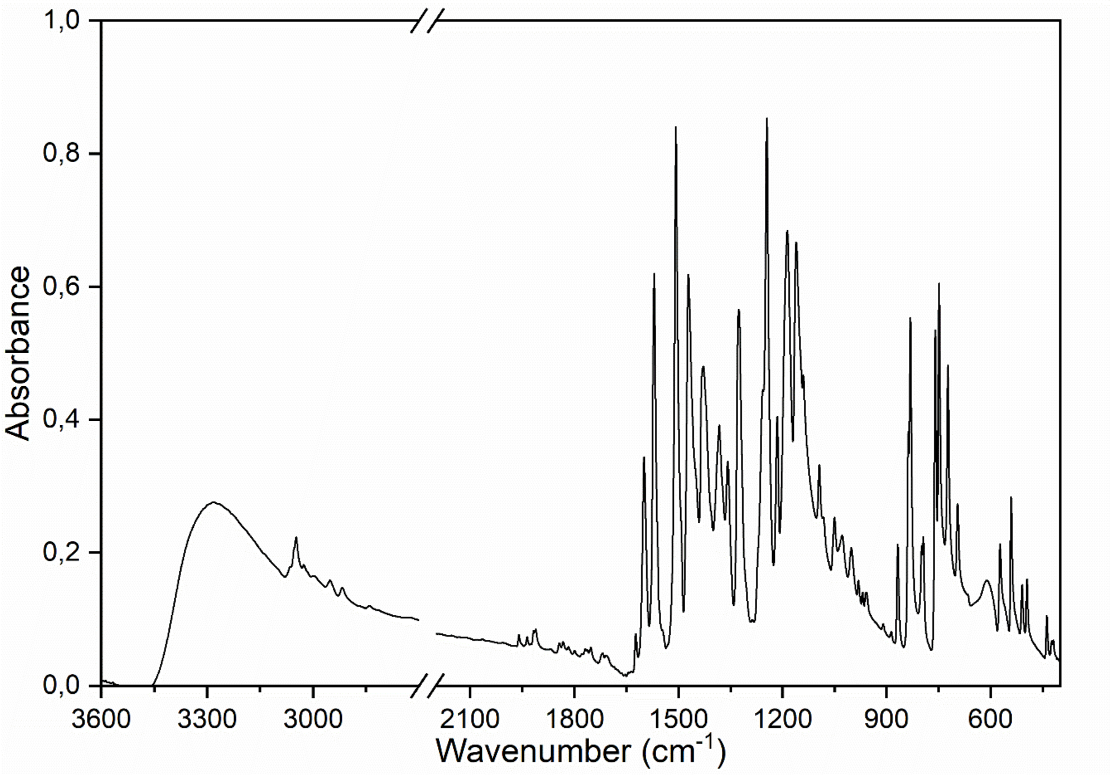

Fig. S21. FTIR spectrum of 2-methyl-8-hydroxyquinoline (MeHQ).

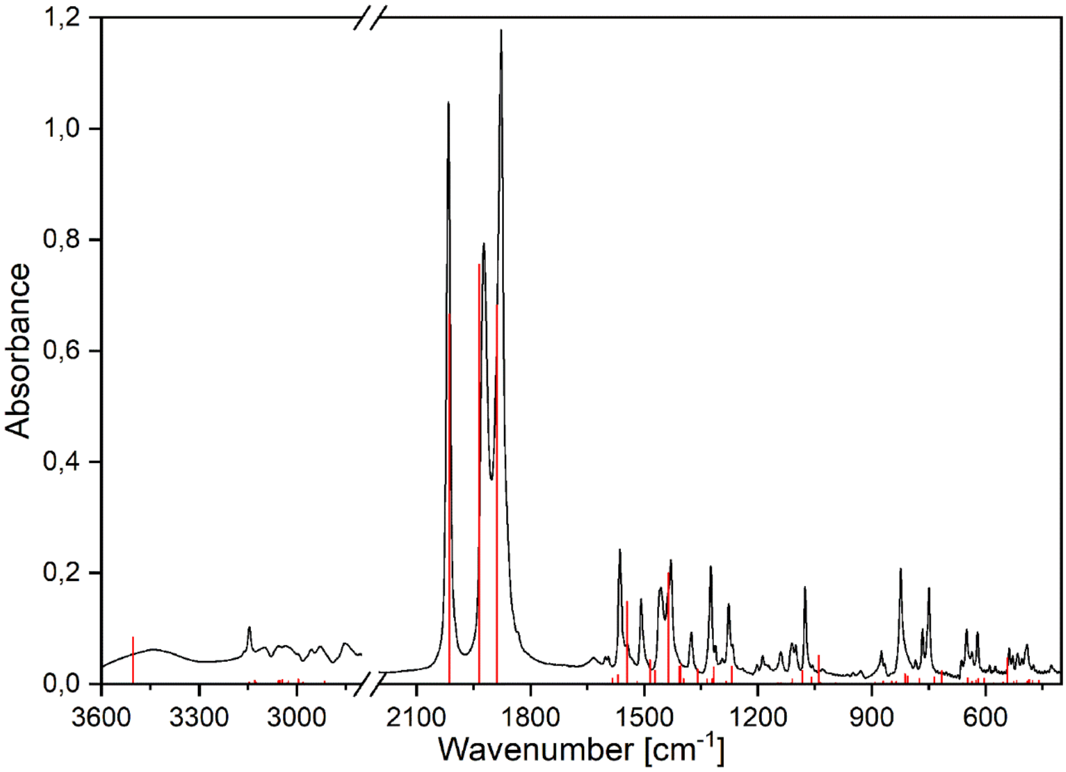

Fig. S22. Experimental and PBE0/def2-TZVP simulated (vertical lines) FTIR spectrum of $[Re(CO)_3(MeQ)Him]$ (**5**).

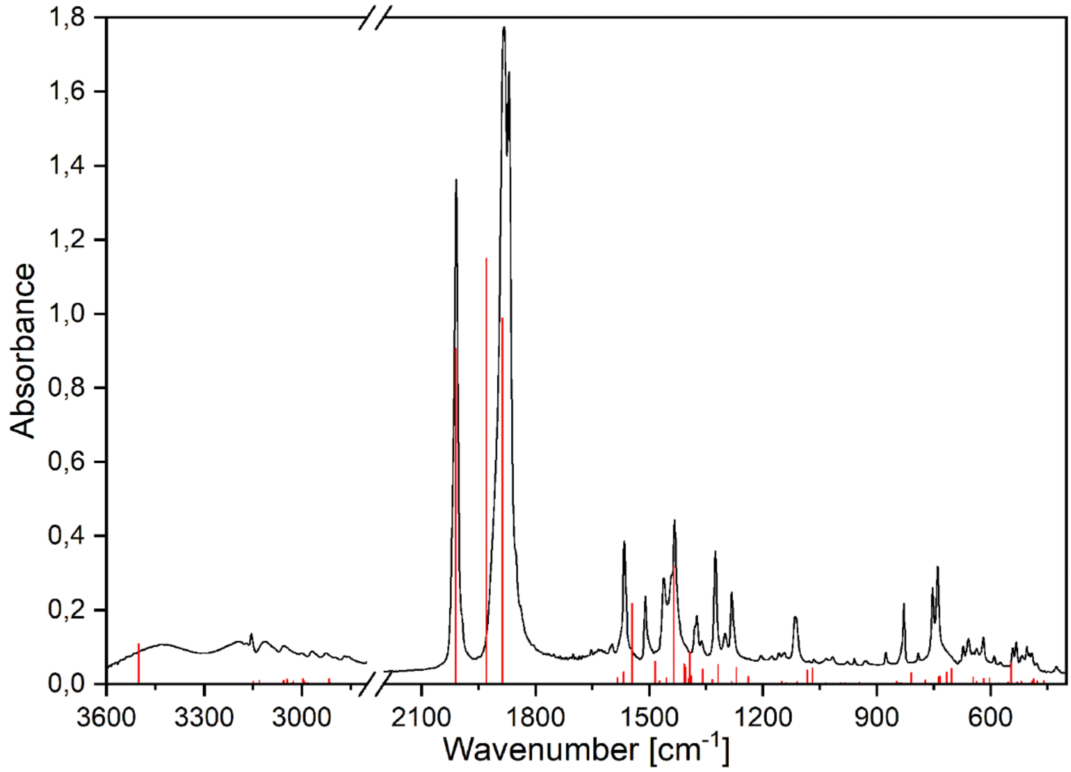

Fig. S23. Experimental and PBE0/def2-TZVP simulated (vertical lines) FTIR spectrum of [Re(CO)$_3$(MeQ)MeHim] (**6**).

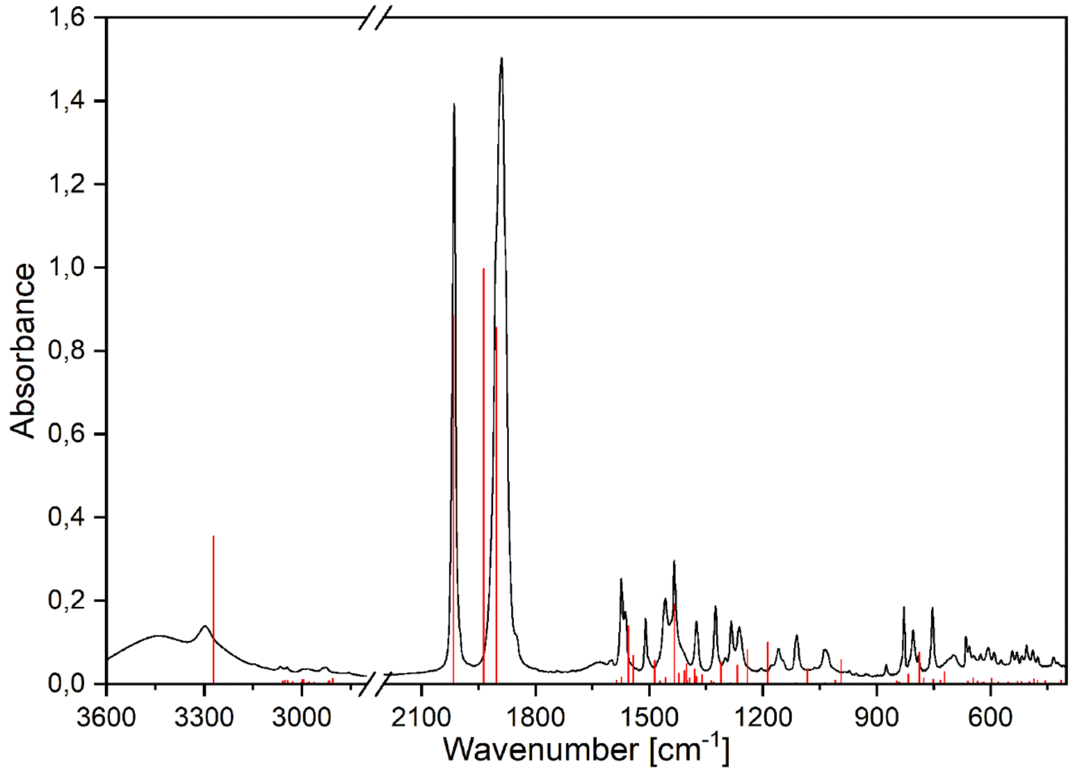

Fig. S24. Experimental and PBE0/def2-TZVP simulated (vertical lines) FTIR spectrum of [Re(CO)$_3$(MeQ)Hdmpz] (**7**).

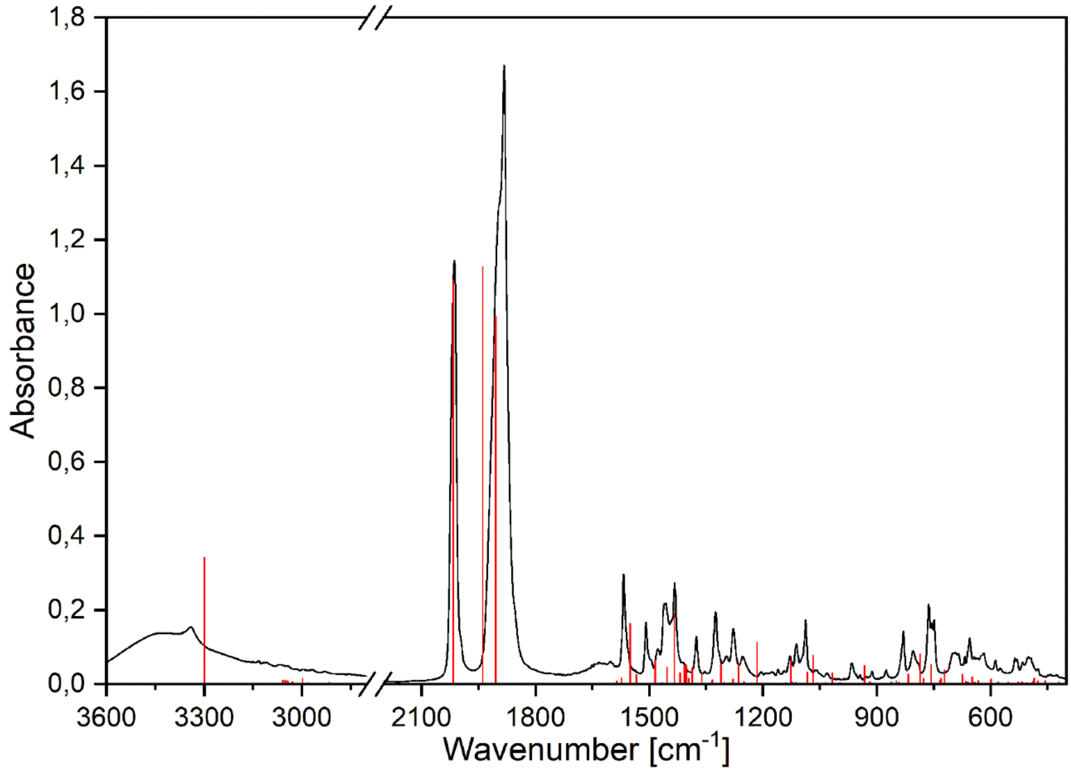

Fig. S25. Experimental and PBE0/def2-TZVP simulated (vertical lines) FTIR spectrum of [Re(CO)$_3$(MeQ)HPhpz] (**8**).

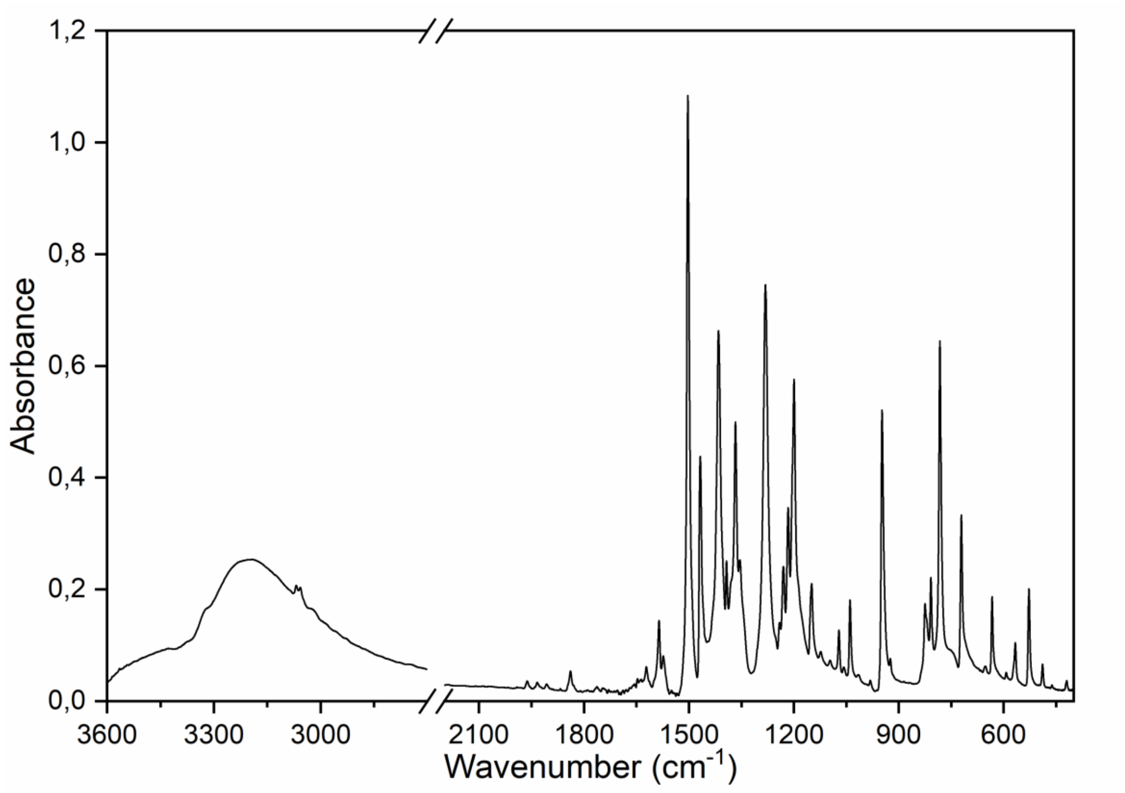

Fig. S26. FTIR spectrum of 5-chloro-8-hydroxyquinoline (ClHQ).

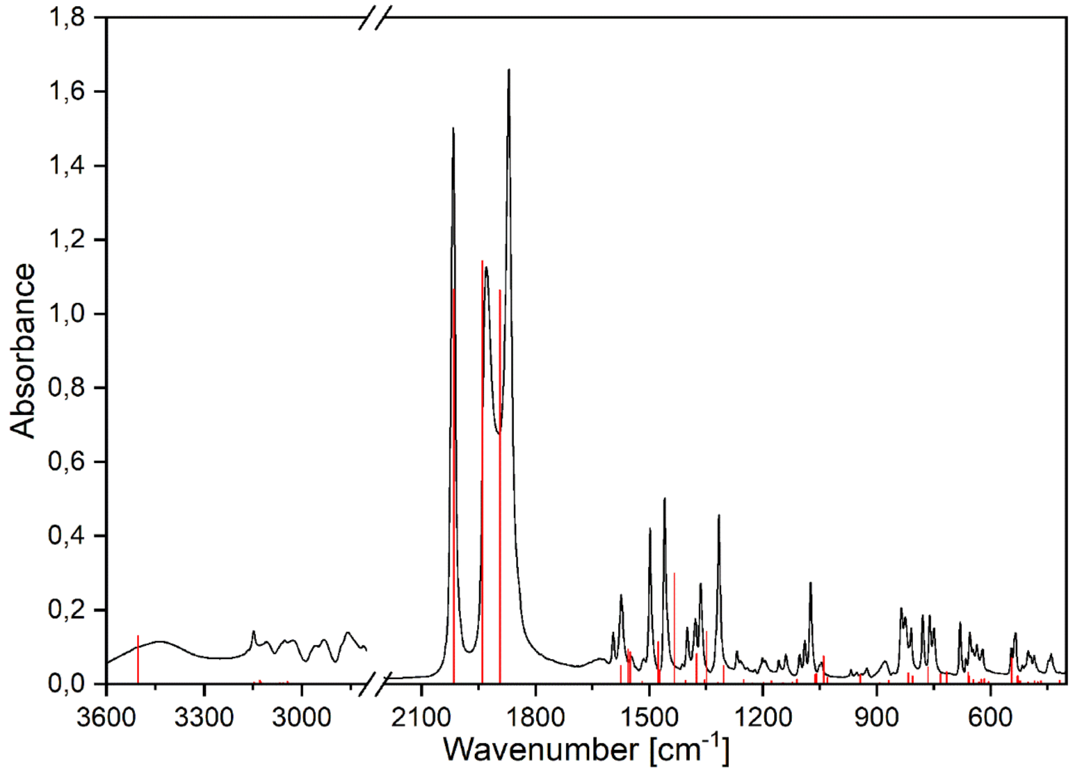

Fig. S27. Experimental and PBE0/def2-TZVP simulated (vertical lines) FTIR spectrum of $[Re(CO)_3(ClQ)Him]$ (**9**).

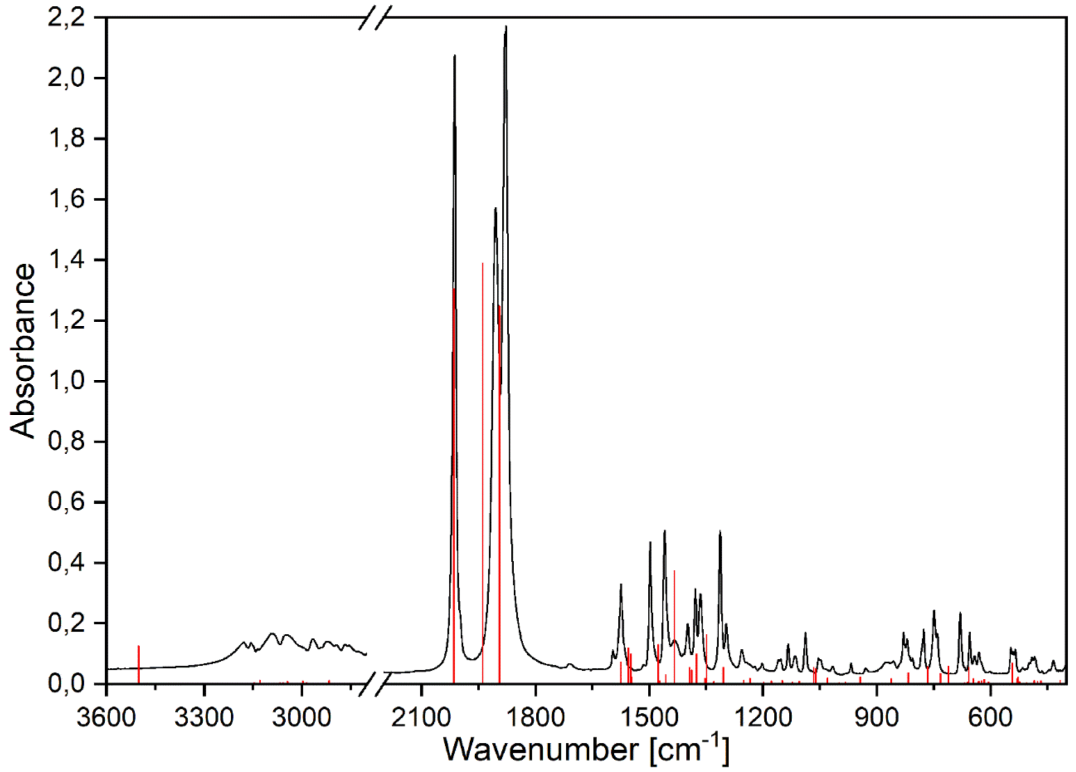

Fig. S28. Experimental and PBE0/def2-TZVP simulated (vertical lines) FTIR spectrum of [Re(CO)$_3$(ClQ)MeHim] (**10**).

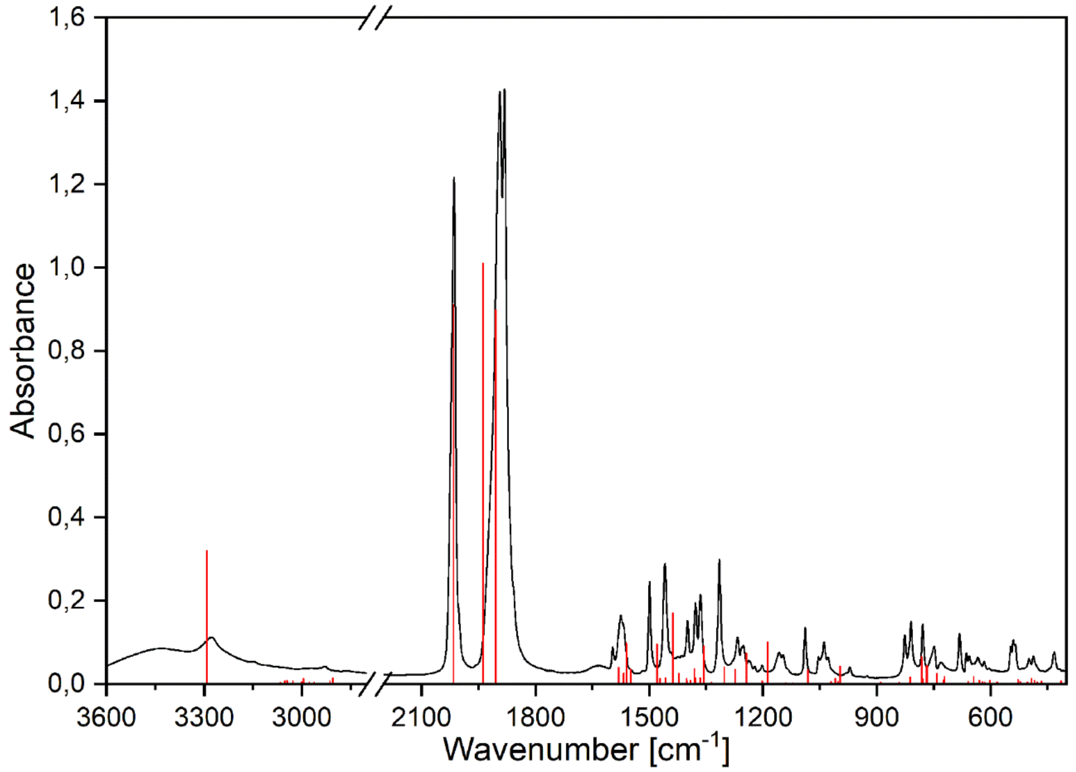

Fig. S29. Experimental and PBE0/def2-TZVP simulated (vertical lines) FTIR spectrum of [Re(CO)$_3$(ClQ)Hdmpz] (**11**).

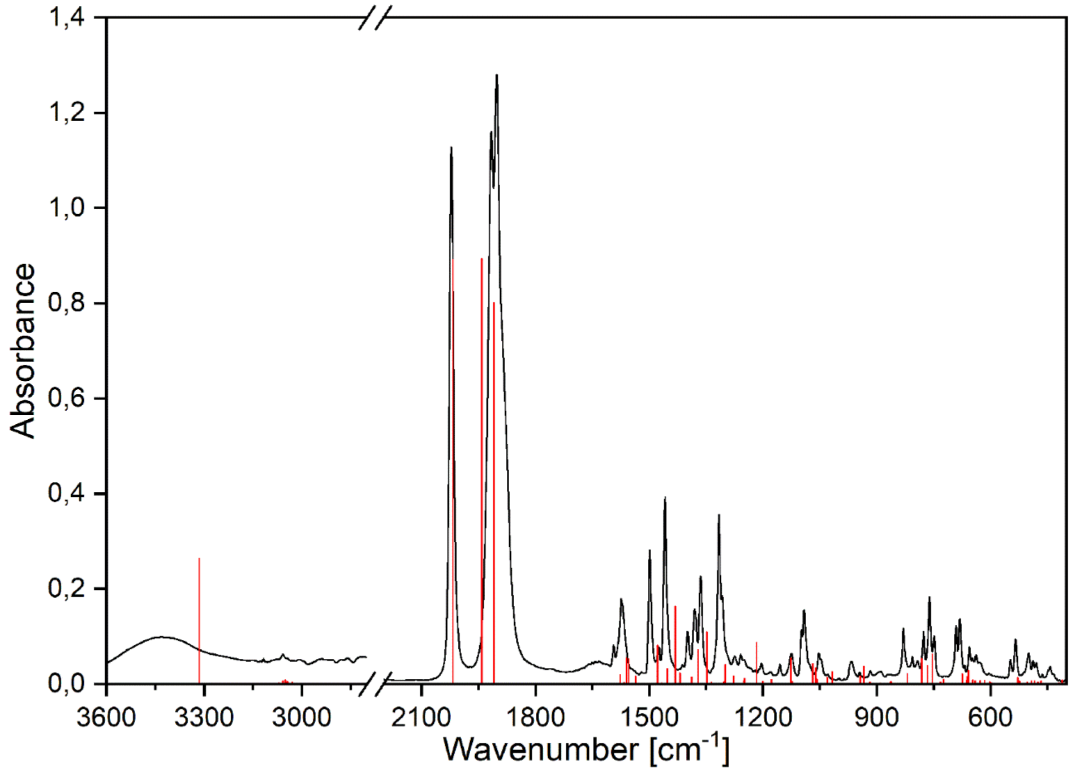

Fig. S30. Experimental and PBE0/def2-TZVP simulated (vertical lines) FTIR spectrum of [Re(CO)$_3$(ClQ)HPhpz] (**12**).

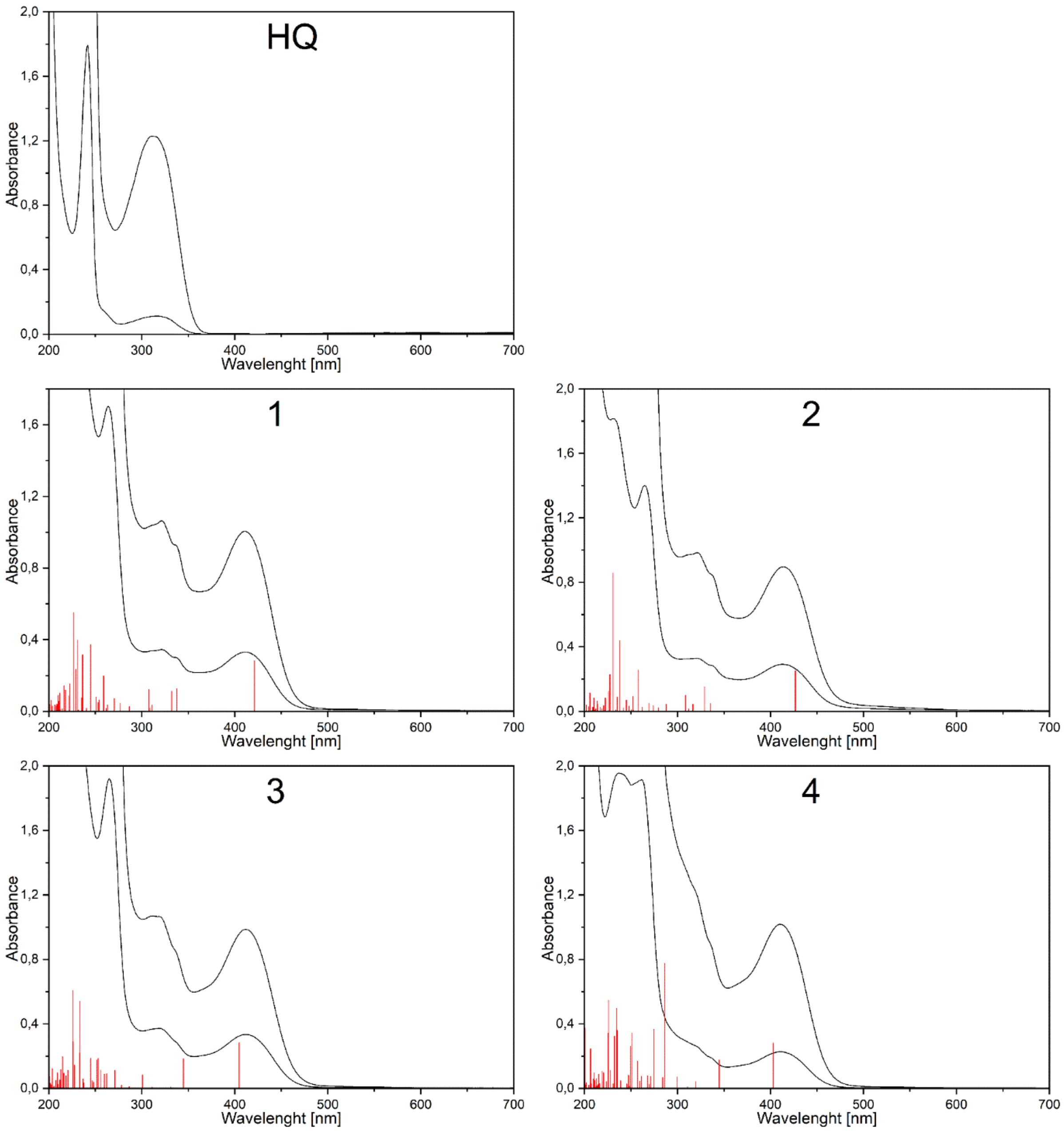

Fig. S31. Experimental UV-Vis absorption spectra (black lines) for 8-hydroxyquinoline (HQ) and its studied rhenium(I) complexes (**1–4**) in two different concentrated MeOH solutions. TD-DFT simulated spectra shown as red vertical lines.

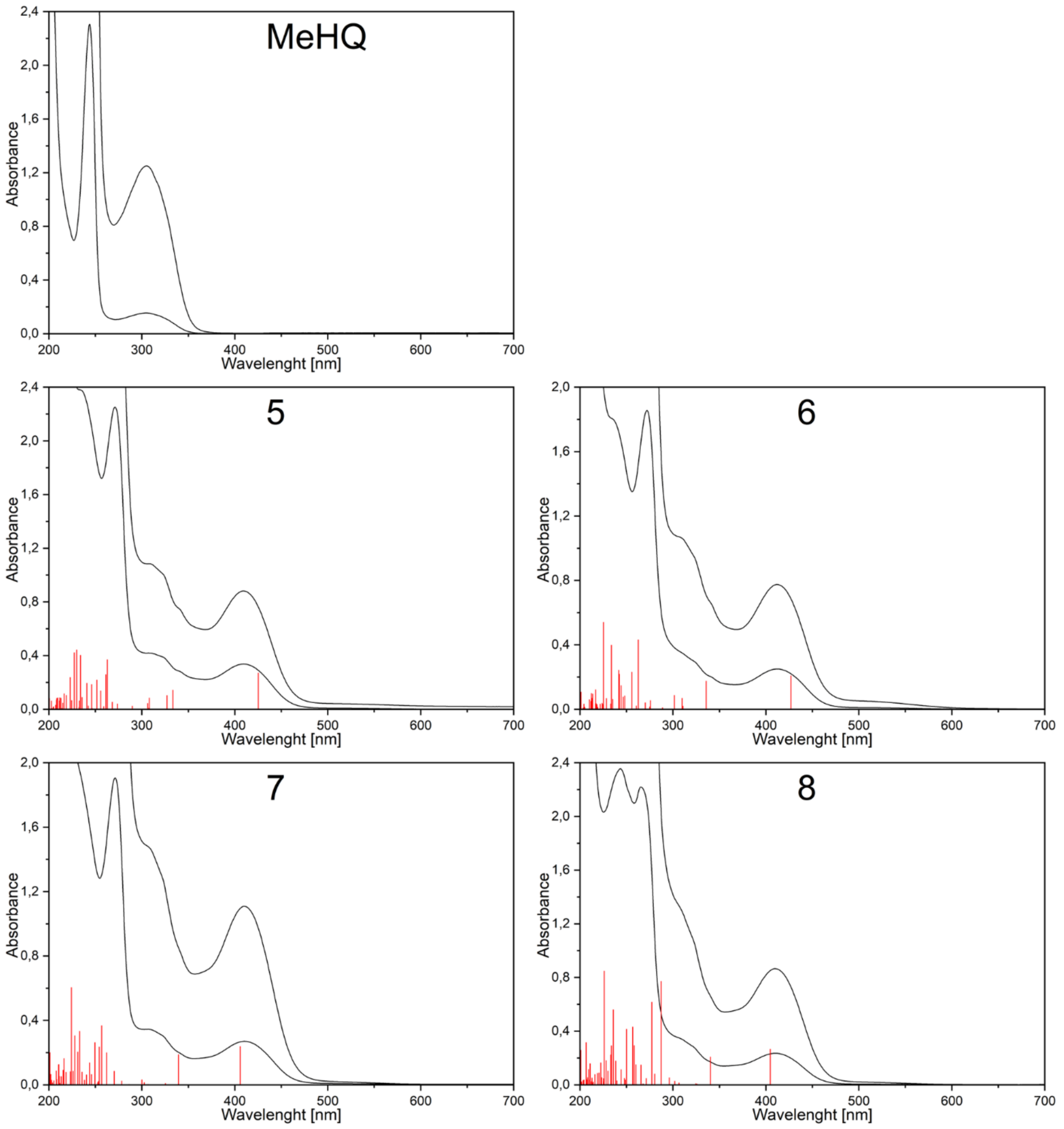

Fig. S32. Experimental UV-Vis absorption spectra (black lines) for 2-methyl-8-hydroxyquinoline (MeHQ) and its studied rhenium(I) complexes (**5–8**) in two different concentrated MeOH solutions. TD-DFT simulated spectra shown as red vertical lines.

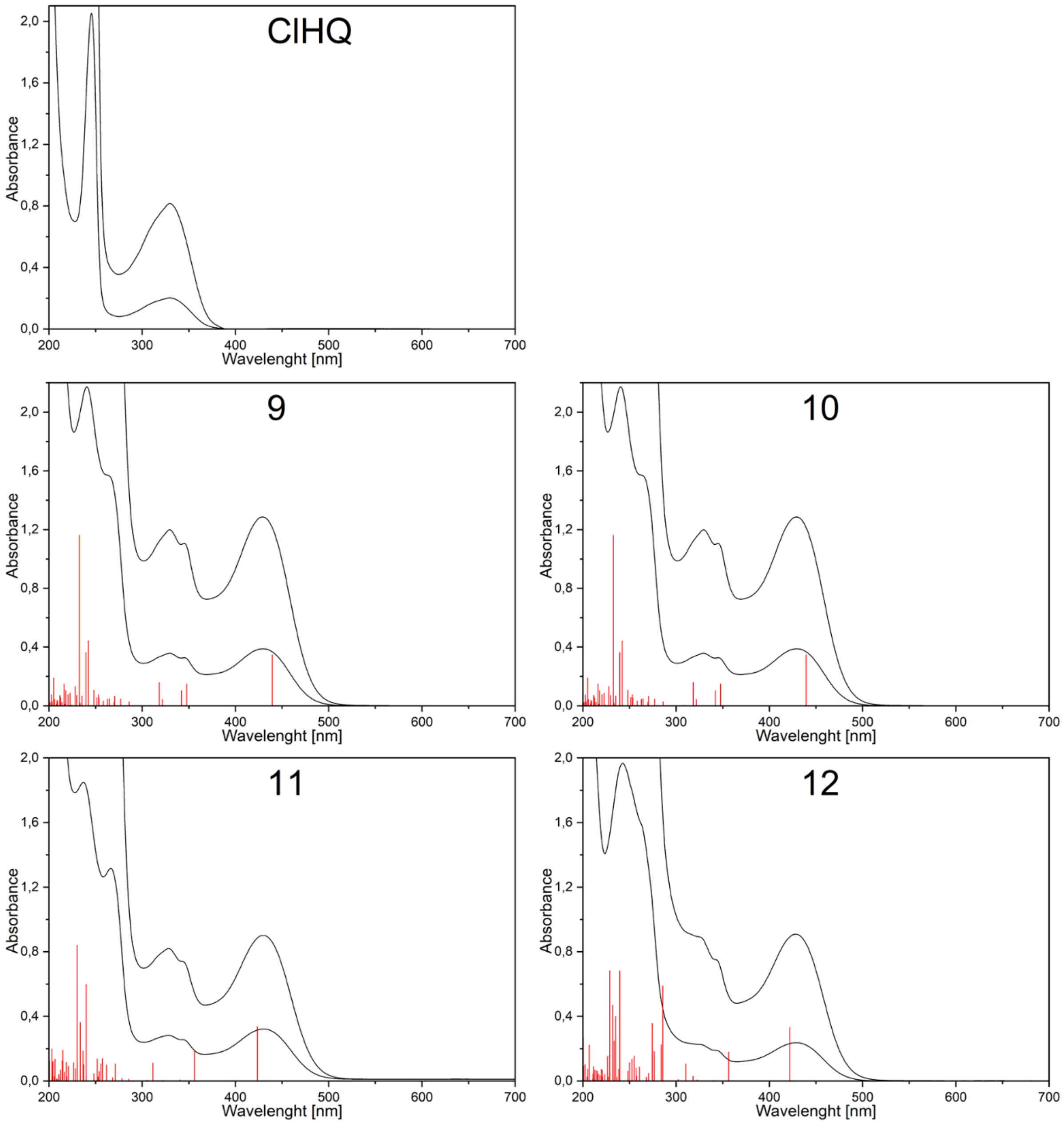

Fig. S33. Experimental UV-Vis absorption spectra (black lines) for 5-chloro-8-hydroxyquinoline (ClHQ) and its studied rhenium(I) complexes (**9–12**) in two different concentrated MeOH solutions. TD-DFT simulated spectra shown as red vertical lines.

Table S18. The selected experimental and TD-PBE0/def2-TZVP/IEFPCM(methanol) calculated absorptions of the studied rhenium(I) complexes.

Complex	Calculated			Experimental
	λ (nm)	Oscillator strength	Main components	λ (nm)
1	421.09	0.0866	H→L (97.8%)	411
	337.43	0.0387	H-1→L (78.0%)	337sh
			H-2→L (18.8%)	
	332.14	0.0342	H-2→L (78.5%)	
			H-1→L (17.7%)	
2	426.68	0.0857	H→L (98.0%)	414
	335.39	0.0164	H-1→L (63.6%)	338sh
			H-2→L (33.1%)	
	329.21	0.0512	H-2→L (63.9%)	
			H-1→L (30.8%)	
3	404.66	0.0922	H→L (97.0%)	412
	344.52	0.0596	H-1→L (95.3%)	337sh
	330.86	0.0025	H-2→L (97.1%)	
4	402.89	0.0911	H→L (96.6%)	411
	344.74	0.0574	H-1→L (93.9%)	337sh
	330.15	0.0011	H-2→L (97.1%)	
5	425.06	0.0730	H→L (98.3%)	409
	333.24	0.0384	H-1→L (82.4%)	342sh
			H-2→L (13.8%)	
	326.96	0.0278	H-2→L (82.5%)	
			H-1→L (13.0%)	
6	426.92	0.0677	H→L (98.3%)	412
	335.66	0.0567	H-1→L (95.3%)	342sh
	330.72	0.0006	H-2→L (97.4%)	
7	405.64	0.0772	H→L (97.6%)	410
	339.30	0.0609	H-1→L (95.4%)	323sh
	325.27	0.0029	H-2→L (96.0%)	
8	404.73	0.0719	H→L (97.6%)	410
	340.37	0.0559	H-1→L (94.2%)	
	325.81	0.0017	H-2→L (94.3%)	
9	439.35	0.1025	H→L (98.0%)	429
	347.72	0.0438	H-1→L (82.3%)	345sh
			H-2→L (14.1%)	
	342.07	0.0303	H-2→L (83.6%)	
			H-1→L (13.2%)	
10	443.56	0.0997	H→L (98.1%)	433
	348.27	0.0353	H-1→L (81.5%)	346sh
			H-2→L (13.9%)	
	341.98	0.0361	H-2→L (82.9%)	
			H-1→L (13.4%)	
11	423.37	0.1091	H→L (97.3%)	430
	356.12	0.0609	H-1→L (95.8%)	345sh
	340.78	0.0024	H-2→L (97.6%)	
12	421.72	0.1080	H→L (97.1%)	428
	356.27	0.0583	H-1→L (94.4%)	344sh
	339.27	0.0012	H-2→L (97.7%)	

Table S19. Calculated transitions (with a contribution greater than 5%) for [Re(CO)$_3$(Q)Him] (**1**)

Wavelength [nm]	Oscillator strength	Main components	%
421.09	0.0866	HOMO → LUMO	97.83
337.43	0.0387	HOMO-1 → LUMO	78.03
		HOMO-2 → LUMO	18.80
332.14	0.0342	HOMO-2 → LUMO	78.51
		HOMO-1 → LUMO	17.70
310.70	0.0103	HOMO-3 → LUMO	76.76
		HOMO → LUMO+2	16.53
308.76	0.0056	HOMO → LUMO+2	61.78
		HOMO-3 → LUMO	20.76
		HOMO-3 → LUMO+2	6.01
		HOMO → LUMO+1	5.22
307.46	0.0369	HOMO → LUMO+1	77.66
		HOMO-5 → LUMO	12.91
		HOMO → LUMO+2	6.48
286.49	0.0081	HOMO → LUMO+3	59.73
		HOMO-1 → LUMO+2	27.49
276.95	0.0138	HOMO-1 → LUMO+2	38.27
		HOMO-2 → LUMO+2	26.17
		HOMO-1 → LUMO+3	18.90
		HOMO → LUMO+3	7.38
270.33	0.0216	HOMO-2 → LUMO+2	25.61
		HOMO-1 → LUMO+2	21.83
		HOMO → LUMO+3	20.18
		HOMO-1 → LUMO+3	13.63
263.01	0.0104	HOMO-2 → LUMO+3	46.02
		HOMO-1 → LUMO+3	33.93
		HOMO-2 → LUMO+4	21.73
		HOMO-2 → LUMO+2	6.88
261.63	0.0049	HOMO-4 → LUMO	99.02
258.90	0.0603	HOMO-1 → LUMO+1	42.21
		HOMO-5 → LUMO	29.90
		HOMO → LUMO+4	6.79
		HOMO → LUMO+1	6.14
		HOMO-2 → LUMO+1	6.05
		HOMO-3 → LUMO+1	5.11
254.11	0.0193	HOMO → LUMO+4	29.58
		HOMO-2 → LUMO+3	19.30
		HOMO-1 → LUMO+1	9.98
		HOMO-1 → LUMO+3	9.02
		HOMO-2 → LUMO+2	8.77
252.84	0.0146	HOMO-2 → LUMO+3	18.15
		HOMO-1 → LUMO+1	15.85
		HOMO-2 → LUMO+2	13.31
		HOMO-3 → LUMO+2	5.78
		HOMO-1 → LUMO+3	5.93
250.60	0.0244	HOMO-2 → LUMO+1	46.72
		HOMO-1 → LUMO+1	18.82
		HOMO-3 → LUMO+2	8.72
		HOMO → LUMO+4	7.86
		HOMO-5 → LUMO	7.42
248.60	0.0008	HOMO-3 → LUMO+2	55.03
		HOMO-2 → LUMO+1	14.42
		HOMO → LUMO+4	11.84
		HOMO → LUMO+2	5.57
244.93	0.1134	HOMO-2 → LUMO+1	25.58
		HOMO-5 → LUMO	20.57
		HOMO-3 → LUMO+1	16.99
		HOMO → LUMO+4	10.97

		HOMO-1 → LUMO+1	9.62
		HOMO-3 → LUMO+2	5.10
240.33	0.0053	HOMO → LUMO+5	47.10
		HOMO-3 → LUMO+3	16.42
		HOMO → LUMO+6	9.93
236.24	0.0961	HOMO-3 → LUMO+1	42.08
		HOMO → LUMO+6	15.24
		HOMO-3 → LUMO+3	10.66
		HOMO-5 → LUMO	10.55
		HOMO → LUMO+5	8.07
235.00	0.0230	HOMO-3 → LUMO+3	30.54
		HOMO-1 → LUMO+4	11.77
		HOMO → LUMO+6	11.71
		HOMO-1 → LUMO+5	5.87
231.87	0.0021	HOMO-6 → LUMO	84.67
230.93	0.1212	HOMO → LUMO+7	38.66
		HOMO-6 → LUMO	7.54
		HOMO-3 → LUMO+1	7.12
		HOMO-3 → LUMO+3	5.22
229.04	0.0716	HOMO-1 → LUMO+4	25.12
		HOMO → LUMO+7	14.48
		HOMO → LUMO+6	12.11
		HOMO-2 → LUMO+4	10.16
		HOMO-1 → LUMO+7	6.84
		HOMO-2 → LUMO+5	5.22
226.71	0.1675	HOMO → LUMO+6	25.93
		HOMO-1 → LUMO+4	13.28
		HOMO-3 → LUMO+1	9.75
		HOMO → LUMO+5	9.17
		HOMO-1 → LUMO+7	7.44
		HOMO → LUMO+7	6.27
		HOMO-2 → LUMO+4	5.12
222.53	0.0470	HOMO-2 → LUMO+5	29.29
		HOMO-2 → LUMO+4	23.13
		HOMO-2 → LUMO+7	11.41
221.12	0.0261	HOMO → LUMO+8	35.48
		HOMO-2 → LUMO+4	9.40
		HOMO → LUMO+10	8.84
		HOMO → LUMO+11	6.15
		HOMO-3 → LUMO+8	5.10
217.82	0.0361	HOMO-1 → LUMO+7	13.04
		HOMO-1 → LUMO+4	9.91
		HOMO-2 → LUMO+5	8.05
		HOMO-2 → LUMO+8	7.75
		HOMO → LUMO+10	7.53
		HOMO-1 → LUMO+5	6.52
		HOMO → LUMO+8	5.60
		HOMO-1 → LUMO+8	5.60
		HOMO-3 → LUMO+3	5.22
216.29	0.0433	HOMO-2 → LUMO+4	20.08
		HOMO-1 → LUMO+4	10.72
		HOMO → LUMO+10	9.98
		HOMO-1 → LUMO+5	7.77
		HOMO-2 → LUMO+10	7.02
		HOMO-2 → LUMO+5	5.20
		HOMO → LUMO+7	5.14
214.33	0.0036	HOMO-1 → LUMO+7	20.55
		HOMO-1 → LUMO+8	15.69
		HOMO-1 → LUMO+4	10.99
		HOMO-2 → LUMO+8	8.85
		HOMO-1 → LUMO+5	7.64
212.56	0.0026	HOMO-8 → LUMO	46.92

		HOMO-9 → LUMO	24.98
		HOMO-7 → LUMO	10.96
211.52	0.0305	HOMO-3 → LUMO+4	42.85
		HOMO-3 → LUMO+5	6.38
		HOMO → LUMO+9	5.88
210.86	0.0013	HOMO-4 → LUMO+1	20.33
		HOMO-7 → LUMO	19.23
		HOMO-5 → LUMO+1	11.55
		HOMO-1 → LUMO+5	8.07
		HOMO-8 → LUMO	6.20
		HOMO-1 → LUMO+10	5.17
210.54	0.0169	HOMO-4 → LUMO+1	25.06
		HOMO-1 → LUMO+10	18.06
		HOMO-1 → LUMO+6	14.42
		HOMO-7 → LUMO	8.88
		HOMO-1 → LUMO+5	8.82
209.63	0.0272	HOMO-4 → LUMO+1	49.25
		HOMO-7 → LUMO	23.85
		HOMO-5 → LUMO+1	9.28
208.65	0.0116	HOMO-4 → LUMO+2	18.00
		HOMO-2 → LUMO+10	9.09
		HOMO-2 → LUMO+7	8.70
		HOMO-3 → LUMO+4	7.24
		HOMO → LUMO+8	7.17
		HOMO-2 → LUMO+4	5.90
208.35	0.0088	HOMO → LUMO+9	66.42
		HOMO-3 → LUMO+4	11.90
207.86	0.0086	HOMO-4 → LUMO+2	76.26
206.35	0.0104	HOMO → LUMO+11	25.28
		HOMO → LUMO+8	16.40
		HOMO-3 → LUMO+4	12.05
		HOMO → LUMO+10	8.88
		HOMO → LUMO+9	8.47
203.77	0.0091	HOMO-2 → LUMO+7	29.68
		HOMO-1 → LUMO+10	13.18
		HOMO-2 → LUMO+11	6.83
		HOMO-2 → LUMO+4	6.79
		HOMO-2 → LUMO+6	6.18
		HOMO → LUMO+11	5.46
202.18	0.0189	HOMO-3 → LUMO+5	33.23
		HOMO-3 → LUMO+6	11.75
		HOMO-1 → LUMO+10	9.51
		HOMO → LUMO+11	7.26
		HOMO-3 → LUMO+4	6.33
200.75	0.0119	HOMO-4 → LUMO+3	47.23
		HOMO-2 → LUMO+6	30.24
		HOMO → LUMO+10	10.14
		HOMO-1 → LUMO+6	6.10

Table S20. Calculated transitions (with a contribution greater than 5%) for [Re(CO)$_3$(Q)MeHim] (**2**)

Wavelength [nm]	Oscillator strength	Main components	%
426.68	0.0857	HOMO → LUMO	98.02
335.39	0.0164	HOMO-1 → LUMO	63.60
		HOMO-2 → LUMO	33.09
329.21	0.0512	HOMO-2 → LUMO	63.85
		HOMO-1 → LUMO	30.78
316.51	0.0147	HOMO-3 → LUMO	93.31
311.97	0.0050	HOMO → LUMO+2	70.34
		HOMO → LUMO+1	11.47
		HOMO-3 → LUMO+2	5.91
308.70	0.0337	HOMO → LUMO+1	72.26
		HOMO-5 → LUMO	11.58
		HOMO → LUMO+2	11.34
287.78	0.0145	HOMO → LUMO+3	71.25
		HOMO-1 → LUMO+2	13.06
279.36	0.0072	HOMO-4 → LUMO	96.71
273.87	0.0120	HOMO-1 → LUMO+2	42.63
		HOMO-2 → LUMO+2	34.05
		HOMO-1 → LUMO+3	6.57
269.04	0.0171	HOMO-1 → LUMO+2	26.92
		HOMO-2 → LUMO+2	24.33
		HOMO-1 → LUMO+3	18.64
		HOMO → LUMO+3	10.23
		HOMO → LUMO+2	5.18
261.91	0.0080	HOMO-1 → LUMO+3	45.79
		HOMO-2 → LUMO+3	31.19
		HOMO-2 → LUMO+2	6.90
		HOMO-3 → LUMO+3	5.51
257.65	0.0868	HOMO-5 → LUMO	42.20
		HOMO-1 → LUMO+1	19.44
		HOMO-2 → LUMO+1	15.42
		HOMO-3 → LUMO+1	10.21
		HOMO → LUMO+1	7.61
252.23	0.0031	HOMO-3 → LUMO+2	55.63
		HOMO → LUMO+4	20.49
		HOMO → LUMO+2	6.59
251.94	0.0309	HOMO-2 → LUMO+3	45.12
		HOMO-2 → LUMO+2	13.26
		HOMO-1 → LUMO+3	9.70
		HOMO → LUMO+5	6.69
250.42	0.0033	HOMO-1 → LUMO+1	68.27
		HOMO-2 → LUMO+1	22.37
247.74	0.0111	HOMO → LUMO+4	58.88
		HOMO-3 → LUMO+2	18.16

		HOMO-3 → LUMO+3	6.03
244.95	0.0239	HOMO-2 → LUMO+1	49.11
		HOMO-3 → LUMO+1	24.00
		HOMO-5 → LUMO	8.66
		HOMO-1 → LUMO+1	6.87
241.64	0.0070	HOMO → LUMO+5	40.49
		HOMO-3 → LUMO+1	11.71
		HOMO-3 → LUMO+3	9.65
		HOMO → LUMO+6	9.23
237.97	0.1483	HOMO-3 → LUMO+1	29.39
		HOMO → LUMO+6	25.05
		HOMO-5 → LUMO	16.55
		HOMO → LUMO+5	5.83
235.01	0.0302	HOMO-3 → LUMO+3	47.73
		HOMO-2 → LUMO+5	7.64
		HOMO → LUMO+6	5.98
233.43	0.0031	HOMO-6 → LUMO	91.04
230.57	0.2924	HOMO → LUMO+6	19.55
		HOMO-3 → LUMO+1	16.09
		HOMO → LUMO+5	12.25
		HOMO → LUMO+7	10.50
		HOMO-5 → LUMO	9.59
227.22	0.0773	HOMO-1 → LUMO+4	20.44
		HOMO → LUMO+7	18.45
		HOMO-2 → LUMO+5	8.91
		HOMO → LUMO+9	8.84
		HOMO → LUMO+6	8.83
225.83	0.0424	HOMO-1 → LUMO+4	23.73
		HOMO → LUMO+7	13.61
		HOMO-2 → LUMO+4	9.81
		HOMO-1 → LUMO+5	9.75
		HOMO → LUMO+6	6.00
		HOMO-1 → LUMO+7	5.39
222.22	0.0273	HOMO-2 → LUMO+4	36.53
		HOMO → LUMO+7	11.58
		HOMO-1 → LUMO+4	7.61
220.76	0.0075	HOMO → LUMO+9	27.91
		HOMO → LUMO+7	9.91
		HOMO → LUMO+8	9.56
		HOMO → LUMO+10	7.33
		HOMO → LUMO+11	7.26
		HOMO-3 → LUMO+9	6.57
220.45	0.0121	HOMO-4 → LUMO+1	89.88
		HOMO-4 → LUMO+2	6.11
218.42	0.0042	HOMO-4 → LUMO+2	82.90
		HOMO-4 → LUMO+1	6.91

217.23	0.0078	HOMO-2 → LUMO+5	25.30
		HOMO-2 → LUMO+4	11.94
		HOMO → LUMO+7	11.34
		HOMO → LUMO+10	10.82
213.82	0.0150	HOMO → LUMO+10	22.70
		HOMO → LUMO+10	13.26
		HOMO-2 → LUMO+6	6.57
		HOMO-1 → LUMO+6	5.44
		HOMO-2 → LUMO+5	5.39
		HOMO-5 → LUMO+1	5.35
213.27	0.0218	HOMO-1 → LUMO+5	18.05
		HOMO → LUMO+9	11.00
		HOMO → LUMO+7	8.52
		HOMO-3 → LUMO+4	8.45
		HOMO-4 → LUMO+3	8.15
		HOMO-2 → LUMO+9	5.41
211.90	0.0053	HOMO-9 → LUMO	57.37
		HOMO-8 → LUMO	29.87
		HOMO-7 → LUMO	5.42
210.61	0.0026	HOMO → LUMO+8	61.88
		HOMO → LUMO+9	10.62
210.10	0.0277	HOMO-7 → LUMO	49.49
		HOMO-5 → LUMO+1	13.58
		HOMO → LUMO+8	8.15
209.30	0.0072	HOMO-4 → LUMO+3	53.70
		HOMO-1 → LUMO+5	7.83
		HOMO-7 → LUMO	6.11
208.88	0.0082	HOMO-4 → LUMO+3	18.60
		HOMO-3 → LUMO+5	16.33
		HOMO → LUMO+11	9.99
		HOMO-1 → LUMO+6	8.24
		HOMO-3 → LUMO+4	7.81
		HOMO-1 → LUMO+5	6.09
		HOMO-1 → LUMO+10	5.07
207.70	0.0014	HOMO-3 → LUMO+5	13.64
		HOMO-3 → LUMO+4	11.90
		HOMO-1 → LUMO+9	9.99
		HOMO-3 → LUMO+6	9.08
		HOMO-2 → LUMO+5	8.19
		HOMO-2 → LUMO+4	5.43
205.86	0.0381	HOMO-3 → LUMO+4	38.59
		HOMO-1 → LUMO+9	14.18
		HOMO → LUMO+11	13.98
		HOMO → LUMO+10	7.46
		HOMO → LUMO+9	5.63
204.44	0.0096	HOMO-1 → LUMO+9	18.72

		HOMO-1 → LUMO+7	18.32
		HOMO-1 → LUMO+4	17.16
		HOMO → LUMO+11	8.30
		HOMO-3 → LUMO+5	6.31
201.83	0.0133	HOMO → LUMO+11	22.32
		HOMO-1 → LUMO+7	14.63
		HOMO-3 → LUMO+5	10.38
		HOMO-1 → LUMO+11	7.25
		HOMO-1 → LUMO+4	6.32
		HOMO-2 → LUMO+11	6.26
200.18	0.0370	HOMO-1 → LUMO+6	18.98
		HOMO → LUMO+11	11.80
		HOMO → LUMO+13	10.61
		HOMO-1 → LUMO+7	8.37

Table S21. Calculated transitions (with a contribution greater than 5%) for [Re(CO)$_3$(Q)Hdmpz] (**3**)

Wavelength [nm]	Oscillator strength	Main components	%
404.66	0.0922	HOMO → LUMO	96.95
344.52	0.0596	HOMO-1 → LUMO	95.29
330.86	0.0025	HOMO-2 → LUMO	97.09
310.87	0.0026	HOMO-3 → LUMO	94.17
305.06	0.0006	HOMO → LUMO+2	73.07
		HOMO-3 → LUMO+2	7.43
		HOMO-1 → LUMO+2	5.94
300.63	0.0267	HOMO → LUMO+1	74.73
		HOMO-5 → LUMO	16.69
286.43	0.0030	HOMO-1 → LUMO+2	58.49
		HOMO → LUMO+3	25.25
278.06	0.0064	HOMO-1 → LUMO+3	39.35
		HOMO-2 → LUMO+2	24.07
		HOMO → LUMO+3	18.29
270.92	0.0364	HOMO → LUMO+3	42.57
		HOMO-2 → LUMO+2	20.00
		HOMO-1 → LUMO+2	18.26
262.15	0.0292	HOMO-2 → LUMO+3	35.18
		HOMO-1 → LUMO+3	18.87
		HOMO-2 → LUMO+2	10.05
		HOMO-1 → LUMO+1	9.68
		HOMO-2 → LUMO+4	5.48
259.45	0.0286	HOMO-1 → LUMO+1	70.46
		HOMO-5 → LUMO	9.17
		HOMO-2 → LUMO+3	6.39
255.57	0.0368	HOMO-2 → LUMO+3	36.79
		HOMO-2 → LUMO+2	26.06
		HOMO-1 → LUMO+3	13.81
254.92	0.0006	HOMO-4 → LUMO	97.85
252,83	0.0603	HOMO → LUMO+4	29.59
		HOMO-5 → LUMO	25.16
		HOMO-3 → LUMO+2	8.63
		HOMO-1 → LUMO+1	8.57
		HOMO → LUMO+1	6.15
		HOMO-3 → LUMO+1	6.59
251.66	0.0572	HOMO → LUMO+4	25.24
		HOMO-3 → LUMO+2	20.90
		HOMO-5 → LUMO	17.42
		HOMO-3 → LUMO+1	9.68
		HOMO-1 → LUMO+1	5.52
		HOMO → LUMO+2	5.44
248.09	0.0121	HOMO-3 → LUMO+2	39.84
		HOMO → LUMO+4	28.28

		HOMO-6 → LUMO	15.46
246.70	0.0154	HOMO-6 → LUMO	29.34
		HOMO-3 → LUMO+2	2.58
		HOMO-2 → LUMO+1	59.93
245.00	0.0602	HOMO-6 → LUMO	38.05
		HOMO-2 → LUMO+1	26.76
		HOMO-3 → LUMO+1	12.73
		HOMO-3 → LUMO+2	6.19
237.46	0.0110	HOMO → LUMO+5	28.45
		HOMO-3 → LUMO+3	23.89
		HOMO → LUMO+6	21.18
		HOMO-5 → LUMO+6	6.71
236.72	0.0196	HOMO-1 → LUMO+4	49.78
		HOMO-3 → LUMO+3	19.92
		HOMO → LUMO+6	5.41
233.56	0.1755	HOMO-3 → LUMO+1	39.85
		HOMO → LUMO+6	19.71
		HOMO-5 → LUMO	13.60
233.24	0.0719	HOMO-3 → LUMO+3	17.45
		HOMO-2 → LUMO+4	16.88
		HOMO → LUMO+5	13.46
		HOMO-1 → LUMO+4	11.56
227.51	0.0465	HOMO-2 → LUMO+4	22.46
		HOMO-7 → LUMO	16.52
		HOMO-2 → LUMO+5	11.85
		HOMO → LUMO+5	9.07
		HOMO-2 → LUMO+6	6.90
226.14	0.0939	HOMO-7 → LUMO	65.59
		HOMO → LUMO+5	11.26
		HOMO → LUMO+6	6.13
225.44	0.1970	HOMO → LUMO+6	19.62
		HOMO-2 → LUMO+4	15.99
		HOMO → LUMO+5	13.47
		HOMO-7 → LUMO	12.11
		HOMO-3 → LUMO+1	8.99
220.43	0.0366	HOMO → LUMO+7	37.46
		HOMO → LUMO+9	16.95
		HOMO-3 → LUMO+7	5.59
		HOMO-3 → LUMO+9	5.15
218.42	0.0250	HOMO → LUMO+7	17.77
		HOMO-1 → LUMO+6	10.91
		HOMO-1 → LUMO+5	8.70
		HOMO-2 → LUMO+5	8.54
		HOMO → LUMO+9	6.26
		HOMO-1 → LUMO+7	6.22
		HOMO-2 → LUMO+9	5.12

216.06	0.0304	HOMO-2 → LUMO+5	28.46
		HOMO-2 → LUMO+10	11.15
		HOMO → LUMO+8	7.99
		HOMO-2 → LUMO+4	5.59
		HOMO → LUMO+5	5.09
214.69	0.0637	HOMO-3 → LUMO+4	26.11
		HOMO-1 → LUMO+7	15.86
		HOMO → LUMO+8	8.72
		HOMO → LUMO+7	7,25
		HOMO-1 → LUMO+6	5.90
212.74	0.0374	HOMO-1 → LUMO+5	37.08
		HOMO-3 → LUMO+4	14.92
		HOMO-1 → LUMO+7	13.58
		HOMO-1 → LUMO+10	6.48
		HOMO-1 → LUMO+6	5.64
211.84	0.0166	HOMO-1 → LUMO+7	27.98
		HOMO-1 → LUMO+9	21.11
		HOMO-2 → LUMO+6	8.55
		HOMO-2 → LUMO+10	7.23
210.17	0.0011	HOMO-9 → LUMO	91.82
210.08	0.0077	HOMO-3 → LUMO+4	30.41
		HOMO → LUMO+5	10.37
		HOMO → LUMO+6	9.03
		HOMO → LUMO+8	6.00
		HOMO-8 → LUMO	5.58
209.20	0.0316	HOMO-8 → LUMO	52.19
		HOMO-5 → LUMO+1	18.25
		HOMO-1 → LUMO+9	5.07
207.51	0.0164	HOMO-1 → LUMO+9	20.99
		HOMO-2 → LUMO+7	13.74
		HOMO-2 → LUMO+6	8.45
		HOMO → LUMO+9	8.13
		HOMO-2 → LUMO+9	6.82
		HOMO-8 → LUMO	6.37
205.76	0.0041	HOMO-4 → LUMO+1	38.04
		HOMO-4 → LUMO+2	35.00
		HOMO → LUMO+8	11.40
205.55	0.0029	HOMO → LUMO+8	59.08
		HOMO-4 → LUMO+1	13.89
		HOMO-4 → LUMO+2	5.29
205.01	0.0037	HOMO-4 → LUMO+2	46.86
		HOMO-4 → LUMO+1	46.22
203.66	0.0404	HOMO-6 → LUMO+2	49.33
		HOMO-5 → LUMO+2	24.18
		HOMO-4 → LUMO+2	8.22
202.57	0.0074	HOMO-3 → LUMO+6	18.38

		HOMO-1 → LUMO+9	17.55
		HOMO-1 → LUMO+6	15.79
		HOMO-3 → LUMO+5	13.29
		HOMO → LUMO+6	5.81
201.56	0.0102	HOMO-2 → LUMO+7	30.51
		HOMO-5 → LUMO+2	9.13
		HOMO-2 → LUMO+9	8.13
		HOMO-2 → LUMO+5	6.92
		HOMO-2 → LUMO+6	5.52
		HOMO-2 → LUMO+4	5.44
200.91	0.0114	HOMO-6 → LUMO+1	49.88
		HOMO-5 → LUMO+2	24.42
		HOMO-5 → LUMO+1	7.38
200.57	0.0241	HOMO-5 → LUMO+2	36.49
		HOMO-6 → LUMO+2	27.44
		HOMO-6 → LUMO+1	17.11
		HOMO-5 → LUMO+1	7.51

Table S22. Calculated transitions (with a contribution greater than 5%) for [Re(CO)$_3$(Q)HPhpz] (**4**)

Wavelength [nm]	Oscillator strength	Main components	%
402.89	0.0911	HOMO → LUMO	96.64
344.74	0.0574	HOMO-1 → LUMO	93.91
330.15	0.0011	HOMO-2 → LUMO	97.08
319.23	0.0136	HOMO → LUMO+1	91.70
310.94	0.0028	HOMO-3 → LUMO	92.12
302.60	0.0009	HOMO → LUMO+3	55.96
		HOMO → LUMO+2	15.15
		HOMO-1 → LUMO+3	7.79
299.70	0.0232	HOMO → LUMO+2	63.00
		HOMO-5 → LUMO	15.65
		HOMO → LUMO+3	15.39
286.28	0.2524	HOMO-1 → LUMO+1	65.22
		HOMO-1 → LUMO+3	8.12
		HOMO → LUMO+5	7.19
284.07	0.0214	HOMO-1 → LUMO+3	54.39
		HOMO-1 → LUMO+1	11.42
		HOMO-2 → LUMO+3	7.42
		HOMO-1 → LUMO+5	5.48
274.80	0.1187	HOMO-2 → LUMO+3	29.54
		HOMO-1 → LUMO+5	17.46
		HOMO-1 → LUMO+1	16.27
		HOMO → LUMO+5	14.82
		HOMO-3 → LUMO+5	5.90
270.75	0.0243	HOMO-2 → LUMO+1	32.84
		HOMO → LUMO+5	29.02
		HOMO-1 → LUMO+3	8.36
		HOMO-2 → LUMO+3	5.96
		HOMO → LUMO+3	4.90
269.61	0.0080	HOMO-2 → LUMO+1	46.02
		HOMO-4 → LUMO	14.43
		HOMO-2 → LUMO+5	13.48
		HOMO-2 → LUMO+3	8.46
		HOMO → LUMO+5	7.69
267.69	0.0250	HOMO-4 → LUMO	72.06
		HOMO-2 → LUMO+3	6.84
261.22	0.0242	HOMO-1 → LUMO+5	24.50
		HOMO-1 → LUMO+2	18.88
		HOMO-2 → LUMO+5	15.56
		HOMO → LUMO+5	9.68
		HOMO-2 → LUMO+3	6.20
259.26	0.0144	HOMO-1 → LUMO+2	56.22
		HOMO-3 → LUMO+1	10.63
		HOMO-1 → LUMO+5	5.38

257.80	0.0005	HOMO → LUMO+4	82.38
		HOMO-1 → LUMO+2	5.08
256.91	0.0548	HOMO-3 → LUMO+1	63.18
		HOMO-2 → LUMO+5	6.62
		HOMO → LUMO+4	6.54
250.86	0.1115	HOMO-5 → LUMO	34.27
		HOMO-3 → LUMO+2	18.32
		HOMO → LUMO+2	9.36
		HOMO-1 → LUMO+2	7.46
		HOMO-2 → LUMO+5	6.26
249.56	0.0843	HOMO-2 → LUMO+5	25.64
		HOMO-3 → LUMO+3	11.68
		HOMO-1 → LUMO+5	10.33
		HOMO-3 → LUMO+2	8.62
		HOMO-3 → LUMO+1	7.36
		HOMO-2 → LUMO+3	7.32
248.19	0.0045	HOMO-1 → LUMO+4	43.45
		HOMO-6 → LUMO+1	28.54
		HOMO-4 → LUMO+4	10.33
247.39	0.0264	HOMO-3 → LUMO+3	49.24
		HOMO-2 → LUMO+5	8.49
245.51	0.0086	HOMO-2 → LUMO+2	85.22
238.89	0.0153	HOMO-6 → LUMO	35.29
		HOMO → LUMO+6	22.18
		HOMO-4 → LUMO+1	8.88
		HOMO → LUMO+7	7.37
		HOMO → LUMO+8	5.95
238.76	0.0124	HOMO-6 → LUMO	55.02
		HOMO → LUMO+6	15.27
		HOMO-4 → LUMO+1	5.76
		HOMO → LUMO+7	5.45
235.20	0.1166	HOMO-4 → LUMO+1	67.52
		HOMO → LUMO+6	6.82
234.29	0.1614	HOMO-3 → LUMO+2	28.29
		HOMO → LUMO+7	20.04
		HOMO-3 → LUMO+5	16.32
		HOMO-5 → LUMO	11.76
		HOMO → LUMO+6	5.75
232.21	0.1055	HOMO-3 → LUMO+5	35.13
		HOMO → LUMO+8	14.97
		HOMO-3 → LUMO+2	14.69
		HOMO-4 → LUMO+1	8.26
230.84	0.0064	HOMO → LUMO+6	27.70
		HOMO → LUMO+7	14.97
		HOMO-7 → LUMO	9.65
		HOMO-3 → LUMO+5	9.49

		HOMO → LUMO+8	7.54
230.70	0.0094	HOMO-7 → LUMO	76.23
		HOMO-8 → LUMO	5.83
227.58	0.0361	HOMO-1 → LUMO+4	35.19
		HOMO-6 → LUMO+1	19.53
		HOMO-1 → LUMO+6	8.28
		HOMO-4 → LUMO+4	5.64
		HOMO → LUMO+7	5.56
225.80	0.1771	HOMO-8 → LUMO	25.57
		HOMO → LUMO+8	14.85
		HOMO → LUMO+7	9.17
		HOMO-3 → LUMO+2	7.11
		HOMO-1 → LUMO+4	6.94
225.25	0.1112	HOMO-8 → LUMO	53.54
		HOMO → LUMO+7	8.04
		HOMO → LUMO+8	7.75
223.36	0.0134	HOMO-1 → LUMO+6	25.58
		HOMO-1 → LUMO+9	18.61
		HOMO-1 → LUMO+8	8.56
		HOMO-2 → LUMO+7	6.06
		HOMO-2 → LUMO+6	5.83
220.53	0.0316	HOMO-2 → LUMO+4	30.66
		HOMO → LUMO+9	18.99
		HOMO-2 → LUMO+7	8.22
		HOMO-2 → LUMO+8	6.05
220.05	0.0151	HOMO-2 → LUMO+4	48.84
		HOMO → LUMO+9	16.01
		HOMO → LUMO+11	10.29
218.92	0.0343	HOMO-1 → LUMO+7	16.42
		HOMO-2 → LUMO+4	12.84
		HOMO → LUMO+11	10.55
		HOMO-1 → LUMO+8	7.57
		HOMO-2 → LUMO+9	7.35
		HOMO → LUMO+8	5.95
217.41	0.0006	HOMO-5 → LUMO+1	90.56
		HOMO-6 → LUMO+1	6.68
216.24	0.0100	HOMO-4 → LUMO+3	27.71
		HOMO-4 → LUMO+2	17.92
		HOMO-2 → LUMO+7	14.92
		HOMO-2 → LUMO+12	6.87
215.12	0.0280	HOMO-4 → LUMO+2	26.00
		HOMO-2 → LUMO+7	14.46
		HOMO-4 → LUMO+3	10.45
		HOMO-2 → LUMO+12	7.22
		HOMO → LUMO+9	5.92
214.32	0.0072	HOMO-4 → LUMO+2	11.00

		HOMO-2 → LUMO+6	10.12
		HOMO-1 → LUMO+9	8.82
		HOMO-1 → LUMO+7	8.79
		HOMO-1 → LUMO+8	8.09
		HOMO-7 → LUMO+1	6.52
		HOMO-1 → LUMO+11	6.16
213.51	0.0047	HOMO-4 → LUMO+3	38.47
		HOMO-4 → LUMO+2	32.47
212.86	0.0199	HOMO-3 → LUMO+4	77.02
		HOMO-6 → LUMO+1	6.80
212.52	0.0045	HOMO-7 → LUMO+1	17.51
		HOMO-11 → LUMO	13.84
		HOMO-9 → LUMO	12.24
		HOMO-1 → LUMO+9	7.90
		HOMO-1 → LUMO+11	7.85
		HOMO-2 → LUMO+6	5.77
212.01	0.0025	HOMO-11 → LUMO	40.53
		HOMO-9 → LUMO	13.72
		HOMO-7 → LUMO+1	12.29
		HOMO-1 → LUMO+9	6.85
210.81	0.0153	HOMO → LUMO+9	12.34
		HOMO-2 → LUMO+8	11.10
		HOMO-1 → LUMO+11	9.88
		HOMO-2 → LUMO+9	8.98
		HOMO → LUMO+12	7.27
		HOMO-2 → LUMO+6	6.81
		HOMO-2 → LUMO+11	6.74
210.44	0.0315	HOMO-1 → LUMO+7	28.60
		HOMO-2 → LUMO+6	8.48
		HOMO → LUMO+11	8.00
		HOMO-2 → LUMO+11	7.64
		HOMO → LUMO+12	6.58
		HOMO-3 → LUMO+6	5.68
209.33	0.0188	HOMO-9 → LUMO	25.13
		HOMO-10 → LUMO	22.02
		HOMO-5 → LUMO+2	17.70
		HOMO-11 → LUMO	10.85
		HOMO-1 → LUMO+11	5.43
207.12	0.0094	HOMO-7 → LUMO+1	15.94
		HOMO-1 → LUMO+11	13.47
		HOMO-1 → LUMO+9	10.60
		HOMO-1 → LUMO+6	6.82
		HOMO → LUMO+12	6.62
		HOMO-2 → LUMO+6	5.03
206.46	0.0798	HOMO-7 → LUMO+1	18.78
		HOMO → LUMO+11	10.15

		HOMO-1 → LUMO+6	9.52
		HOMO-3 → LUMO+7	7.75
		HOMO-2 → LUMO+6	6.59
		HOMO-4 → LUMO+5	5.20
204.55	0.0066	HOMO → LUMO+10	76.59
204.23	0.0031	HOMO-4 → LUMO+5	48.43
		HOMO-3 → LUMO+6	8.89
		HOMO-3 → LUMO+7	5.34
		HOMO-3 → LUMO+8	5.07
201.61	0.0110	HOMO-1 → LUMO+8	23.12
		HOMO-1 → LUMO+11	20.89
		HOMO-3 → LUMO+7	8.13
		HOMO-4 → LUMO+5	6.38
		HOMO-3 → LUMO+8	5.90
		HOMO-1 → LUMO+9	5.75
200.98	0.0071	HOMO-5 → LUMO+3	81.08
		HOMO-5 → LUMO+2	5.27
200.56	0.1215	HOMO-4 → LUMO+4	58.66
		HOMO-6 → LUMO+1	15.38
		HOMO-3 → LUMO+6	5.14

Table S23. Calculated transitions (with a contribution greater than 5%) for [Re(CO)$_3$(MeQ)Him] (**5**)

Wavelength [nm]	Oscillator strength	Main components	%
425.06	0.0730	HOMO → LUMO	98.25
333.24	0.0384	HOMO-1 → LUMO	82.39
		HOMO-2 → LUMO	13.83
326.96	0.0278	HOMO-2 → LUMO	82.51
		HOMO-1 → LUMO	13.02
307.93	0.0226	HOMO → LUMO+1	73.63
		HOMO-4 → LUMO	13.81
		HOMO → LUMO+2	8.19
307.50	0.0026	HOMO → LUMO+2	77.67
		HOMO → LUMO+1	6.71
		HOMO-3 → LUMO+2	6.37
306.34	0.0119	HOMO-3 → LUMO	92.31
289.64	0.0064	HOMO → LUMO+3	72.73
		HOMO-1 → LUMO+2	9.99
273.60	0.0109	HOMO-1 → LUMO+2	60.72
		HOMO-1 → LUMO+3	12.43
		HOMO-2 → LUMO+2	10.11
268.14	0.0143	HOMO-2 → LUMO+2	32.54
		HOMO-1 → LUMO+3	21.25
		HOMO-1 → LUMO+2	14.45
		HOMO → LUMO+3	10.10
262.72	0.1000	HOMO-4 → LUMO	34.11
		HOMO-1 → LUMO+1	17.53
		HOMO-2 → LUMO+3	13.49
		HOMO → LUMO+1	7.35
		HOMO → LUMO+4	7.01
		HOMO-2 → LUMO+2	5.90
261.31	0.0699	HOMO-2 → LUMO+3	27.09
		HOMO-1 → LUMO+3	19.03
		HOMO-4 → LUMO	18.27
		HOMO-2 → LUMO+1	15.47
258.80	0.0055	HOMO-5 → LUMO	98.16
255.67	0.0372	HOMO → LUMO+4	77.44
		HOMO-1 → LUMO+1	6.09
251.56	0.0590	HOMO-2 → LUMO+3	30.80
		HOMO-2 → LUMO+2	28.53
		HOMO-1 → LUMO+3	10.81
		HOMO-1 → LUMO+5	8.05
246.40	0.0019	HOMO-3 → LUMO+2	63.20
		HOMO → LUMO+5	11.42
		HOMO → LUMO+2	6.03
245.84	0.0499	HOMO-1 → LUMO+1	59.44
		HOMO-1 → LUMO+3	13.27

		HOMO-2 → LUMO+1	7.52
		HOMO-4 → LUMO	6.99
242.27	0.0066	HOMO → LUMO+5	39.05
		HOMO-2 → LUMO+1	17.20
		HOMO-3 → LUMO+3	11.68
		HOMO-3 → LUMO+2	9.79
240.62	0.0525	HOMO-2 → LUMO+1	48.32
		HOMO-2 → LUMO+3	10.77
		HOMO-3 → LUMO+1	9.13
		HOMO-3 → LUMO+3	7.21
235.50	0.0239	HOMO → LUMO+6	25.15
		HOMO → LUMO+7	23.02
		HOMO-3 → LUMO+1	15.16
		HOMO → LUMO+8	6.49
233.78	0.1090	HOMO → LUMO+7	30.34
		HOMO-3 → LUMO+1	22.32
		HOMO-3 → LUMO+3	9.33
232.68	0.0168	HOMO-6 → LUMO	21.65
		HOMO-3 → LUMO+3	18.80
		HOMO → LUMO+5	12.08
		HOMO → LUMO+6	11.35
		HOMO-3 → LUMO+1	8.72
232.36	0.0038	HOMO-6 → LUMO	70.85
		HOMO-3 → LUMO+3	5.85
229.79	0.1194	HOMO-1 → LUMO+4	33.83
		HOMO-3 → LUMO+1	11.12
		HOMO → LUMO+6	9.89
		HOMO → LUMO+7	7.04
		HOMO-1 → LUMO+7	6.42
		HOMO-2 → LUMO+4	5.63
227.23	0.1140	HOMO-3 → LUMO+1	16.43
		HOMO → LUMO+6	11.44
		HOMO-2 → LUMO+4	10.19
		HOMO-1 → LUMO+4	9.55
		HOMO → LUMO+7	7.79
		HOMO-3 → LUMO+3	7.19
		HOMO-1 → LUMO+5	5.45
224.25	0.0177	HOMO → LUMO+8	37.97
		HOMO-2 → LUMO+4	14.23
		HOMO → LUMO+10	11.73
222.77	0.0641	HOMO-2 → LUMO+5	29.72
		HOMO-2 → LUMO+4	14.61
		HOMO-2 → LUMO+7	8.16
		HOMO-2 → LUMO+6	5.01
218.64	0.0281	HOMO-2 → LUMO+5	14.83
		HOMO-1 → LUMO+7	10.61

		HOMO → LUMO+10	10.14
		HOMO-3 → LUMO+3	8.18
		HOMO-1 → LUMO+4	8.02
		HOMO-2 → LUMO+8	7.92
		HOMO-1 → LUMO+8	5.56
		HOMO-1 → LUMO+5	5.41
216.14	0.0314	HOMO-2 → LUMO+4	23.03
		HOMO → LUMO+10	17.15
		HOMO-1 → LUMO+4	17.09
		HOMO-1 → LUMO+5	9.16
214.85	0.0125	HOMO-1 → LUMO+7	16.82
		HOMO-8 → LUMO	14.73
		HOMO-1 → LUMO+8	13.85
		HOMO-2 → LUMO+4	5.77
213.85	0.0018	HOMO-8 → LUMO	43.72
		HOMO-9 → LUMO	11.78
		HOMO → LUMO+9	10.42
		HOMO-7 → LUMO	8.11
212.74	0.0224	HOMO → LUMO+9	38.82
		HOMO-8 → LUMO	12.70
211.54	0.0234	HOMO-7 → LUMO	47.13
		HOMO-4 → LUMO+1	23.10
		HOMO → LUMO+9	5.49
211.04	0.0170	HOMO-3 → LUMO+4	34.12
		HOMO → LUMO+9	18.70
		HOMO → LUMO+8	13.91
		HOMO → LUMO+11	6.64
209.33	0.0231	HOMO → LUMO+11	19.05
		HOMO-1 → LUMO+10	13.18
		HOMO-2 → LUMO+6	9.61
		HOMO-1 → LUMO+6	8.70
		HOMO-1 → LUMO+5	6.92
		HOMO-3 → LUMO+4	6.13
		HOMO-2 → LUMO+11	5.09
208.31	0.0219	HOMO-3 → LUMO+4	27.40
		HOMO → LUMO+10	14.34
		HOMO → LUMO+11	11.05
		HOMO-1 → LUMO+5	8.42
		HOMO → LUMO+8	6.49
		HOMO-2 → LUMO+10	6.47
		HOMO-1 → LUMO+6	5.12
208.08	0.0186	HOMO-1 → LUMO+10	12.47
		HOMO-2 → LUMO+7	10.51
		HOMO-1 → LUMO+6	10.27
		HOMO-2 → LUMO+10	10.27
		HOMO-5 → LUMO+1	6.43

		HOMO-3 → LUMO+4	5.58
		HOMO-2 → LUMO+6	5.24
206.99	0.0084	HOMO-5 → LUMO+1	83.30
204.98	0.0052	HOMO-5 → LUMO+2	88.85
		HOMO-5 → LUMO+1	3.97
204.47	0.0040	HOMO-2 → LUMO+7	25.66
		HOMO-1 → LUMO+10	24.09
		HOMO → LUMO+11	9.24
		HOMO-2 → LUMO+4	6.43
203.25	0.0018	HOMO-4 → LUMO+2	95.90
202.48	0.0165	HOMO-3 → LUMO+5	36.65
		HOMO-3 → LUMO+6	10.98
		HOMO-2 → LUMO+7	6.10
		HOMO-1 → LUMO+10	5.01
200.44	0.0216	HOMO-5 → LUMO+3	21.12
		HOMO → LUMO+10	11.37
		HOMO-1 → LUMO+8	8.85
		HOMO-1 → LUMO+7	7.15
		HOMO-1 → LUMO+5	5.68

Table S24. Calculated transitions (with a contribution greater than 5%) for [Re(CO)$_3$(MeQ)MeHim] (**6**)

Wavelength [nm]	Oscillator strength	Main components	%
426.92	0.0677	HOMO → LUMO	98.32
335.66	0.0567	HOMO-1 → LUMO	95.32
330.72	0.0006	HOMO-2 → LUMO	97.43
310.76	0.0062	HOMO → LUMO+2	66.69
		HOMO → LUMO+1	21.15
309.66	0.0222	HOMO → LUMO+1	63.00
		HOMO → LUMO+2	20.85
		HOMO-5 → LUMO	10.76
301.38	0.0280	HOMO-3 → LUMO	95.71
288.67	0.0032	HOMO → LUMO+3	62.04
		HOMO-1 → LUMO+2	9.62
		HOMO-1 → LUMO+3	9.09
		HOMO-2 → LUMO+2	5.69
275.64	0.0177	HOMO-1 → LUMO+2	49.97
		HOMO-1 → LUMO+3	11.38
		HOMO-1 → LUMO+1	11.23
		HOMO-4 → LUMO	9.68
274.97	0.0046	HOMO-4 → LUMO	83.05
		HOMO-1 → LUMO+2	8.06
270.17	0.0131	HOMO-2 → LUMO+2	47.47
		HOMO → LUMO+3	12.94
		HOMO-1 → LUMO+3	9.71
		HOMO-4 → LUMO	5.84
262.68	0.1400	HOMO-5 → LUMO	42.61
		HOMO-1 → LUMO+1	28.59
		HOMO → LUMO+3	8.62
		HOMO → LUMO+1	7.68
260.54	0.0066	HOMO-2 → LUMO+3	57.37
		HOMO-2 → LUMO+1	31.08
255.76	0.0751	HOMO-1 → LUMO+3	32.08
		HOMO-2 → LUMO+2	19.40
		HOMO-5 → LUMO	5.84
		HOMO-1 → LUMO+2	5.69
247.85	0.0270	HOMO-3 → LUMO+2	42.90
		HOMO → LUMO+5	24.55
		HOMO → LUMO+4	7.32
246.41	0.0246	HOMO → LUMO+4	67.87
		HOMO-3 → LUMO+2	9.48
		HOMO-1 → LUMO+1	5.80
244.06	0.0478	HOMO → LUMO+5	27.23
		HOMO-3 → LUMO+2	20.11
		HOMO-1 → LUMO+1	18.06
		HOMO-5 → LUMO	7.45

		HOMO-1 → LUMO+3	5.95
242.23	0.0708	HOMO-2 → LUMO+1	21.95
		HOMO-1 → LUMO+1	14.37
		HOMO-2 → LUMO+3	13.05
		HOMO → LUMO+5	12.08
		HOMO-1 → LUMO+3	9.27
		HOMO-5 → LUMO	5.47
241.48	0.0787	HOMO-2 → LUMO+1	37.70
		HOMO-2 → LUMO+3	20.54
		HOMO-1 → LUMO+1	8.66
		HOMO → LUMO+4	7.98
		HOMO-5 → LUMO	5.35
235.06	0.0202	HOMO-3 → LUMO+1	38.19
		HOMO → LUMO+7	10.67
		HOMO-3 → LUMO+3	8.10
		HOMO-6 → LUMO	7.18
		HOMO-1 → LUMO+5	7.73
		HOMO → LUMO+6	7.13
233.63	0.1290	HOMO → LUMO+7	40.50
		HOMO-3 → LUMO+3	7.96
		HOMO-3 → LUMO+1	7.72
		HOMO-2 → LUMO+5	6.62
		HOMO-1 → LUMO+7	6.48
232.98	0.0108	HOMO-6 → LUMO	82.78
228.43	0.0217	HOMO-3 → LUMO+3	24.21
		HOMO-3 → LUMO+1	20.12
		HOMO → LUMO+6	18.60
		HOMO-1 → LUMO+5	15.92
225.08	0.1752	HOMO → LUMO+6	45.43
		HOMO-3 → LUMO+1	13.74
		HOMO-3 → LUMO+3	6.30
		HOMO → LUMO+7	6.14
224.20	0.0103	HOMO → LUMO+10	27.70
		HOMO-2 → LUMO+4	24.06
		HOMO-2 → LUMO+5	5.91
		HOMO → LUMO+6	5.69
		HOMO-1 → LUMO+5	5.09
223.75	0.0121	HOMO-2 → LUMO+4	21.76
		HOMO → LUMO+10	18.54
		HOMO-2 → LUMO+7	17.66
		HOMO-1 → LUMO+4	13.14
		HOMO-1 → LUMO+7	7.33
221.77	0.0106	HOMO-2 → LUMO+5	28.04
		HOMO → LUMO+10	12.46
		HOMO-1 → LUMO+7	10.19
		HOMO-1 → LUMO+4	8.75

		HOMO → LUMO+7	5.52
218.94	0.0042	HOMO → LUMO+8	41.39
		HOMO-2 → LUMO+4	13.24
		HOMO-4 → LUMO+1	5.87
		HOMO-2 → LUMO+7	5.28
218.05	0.0094	HOMO-4 → LUMO+1	71.10
		HOMO-4 → LUMO+2	13.99
217.24	0.0116	HOMO-7 → LUMO	33.08
		HOMO-8 → LUMO	18.53
		HOMO-4 → LUMO+2	15.48
		HOMO-9 → LUMO	8.67
		HOMO → LUMO+8	5.47
216.65	0.0393	HOMO-1 → LUMO+4	36.94
		HOMO-1 → LUMO+7	12.20
		HOMO-2 → LUMO+7	7.97
		HOMO-3 → LUMO+3	7.31
		HOMO-4 → LUMO+2	7.30
		HOMO-1 → LUMO+8	5.26
216.58	0.0110	HOMO-4 → LUMO+2	49.21
		HOMO-4 → LUMO+1	15.41
		HOMO-7 → LUMO	9.18
		HOMO-8 → LUMO	7.18
213.55	0.0307	HOMO → LUMO+9	36.80
		HOMO-1 → LUMO+7	10.25
		HOMO → LUMO+8	10.13
		HOMO-2 → LUMO+7	7.50
		HOMO-4 → LUMO+2	5.75
212.54	0.0220	HOMO → LUMO+9	29.65
		HOMO-2 → LUMO+7	26.09
		HOMO-1 → LUMO+7	6.17
212.07	0.0321	HOMO → LUMO+9	15.59
		HOMO-1 → LUMO+10	14.38
		HOMO-3 → LUMO+5	8.88
		HOMO-1 → LUMO+5	6.85
211.42	0.0161	HOMO-8 → LUMO	28.38
		HOMO-5 → LUMO+1	17.03
		HOMO-7 → LUMO	13.48
		HOMO-9 → LUMO	7.94
		HOMO → LUMO+8	7.87
210.00	0.0194	HOMO → LUMO+11	11.23
		HOMO-8 → LUMO	9.41
		HOMO-2 → LUMO+5	8.85
		HOMO → LUMO+8	8.38
		HOMO-2 → LUMO+4	7.99
		HOMO-7 → LUMO	6.80
		HOMO-3 → LUMO+4	5.81

		HOMO-1 → LUMO+7	5.47
205.60	0.0013	HOMO-4 → LUMO+3	83.45
204.98	0.0042	HOMO-5 → LUMO+2	95.36
204.01	0.0105	HOMO-3 → LUMO+5	38.92
		HOMO-1 → LUMO+5	11.46
		HOMO-3 → LUMO+4	8.51
		HOMO-2 → LUMO+10	8.46
		HOMO-1 → LUMO+6	6.53
203.30	0.0028	HOMO-1 → LUMO+10	36.75
		HOMO-3 → LUMO+5	17.41
200.95	0.0347	HOMO → LUMO+11	34.10
		HOMO-3 → LUMO+4	22.00
		HOMO-1 → LUMO+6	8.17
		HOMO-1 → LUMO+5	5.22

Table S25. Calculated transitions (with a contribution greater than 5%) for [Re(CO)$_3$(MeQ)Hdmpz] (**7**)

Wavelength [nm]	Oscillator strength	Main components	%
405.64	0.0772	HOMO → LUMO	97.64
339.30	0.0609	HOMO-1 → LUMO	95.39
325.27	0.0029	HOMO-2 → LUMO	96.04
306.67	0.0010	HOMO-3 → LUMO	91.51
302.42	0.0045	HOMO → LUMO+1	41.51
		HOMO → LUMO+2	36.61
		HOMO-4 → LUMO	6.53
299.91	0.0102	HOMO → LUMO+2	45.24
		HOMO → LUMO+1	32.64
		HOMO-4 → LUMO	14.30
286.17	0.0010	HOMO → LUMO+3	51.83
		HOMO-1 → LUMO+2	32.48
278.22	0.0077	HOMO-1 → LUMO+3	37.13
		HOMO-1 → LUMO+2	22.84
		HOMO-1 → LUMO+1	12.11
		HOMO → LUMO+3	11.10
		HOMO-2 → LUMO+2	6.94
270.07	0.0276	HOMO-2 → LUMO+2	27.76
		HOMO → LUMO+3	21.82
		HOMO-1 → LUMO+2	18.30
		HOMO-1 → LUMO+3	7.52
		HOMO-1 → LUMO+4	5.01
261.88	0.0647	HOMO-2 → LUMO+3	20.04
		HOMO-4 → LUMO	18.52
		HOMO-1 → LUMO+1	16.25
		HOMO-2 → LUMO+1	5.57
		HOMO-2 → LUMO+2	5.02
256.56	0.1192	HOMO-4 → LUMO	26.88
		HOMO-2 → LUMO+3	24.32
		HOMO-2 → LUMO+1	11.40
		HOMO-1 → LUMO+3	10.39
		HOMO → LUMO+1	8.51
253.97	0.0762	HOMO-2 → LUMO+2	36.11
		HOMO-2 → LUMO+3	20.81
		HOMO-1 → LUMO+3	13.67
253.01	0.0070	HOMO → LUMO+4	47.92
		HOMO-1 → LUMO+1	32.08
252.26	0.0045	HOMO-5 → LUMO	86.70
		HOMO → LUMO+4	5.14
249.50	0.0852	HOMO-1 → LUMO+1	28.96
		HOMO → LUMO+4	16.04
		HOMO-4 → LUMO	11.61
		HOMO-3 → LUMO+2	9.20

		HOMO-1 → LUMO+3	8.38
		HOMO-3 → LUMO+1	6.72
		HOMO-5 → LUMO	5.56
245.73	0.0207	HOMO-3 → LUMO+2	50.31
		HOMO-6 → LUMO	16.49
		HOMO → LUMO+4	11.06
243.65	0.0445	HOMO-6 → LUMO	72.76
		HOMO-3 → LUMO+2	9.39
		HOMO → LUMO+4	5.45
240.39	0.0201	HOMO-4 → LUMO+1	68.19
		HOMO-2 → LUMO+3	17.55
238.20	0.0095	HOMO-3 → LUMO+3	27.94
		HOMO → LUMO+5	24.05
		HOMO → LUMO+6	16.22
		HOMO-3 → LUMO+1	8.41
235.64	0.0256	HOMO-1 → LUMO+4	52.02
		HOMO-3 → LUMO+3	11.28
		HOMO → LUMO+6	6.87
		HOMO-1 → LUMO+7	6.81
232.96	0.1081	HOMO-3 → LUMO+1	37.03
		HOMO → LUMO+6	18.95
		HOMO-1 → LUMO+4	8.55
230.92	0.0664	HOMO → LUMO+5	28.10
		HOMO-3 → LUMO+3	19.26
		HOMO-3 → LUMO+1	11.51
		HOMO-2 → LUMO+4	7.38
		HOMO → LUMO+6	5.25
227.73	0.0990	HOMO-2 → LUMO+4	22.24
		HOMO → LUMO+5	12.79
		HOMO → LUMO+7	10.60
		HOMO-3 → LUMO+1	8.32
		HOMO-2 → LUMO+7	7.52
		HOMO → LUMO+6	7.43
225.97	0.0275	HOMO-7 → LUMO	87.73
224.16	0.1961	HOMO-2 → LUMO+4	19.06
		HOMO → LUMO+6	16.80
		HOMO-3 → LUMO+1	9.94
		HOMO-3 → LUMO+3	9.14
		HOMO → LUMO+5	7.99
222.90	0.0266	HOMO → LUMO+7	43.56
		HOMO → LUMO+9	13.84
		HOMO-2 → LUMO+4	7.46
		HOMO-3 → LUMO+7	5.38
218.34	0.0253	HOMO-2 → LUMO+5	12.69
		HOMO → LUMO+9	11.29
		HOMO-1 → LUMO+5	10.21

		HOMO-1 → LUMO+6	9.72
		HOMO → LUMO+7	8.46
		HOMO-1 → LUMO+7	5.42
216.11	0.0532	HOMO-1 → LUMO+7	13.50
		HOMO → LUMO+10	13.05
		HOMO → LUMO+9	12.20
		HOMO-3 → LUMO+4	11.06
		HOMO → LUMO+8	10.93
215.21	0.0295	HOMO-2 → LUMO+5	22.42
		HOMO → LUMO+10	9.78
		HOMO-2 → LUMO+10	8.68
		HOMO-2 → LUMO+4	8.25
		HOMO → LUMO+9	7.25
213.06	0.0165	HOMO-1 → LUMO+7	47.86
		HOMO-1 → LUMO+5	7.54
		HOMO-1 → LUMO+8	6.54
		HOMO-9 → LUMO	5.37
		HOMO-1 → LUMO+4	5.11
211.75	0.0031	HOMO-9 → LUMO	88.98
210.91	0.0171	HOMO-3 → LUMO+4	25.38
		HOMO-1 → LUMO+5	12.42
		HOMO-8 → LUMO	9.92
		HOMO-4 → LUMO+1	9.42
		HOMO-1 → LUMO+9	8.94
210.59	0.0409	HOMO-8 → LUMO	31.21
		HOMO-1 → LUMO+9	19.99
		HOMO-4 → LUMO+1	14.74
		HOMO-1 → LUMO+6	7.62
		HOMO-1 → LUMO+5	7.22
		HOMO-1 → LUMO+10	5.22
210.20	0.0408	HOMO-3 → LUMO+4	31.49
		HOMO → LUMO+8	14.92
		HOMO-8 → LUMO	10.14
		HOMO-1 → LUMO+5	8.86
		HOMO-4 → LUMO+1	8.16
208.12	0.0112	HOMO → LUMO+8	46.71
		HOMO → LUMO+10	12.19
		HOMO-2 → LUMO+7	9.41
		HOMO → LUMO+7	7.14
		HOMO-2 → LUMO+6	5.07
207.76	0.0280	HOMO-1 → LUMO+9	12.96
		HOMO → LUMO+9	11.54
		HOMO → LUMO+8	11.31
		HOMO-2 → LUMO+7	9.60
		HOMO-3 → LUMO+4	9.17
		HOMO-2 → LUMO+9	8.93

		HOMO-1 → LUMO+10	7.01
		HOMO-2 → LUMO+6	5.16
205.30	0.0081	HOMO-4 → LUMO+2	85.26
203.96	0.0041	HOMO-5 → LUMO+1	60.00
		HOMO-5 → LUMO+2	24.15
		HOMO-4 → LUMO+2	6.00
202.72	0.0092	HOMO-1 → LUMO+9	18.78
		HOMO-3 → LUMO+5	13.06
		HOMO-1 → LUMO+6	12.54
		HOMO-3 → LUMO+6	12.47
		HOMO-2 → LUMO+7	6.83
		HOMO-5 → LUMO+1	6.21
201.88	0.0210	HOMO-6 → LUMO+2	18.39
		HOMO-2 → LUMO+7	14.66
		HOMO-6 → LUMO+1	7.74
		HOMO-5 → LUMO+2	7.10
		HOMO-5 → LUMO+1	6.49
		HOMO-2 → LUMO+9	5.80
201.81	0.0037	HOMO-5 → LUMO+2	54.84
		HOMO-5 → LUMO+1	14.77
		HOMO-3 → LUMO+5	5.64
201.06	0.0650	HOMO-5 → LUMO+1	41.94
		HOMO-5 → LUMO	7.05
		HOMO-2 → LUMO+7	6.54
		HOMO-1 → LUMO+6	5.40
200.33	0.0147	HOMO-4 → LUMO+3	75.73
		HOMO-8 → LUMO	5.75
		HOMO-4 → LUMO+1	5.05

Table S26. Calculated transitions (with a contribution greater than 5%) for [Re$(CO)_3$(MeQ)HPhpz] (**8**)

Wavelength [nm]	Oscillator strength	Main components	%
404.73	0.0719	HOMO → LUMO	97.55
340.37	0.0559	HOMO-1 → LUMO	94.21
325.81	0.0017	HOMO-2 → LUMO	94.25
324.62	0.0026	HOMO → LUMO+1	92.16
306.40	0.0038	HOMO-3 → LUMO	88.80
301.82	0.0072	HOMO → LUMO+2	69.83
		HOMO-5 → LUMO	15.02
296.03	0.0143	HOMO → LUMO+3	62.88
		HOMO-5 → LUMO	6.41
287.04	0.2086	HOMO-1 → LUMO+1	55.54
		HOMO → LUMO+5	13.43
		HOMO → LUMO+3	8.14
		HOMO-1 → LUMO+3	7.38
280.28	0.0218	HOMO-1 → LUMO+3	45.00
		HOMO-1 → LUMO+2	17.69
		HOMO-1 → LUMO+1	8.55
		HOMO-1 → LUMO+5	7.54
277.09	0.1666	HOMO → LUMO+5	44.48
		HOMO-1 → LUMO+1	22.35
		HOMO-1 → LUMO+5	8.33
271.09	0.0128	HOMO-2 → LUMO+1	45.93
		HOMO-2 → LUMO+3	20.36
		HOMO-1 → LUMO+3	7.50
267.73	0.0021	HOMO-2 → LUMO+1	33.18
		HOMO-2 → LUMO+5	14.14
		HOMO-4 → LUMO	13.09
		HOMO-2 → LUMO+3	12.17
		HOMO-2 → LUMO+2	10.54
265.55	0.0401	HOMO-4 → LUMO	73.85
261.25	0.0009	HOMO → LUMO+4	88.22
260.03	0.0406	HOMO-1 → LUMO+2	24.54
		HOMO-1 → LUMO+3	10.49
		HOMO → LUMO+5	10.18
		HOMO-1 → LUMO+5	9.83
		HOMO-2 → LUMO+5	7.43
		HOMO-5 → LUMO	6.20
258.15	0.0792	HOMO-5 → LUMO	27.07
		HOMO-3 → LUMO+1	16.20
		HOMO-1 → LUMO+5	10.57
		HOMO → LUMO+2	7.16
		HOMO-2 → LUMO+2	7.09
		HOMO-2 → LUMO+5	6.92
256.57	0.1165	HOMO-3 → LUMO+1	64.90

		HOMO → LUMO+5	7.10
		HOMO-5 → LUMO	5.95
249.99	0.1120	HOMO-1 → LUMO+2	38.00
		HOMO-5 → LUMO	14.92
		HOMO-2 → LUMO+5	7.83
		HOMO-3 → LUMO+2	7.78
		HOMO-1 → LUMO+3	5.76
248.80	0.0099	HOMO-1 → LUMO+4	46.93
		HOMO-6 → LUMO+1	28.87
		HOMO-4 → LUMO+4	10.18
247.66	0.0132	HOMO-2 → LUMO+5	21.70
		HOMO-2 → LUMO+3	21.61
		HOMO-1 → LUMO+5	20.16
		HOMO-2 → LUMO+1	5.80
		HOMO-1 → LUMO+7	5.75
244.14	0.0308	HOMO-3 → LUMO+3	46.59
		HOMO-3 → LUMO+2	10.11
		HOMO → LUMO+7	7.16
239.54	0.0078	HOMO-2 → LUMO+2	63.90
		HOMO-2 → LUMO+5	21.03
		HOMO-2 → LUMO+3	8.99
238.23	0.0481	HOMO → LUMO+6	38.92
		HOMO-4 → LUMO+1	23.07
		HOMO → LUMO+7	8.45
		HOMO → LUMO+8	7.08
237.12	0.0005	HOMO-6 → LUMO	96.64
235.71	0.1517	HOMO → LUMO+6	20.71
		HOMO-3 → LUMO+5	17.14
		HOMO → LUMO+7	14.93
		HOMO-3 → LUMO+2	13.11
		HOMO-4 → LUMO+1	12.30
233.64	0.0789	HOMO-4 → LUMO+1	52.58
		HOMO-3 → LUMO+3	13.42
		HOMO-3 → LUMO+5	6.07
232.98	0.0603	HOMO → LUMO+8	22.91
		HOMO-3 → LUMO+2	22.00
		HOMO → LUMO+6	16.19
		HOMO → LUMO+7	5.55
229.94	0.0279	HOMO-3 → LUMO+5	27.70
		HOMO → LUMO+7	16.32
		HOMO → LUMO+8	11.35
		HOMO-3 → LUMO+2	10.71
229.42	0.0010	HOMO-7 → LUMO	70.94
		HOMO-8 → LUMO	17.70
227.98	0.0489	HOMO-1 → LUMO+4	22.95
		HOMO-6 → LUMO+1	18.53

		HOMO-1 → LUMO+6	10.49
		HOMO → LUMO+7	9.35
226.04	0.2296	HOMO-1 → LUMO+4	12.43
		HOMO-3 → LUMO+2	12.35
		HOMO → LUMO+8	10.40
		HOMO-6 → LUMO+1	8.73
		HOMO → LUMO+7	8.72
		HOMO → LUMO+9	6.72
		HOMO-3 → LUMO+5	5.50
225.14	0.0123	HOMO-8 → LUMO	67.20
		HOMO-7 → LUMO	17.80
		HOMO-5 → LUMO+1	5.27
224.91	0.0068	HOMO-5 → LUMO+1	91.07
223.26	0.0147	HOMO-1 → LUMO+6	24.52
		HOMO-1 → LUMO+9	15.53
		HOMO-1 → LUMO+4	6.92
		HOMO-2 → LUMO+6	6.21
		HOMO-2 → LUMO+7	6.16
		HOMO-6 → LUMO+1	5.00
222.21	0.0446	HOMO → LUMO+9	38.33
		HOMO → LUMO+11	10.57
		HOMO → LUMO+8	8.48
		HOMO-2 → LUMO+9	5.79
220.34	0.0239	HOMO-2 → LUMO+4	60.12
		HOMO → LUMO+11	7.03
		HOMO-2 → LUMO+3	5.18
218.83	0.0232	HOMO-2 → LUMO+4	24.03
		HOMO-1 → LUMO+7	13.83
		HOMO → LUMO+11	11.70
		HOMO-2 → LUMO+9	8.60
		HOMO-1 → LUMO+8	6.71
		HOMO-3 → LUMO+5	5.17
215.94	0.0209	HOMO-2 → LUMO+7	30.25
		HOMO-2 → LUMO+12	15.28
214.16	0.0051	HOMO-4 → LUMO+2	28.46
		HOMO-11 → LUMO	22.21
		HOMO-9 → LUMO	17.18
		HOMO-4 → LUMO+3	7.84
		HOMO-5 → LUMO+2	6.31
213.81	0.0056	HOMO → LUMO+12	13.73
		HOMO-1 → LUMO+9	12.49
		HOMO-7 → LUMO+1	11.89
		HOMO-2 → LUMO+6	6.27
		HOMO-3 → LUMO+6	5.63
213.40	0.0024	HOMO-4 → LUMO+2	32.85
		HOMO-11 → LUMO	27.86

		HOMO-9 → LUMO	7.89
		HOMO-4 → LUMO+3	6.57
212.87	0.0136	HOMO-3 → LUMO+4	67.14
212.68	0.0095	HOMO-1 → LUMO+9	19.08
		HOMO-7 → LUMO+1	16.18
		HOMO-3 → LUMO+4	9.44
		HOMO-1 → LUMO+11	7.17
		HOMO-11 → LUMO	6.80
		HOMO-1 → LUMO+6	6.79
211.90	0.0064	HOMO → LUMO+9	11.47
		HOMO-2 → LUMO+6	9.25
		HOMO → LUMO+10	8.32
		HOMO → LUMO+11	7.45
		HOMO-1 → LUMO+9	6.74
		HOMO-2 → LUMO+9	6.41
210.74	0.0427	HOMO-5 → LUMO+2	26.21
		HOMO-9 → LUMO	23.55
		HOMO-10 → LUMO	15.39
		HOMO-11 → LUMO	8.03
		HOMO-1 → LUMO+11	6.07
209.61	0.0098	HOMO-4 → LUMO+3	45.48
		HOMO-4 → LUMO+2	8.10
		HOMO-2 → LUMO+6	5.11
209.14	0.0311	HOMO-1 → LUMO+7	22.29
		HOMO-4 → LUMO+3	13.14
		HOMO-1 → LUMO+11	11.00
		HOMO-1 → LUMO+8	10.83
		HOMO-2 → LUMO+11	8.22
		HOMO-4 → LUMO+2	7.27
		HOMO-2 → LUMO+8	5.05
208.06	0.0148	HOMO → LUMO+10	37.70
		HOMO-7 → LUMO+1	21.77
		HOMO-1 → LUMO+11	9.32
207.35	0.0101	HOMO → LUMO+10	27.76
		HOMO → LUMO+12	14.41
		HOMO-2 → LUMO+9	5.98
		HOMO-2 → LUMO+12	5.24
		HOMO-2 → LUMO+8	5.28
		HOMO-1 → LUMO+11	5.09
206.48	0.0853	HOMO-7 → LUMO+1	21.67
		HOMO → LUMO+11	11.70
		HOMO-1 → LUMO+6	9.73
		HOMO-1 → LUMO+11	6.03
		HOMO → LUMO+10	5.25
204.11	0.0098	HOMO-5 → LUMO+3	74.48
		HOMO-5 → LUMO+2	8.95

		HOMO-9 → LUMO	5.72
203.18	0.0088	HOMO-4 → LUMO+5	22.42
		HOMO-3 → LUMO+7	15.51
		HOMO-3 → LUMO+6	13.12
		HOMO-3 → LUMO+8	11.57
201.67	0.0061	HOMO-4 → LUMO+5	25.05
		HOMO-1 → LUMO+11	18.47
		HOMO-1 → LUMO+8	17.70
200.89	0.0699	HOMO-4 → LUMO+4	45.31
		HOMO-3 → LUMO+6	17.05
		HOMO-6 → LUMO+1	12.72
		HOMO → LUMO+11	5.18
200.09	0.1348	HOMO-3 → LUMO+6	20.76
		HOMO-4 → LUMO+4	17.24
		HOMO-4 → LUMO+5	10.55
		HOMO-6 → LUMO+1	7.81
		HOMO-2 → LUMO+11	5.08

Table S27. Calculated transitions (with a contribution greater than 5%) for [Re(CO)$_3$(ClQ)Him] (**9**)

Wavelength [nm]	Oscillator strength	Main components	%
439.35	0.1025	HOMO → LUMO	98.03
347.72	0.0438	HOMO-1 → LUMO	82.32
		HOMO-2 → LUMO	14.10
342.07	0.0303	HOMO-2 → LUMO	83.57
		HOMO-1 → LUMO	13.15
321.59	0.0128	HOMO-3 → LUMO	96.70
318.23	0.0469	HOMO → LUMO+1	87.57
		HOMO-5 → LUMO	9.58
308.53	0.0008	HOMO → LUMO+2	83.18
		HOMO-3 → LUMO+2	9.12
285.97	0.0078	HOMO → LUMO+3	56.59
		HOMO-1 → LUMO+2	30.28
		HOMO-3 → LUMO+3	5.44
276.90	0.0136	HOMO-1 → LUMO+2	37.19
		HOMO-2 → LUMO+2	26.12
		HOMO-1 → LUMO+3	18.41
		HOMO → LUMO+3	8.58
270.26	0.0191	HOMO-2 → LUMO+2	23.22
		HOMO → LUMO+3	19.03
		HOMO-1 → LUMO+2	18.34
		HOMO-4 → LUMO	12.51
		HOMO-1 → LUMO+3	10.91
269.58	0.0072	HOMO-4 → LUMO	86.85
264.48	0.0143	HOMO-1 → LUMO+1	74.76
		HOMO-5 → LUMO	13.40
262.55	0.0133	HOMO-2 → LUMO+3	44.55
		HOMO-1 → LUMO+3	33.92
		HOMO-2 → LUMO+2	5.93
258.13	0.0098	HOMO-2 → LUMO+1	66.80
		HOMO-1 → LUMO+1	14.71
		HOMO-5 → LUMO	7.65
253.46	0.0157	HOMO → LUMO+4	40.58
		HOMO-2 → LUMO+1	10.84
		HOMO-2 → LUMO+3	7.57
		HOMO-3 → LUMO+2	6.74
		HOMO-1 → LUMO+3	6.39
		HOMO-2 → LUMO+2	5.13
253.07	0.0225	HOMO-2 → LUMO+3	28.51
		HOMO-2 → LUMO+2	16.09
		HOMO-1 → LUMO+3	8.92
		HOMO → LUMO+4	7.56
		HOMO-5 → LUMO	5.55
		HOMO-3 → LUMO+1	5.34
251.38	0.0166	HOMO-3 → LUMO+2	38.58
		HOMO-3 → LUMO+1	28.42
		HOMO-5 → LUMO	8.68

		HOMO → LUMO+2	6.19
		HOMO-2 → LUMO+1	5.90
248.14	0.0312	HOMO → LUMO+4	37.02
		HOMO-3 → LUMO+2	28.42
		HOMO-3 → LUMO+1	15.30
242.01	0.1306	HOMO → LUMO+5	36.11
		HOMO-5 → LUMO	25.67
		HOMO-3 → LUMO+1	20.89
239.48	0.1074	HOMO → LUMO+6	26.89
		HOMO-3 → LUMO+3	22.50
		HOMO-5 → LUMO	10.05
		HOMO-3 → LUMO+1	8.27
		HOMO → LUMO+5	7.60
		HOMO-3 → LUMO+6	5.64
235.30	0.0194	HOMO-3 → LUMO+3	34.62
		HOMO-1 → LUMO+4	10.36
		HOMO → LUMO+6	6.63
		HOMO → LUMO+5	6.33
		HOMO-1 → LUMO+6	5.10
233.26	0.0049	HOMO-6 → LUMO	90.42
232.54	0.3433	HOMO → LUMO+5	25.21
		HOMO → LUMO+6	22.08
		HOMO → LUMO+7	9.25
		HOMO-5 → LUMO	7.17
		HOMO-1 → LUMO+4	6.60
		HOMO-3 → LUMO+1	6.31
229.64	0.0207	HOMO → LUMO+7	51.19
		HOMO → LUMO+6	14.11
		HOMO-2 → LUMO+7	5.02
227.87	0.0387	HOMO-1 → LUMO+4	38.08
		HOMO-1 → LUMO+7	14.02
		HOMO-2 → LUMO+4	11.44
224.22	0.0018	HOMO → LUMO+8	64.17
		HOMO → LUMO+9	23.97
222.57	0.0255	HOMO-2 → LUMO+5	23.55
		HOMO-2 → LUMO+4	20.71
		HOMO-2 → LUMO+7	10.01
		HOMO-2 → LUMO+6	9.98
		HOMO → LUMO+7	7.00
		HOMO-1 → LUMO+5	5.54
220.41	0.0227	HOMO → LUMO+9	32.35
		HOMO-2 → LUMO+4	10.90
		HOMO → LUMO+12	8.41
		HOMO → LUMO+11	6.55
		HOMO-3 → LUMO+8	6.25
		HOMO → LUMO+8	5.14
217.79	0.0308	HOMO-1 → LUMO+7	11.68
		HOMO → LUMO+11	9.18
		HOMO-1 → LUMO+4	8.04

		HOMO-2 → LUMO+5	7.68
		HOMO-1 → LUMO+5	7.21
		HOMO-1 → LUMO+6	5.75
		HOMO-2 → LUMO+8	5.10
216.70	0.0038	HOMO-4 → LUMO+1	95.62
216.12	0.0438	HOMO-2 → LUMO+4	18.55
		HOMO-1 → LUMO+4	8.51
		HOMO-2 → LUMO+5	8.44
		HOMO → LUMO+11	8.34
		HOMO-2 → LUMO+11	7.69
		HOMO → LUMO+7	7.17
		HOMO-1 → LUMO+6	6.16
214.06	0.0069	HOMO-1 → LUMO+7	21.91
		HOMO-1 → LUMO+4	14.45
		HOMO-1 → LUMO+5	9.24
		HOMO-1 → LUMO+8	8.46
		HOMO-1 → LUMO+9	7.94
		HOMO-7 → LUMO	5.16
212.59	0.0068	HOMO-7 → LUMO	76.82
		HOMO-5 → LUMO+1	5.39
212.20	0.0158	HOMO-3 → LUMO+4	31.19
		HOMO-11 → LUMO	9.37
		HOMO-10 → LUMO	9.18
		HOMO-8 → LUMO	8.64
211.74	0.0206	HOMO-8 → LUMO	21.72
		HOMO-11 → LUMO	20.44
		HOMO-10 → LUMO	20.27
		HOMO-3 → LUMO+4	14.93
211.36	0.0204	HOMO-1 → LUMO+5	23.90
		HOMO-1 → LUMO+11	22.63
		HOMO-1 → LUMO+6	12.78
		HOMO → LUMO+11	8.27
		HOMO-2 → LUMO+6	6.84
209.18	0.0085	HOMO-4 → LUMO+2	60.11
208.56	0.0113	HOMO-4 → LUMO+2	34.01
		HOMO-2 → LUMO+11	7.94
		HOMO-2 → LUMO+6	6.11
		HOMO-2 → LUMO+5	5.47
		HOMO-2 → LUMO+7	5.01
208.07	0.0040	HOMO → LUMO+10	62.68
		HOMO-3 → LUMO+4	12.29
205.79	0.0128	HOMO → LUMO+12	23.78
		HOMO → LUMO+10	14.49
		HOMO → LUMO+9	13.25
		HOMO-3 → LUMO+4	5.88
205.28	0.0002	HOMO-9 → LUMO	95.00
204.92	0.0558	HOMO-5 → LUMO+1	24.91
		HOMO-8 → LUMO	20.34
		HOMO-10 → LUMO	14.51

		HOMO-2 → LUMO+7	7.77
203.38	0.0079	HOMO-2 → LUMO+7	25.66
		HOMO-8 → LUMO	8.53
		HOMO-2 → LUMO+12	5.98
		HOMO-2 → LUMO+4	5.93
		HOMO-5 → LUMO+1	5.73
202.60	0.0219	HOMO-3 → LUMO+5	21.58
		HOMO-3 → LUMO+6	16.60
		HOMO-1 → LUMO+10	12.05
		HOMO → LUMO+12	11.59
		HOMO-3 → LUMO+4	6.65
201.13	0.0067	HOMO-4 → LUMO+3	50.91
		HOMO-1 → LUMO+7	8.50
		HOMO → LUMO+11	8.25

Table S28. Calculated transitions (with a contribution greater than 5%) for [Re(CO)$_3$(ClQ)MeHim] (**10**)

Wavelength [nm]	Oscillator strength	Main components	%
443.56	0.0997	HOMO → LUMO	98.11
348.27	0.0353	HOMO-1 → LUMO	81.50
		HOMO-2 → LUMO	13.85
341.98	0.0361	HOMO-2 → LUMO	82.92
		HOMO-1 → LUMO	13.35
326.24	0.0080	HOMO-3 → LUMO	94.87
319.14	0.0448	HOMO → LUMO+1	87.60
		HOMO-5 → LUMO	9.25
310.34	0.0012	HOMO → LUMO+2	83.71
		HOMO-3 → LUMO+2	6.77
287.65	0.0129	HOMO-4 → LUMO	97.68
285.89	0.0071	HOMO → LUMO+3	59.20
		HOMO-1 → LUMO+2	28.29
275.14	0.0170	HOMO-1 → LUMO+2	34.28
		HOMO-2 → LUMO+2	25.69
		HOMO-1 → LUMO+3	15.42
		HOMO → LUMO+3	11.15
		HOMO-2 → LUMO+3	7.23
270.01	0.0192	HOMO-2 → LUMO+2	25.16
		HOMO-1 → LUMO+2	23.24
		HOMO → LUMO32	17.63
		HOMO-1 → LUMO+3	15.52
263.39	0.0183	HOMO-1 → LUMO+1	65.94
		HOMO-5 → LUMO	15.05
		HOMO-2 → LUMO+1	8.81
260.80	0.0152	HOMO-2 → LUMO+3	33.88
		HOMO-1 → LUMO+3	33.14
		HOMO-2 → LUMO+1	8.64
		HOMO-3 → LUMO+3	8.57
258.45	0.0080	HOMO-2 → LUMO+1	47.39
		HOMO-1 → LUMO+1	27.66
		HOMO-5 → LUMO	9.45
252.85	0.0371	HOMO-2 → LUMO+3	31.06
		HOMO-2 → LUMO+2	21.88
		HOMO-1 → LUMO+3	10.40
		HOMO-3 → LUMO+1	7.27
		HOMO-1 → LUMO+6	5.29
252.43	0.0080	HOMO-3 → LUMO+2	67.97
		HOMO → LUMO+2	9.16
251.80	0.0167	HOMO-3 → LUMO+1	45.65
		HOMO-2 → LUMO+1	23.05
		HOMO-5 → LUMO	10.81
		HOMO-2 → LUMO+3	7.87
245.20	0.0070	HOMO → LUMO+4	70.31
		HOMO-3 → LUMO+1	7.29
242.08	0.1446	HOMO → LUMO+5	33.04

		HOMO-5 → LUMO	26.62
		HOMO-3 → LUMO+1	14.62
		HOMO → LUMO+4	6.19
239.59	0.0716	HOMO → LUMO+6	39.59
		HOMO-3 → LUMO+3	19.17
		HOMO-3 → LUMO+6	7.47
		HOMO-5 → LUMO	7.09
235.20	0.0464	HOMO-3 → LUMO+3	25.61
		HOMO-6 → LUMO	21.47
		HOMO → LUMO+5	8.91
		HOMO-1 → LUMO+4	6.24
234.72	0.0064	HOMO-6 → LUMO	72.88
		HOMO-3 → LUMO+3	9.44
233.41	0.3575	HOMO → LUMO+5	36.11
		HOMO → LUMO+6	23.68
		HOMO-5 → LUMO	8.80
		HOMO-3 → LUMO+1	8.37
227.46	0.0154	HOMO-1 → LUMO+4	41.50
		HOMO-1 → LUMO+7	9.49
		HOMO-4 → LUMO+1	7.86
		HOMO-2 → LUMO+4	5.15
227.29	0.0281	HOMO-4 → LUMO+1	88.33
225.44	0.0005	HOMO → LUMO+7	48.91
		HOMO → LUMO+4	8.88
		HOMO-2 → LUMO+6	5.04
224.76	0.0027	HOMO → LUMO+9	53.58
		HOMO → LUMO+8	31.45
221.14	0.0328	HOMO → LUMO+7	18.53
		HOMO-2 → LUMO+5	16.84
		HOMO-2 → LUMO+6	10.41
		HOMO-1 → LUMO+5	8.97
		HOMO-3 → LUMO+5	7.91
		HOMO-2 → LUMO+4	7.56
220.43	0.0082	HOMO-4 → LUMO+2	73.70
219.57	0.0205	HOMO → LUMO+10	14.80
		HOMO-4 → LUMO+2	13.96
		HOMO → LUMO+11	10.94
		HOMO-2 → LUMO+4	9.44
		HOMO → LUMO+8	7.64
		HOMO → LUMO+9	7.04
		HOMO → LUMO+12	6.30
216.80	0.0458	HOMO-2 → LUMO+4	17.33
		HOMO-2 → LUMO+5	12.44
		HOMO → LUMO+7	8.66
		HOMO → LUMO+6	7.56
		HOMO-2 → LUMO+10	7.26
		HOMO-2 → LUMO+8	6.11
		HOMO-1 → LUMO+4	5.45
215.26	0.0046	HOMO-1 → LUMO+6	16.83

		HOMO → LUMO+11	12.90
		HOMO-2 → LUMO+4	11.63
		HOMO-3 → LUMO+11	6.01
		HOMO-2 → LUMO+11	5.68
214.05	0.0063	HOMO-7 → LUMO	70.72
		HOMO-8 → LUMO	10.52
		HOMO-11 → LUMO	8.92
212.32	0.0330	HOMO-1 → LUMO+5	32.31
		HOMO-1 → LUMO+11	11.66
		HOMO → LUMO+11	10.81
		HOMO-2 → LUMO+6	9.35
211.78	0.0051	HOMO-11 → LUMO	31.41
		HOMO-8 → LUMO	26.50
		HOMO-7 → LUMO	16.21
		HOMO-10 → LUMO	11.78
210.39	0.0064	HOMO-4 → LUMO+3	66.90
209.47	0.0094	HOMO-3 → LUMO+4	34.70
		HOMO-1 → LUMO+11	8.45
		HOMO-3 → LUMO+6	5.59
		HOMO-1 → LUMO+8	5.50
		HOMO → LUMO+12	5.36
209.16	0.0022	HOMO → LUMO+8	29.67
		HOMO → LUMO+10	28.36
		HOMO → LUMO+9	23.04
207.73	0.0163	HOMO-2 → LUMO+5	13.73
		HOMO-4 → LUMO+3	11.30
		HOMO → LUMO+10	10.06
		HOMO → LUMO+12	7.56
		HOMO-3 → LUMO+6	6.10
		HOMO-1 → LUMO+11	5.60
		HOMO-3 → LUMO+5	5.46
205.72	0.0158	HOMO-3 → LUMO+4	18.54
		HOMO-1 → LUMO+8	9.42
		HOMO-1 → LUMO+11	8.52
		HOMO-1 → LUMO+10	8.39
		HOMO-2 → LUMO+11	8.18
		HOMO → LUMO+12	5.48
205.64	0.0012	HOMO-9 → LUMO	84.66
205.07	0.0160	HOMO-5 → LUMO+1	23.20
		HOMO-8 → LUMO	18.56
		HOMO-10 → LUMO	13.92
		HOMO-9 → LUMO	10.08
		HOMO-3 → LUMO+6	7.44
204.78	0.0484	HOMO → LUMO+12	16.50
		HOMO-1 → LUMO+7	14.70
		HOMO-1 → LUMO+4	7.22
		HOMO → LUMO+11	7.11
202.13	0.0445	HOMO-3 → LUMO+5	13.70
		HOMO-1 → LUMO+7	12.47

HOMO → LUMO+12	11.91
HOMO-3 → LUMO+6	11.77
HOMO-1 → LUMO+11	9.14
HOMO-1 → LUMO+4	6.58
HOMO → LUMO+11	5.22

Table S29. Calculated transitions (with a contribution greater than 5%) for [Re(CO)$_3$(ClQ)Hdmpz] (**11**)

Wavelength [nm]	Oscillator strength	Main components	%
423.37	0.1091	HOMO → LUMO	97.34
356.12	0.0609	HOMO-1 → LUMO	95.78
340.78	0.0024	HOMO-2 → LUMO	97.63
322.34	0.0016	HOMO-3 → LUMO	97.09
311.36	0.0360	HOMO → LUMO+1	84.93
		HOMO-6 → LUMO	6.82
		HOMO-5 → LUMO	5.06
304.82	0.0020	HOMO → LUMO+2	77.28
		HOMO-3 → LUMO+2	8.48
		HOMO-1 → LUMO+2	7.46
285.56	0.0038	HOMO-1 → LUMO+2	60.53
		HOMO → LUMO+3	22.26
278.14	0.0054	HOMO-1 → LUMO+3	36.92
		HOMO-2 → LUMO+4	24.66
		HOMO → LUMO+3	19.40
271.00	0.0343	HOMO → LUMO+3	44.10
		HOMO-2 → LUMO+2	20.64
		HOMO-1 → LUMO+2	16.35
268.07	0.0066	HOMO-1 → LUMO+1	91.23
262.32	0.0009	HOMO-4 → LUMO	97.85
261.63	0.0323	HOMO-2 → LUMO+5	37.72
		HOMO-1 → LUMO+3	23.59
		HOMO-2 → LUMO+2	7.93
		HOMO-2 → LUMO+1	6.09
		HOMO-2 → LUMO+4	5.79
257.06	0.0448	HOMO-5 → LUMO	45.66
		HOMO-2 → LUMO+1	21.70
		HOMO-3 → LUMO+1	17.42
255.59	0.0345	HOMO-2 → LUMO+5	32.40
		HOMO-2 → LUMO+2	23.73
		HOMO-1 → LUMO+3	12.65
253.76	0.0191	HOMO-2 → LUMO+1	52.59
		HOMO-5 → LUMO	22.78
		HOMO-2 → LUMO+5	9.23
		HOMO-6 → LUMO	8.85
252.68	0.0078	HOMO-3 → LUMO+2	44.12
		HOMO → LUMO+4	27.06
		HOMO → LUMO+2	8.64
		HOMO-3 → LUMO+1	5.38
251.65	0.0444	HOMO-6 → LUMO	34.96
		HOMO-3 → LUMO+1	28.17
		HOMO → LUMO+4	11.35
		HOMO-2 → LUMO+1	10.69
		HOMO-5 → LUMO	5.60
248.14	0.0147	HOMO → LUMO+4	49.41
		HOMO-3 → LUMO+2	31.14

239.82	0.1942	HOMO-3 → LUMO+1	28.64
		HOMO-6 → LUMO	26.10
		HOMO → LUMO+5	19.32
		HOMO-5 → LUMO	6.75
		HOMO-3 → LUMO+3	5.96
237.07	0.0328	HOMO-3 → LUMO+3	47.76
		HOMO-1 → LUMO+4	15.13
		HOMO → LUMO+6	6.81
236.43	0.0601	HOMO → LUMO+6	37.10
		HOMO-1 → LUMO+4	30.71
		HOMO-3 → LUMO+6	7.07
233.47	0.1179	HOMO → LUMO+5	18.16
		HOMO-1 → LUMO+4	17.00
		HOMO-2 → LUMO+4	15.35
		HOMO-3 → LUMO+3	7.17
		HOMO-2 → LUMO+6	5.85
230.03	0.2734	HOMO → LUMO+5	43.84
		HOMO → LUMO+6	15.92
		HOMO-2 → LUMO+4	5.96
		HOMO-3 → LUMO+1	5.91
228.14	0.0250	HOMO-7 → LUMO	79.00
226.27	0.0368	HOMO-2 → LUMO+4	34.81
		HOMO-7 → LUMO	12.42
		HOMO-2 → LUMO+6	11.36
		HOMO-2 → LUMO+7	5.35
220.69	0.0294	HOMO → LUMO+7	30.09
		HOMO → LUMO+8	23.62
		HOMO → LUMO+10	6.13
219.14	0.0087	HOMO → LUMO+8	56.74
		HOMO → LUMO+7	13.53
		HOMO-3 → LUMO+4	6.18
218.39	0.0379	HOMO-1 → LUMO+6	15.17
		HOMO → LUMO+7	10.77
		HOMO-1 → LUMO+5	7.56
		HOMO → LUMO+10	6.88
		HOMO-1 → LUMO+7	6.67
		HOMO-2 → LUMO+5	6.27
		HOMO → LUMO+8	6.11
216.40	0.0181	HOMO-2 → LUMO+5	23.65
		HOMO-1 → LUMO+6	7.19
		HOMO → LUMO+10	6.73
		HOMO → LUMO+11	6.34
		HOMO-2 → LUMO+11	6.28
		HOMO-2 → LUMO+6	5.66
		HOMO-2 → LUMO+4	5.52
		HOMO-3 → LUMO+5	5.45
214.93	0.0618	HOMO-3 → LUMO+4	23.18
		HOMO-1 → LUMO+7	12.24
		HOMO → LUMO+11	8.31

		HOMO-1 → LUMO+4	7.12
		HOMO-1 → LUMO+5	7.08
		HOMO-1 → LUMO+10	5.38
214.08	0.0405	HOMO-1 → LUMO+5	46.94
		HOMO-3 → LUMO+4	13.63
		HOMO-1 → LUMO+10	6.10
		HOMO → LUMO+10	5.89
212.04	0.0219	HOMO-8 → LUMO	63.69
		HOMO-6 → LUMO+1	7.03
		HOMO-1 → LUMO+7	6.01
211.78	0.0084	HOMO-1 → LUMO+7	34.21
		HOMO-1 → LUMO+10	10.98
		HOMO-8 → LUMO	10.40
		HOMO-4 → LUMO+1	6.34
211.35	0.0007	HOMO-4 → LUMO+1	88.18
		HOMO-8 → LUMO	5.87
210.43	0.0128	HOMO-3 → LUMO+4	31.89
		HOMO → LUMO+7	12.66
		HOMO → LUMO+9	10.90
		HOMO → LUMO+11	9.48
209.02	0.0030	HOMO-11 → LUMO	91.96
208.08	0.0043	HOMO-1 → LUMO+10	25.69
		HOMO → LUMO+10	9.47
		HOMO-2 → LUMO+7	8.44
		HOMO-2 → LUMO+5	7.17
		HOMO-2 → LUMO+10	5.97
206.35	0.0025	HOMO-9 → LUMO	92.24
206.25	0.0437	HOMO-5 → LUMO+1	60.63
		HOMO-4 → LUMO+2	12.61
		HOMO-6 → LUMO+1	5.46
206.12	0.0080	HOMO-4 → LUMO+2	71.27
		HOMO-5 → LUMO+1	10.22
205.43	0.0087	HOMO → LUMO+9	54.78
		HOMO-1 → LUMO+10	7.50
		HOMO-4 → LUMO+2	6.41
204.12	0.0391	HOMO-5 → LUMO+2	38.77
		HOMO-6 → LUMO+2	16.16
		HOMO-10 → LUMO	13.17
		HOMO-6 → LUMO+1	9.11
		HOMO → LUMO+9	5.36
203.36	0.0096	HOMO-5 → LUMO+2	12.98
		HOMO-3 → LUMO+6	12.15
		HOMO-6 → LUMO+1	10.96
		HOMO-1 → LUMO+6	9.38
		HOMO-1 → LUMO+10	8.89
		HOMO → LUMO+9	6.77
		HOMO-10 → LUMO	5.61
		HOMO-6 → LUMO+2	5.11
203.12	0.0645	HOMO-6 → LUMO+1	24.33

		HOMO-10 → LUMO	15.61
		HOMO-3 → LUMO+6	9.11
		HOMO-3 → LUMO+5	6.85
202.00	0.0010	HOMO-2 → LUMO+7	33.18
		HOMO-2 → LUMO+5	15.83
		HOMO-2 → LUMO+6	7.71
200.49	0.0406	HOMO-3 → LUMO+5	15.47
		HOMO → LUMO+10	14.92
		HOMO-1 → LUMO+11	11.92
		HOMO-1 → LUMO+5	7.76
		HOMO-1 → LUMO+6	7.12
		HOMO-3 → LUMO+6	6.95
		HOMO-2 → LUMO+10	6.39

Table S30. Calculated transitions (with a contribution greater than 5%) for [Re(CO)$_3$(ClQ)HPhpz] (**12**)

Wavelength [nm]	Oscillator strength	Main components	%
421.72	0.1080	HOMO → LUMO	97.14
356.27	0.0583	HOMO-1 → LUMO	94.35
339.27	0.0012	HOMO-2 → LUMO	97.69
322.56	0.0023	HOMO-3 → LUMO	93.13
318.15	0.0094	HOMO → LUMO+1	88.28
310.29	0.0341	HOMO → LUMO+2	82.34
		HOMO-6 → LUMO	11.91
302.18	0.0029	HOMO → LUMO+3	69.85
		HOMO-1 → LUMO+3	10.05
285.60	0.1914	HOMO-1 → LUMO+1	43.72
		HOMO-1 → LUMO+3	24.24
		HOMO → LUMO+5	9.90
		HOMO → LUMO+3	5.64
		HOMO-3 → LUMO+5	5.36
283.92	0.0728	HOMO-1 → LUMO+3	39.26
		HOMO-1 → LUMO+1	28.10
		HOMO-1 → LUMO+5	8.77
		HOMO-2 → LUMO+3	8.77
276.50	0.0589	HOMO-4 → LUMO	74.79
		HOMO-1 → LUMO+1	8.01
		HOMO-2 → LUMO+3	6.46
274.25	0.1159	HOMO-2 → LUMO+3	21.08
		HOMO → LUMO+5	17.96
		HOMO-1 → LUMO+5	15.01
		HOMO-4 → LUMO	14.83
		HOMO-1 → LUMO+1	13.09
		HOMO-3 → LUMO+5	6.91
270.23	0.0154	HOMO → LUMO+5	31.79
		HOMO-2 → LUMO+1	17.88
		HOMO-2 → LUMO+3	16.10
		HOMO-1 → LUMO+3	7.82
		HOMO → LUMO+3	7.29
268.32	0.0029	HOMO-2 → LUMO+1	42.08
		HOMO-1 → LUMO+2	32.21
		HOMO-2 → LUMO+5	13.88
		HOMO-2 → LUMO+3	6.52
267.95	0.0076	HOMO-1 → LUMO+2	55.74
		HOMO-2 → LUMO+1	20.33
		HOMO-2 → LUMO+5	5.23
		HOMO → LUMO+5	5.07
260.47	0.0282	HOMO-1 → LUMO+5	29.63
		HOMO-2 → LUMO+5	19.08
		HOMO → LUMO+5	12.13
		HOMO-3 → LUMO+1	6.57
		HOMO-2 → LUMO+3	6.24
257.60	0.0096	HOMO-3 → LUMO+1	40.96

		HOMO → LUMO+4	24.97
		HOMO-3 → LUMO+2	5.83
256.93	0.0259	HOMO → LUMO+4	59.80
		HOMO-3 → LUMO+1	23.34
254.92	0.0499	HOMO-2 → LUMO+2	33.92
		HOMO-3 → LUMO+2	23.82
		HOMO-6 → LUMO	20.09
		HOMO → LUMO+4	8.43
252.48	0.0440	HOMO-2 → LUMO+2	54.31
		HOMO-3 → LUMO+2	20.72
		HOMO-6 → LUMO	9.20
249.77	0.0371	HOMO-3 → LUMO+3	32.24
		HOMO-2 → LUMO+5	19.38
		HOMO-1 → LUMO+5	8.25
		HOMO-3 → LUMO+1	7.87
248.25	0.0201	HOMO-3 → LUMO+3	28.67
		HOMO-2 → LUMO+5	22.68
		HOMO-2 → LUMO+3	7.60
247.91	0.0028	HOMO-1 → LUMO+4	39.81
		HOMO-5 → LUMO+1	30.75
		HOMO-4 → LUMO+4	10.66
245.90	0.0003	HOMO-5 → LUMO	95.49
239.55	0.2215	HOMO-6 → LUMO	34.17
		HOMO-3 → LUMO+2	33.93
		HOMO → LUMO+7	14.87
238.37	0.0241	HOMO → LUMO+6	25.76
		HOMO → LUMO+7	18.04
		HOMO-4 → LUMO+1	17.38
		HOMO → LUMO+8	13.38
236.46	0.0076	HOMO-7 → LUMO	93.16
235.10	0.1301	HOMO-4 → LUMO+1	51.29
		HOMO → LUMO+6	18.55
		HOMO-3 → LUMO+5	11.84
233.40	0.0803	HOMO-3 → LUMO+4	42.61
		HOMO-4 → LUMO+1	20.07
		HOMO → LUMO+8	11.82
		HOMO → LUMO+5	5.24
232.04	0.1526	HOMO → LUMO+6	37.75
		HOMO → LUMO+7	18.98
		HOMO-3 → LUMO+4	7.09
228.77	0.2216	HOMO → LUMO+8	33.51
		HOMO → LUMO+7	24.00
		HOMO → LUMO+9	5.10
227.33	0.0083	HOMO-8 → LUMO	57.33
		HOMO-9 → LUMO	28.66
226.36	0.0495	HOMO-1 → LUMO+4	40.35
		HOMO-5 → LUMO	25.47
		HOMO-4 → LUMO+4	6.64
223.26	0.0132	HOMO → LUMO+6	25.49

		HOMO → LUMO+9	15.62
		HOMO-2 → LUMO+7	9.05
		HOMO-1 → LUMO+8	8.36
		HOMO-1 → LUMO+4	7.08
		HOMO-2 → LUMO+8	6.61
221.07	0.0089	HOMO-4 → LUMO+2	90.05
220.73	0.0210	HOMO → LUMO+9	36.91
		HOMO-2 → LUMO+8	10.02
		HOMO → LUMO+10	7.11
		HOMO-2 → LUMO+9	6.11
219.74	0.0238	HOMO → LUMO+10	17.61
		HOMO-1 → LUMO+7	14.11
		HOMO-2 → LUMO+4	12.66
		HOMO-1 → LUMO+8	8.21
		HOMO → LUMO+12	6.03
		HOMO-2 → LUMO+6	5.82
		HOMO-3 → LUMO+12	5.16
218.57	0.0012	HOMO-2 → LUMO+4	75.48
218.15	0.0114	HOMO → LUMO+10	54.43
		HOMO → LUMO+12	12.85
		HOMO-1 → LUMO+7	5.27
216.45	0.0141	HOMO-4 → LUMO+3	35.32
		HOMO-2 → LUMO+7	15.70
		HOMO-2 → LUMO+9	6.05
		HOMO-2 → LUMO+13	5.54
215.16	0.0193	HOMO-4 → LUMO+3	24.16
		HOMO-1 → LUMO+6	10.14
		HOMO-2 → LUMO+6	8.78
		HOMO → LUMO+9	7.53
		HOMO-2 → LUMO+13	6.20
		HOMO-2 → LUMO+7	5.12
214.84	0.0205	HOMO-4 → LUMO+3	21.09
		HOMO-1 → LUMO+7	19.70
		HOMO-1 → LUMO+9	7.92
		HOMO-1 → LUMO+8	7.48
		HOMO-1 → LUMO+12	5.31
212.87	0.0218	HOMO-3 → LUMO+4	54.61
		HOMO-9 → LUMO	8.92
		HOMO-13 → LUMO	5.14
212.70	0.0044	HOMO-9 → LUMO	21.44
		HOMO-3 → LUMO+4	21.40
		HOMO-13 → LUMO	9.79
		HOMO-7 → LUMO+1	8.09
		HOMO-8 → LUMO	6.46
212.04	0.0017	HOMO-7 → LUMO+1	25.11
		HOMO-1 → LUMO+9	13.37
		HOMO-13 → LUMO	9.04
		HOMO-9 → LUMO	7.77
		HOMO-1 → LUMO+12	5.33

211.25	0.0288	HOMO-1 → LUMO+7	14.12
		HOMO-2 → LUMO+6	13.84
		HOMO → LUMO+13	10.12
		HOMO-13 → LUMO	7.08
		HOMO-3 → LUMO+4	6.18
		HOMO-2 → LUMO+12	5.42
		HOMO-1 → LUMO+6	5.38
211.07	0.0075	HOMO-10 → LUMO	60.68
		HOMO-13 → LUMO	18.24
		HOMO-9 → LUMO	5.52
210.78	0.0139	HOMO-6 → LUMO+1	11.63
		HOMO → LUMO+9	11.27
		HOMO-2 → LUMO+8	10.08
		HOMO-2 → LUMO+9	9.78
		HOMO-2 → LUMO+12	7.10
		HOMO-2 → LUMO+6	5.58
		HOMO-1 → LUMO+9	5.11
210.36	0.0033	HOMO-6 → LUMO+1	78.91
207.76	0.0012	HOMO-1 → LUMO+12	19.31
		HOMO-2 → LUMO+6	12.46
		HOMO-1 → LUMO+9	7.35
		HOMO-3 → LUMO+7	5.17
206.66	0.0722	HOMO-7 → LUMO+1	31.53
		HOMO-1 → LUMO+6	12.29
		HOMO → LUMO+12	7.54
		HOMO-3 → LUMO+8	7.43
		HOMO-3 → LUMO+7	5.98
		HOMO → LUMO+8	5.10
206.56	0.0001	HOMO-11 → LUMO	96.45
205.03	0.0241	HOMO-12 → LUMO	19.78
		HOMO-13 → LUMO	17.85
		HOMO-6 → LUMO+2	17.77
		HOMO-10 → LUMO	13.28
		HOMO-8 → LUMO	6.87
		HOMO-4 → LUMO+5	5.75
204.84	0.0045	HOMO-4 → LUMO+5	41.66
		HOMO-3 → LUMO+8	6.91
		HOMO-3 → LUMO+6	6.34
204.31	0.0079	HOMO → LUMO+11	70.13
202.37	0.0220	HOMO-1 → LUMO+12	20.30
		HOMO-1 → LUMO+8	19.12
		HOMO-5 → LUMO+2	7.46
		HOMO-1 → LUMO+7	6.27
		HOMO-1 → LUMO+9	5.66
		HOMO-4 → LUMO+5	5.14
202.30	0.0334	HOMO-5 → LUMO+2	83.58
200.86	0.0305	HOMO-3 → LUMO+7	11.84
		HOMO-13 → LUMO	10.75
		HOMO-4 → LUMO+4	10.66

		HOMO-6 → LUMO+2	9.31
		HOMO-3 → LUMO+6	6.35
		HOMO-9 → LUMO	5.69
		HOMO-1 → LUMO+13	5.68
		HOMO-8 → LUMO	5.11
200.36	0.0869	HOMO-4 → LUMO+4	36.64
		HOMO-5 → LUMO+1	9.73
		HOMO → LUMO+12	7.63
		HOMO-3 → LUMO+6	5.58
		HOMO-3 → LUMO+7	5.56

Table S31. DFT (PBE0/def2-TZVP/ECP(Re)/IEFPCM(methanol)) calculated compositions of the highest occupied and lowest unoccupied molecular orbitals of studied tricarbonyl rhenium(I) complexes, divided for composing fragments (L_B and L_M – bidentate and monodentate ligand, respectively).

Complex	MO	Re	CO	L_B	L_M
**2**	LUMO	3	3	93	1
	HOMO	13	9	77	1
	HOMO-1	56	30	7	7
	HOMO-2	58	31	10	1
**3**	LUMO	3	3	93	1
	HOMO	13	9	76	2
	HOMO-1	50	26	6	18
	HOMO-2	60	32	7	1
**5**	LUMO	5	3	92	0
	HOMO	12	9	78	1
	HOMO-1	57	30	6	7
	HOMO-2	59	31	9	1
**6**	LUMO	5	2	93	0
	HOMO	12	9	78	1
	HOMO-1	49	27	10	14
	HOMO-2	60	32	8	0
**7**	LUMO	5	2	93	0
	HOMO	12	9	78	1
	HOMO-1	50	26	5	19
	HOMO-2	62	31	6	1
**8**	LUMO	5	2	85	8
	HOMO	12	9	77	2
	HOMO-1	43	23	4	30
	HOMO-2	61	32	6	1
**9**	LUMO	3	3	93	1
	HOMO	11	8	80	1
	HOMO-1	57	30	6	7
	HOMO-2	58	32	9	1
**10**	LUMO	3	3	93	1
	HOMO	12	8	79	1
	HOMO-1	57	29	7	7
	HOMO-2	57	31	10	2
**11**	LUMO	3	2	94	1
	HOMO	12	8	79	1
	HOMO-1	50	25	6	19
	HOMO-2	60	32	7	1
**12**	LUMO	2	3	94	1
	HOMO	12	8	79	1
	HOMO-1	42	21	6	31
	HOMO-2	60	32	7	1

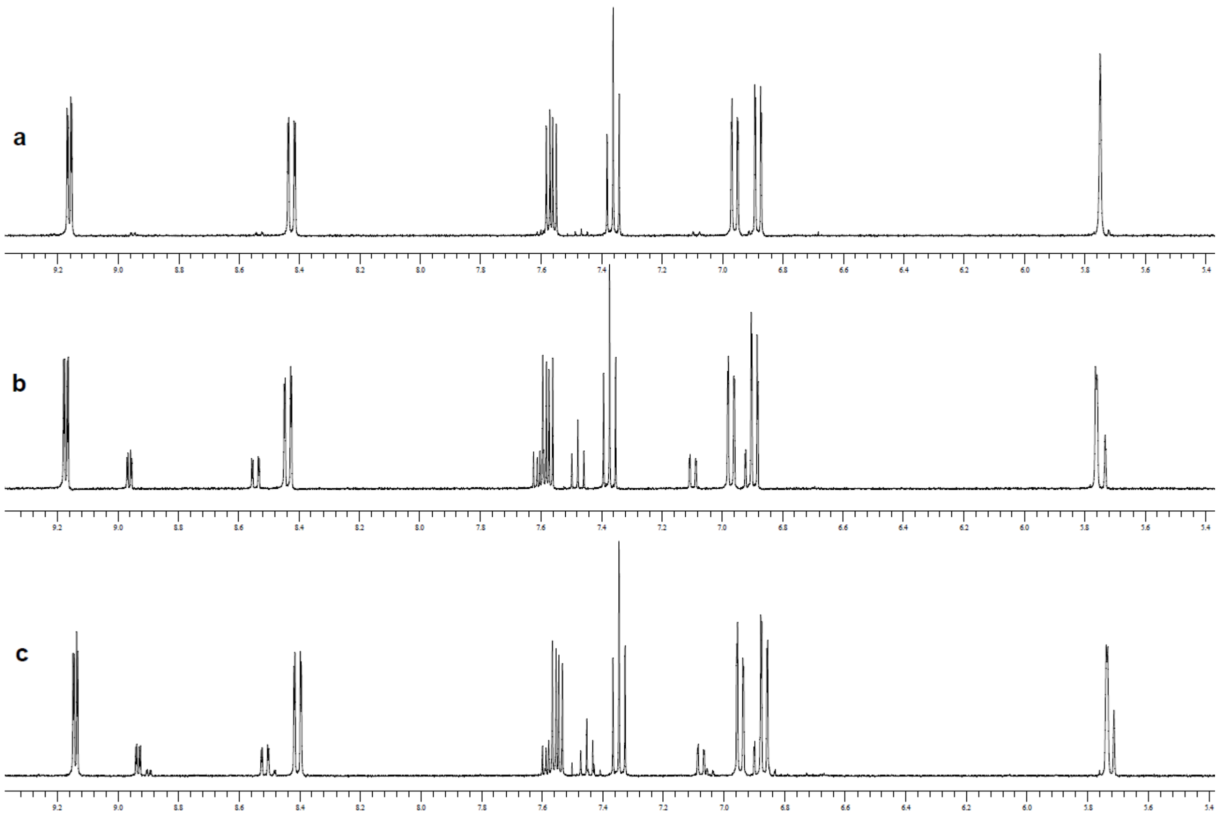

Fig. S34. Exchange of pyrazole ligand with DMSO and H_2O. ^{1}H NMR spectra of complex **3** (6 mg) in DMSO-d_6 (250 μL): a) 5 min. after dissolution in DMSO, b) 40 hrs after dissolution in DMSO, c) 1 week after addition of H_2O.

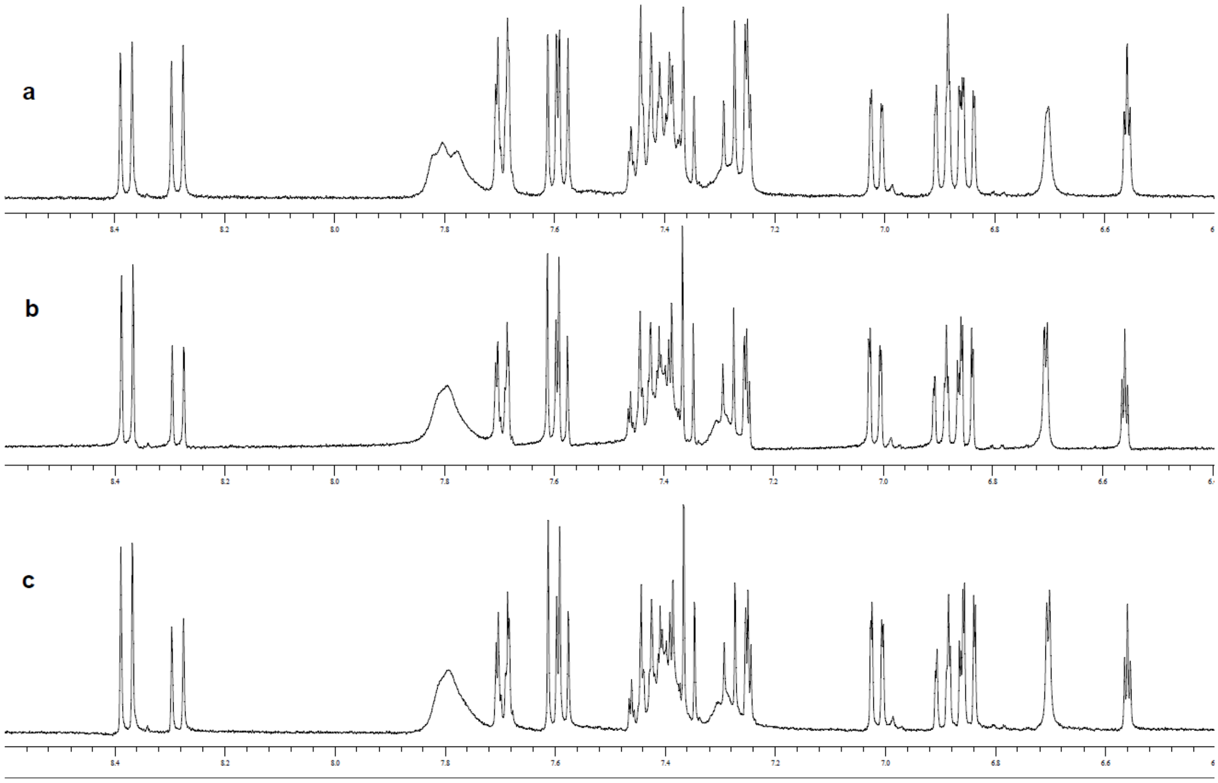

Fig. S35. Exchange of phenylpyrazole ligand with DMSO. ^{1}H NMR spectra of complex **8** (6 mg) in DMSO-d_6 (250 μL): a) 5 min. b) 5 hrs c) 24 hrs after dissolution in DMSO.

[Re(CO)3(MeQ)MeHim]

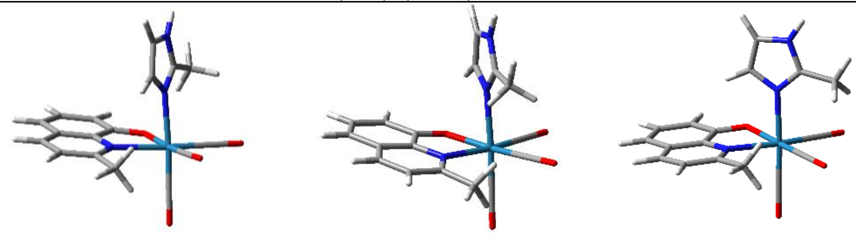

6a **6b** **6c**

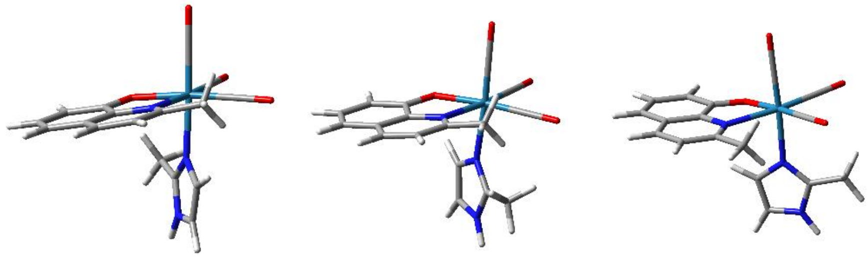

6a' **6b'** **6c'**

[Re(CO)3(MeQ)Hdmpz]

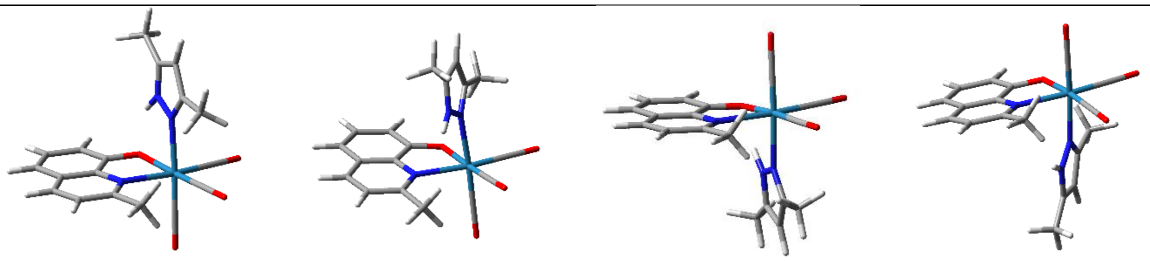

7a **7b** **7a'** **7b'**

[Re(CO)3(MeQ)DMSO]

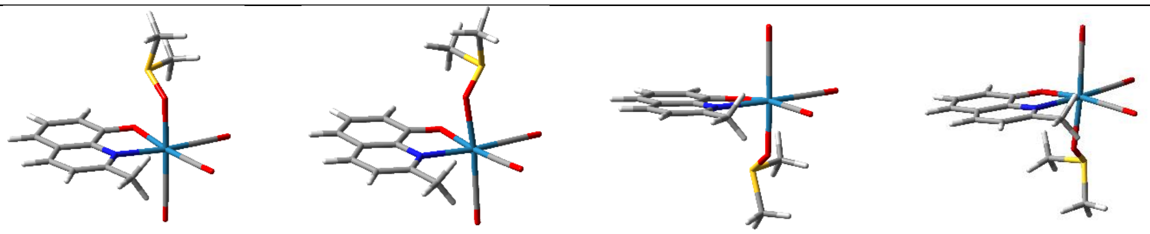

dmso-a **dmso-b** **dmso-a'** **dmso-b'**

Fig. S36. Structures of tricarbonyl rhenium(I) complexes with 2-methyl-8-hydroxyquinolinato ligand (MeQ^{-}) and 2-methylimidazole (MeHim), or 3,5-dimethylpyrazole (Hdmpz), or DMSO calculated at the B3LYP/aug-cc-pVTZ(N,O,S,C,H)/def2TZVP(Re) level. The structures denoted by *prime* are isoenergetic with their *no prime* counterparts (see Table S32).

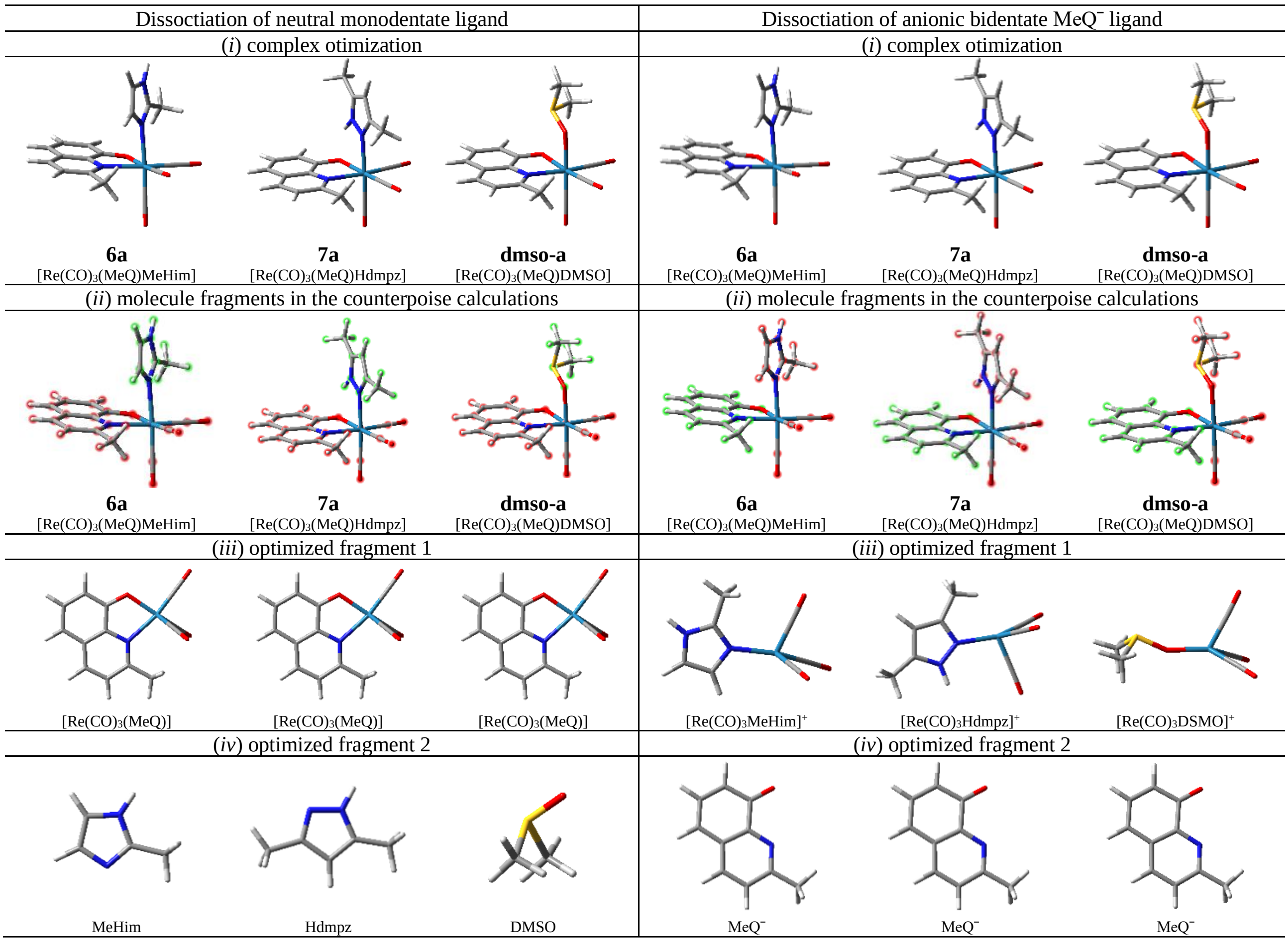

Fig. S37. Scheme of the calculation of interaction energies corrected for BSSE (by the Boys–Bernardi counterpoise, CP, method) and deformation of monomers upon complexation: (*i*) optimization of the complex; (*ii*) division of the complex on the appropriate fragments and Counterpoise=2 calculations. The complex fragments 1 and 2 in the CP calculations are highlighted in red and green, respectively. (*iii*) optimization of appropriate fragments 1 and (*iv*) 2. This scheme is shown for the most stable tricarbonyl rhenium(I) complexes with 2-methyl-8-hydroxyquinolinato (MeO‾) and 2-methylimidazole (MeHim), or 3,5-dimethylpyrazole (Hdmpz), or DMSO obtained at the B3LYP/aug-cc-pVTZ(N,O,S,C,H)/def2TZVP(Re) level. When defining the Counterpoise=2 calculations the charge and multiplicity of which specie (dimer, fragment 1, fragment 2) must be defined. Here, complexes and their appropriate fragments are neutral singlets when MeHim, Hdmpz or DMSO are defined as fragment 2. However, when fragment 2 is anionic MeO‾, then the whole complex is neutral singlet and fragment 1 is a cation singlet ($[Re(CO)_3MeHim]^+$, $[Re(CO)_3Hdmpz]^+$, or $[Re(CO)_3DMSO]^+$).

Table S32. Energies (1-7, a.u.)* of appropriate complex fragments used for calculation of the seven-point interaction energy (ΔE_7) and its components (kcal/mol) for biding the bidentate (MeQ$^-$) and monodentate ligands (Hdmpz, dmso, or MeHim) with the $Re(CO)_3$ system: uncorrected interaction energy (ΔE), counterpoise corrected interaction energy (ΔE_{CP}), basis set superposition error (BSSE), deformation energy (ΔE_{def}). Structures of the complexes are shown in Fig. S36 and the scheme of counterpoise calculations are shown in Fig. S37. The most stable, isoenegetic structures are highlighted in red. Results obtained at the B3LYP/aug-cc-pVTZ(N,O,S,C,H)/def2TZVP(Re) computational level.

**Dissociation of neutral monodentate ligand**												
	1	2	3	4	5	6	7	ΔE	ΔE_{CP}	BSSE	ΔE_7	ΔE_{def}
6a	-1200.348636	-934.6575062	-265.640665	-934.6568579	-265.640368	-934.672846	-265.641630	-32.26	-31.67	0.59	-20.84	10.82
6b	-1200.346561	-934.6570426	-265.640470	-934.6563873	-265.640179	-934.672846	-265.641630	-31.37	-30.78	0.59	-19.54	11.24
6c	-1200.346362	-934.6556814	-265.640393	-934.6550213	-265.640113	-934.672846	-265.641630	-32.15	-31.56	0.59	-19.42	12.14
6a'	-1200.348636	-934.657513	-265.640663	-934.656865	-265.640366	-934.672846	-265.641630	-32.26	-31.66	0.59	-20.84	10.82
6b'	-1200.346561	-934.657040	-265.640470	-934.656385	-265.640179	-934.672846	-265.641630	-31.37	-30.78	0.59	-19.54	11.24
6c'	-1200.346362	-934.6556886	-265.640393	-934.6550285	-265.640113	-934.672846	-265.641630	-32.14	-31.55	0.59	-19.42	12.13
7a	-1239.667211	-934.656218	-304.955315	-934.655521	-304.954984	-934.672846	-304.957250	-35.58	-34.94	0.64	-22.64	12.29
7b	-1239.659393	-934.657496	-304.956378	-934.656814	-304.956057	-934.672846	-304.957250	-29.19	-28.56	0.63	-17.75	10.81
7a'	-1239.667210	-934.656226	-304.955330	-934.655530	-304.955000	-934.672846	-304.957250	-35.57	-34.92	0.64	-22.65	12.28
7b'	-1239.659393	-934.657503	-304.956376	-934.656821	-304.956054	-934.672846	-304.957250	-29.19	-28.56	0.63	-17.75	10.81
dmso-a	-1488.004992	-934.6597432	-553.3037841	-934.6593039	-553.3034085	-934.672846	-553.306004	-26.53	-26.02	0.51	-15.89	10.13
dmso-b	-1488.004214	-934.6598253	-553.3045143	-934.6594031	-553.3041906	-934.672846	-553.306004	-25.49	-25.02	0.47	-15.45	9.57
dmso-a'	-1488.004992	-934.6597437	-553.3037872	-934.6593044	-553.3034117	-934.672846	-553.306004	-26.53	-26.02	0.51	-15.89	10.12
dmso-b'	-1488.004214	-934.6598261	-553.3045162	-934.6594039	-553.3041926	-934.672846	-553.306004	-25.49	-25.02	0.47	-15.45	9.57
**Dissociation of anionic bidentate MeQ$^-$ ligand**												
	1	2	3	4	5	6	7	ΔE	ΔE_{CP}	BSSE	ΔE_7	ΔE_{def}
6a	-1200.348636	-683.9551615	-516.0904351	-683.9543529	-516.0899904	-683.965563	-516.09802	-190.95	-190.16	0.79	-178.09	12.07
6b	-1200.346561	-683.953981	-516.0901682	-683.9531551	-516.0897302	-683.965563	-516.09802	-190.56	-189.77	0.79	-176.78	12.99
6c	-1200.346362	-683.9540569	-516.0905931	-683.9532513	-516.0901602	-683.965563	-516.09802	-190.10	-189.33	0.78	-176.67	12.66
6a'	-1200.348636	-683.9551597	-516.090435	-683.9543511	-516.0899902	-683.965563	-516.09802	-190.95	-190.16	0.79	-178.09	12.07
6b'	-1200.346561	-683.9539799	-516.0901664	-683.953154	-516.0897284	-683.965563	-516.09802	-190.56	-189.77	0.79	-176.78	12.99
6c'	-1200.346362	-683.9540497	-516.0905935	-683.9532444	-516.0901606	-683.965563	-516.09802	-190.11	-189.33	0.78	-176.67	12.66
7a	-1239.667211	-723.2586785	-516.0891836	-723.2578211	-516.0887337	-723.280169	-516.09802	-201.21	-200.39	0.82	-180.54	19.85
7b	-1239.659393	-723.2641161	-516.0904338	-723.2632864	-516.0899833	-723.280169	-516.09802	-192.10	-191.29	0.80	-175.65	15.64
7a'	-1239.66721	-723.2587084	-516.0891744	-723.2578517	-516.0887247	-723.280169	-516.09802	-201.20	-200.38	0.82	-180.54	19.84
7b'	-1239.659393	-723.2641044	-516.090429	-723.2632746	-516.0899786	-723.280169	-516.09802	-192.11	-191.30	0.80	-175.65	15.65
dmso-a	-1488.004992	-971.6029146	-516.0894691	-971.6019794	-516.0890687	-971.631027	-516.09802	-197.00	-196.16	0.84	-172.32	23.85
dmso-b	-1488.004214	-971.6064553	-516.0903204	-971.6055885	-516.0899201	-971.631027	-516.09802	-193.72	-192.92	0.80	-171.87	21.05
dmso-a'	-1488.004992	-971.6029171	-516.0894789	-971.6019819	-516.0890785	-971.631027	-516.09802	-197.00	-196.16	0.84	-172.32	23.84
dmso-b'	-1488.004214	-971.6064559	-516.0903204	-971.6055892	-516.0899201	-971.631027	-516.09802	-193.72	-192.92	0.80	-171.87	21.05

*the energies in the 1-7 columns are the following : 1 – energy of optimized complex, 2 – energy of fragment 1 in the geometry and basis set of the complex, 3 – energy of fragment 2 in the geometry and basis set of the complex, 4 – energy of fragment 1 in the geometry of the complex but its basis set, 5 – energy of fragment 2 in the geometry of the complex but its basis set, 6 – energy of optimized fragment 1 in its basis set, 7 – energy of optimized fragment 2 in its basis set. The energies 1-5 are obtained during the Counterpoise=2 calculations for estimation of ΔE_{CP} energies and BSSE. Additional two calculations, energies in columns 6 and 7, are performed to estimate the deformation of monomers upon complexation. See Fig. S37 which monomers (in columns 6 and 7) were used in each calculation. The energies of optimised monomers in their basis set are the following: $[Re(CO)_3(MeQ)]$ = -934.672846 a.u.; MeHim: = -265.641630 a.u.; Hdmpz = -304.957250 a.u; DMSO = -553.306004 au; MeO^- = -516.088725 a.u.; $[Re(CO)_3MeHim]^+$ = -683.965563 a.u.; $[Re(CO)_3Hdmpz]^+$ = -723.280169 a.u; $[Re(CO)_3DSMO]^+$ = -971.631027 a.u.

The energies are defined as: ΔE = (1-4-5); ΔE_{CP} = (1-2-3); BSSE = (4+5)-(2+3) = ΔE_{CP} - ΔE; ΔE_7 = (1-2-3+4+5-6-7); ΔE_{def} = (4+5)-(6+7) = ΔE_7 - ΔE_{CP}. The factor of 627.5095 was used to convert a.u. units into kcal/mol.

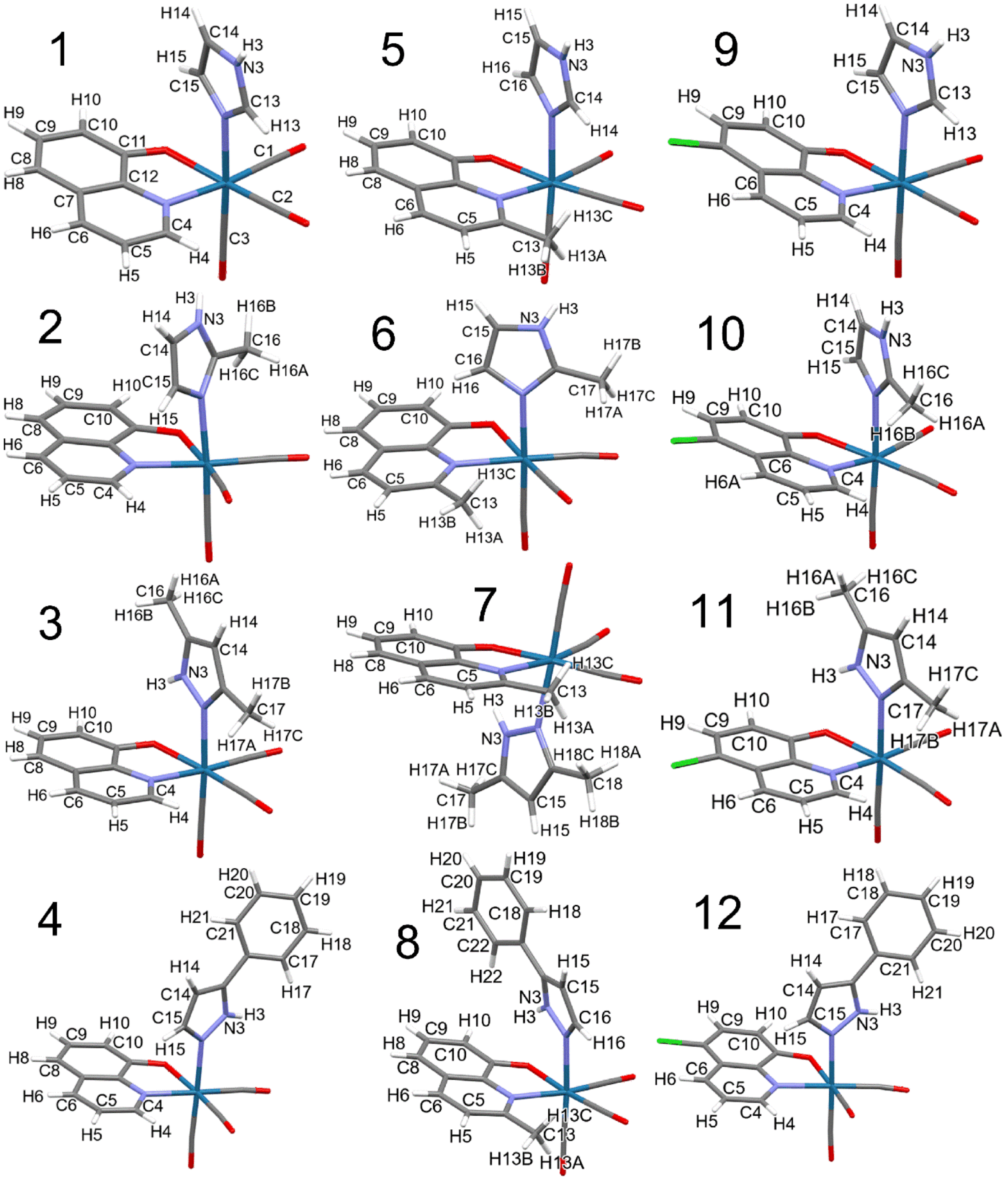

Figure S38. Atom labeling in tricarbonyl rhenium(I) complexes for NMR assignment.

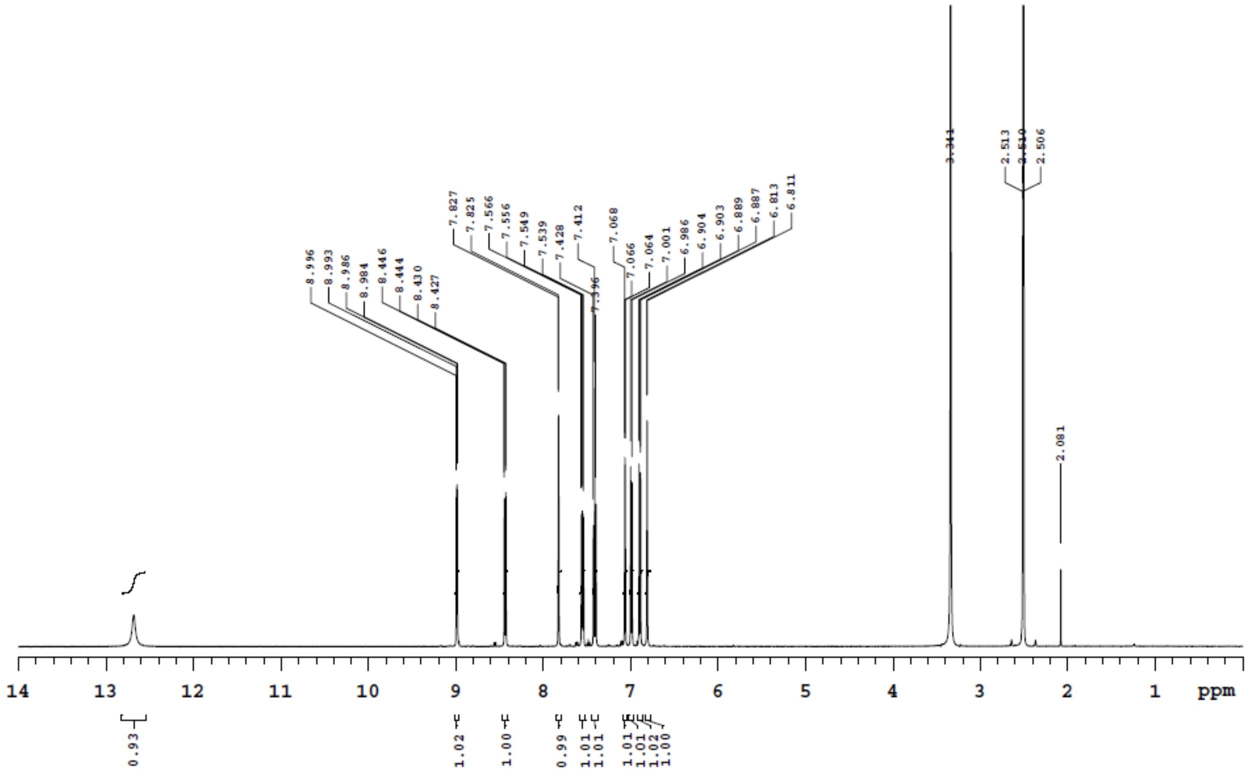

Fig. S39a. ^{1}H NMR spectrum of [Re(CO)$_3$(Q)Him] (**1**) in DMSO-*d6*.

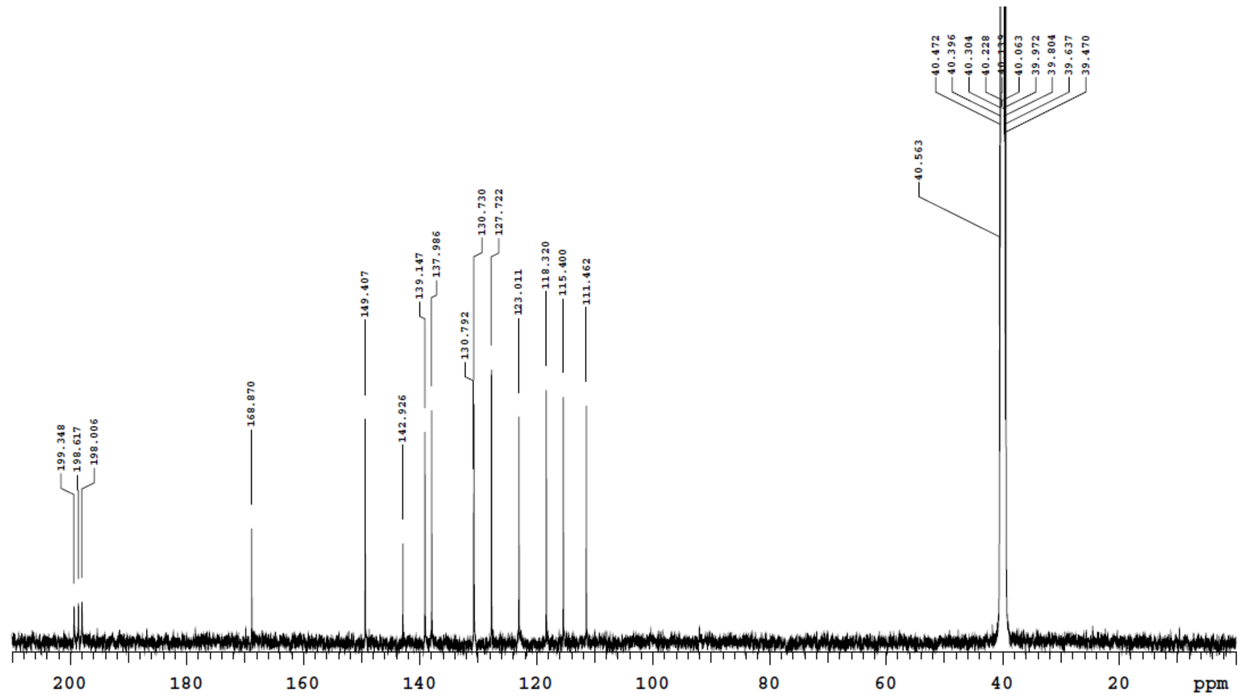

Fig. S39b. ^{13}C NMR spectrum of [Re(CO)$_3$(Q)Him] (**1**) in DMSO-*d6*.

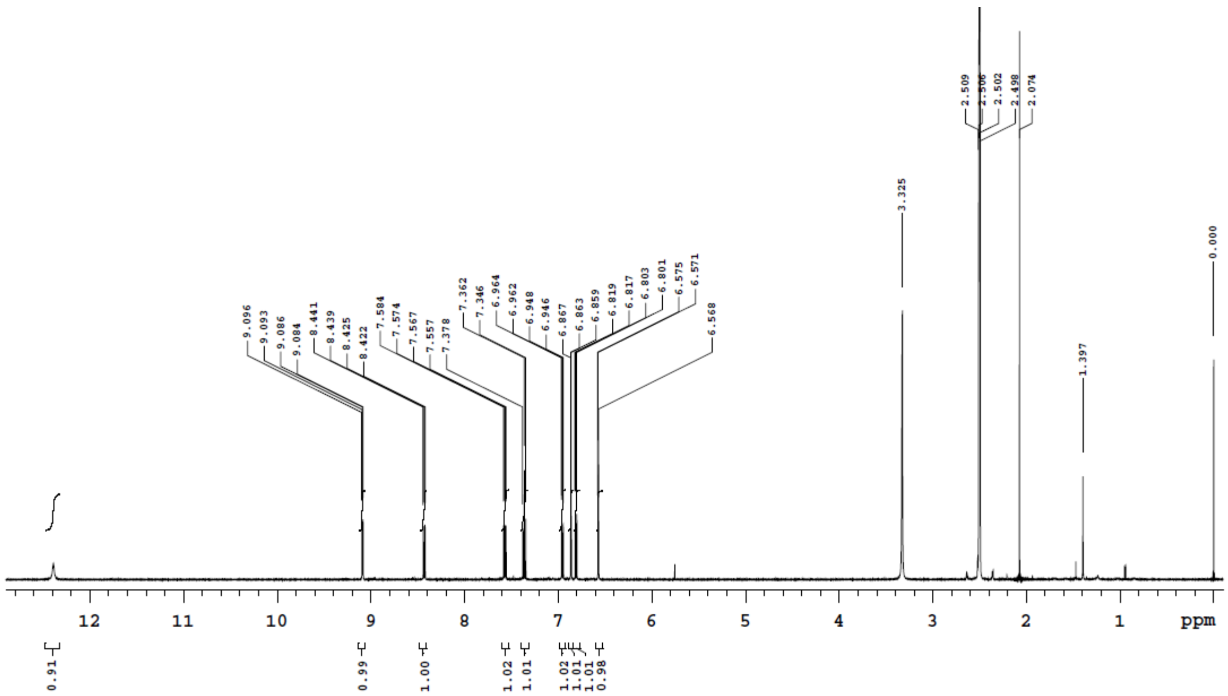

Fig. S40a. ^{1}H NMR spectrum of [Re(CO)$_3$(Q)MeHim] (**2**) in DMSO-*d6*.

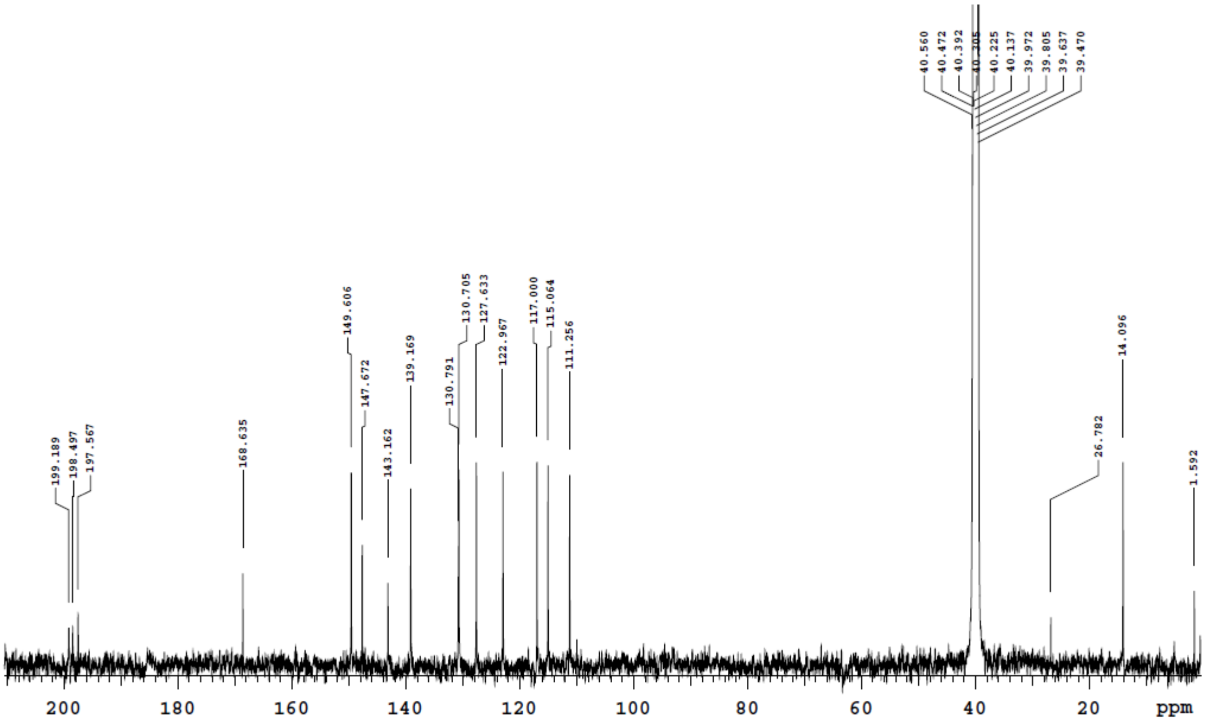

Fig. S40b. ^{13}C NMR spectrum of [Re(CO)$_3$(Q)MeHim] (**2**) in DMSO-*d6*.

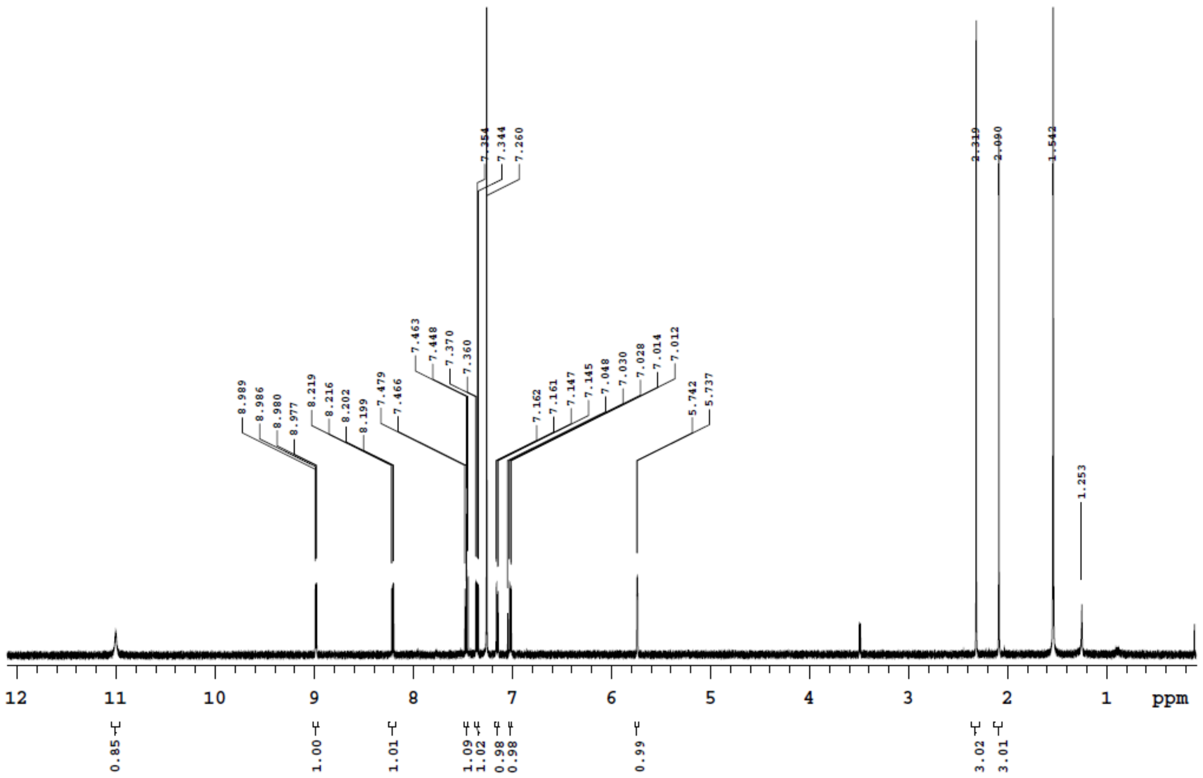

Fig. S41a. ^{1}H NMR spectrum of [Re(CO)$_3$(Q)Hdmpz] (**3**) in $CDCl_3$.

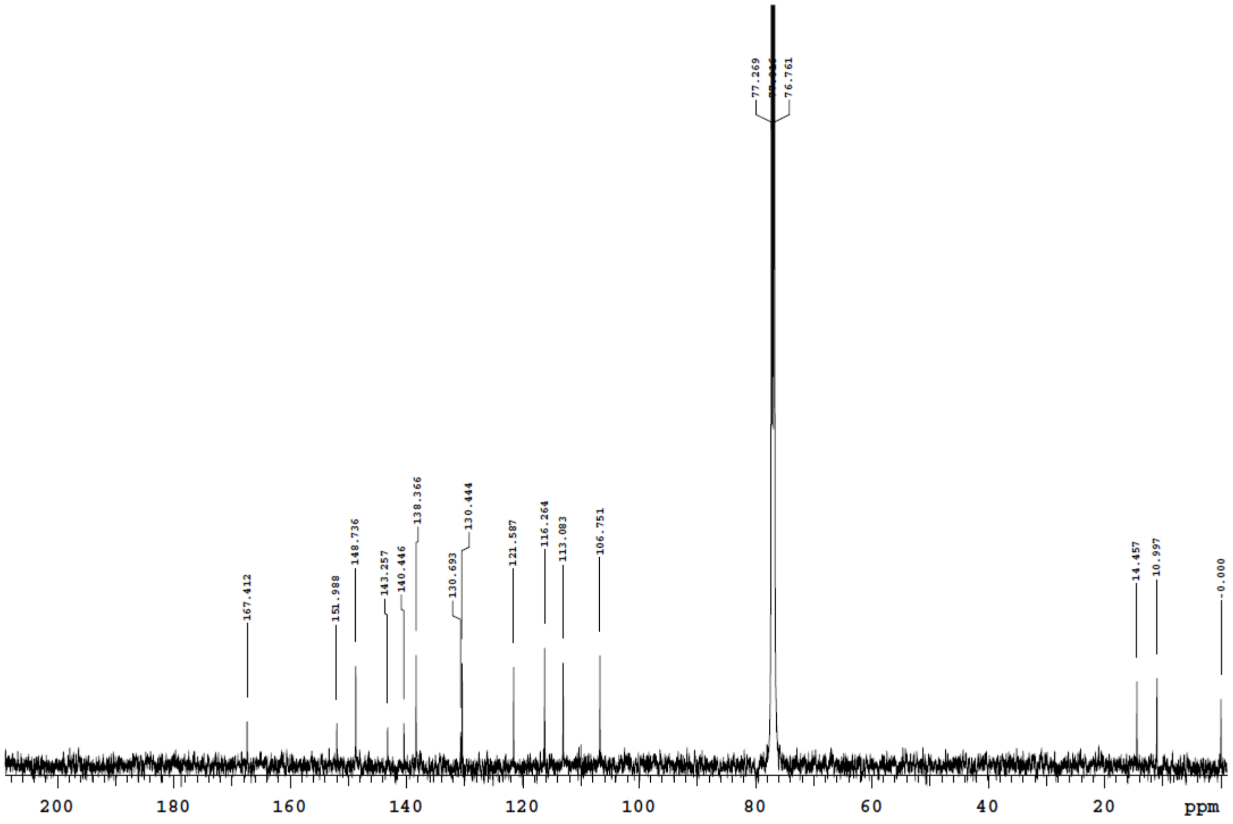

Fig. S41b. ^{13}C NMR spectrum of [Re(CO)$_3$(Q)Hdmpz] (**3**) in $CDCl_3$.

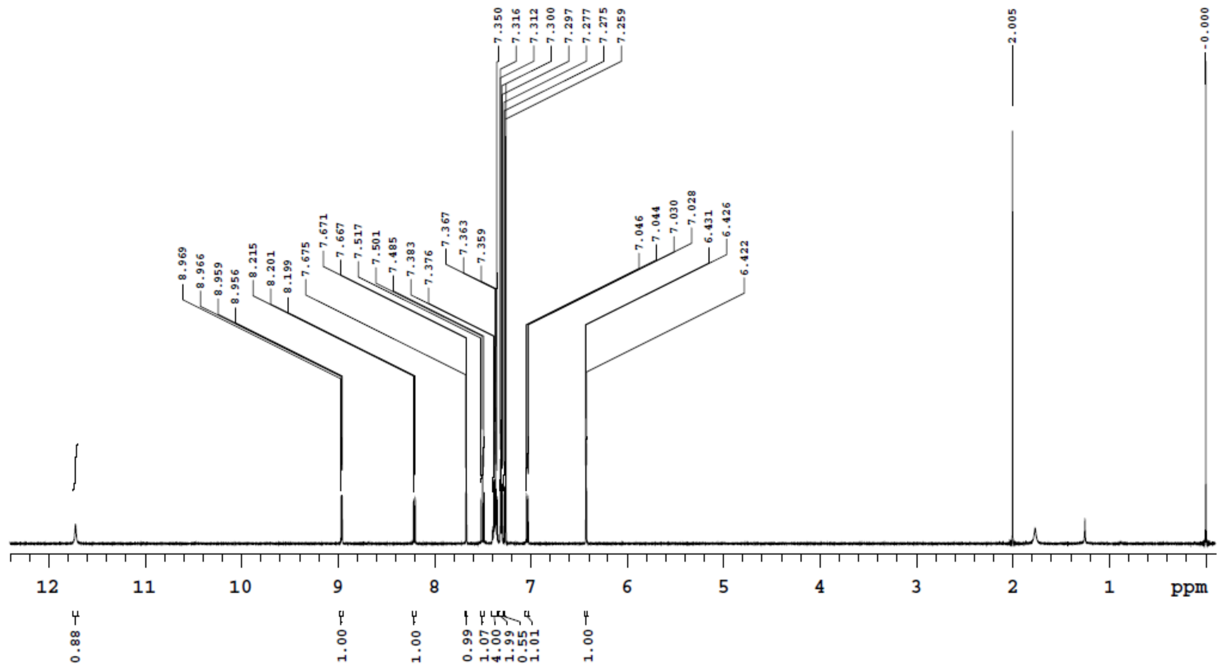

Fig. S42a. ^{1}H NMR spectrum of [Re(CO)$_3$(Q)HPhpz] (**4**) in $CDCl_3$.

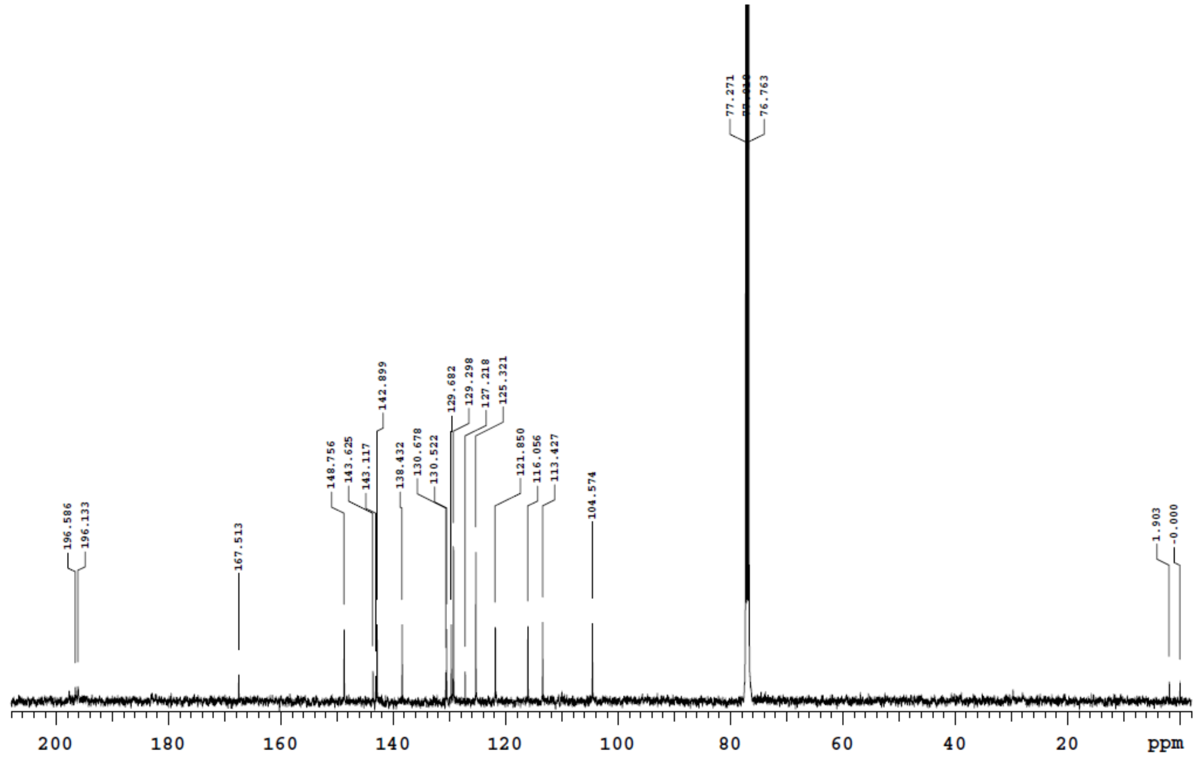

Fig. S42b. ^{13}C NMR spectrum of [Re(CO)$_3$(Q)HPhpz] (**4**) in $CDCl_3$.

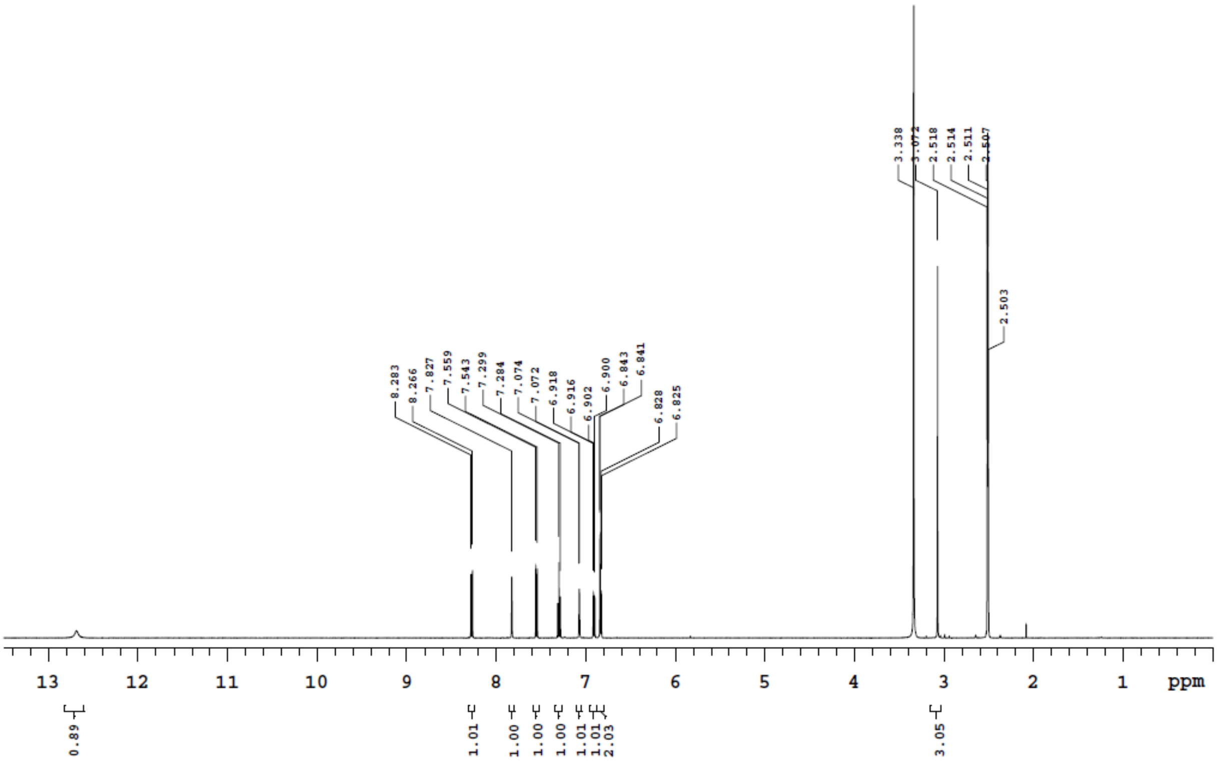

Fig. S43a. ^{1}H NMR spectrum of [Re(CO)$_3$(MeQ)Him] (**5**) in DMSO-*d6*.

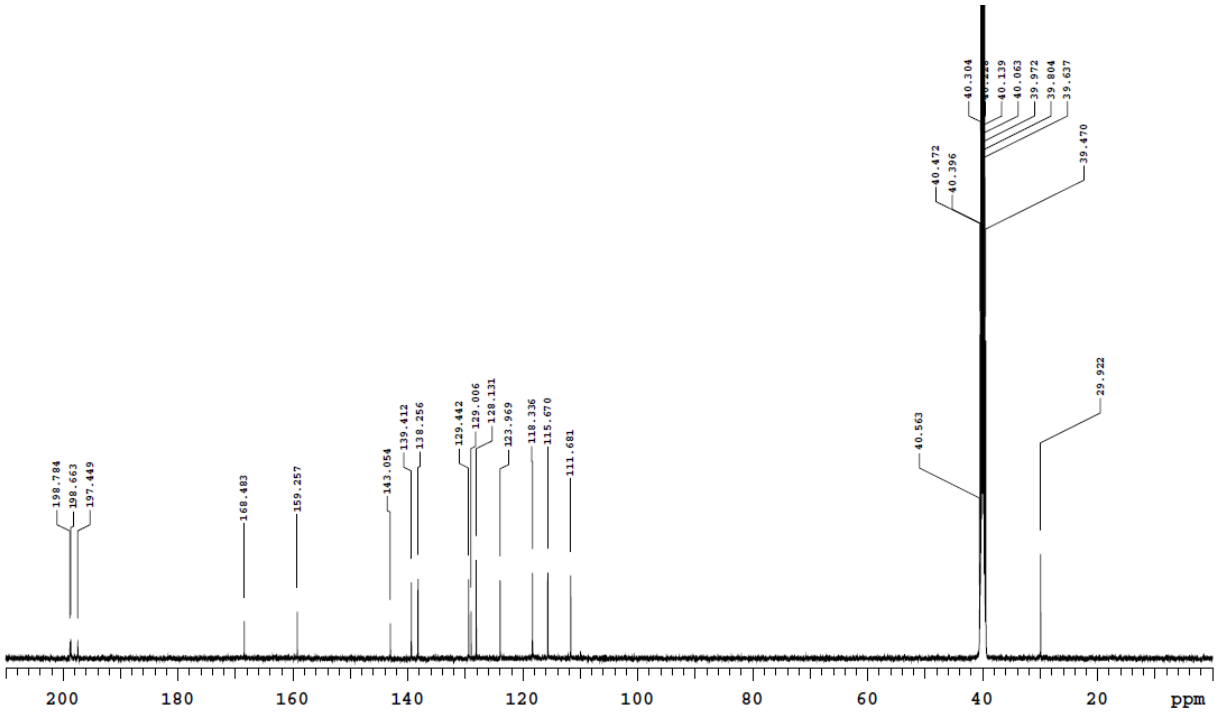

Fig. S43b. ^{13}C NMR spectrum of [Re(CO)$_3$(MeQ)Him] (**5**) in DMSO-*d6*.

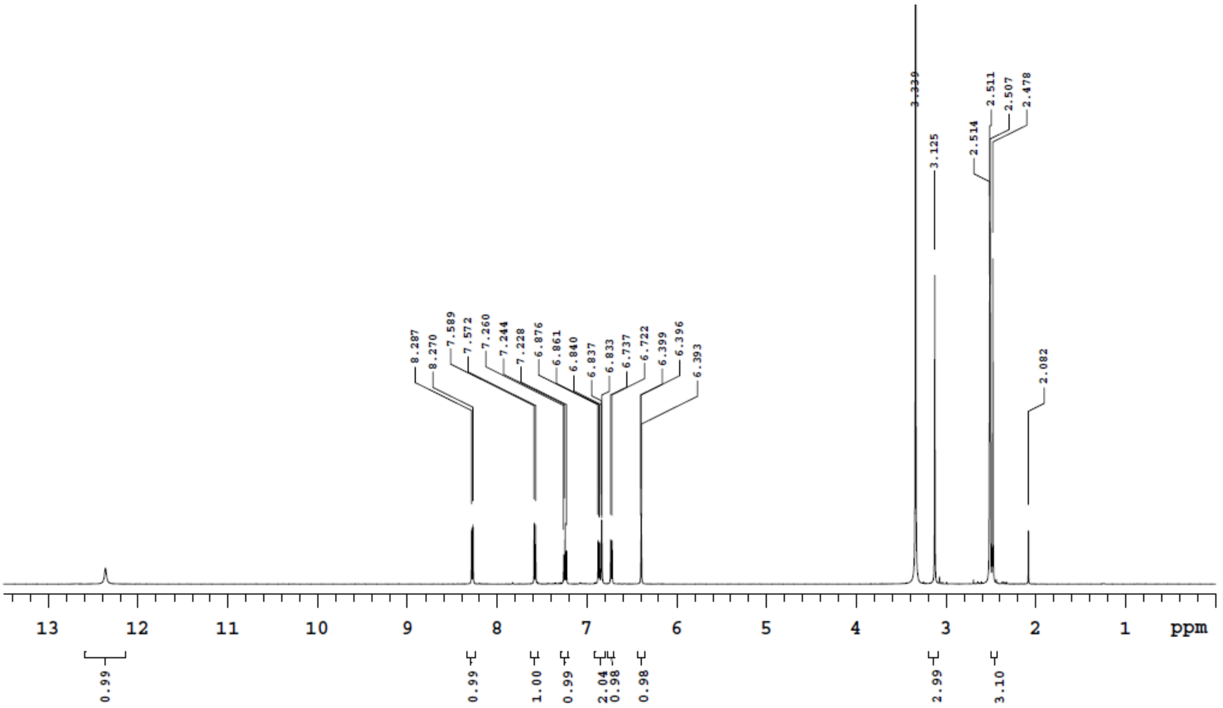

Fig. S44a. ^{1}H NMR spectrum of [Re(CO)$_3$(MeQ)MeHim] (**6**) in DMSO-*d6*.

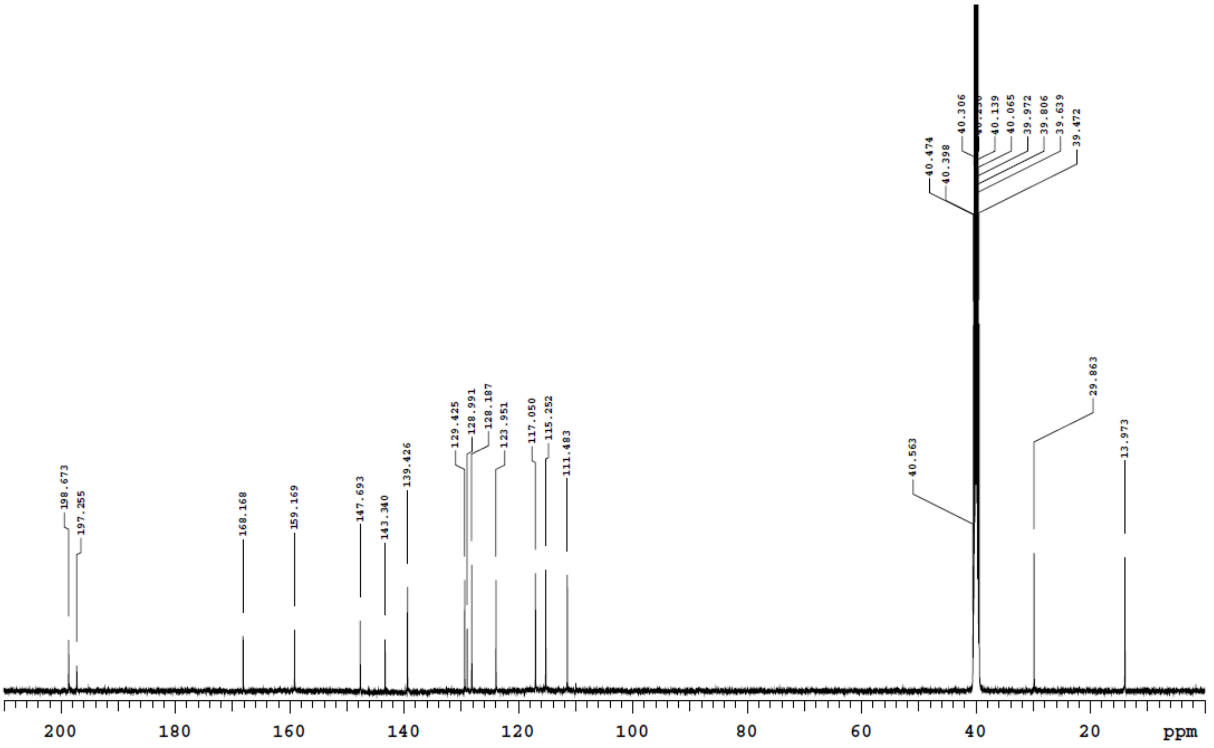

Fig. S44b. ^{13}C NMR spectrum of [Re(CO)$_3$(MeQ)MeHim] (**6**) in DMSO-*d6*.

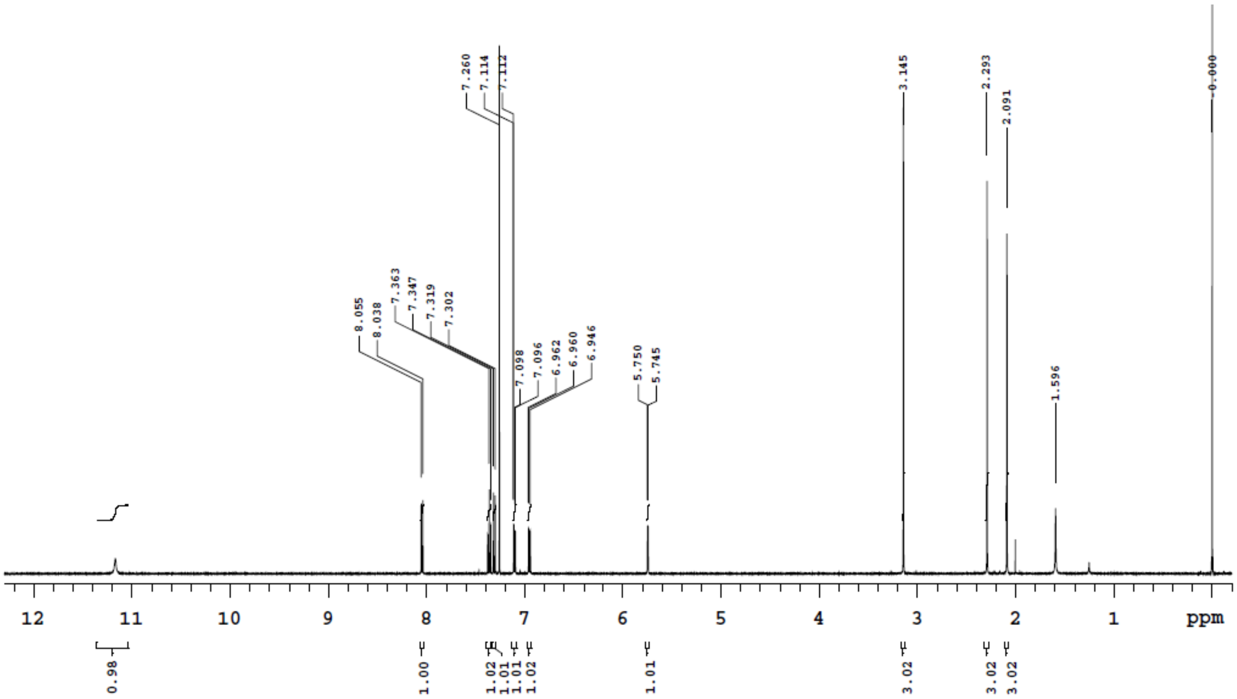

Fig. S45a. ^{1}H NMR spectrum of [Re(CO)$_3$(MeQ)Hdmpz] (**7**) in $CDCl_3$.

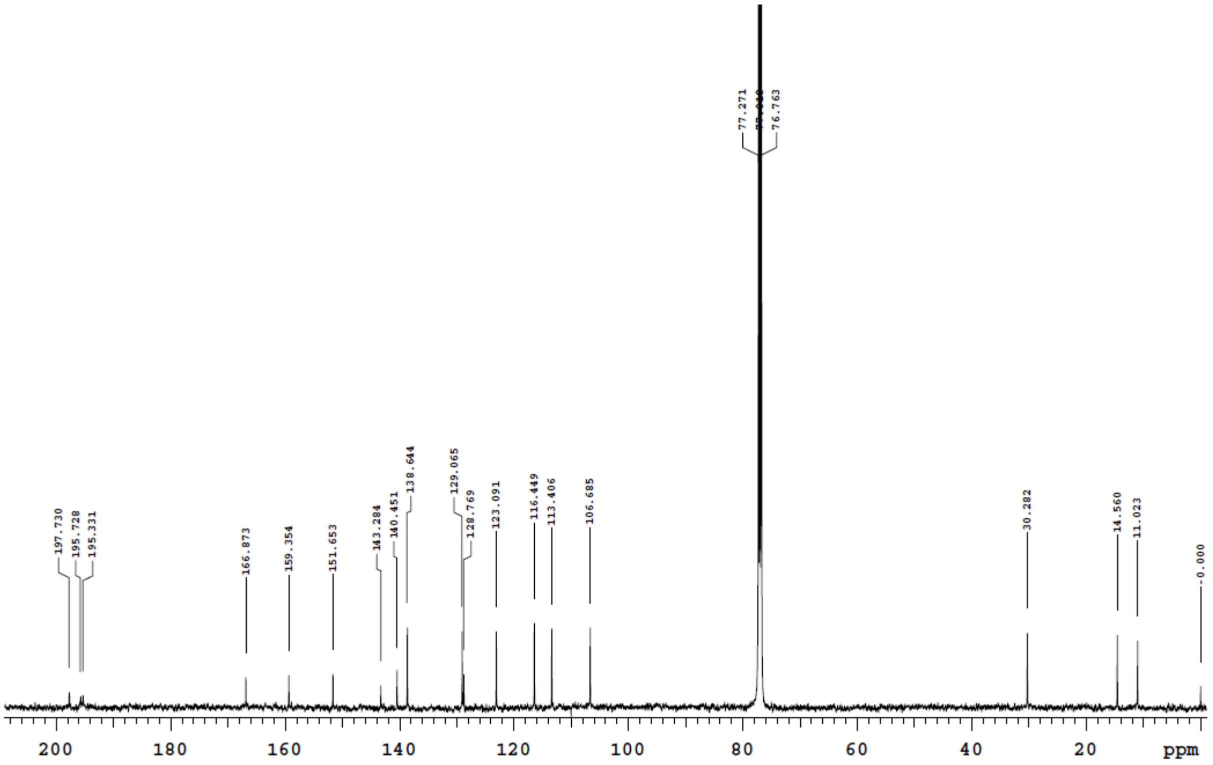

Fig. S45b. ^{13}C NMR spectrum of [Re(CO)$_3$(MeQ)Hdmpz] (**7**) in $CDCl_3$.

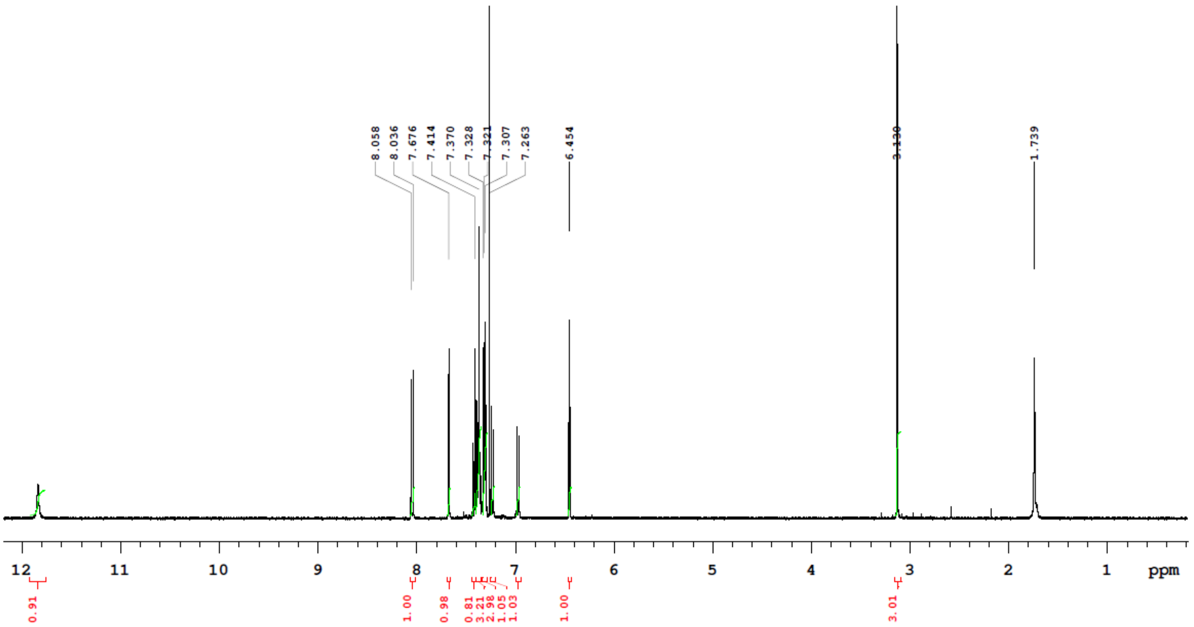

Fig. S46a. ^{1}H NMR spectrum of [Re(CO)$_3$(MeQ)HPhpz] (**8**) in $CDCl_3$.

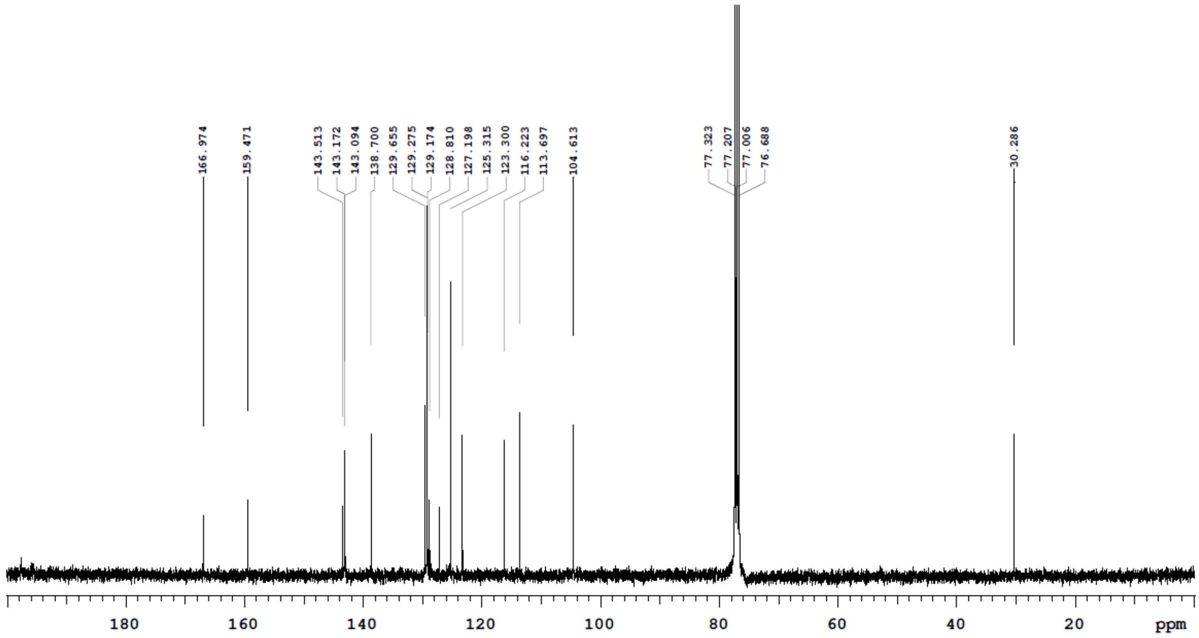

Fig. S46b. ^{13}C NMR spectrum of [Re(CO)$_3$(MeQ)HPhpz] (**8**) in $CDCl_3$.

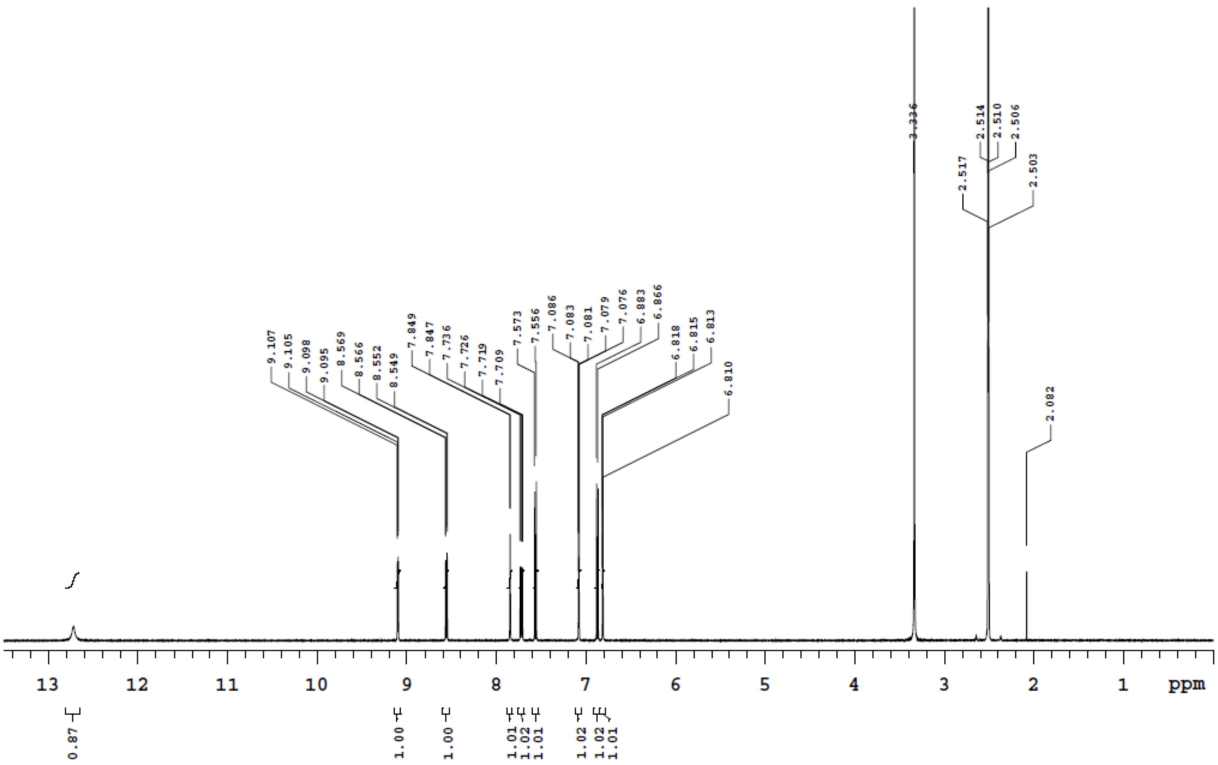

Fig. S47a. ^{1}H NMR spectrum of [Re(CO)$_3$(ClQ)Him] (**9**) in DMSO-*d6*.

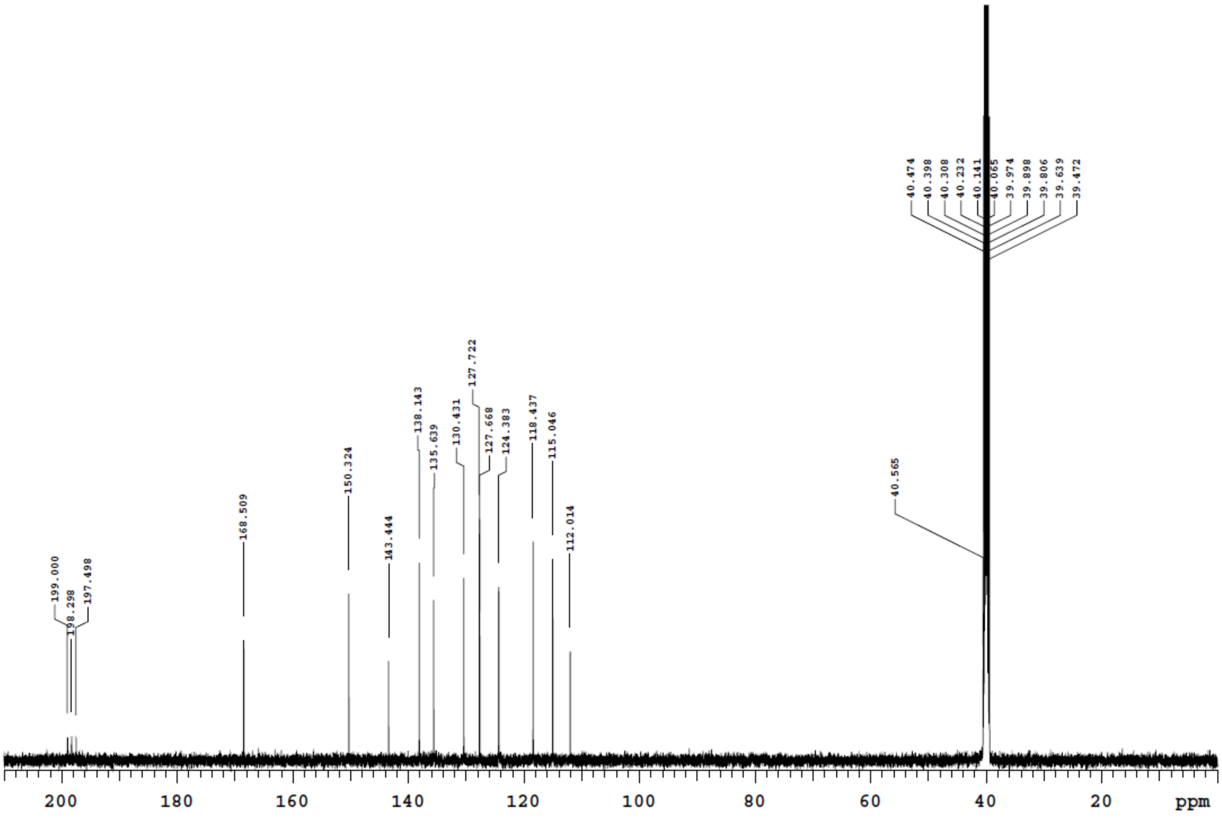

Fig. S47b. ^{13}C NMR spectrum of [Re(CO)$_3$(ClQ)Him] (**9**) in DMSO-*d6*.

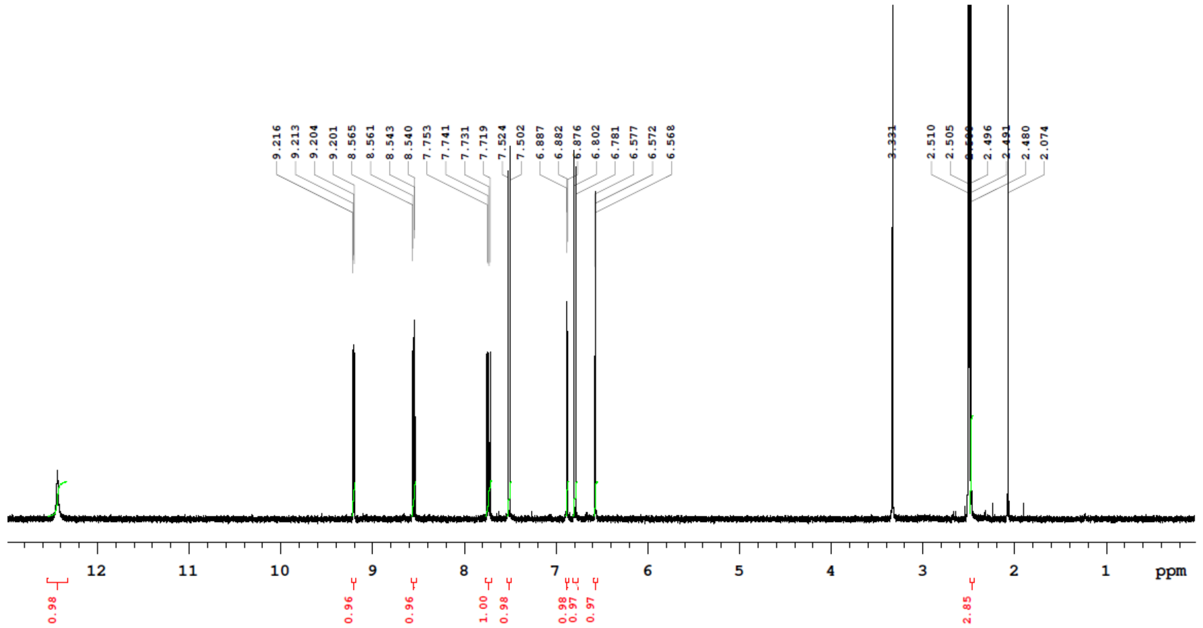

Fig. S48a. ^{1}H NMR spectrum of [Re(CO)$_3$(ClQ)MeHim] (**10**) in DMSO-*d6*.

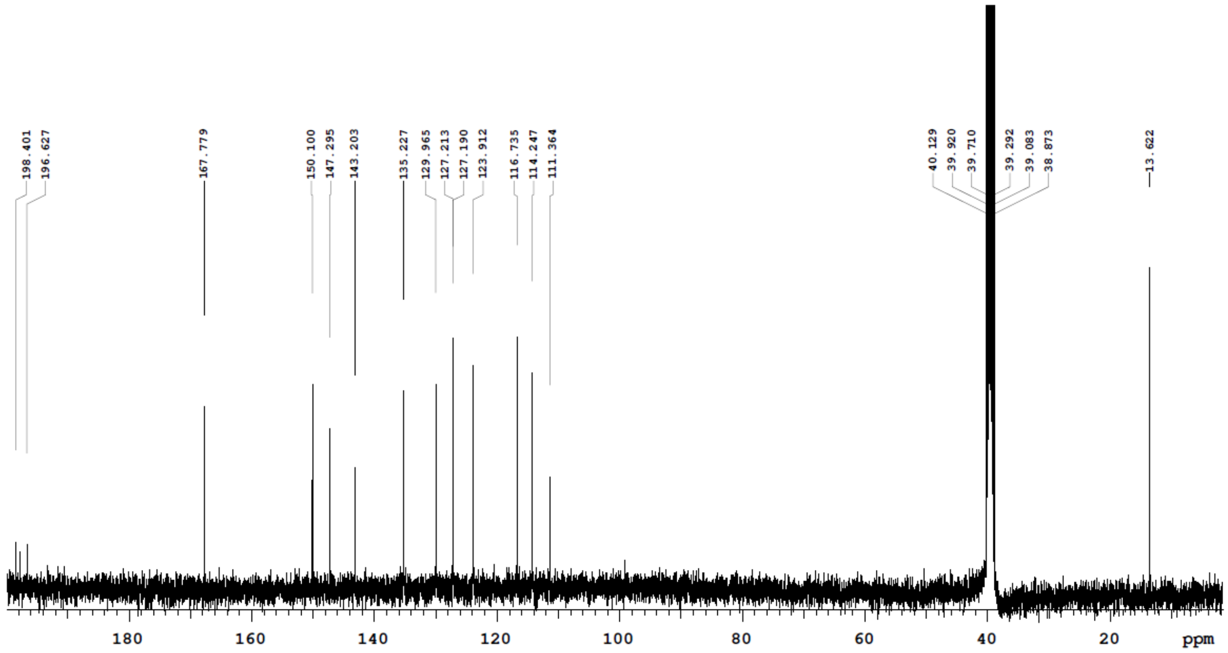

Fig. S48b. ^{13}C NMR spectrum of [Re(CO)$_3$(ClQ)MeHim] (**10**) in DMSO-*d6*.

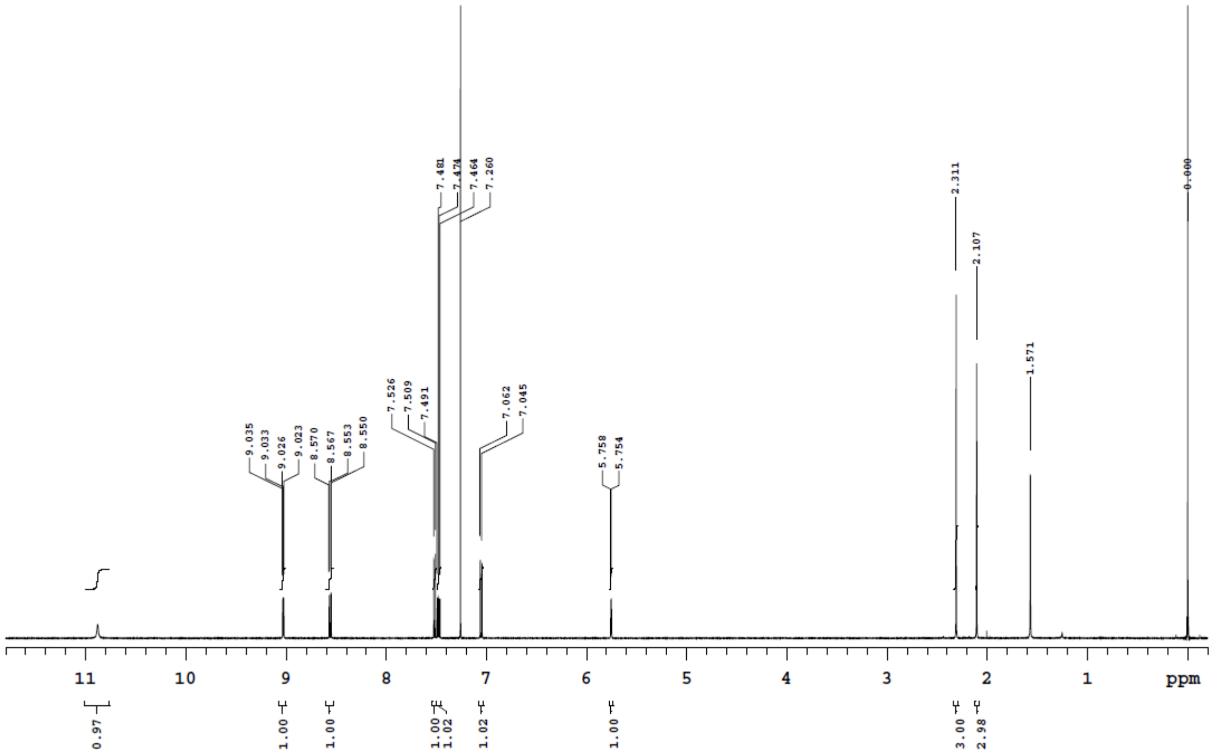

Fig. S49a. ^{1}H NMR spectrum of [Re(CO)$_3$(ClQ)Hdmpz] (**11**) in $CDCl_3$.

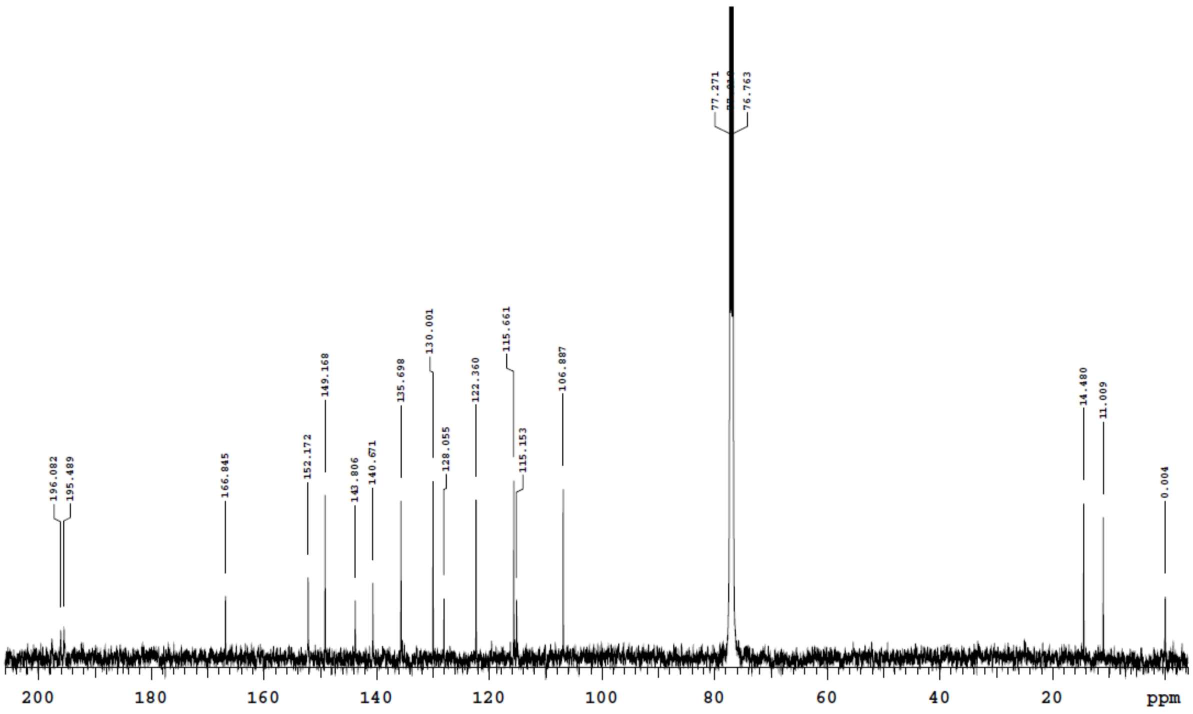

Fig. S49b. ^{13}C NMR spectrum of [Re(CO)$_3$(ClQ)Hdmpz] (**11**) in $CDCl_3$.

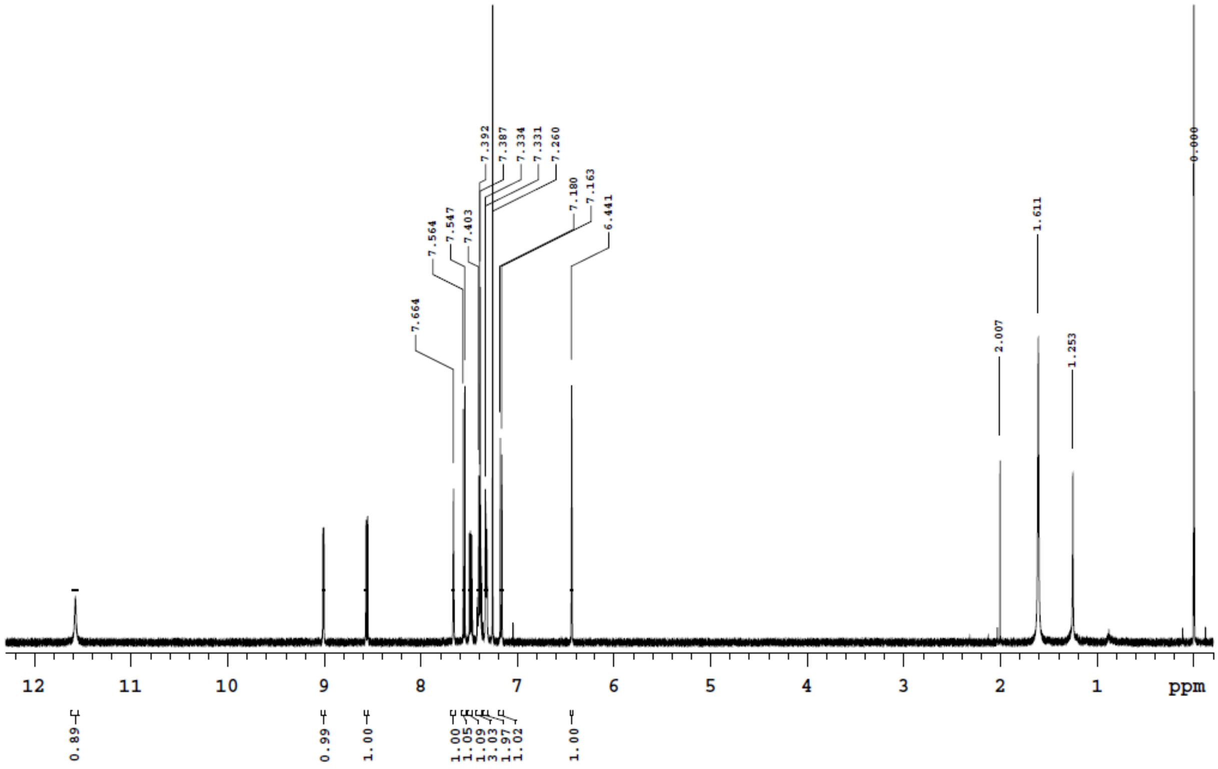

Fig. S50a. ^{1}H NMR spectrum of $[Re(CO)_3(ClQ)HPhpz]$ (**12**) in $CDCl_3$.

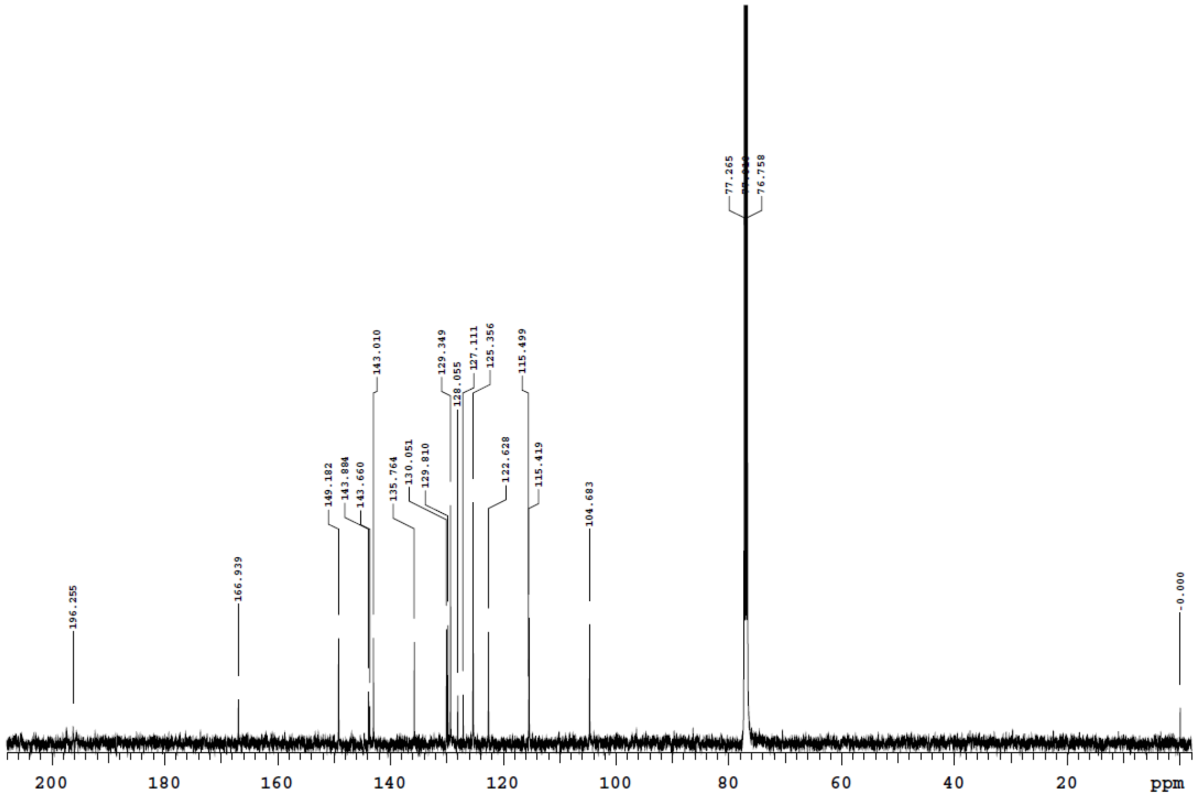

Fig. S50b. ^{13}C NMR spectrum of $[Re(CO)_3(ClQ)HPhpz]$ (**12**) in $CDCl_3$.
